# NHEJ and HDR can occur simultaneously during gene integration into the genome of *Aspergillus niger*

**DOI:** 10.1186/s40694-024-00180-7

**Published:** 2024-08-05

**Authors:** Susanne Fritsche, Aline Reinfurt, Felix Fronek, Matthias G. Steiger

**Affiliations:** 1 Austrian Centre of Industrial Biotechnology, Muthgasse 18, Vienna, Austria; 2 Institute of Chemical, Environmental and Bioscience Engineering, Research Group Biochemistry, Technische Universität Wien, Gumpendorferstrasse 1A, Vienna, 1060 Austria

**Keywords:** DNA repair, CRISPR/Cas9-mediated genome editing, DNA modification, Homology-directed repair, Non-homologous end joining, Self-replicating plasmid, Palindrome

## Abstract

Non-homologous end joining (NHEJ) and homology-directed repair (HDR) are two mechanisms in filamentous fungi to repair DNA damages. NHEJ is the dominant response pathway to rapidly join DNA double-strand breaks, but often leads to insertions or deletions. On the other hand, HDR is more precise and utilizes a homologous DNA template to restore the damaged sequence. Both types are exploited in genetic engineering approaches ranging from knock-out mutations to precise sequence modifications.

In this study, we evaluated the efficiency of an HDR based gene integration system designed for the *pyrG* locus of *Aspergillus niger*. While gene integration was achieved at a rate of 91.4%, we also discovered a mixed-type repair (MTR) mechanism with simultaneous repair of a Cas9-mediated double-strand break by both NHEJ and HDR. In 20.3% of the analyzed transformants the donor DNA was integrated by NHEJ at the 3’ end and by HDR at the 5’ end of the double-strand break. Furthermore, sequencing of the locus revealed different DNA repair mechanisms at the site of the NHEJ event.

Together, the results support the applicability of the genome integration system and a novel DNA repair type with implication on the diversity of genetic modifications in filamentous fungi.

**Supplementary Information:**

The online version contains supplementary material available at 10.1186/s40694-024-00180-7.

## Introduction

The filamentous fungus *Aspergillus niger* is an important cell factory in the biotech industry, which is used to produce organic acids such as citric and gluconic acid, as well as proteins, like glucoamylase or phytase [1, 2]. Genetic engineering offers a powerful approach to enhance filamentous fungi in terms of their productivity as well as to minimize undesirable traits like side-product formation. These engineering approaches started in fungi by transforming plasmid [3, 4] or cosmid DNA [5], which was introduced into the genome, relying on ectopic genome integration through non-homologous end joining (NHEJ). Additionally, homology-directed repair (HDR) using typically linear expression cassettes with homologous 5´ and 3´ flanking regions was employed for genome deletions [6]. In *Saccharomyces cerevisiae*, DNA double-strand break repair is facilitated primarily via homologous recombination (HR) [7] and is mediated by the RAD52 epistasis group: RAD50, RAD51, RAD52, RAD54, RAD55, RAD57, RAD59, RFA1, MRE11, XRS2, and RDH54/TID1. This group of genes is highly conserved amongst eukaryotes, including *A. niger* [4, 8, 9]. However, the occurrence of HR events in *A. niger* was found to be low, standing at 1.78–7%, with NHEJ appearing to be the primary DNA repair mechanism [4, 9, 10]. The introduction of specific genetic modifications in *A. niger* is a challenging task due to this factor. Upon the deletion of NHEJ factors Ku70 (*kusA* in *A. niger*) and Ku80 (*kusB* in *A. niger*), the occurrence of HR significantly increased by more than 80% [9, 10]. Through this, site-specific gene editing became more accessible, and NHEJ-deficient strains are thus used by many groups as a tool to enable targeted gene engineering. One advantage is that transformant strains obtained in a NHEJ-deficient background have a lower mutation prevalence compared to transformants obtained in the wild-type background, which enables the generation of strains with less off-target mutations [11]. Normal growth and conidiation efficieny are reported for NHEJ-deficient strains, but it should be considered that such strains have a limited capacity for double-strand break repair and are consequently more sensitive to stress conditions creating strand breaks such as UV and X-rays [9]. Furthermore, NHEJ-deficient strains showed a slightly higher mutation rate than wild-type strains upon frequent passaging, suggesting that NHEJ contributes to genome stability [12]. A homologous transformation system for *A. niger* based on the *pyrG* gene was described by van Hartingsveldt et al., 1987 [13]. It was found that the transformation frequency based on the *pyrG* gene was at least tenfold higher than the heterologous transformation system for *A. niger* using the *amdS* gene and *argB* gene of *Aspergillus nidulans*, and the *pyr4* gene of *Neurospora crassa* [13–15].

Nødvig et al. [16] were the first to apply CRISPR/Cas9 [17] to several species of *Aspergillus* including *A. niger*. Since then, the usage of this system has been adapted and improved, for example, by finding suitable promoters for guide RNA expression based on 5 S RNA [18] or tRNA promoters [19] and the topic was well reviewed [20, 21]. In addition to CRISPR/Cas9 systems, alternative gene editing and integration tools for filamentous fungi are available. These include systems based on the Cre-*loxP* system involving site-specific recombination events [22–24] and the FLP/FRT system, which, similar to the Cre-*loxP* system, relies on site-specific recombination events [25]. Both strategies are recognized as efficient genetic engineering tools. However, the insertion of specific recombination sites into the genome is necessary and therefore not scarless.

In 2017 we proposed a toolkit for metabolic pathway construction and genetic engineering in *A. niger* [26]. This system consists of a modular vector construction system called GoldenMOCS, based on the Golden Gate cloning approach [27], and a gene integration system for *A. niger* using CRISPR/Cas9 and self-replicating plasmids. The GoldenMOCS platform enables the versatile integration of host-specific parts such as promoters, terminators and resistance cassettes, replication origins or genome integration loci to customize the plasmid to the needs of the experiment and the host cell to be modified. Parts libraries for other organisms like *Pichia pastoris* and *Yarrowia lipolytica* are available [28, 29]. The fungal gene integration system uses a *pyrG* split-marker approach in combination with a transient Cas9 expression to enable selection on the integration event. For this approach, two plasmids are used that can be constructed with the GoldenMOCS pipeline: a Cas9/sgRNA-containing plasmid and a plasmid containing the integration cassette. The plasmids are co-transfomed into *A. niger* and can be transiently maintained in the fungal host using a size-reduced AMA1 version, which is readily lost after hygromycin selection is stopped. A special feature of the system is that the linear integration cassette can be released from the integration plasmid in vivo by Cas9 thereby the same gRNA/Cas9 complex is used to cut the plasmid and the genomic *pyrG* locus.

Upon successful homologous recombination of the *pyrG* split-marker, uridine prototrophy is restored, which is exploited as a selection marker for the integration event. Due to the modular character of the GoldenMOCS system up to eight different expression cassettes can be integrated into the *pyrG* locus using this strategy. The strains obtained in this way most likely have the cassette correctly integrated into the *pyrG* locus, with a minimal screening effort [30].

In this study, we evaluate this integration system and its HDR efficiency at the targeted *pyrG* locus of *A. niger* and can confirm the previously reported high targeting efficiency of the system. In addition, we observed a novel mixed-type repair (MTR) mechanism in which the predicted double-strand break mediated by CRISPR/Cas9 was simultaneously repaired by HDR and on the other side of the integration cassette by NHEJ.

## Materials and methods

### Strains

*A. niger* strains ATCC 1015 [31] and the derivative industrial strain ACIB1 were used as parental strains for genomic integration studies. Parental strains were transformed with pCAS_gpyrG1 according to the protocol of Sarkari et al. [26] to obtain *A. niger* strains ATCC 1015 pyrG^m1^ and ACIB1 pyrG^m1^, respectively.

### Plasmid construction and proliferation in *E. coli*

The Golden Gate cloning system [32] was employed for plasmid construction. Vectors for gene integration at the *pyrG* locus (Additional File 1: Table S1) were assembled following the protocol outlined by Sarkari et al. [26] and harbored the integration cassettes with the sequences listed in Additional File 1: Text S1. Plasmid proliferation was performed in *E. coli* Top10, with transformants cultivated on LB agar supplemented with 50 µg/mL kanamycin, 100 µg/mL ampicillin, or 100 µg/mL hygromycin B.

### Transformation in *A. niger* and PCR verification

Protoplast transformations of *A. niger* strains was conducted as previously described [33] using 0.4 mg/mL VinoTaste (Novozymes, Bagsværd, Denmark) in SMC as lysing enzymes. Transformants were selected on minimal medium plates containing 200 µg/mL hygromycin B. Purification of transformants involved three rounds of single colony isolation on selection medium: one round with hygromycin B (100 µg/mL) and two rounds on minimal medium alone. To verify the resulting transformants, three PCRs were performed on the genomic DNA using Q5 Polymerase (New England Biolabs, Ipswich, Massachusetts, USA). The primers used for the verification PCRs are listed in Additional File 1: Table S2.

### Sequence analysis

PCR products from gDNA were purified from a gel using HiYield PCR Clean-up/Gel Extraction Kit following the manufacture’s protocol. Sanger sequencing was performed by Microsynth, Balgach, Switzerland, using primers listed in Additional File 1: Table S2. For PCR verification primers of sets A – E were selected depending on the integrated cassette and are assigned in Additional File 2: Table S3. Sequence analysis was performed using QIAGEN CLC Main Workbench 21.

## Results

### Evaluation of an integration system confirms a > 90% gene integration efficiency

To evaluate the genomic integration efficiency of the system introduced by Sarkari et al. [26], 33 different integration cassettes with sizes from 3556 to 8898 bp were transformed. The cassettes differed in promoter and coding sequences while the terminator sequence, the homologous arms for HDR of the targeted *pyrG* locus, and the transformation vector remained the same (Additional File 2: Table S3).

In total, the integration profile at the *pyrG* locus of 140 transformants was analyzed by three PCR reactions: PCR1 amplified the fragment from the *pyrG* 5’ region to the integration cassette. With PCR2, a fragment ranging from the integration cassette to the *pyrG* 3’ region was obtained and PCR3 covered the entire *pyrG* locus. Table 1 provides a classification of the integration events based on the PCR results. In summary, in 128 of 140 tested transformants, the genomic integration of the cassette was confirmed. Twelve potential transformants were excluded from the analysis: For five transformants a wild-type fragment and a fragment conforming integration was obtained. These transformants were considered to be heterokaryons or mixed colonies. For three strains it was not possible to obtain all test PCRs and four strains showed the wild-type PCR fragments. Probably due to contamination with uridine prototrophic wild-type strains. Overall, an integration efficiency of 91.4% could be achieved.

Interestingly, transformants with cassette integration could be divided into two groups depending on the PCR result: In 102 transformants, the fragment length after PCRs of the integration site was as expected, indicating targeted HDR on both sites of the cassette. However, in 26 cases (20.3%) PCR1 and PCR3 showed fragments longer than expected. While PCR2 showed the expected amplicon length in all 26 cases, indicating correct HDR on the 5’ end of the double-strand break. PCR results of clones derived from transformation with two different integration vectors are presented in Additional File 2: Figure S1.

**Table 1:** Genomic integration efficiency of *A. niger* strains

PCR verification result	Number of transformants	%
Transformants screened	140	100
Cassette integration at *pyrG* locus	128	91.4
Heterokaryotic or mixed colony	5	3.6
Non-conclusive PCR result	3	2.1
No cassette integration	4	2.9

To explain the unexpected mutation outcome, the DNA profile at the 3’ end of the CRISPR/Cas9 mediated double-strand break was analyzed by Sanger sequencing.

### Simultaneous repair of a double-strand break by both non-homologous end joining and homologous recombination

**Fig. 1:**
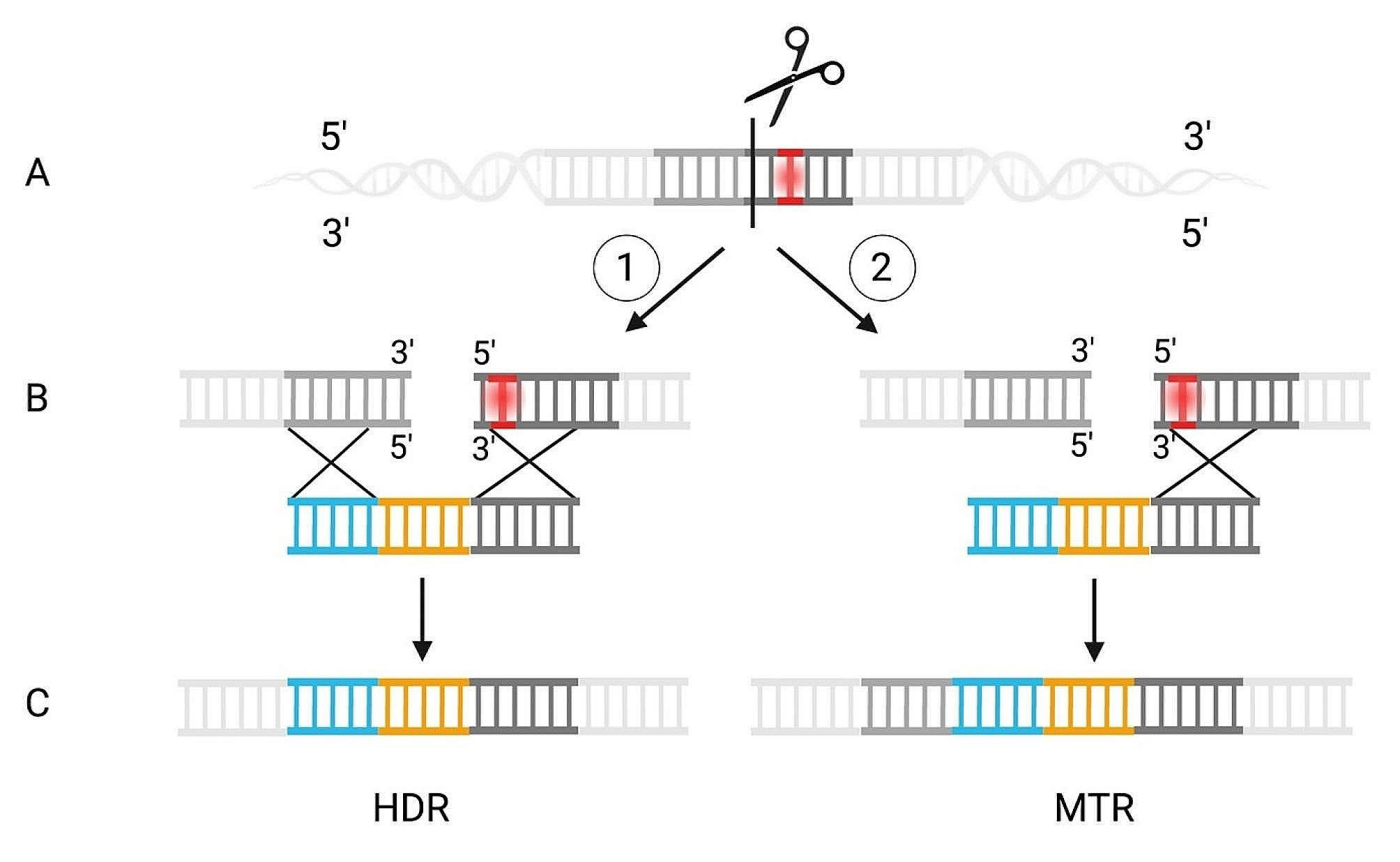
Repair mechanisms of a double-strand break by homology-directed repair and mixed-type repair in *A. niger*. (**A**) The 5’ region of *pyrG* is highlighted in light grey and the *pyrG* gene in dark grey. A mutation in the *pyrG* gene (red dot) leads to a uridine auxotroph strain. The cutting site of Cas9 is highlighted by the black line. (**B**) The double-strand break results in a 3’ end and a 5’ end and is upstream from the mutation of the *pyrG* gene. The donor template (orange) has flanks designed for a double crossover event. The blue flank is the sequence of the 5’ region, and the dark grey flank is homologous to the *pyrG* gene, restoring the mutation. (**C**) The first integration mechanism is a homology-directed repair (HDR). The second integration form is a mixed-type repair (MTR) with a non-homologous end joining (NHEJ) event at the 3’ end and a HDR at the 5’ end of the double-strand break, respectively. The *pyrG* locus is restored for the direct selection of positive integration transformants on minimal medium without uridine

Analysis of the genome integration site revealed two distinct integration events of the cassettes during repair of the double-strand break, as shown in Fig. 1. Initially, the Cas9-sgRNA complex facilitated a cut at the *pyrG* gene upstream from the nonsense mutation (Fig. 1A). The co-transformed donor DNA was flanked with homologous arms on both sides of the integration cassette (Fig. 1B). In one class of analyzed transformants, the homologous arms flanking the DNA fragment facilitated the expected HDR of the lesion by a double cross-over event. In the second class, representing 20.3% of the transformants, the DNA fragment was inserted by a distinct repair mechanism at each site of the double-strand break: At the 5’ end, the DNA fragment was introduced as expected by homologous recombination, thus the INDEL mutation was repaired resulting in uridine prototrophy. However, the 5’-flanking sequence of the DNA fragment was inserted by NHEJ (Fig. 1C). Because of the simultaneous occurrence of both repair mechanisms, NHEJ and HDR, we refer to a MTR mechanism in the following.

**Fig. 2:**
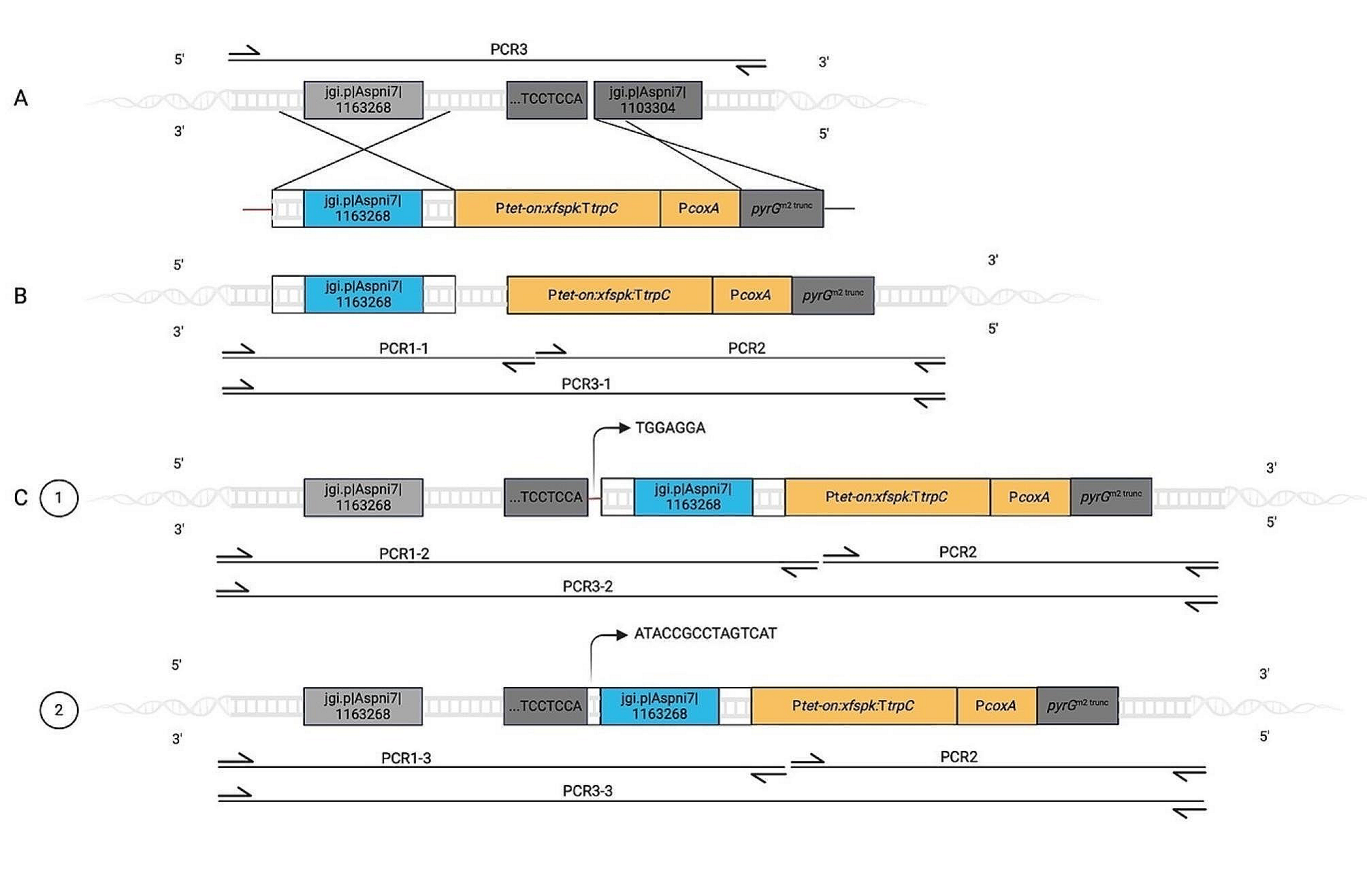
Integration of an inducible expression cassette at the *pyrG* locus by HDR and variants of MTR. (**A**) Promoter region (light grey) and CDS (dark grey) of *pyrG* with Cas9-mediated double-strand break. PCR3 of the parental strain *A. niger* pyrG^m1^ gives an amplicon with 4152 bp. The donor fragment consists of a 5’ flank homologous arm (blue), an inducible cassette for heterologous expression of the phosphoketolase Xfspk, followed by the promoter *coxA* (orange). The 3’ flanking sequence is a truncated *pyrG* CDS (dark grey). (**B**) Integration via HDR was verified by PCR1-1, PCR2, and PCR3-1 with amplicon sizes of 3793, 6841, and 10,692 bp, respectively. (**C**) Integration via MTR with NHEJ on the 3’ end and HDR at the 5’ end of the double-strand break. In clone 1, NHEJ of sequences form a palindrome. In clone 2, the donor DNA is shortened by end resection prior to the NHEJ event. PCR1-2 and 3 − 2 are 5085 bp and 11,984 bp for variant 1 and 4974 and 11,873 bp for variant 2

A detailed description of MTR is provided for the integration of an inducible expression cassette at the *pyrG* locus of *A. niger* ATCC 1015. The core of the integration construct consists of a tet-on promoter [34] and the heterologous coding sequence of Xfspk, a phosphoketolase from *Bifidobacterium longum* [35], that is followed by the *trpC* terminator. Upstream, the cassette is flanked with the homologous sequence of the *pyrG* promoter region, consisting of the coding sequence of jgi.p7⎪Aspni7⎪1163268 and 119 bp and 73 bp of its 5’ and 3’ region, respectively. Downstream of the cassette, the sequence is a truncated version of the *pyrG* gene (*pyrG*^m2,trunc^, 679 bp) under the control of the *coxA* promoter. *PyrG*^m2,trunc^ is homologous to the genomic sequence after where the Cas9 has facilitated the double-strand break [17]. The integration system is designed to facilitate a double-strand break after the seventh base pair of the startcodon ATG of the *pyrG* gene. Notably, the 3’ end of the genomic dsDNA ends with the nucleotides 5’-TCCTCCA (Fig. 2A).

Three individual clones transformed with the expression cassette *tet-on:xfspk:trpC* were analyzed: In the first case, the integration cassette recombined with the genomic DNA by the expected double cross-over event, thus restoring the uridine prototrophy. The successful integration was verified by three PCRs. PCR1-1 amplified 3793 bp upstream of the homologous region to the terminator *crgA* of the tet-on transactivator rtTA2S-M2. PCR2 covered 6841 bp from the tetO7 promoter to the region downstream of *PyrG*^m2,trunc^. PCR3-1 covered the entire integration cassette, including the respective up- and downstream regions (Fig. 2B), which are 10,692 bp.

In the second case, the expression cassette was integrated by MTR with two observed variations (Fig. 2C). Both showed insertion of the cassette by homologous recombination of *pyrG*^m2,trunc^ at the 5’ end of the double-strand break. The 3’ end, however, was repaired by end joining mechanisms:

In clone 1, ending nucleotides of the additionally integrated 5’ homologous arm were 5’-TGGAGGA. This forms a palindromic sequence with 5’-TCCTCCA that are ending nucleotides at the double-strand break mediated by Cas9. Sequences are joined, and the amplicon of PCR1 is 1292 bp longer compared to the region after repair by homologous recombination.

In clone 2, the 5’ end of the flanking arm of the donor DNA was shortened by 111 bp, and the ending nucleotides were 5’-ATACCGCCTAGTCAT. The double-strand break in the genome at the 3’ end was then repaired by NHEJ of the donor sequence. PCR1 thus amplified a fragment that is 1181 bp longer compared to the locus after HDR.

## Discussion

There are two major mechanisms for rejoining double-strand breaks in the filamentous fungus *A. niger*, namely NHEJ and HDR. HDR leads to accurate repair of DNA damages by end resection to generate single-stranded DNA overhangs for the recombination event. The NHEJ pathway, however, suppresses end resection and promotes ligation of DNA strands. It is often accompanied by insertions or deletions (INDEL) at the repair site and it is the predominant fungal DNA damage response pathway [36, 37]. In genetic engineering, HDR enables the introduction of precise genetic changes by the insertion of desired DNA sequences. NHEJ, on the other hand, allows efficient gene deactivations by the introduction of random mutations. So far, it is known that either NHEJ or HDR facilitates the repair of a DNA double-strand break in the genome. However, there is evidence that both pathways are activated concomitantly to provide genome integrity [38].

Over time, various genome-editing methods have been described, and the advancement of CRISPR/Cas9-based systems has profoundly influenced modern genome engineering. The technology has been constantly developed and provides a tool base for enabling precise changes of DNA at a specific locus. On the other hand, the availability of various DNA repair mechanisms to the organisms and unknown molecular interaction has triggered unintended DNA modifications [39]. In filamentous fungi, these unexpected outcomes were reported as large deletions of off-target genes [40] or the action of multiple DNA repair pathways on the targeted locus [41].

Here, we report the discovery of a DNA repair mechanism initiated by a CRISPR/Cas9 integration tool designed for the *pyrG* locus that is linked to a selection system. The concept requires HDR of one end of the double-strand break but eventually allows other repair pathways on the second end of the DNA lesion, providing a unique opportunity to observe different types of repair mechanisms at this site. We observe a novel MTR mechanism and describe the simultaneous DNA damage response of NHEJ and HDR, each acting on one particular end of a double-strand break. The 5’ end was always repaired by HDR which is enforced by the selection system for uridine prototrophy. In contrast, the 3’ end in 20.3% of observed integration events was repaired by NHEJ. If a homologous repair template is present that leads to a scarless genomic sequence after the integration event, it is difficult to analyze whether the double-strand break is mainly caused by Cas9 or triggered in an untargeted manner by radiation or chemicals. In the case of MTR, however, one site of the double-strand break was repaired by NHEJ, so that the genomic sequence retains the information about the original site of the double-strand break, which in this case was exactly three base pairs upstream of the PAM site [17]. This shows that Cas9 is specifically cutting the genomic DNA at the *pyrG* site in the two presented cases (Fig. 2). We further demonstrate that DNA strands were either directly joined together or that the respective integration cassette was shortened prior to integration.

**Fig. 3:**
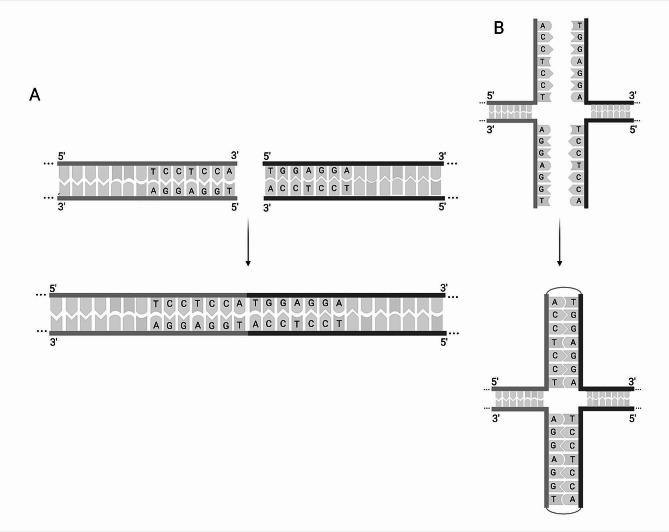
Model of double-strand break repair by formation of a palindrome. (**A**) Two inverted repeats of seven nucleotides (5’-TCCTCCA and 5’-TGGAGGA) are adjacent to one another and after NHEJ form a palindrome. (**B**) Extrusion of double-strands results in branch migration and leads to the formation of four-way junction. Light grey and dark grey DNA strands represent the genomic DNA and the donor DNA, respectively

Generally, Cas9 generates blunt ends at the cutting site [17, 42, 43], which, in our case, is located not only on the specific locus in the genome but also on the integrating plasmid. This facilitates the release of the integration cassette as a linearized DNA fragment after transformation. Because Cas9 cuts 3 nucleotides upstream of the PAM site 5’-AGG, these six nucleotides form the 3’ end of the double-strand break in the genome. In fact, the same nucleotides in an inverted manner remain also at the 5’ end of the homologous arm of the cassette released from the integrating plasmid. Consequently, a palindrome, where the sequence on one strand is the reverse complement of the sequence on the other strand, is formed after the respective strands are joined (Fig. 3A). Presumably, the end joining was mediated by the palindrome sequence itself. The double-strands of each DNA end separate and respective inverted repeats bind and convert to a four-way branch structure as shown in Fig. 3B.

In *A. fumigatus,* it is reported that microhomology-mediated end joining (MMEJ), that employs microhomologous ends flanking the integration cassette, is a highly efficient repair mechanism of CRISPR/Cas9-mediated mutagenesis [44]. Our findings suggest that the utilization of flanks forming a palindrome to the ends of a double-strand break could, therefore, also enhance DNA integration. A similar mechanism proposed as an intermolecular model of palindrome formation was demonstrated in *S. cerevisiae*. Evidence was reported that an in vivo expressed endonuclease releases linear DNA fragments from two transformed plasmids harboring identical short inverted repeats of 42 bp near the cutting site. The findings suggest a 5′ to 3′ resection of DNA ends resulting in 3’ single overhangs that include the respective short inverted repeats. A homologous recombination event then mediates the ligation of the DNA strands [45]. In contrast, our system already generates DNA strands with the inverted repeats at the blunt ends that are joined without previous end resection and therefore suggests NHEJ as the acting mechanism, possibly mediated by the present palindrome. Our second analyzed case supports this outcome where the 5’ homologous arm of the integration cassette was shortened as a result of the NHEJ pathway and subsequently integrated into the genome.

Overall, it still needs to be elucidated why NHEJ was favored over a HDR at this site. One possible explanation is the simultaneous activation of the HDR and NHEJ pathway and the design of the *pyrG* repair fragment. The crossing-over event with the truncated version of the *pyrG* (*pyrG*^m2,trunc^) simultaneously integrates the *pcoxA* promoter and ensures growth without uridine after this DNA damage response. The precise DNA repair on the 3’ end of the double-strand-break, which is the promoter region of *pyrG*, is, however, not essential for growth. Therefore, NHEJ, the predominant form of double-strand break repair, can compete with HDR on this side of the DNA lesion. It is also likely that there will be competition for the factors that are recruited at the targeted integration site. At the decision point, the palindrome might increase the probability of repair by NHEJ which leads to MTR. In this respect, the prevention of a palindrome formation may be desired, which is in-line with the use of NHEJ deficient backgrounds for strain generation, which have the advantage to promote homologous recombination and acquire fewer mutations during the transformation procedure [11]. On the other hand, the formation of short palindrome sequences could be used as a new tool for the integration at a specific locus facilitated by NHEJ instead of using long homologous arms used for HDR.

## Conclusion and outlook

The evaluation of the transformation system by Sarkari et al., 2017 confirmed that this integration pipeline enables efficient and flexible introduction of different expression cassettes at the *pyrG* locus of *A. niger* strains. It was also observed that the CRISPR/Cas9-mediated double-strand break can be repaired with a template DNA simultaneously by NHEJ and HDR pathways which is referred to as MTR. The MTR mechanism allows for an additional damage response through NHEJ, ensuring the stabilization of the double-strand break, while HDR, operating in parallel, provides the accuracy to repair the mutated *pyrG* gene. The results emphasize the need to further understand factors influencing MTR, especially the impact of the palindrome on the genome repair mechanism.

### Electronic supplementary material

Below is the link to the electronic supplementary material.

Supplementary Material 1

Supplementary Material 2

## Data Availability

All data generated or analysed during this study are included in the manuscript and its supplementary information files. The industrial strain ACIB1 is not publicly available.
