# Supplementary material for: NHEJ and HDR can occur simultaneously during gene integration into the genome of Aspergillus niger
Source: Fungal Biol Biotechnol. 2024 Aug 5;11:10. doi: 10.1186/s40694-024-00180-7 (PMC11301975; doi:10.1186/s40694-024-00180-7)
Supplement: Supplementary file 1 — Supplementary Material 1 [file 40694_2024_180_MOESM1_ESM.pdf]

Supplementary Table S1 Vectors used for transformation of integration cassettes.

**Name**	**Description**	**Addgene reference**
pCAS_gpyrG1 (pMST621-BB3_gpyrG1_cas9)	CRISPR plasmid with gRNA for pyrG1, Cas9	90276
pCAS_gpyrG2 (pMST620-BB3_gpyrG2_cas9)	CRISPR plasmid with gRNA for pyrG2, Cas9	90277
	BB3 recipient for homologous integration in pyrG locus with linker AC	90280
pMST1212 - BB2_E_BC_pcoxA:pyrGtrunc_AMA1_2.8	BB2 with split pyrG marker for homologous integration with linker BC (for 1 expression cassette)	90286
pMST1213 - BB2_E_CD_pcoxA:pyrGtrunc_AMA1_2.8	BB2 with split pyrG marker for homologous integration with linker BC (for 2 expression cassettes)	90287
pMST636_BB3_L_AD_hyg_KanOri_pyrG5′	BB3 recipient for homologous integration in pyrG locus with linker AD	90281

Supplementary Text S1 Nucleotide sequences of integration cassettes derived from transformation vectors (pAR and pSF). Cas9 from co-transformed plasmid pMST620-BB3_gpyrG2_cas9 cuts three nucleotides upstream from the PAM sequence resulting in the following linear DNA fragments:

>pAH035_IC
TGGAGGaAGGGATCCGCCAAGGATGCTCTGGATCCGCATCCGGCCGCTCTTGCGCCCCATCAATCGCCCGACTATAAATCGAACTACTTTCGGCATCTTCTAGACTTCCTA
ATACCGCCTAGTCATAGCAGATTCAAGCTGAGAACACCACAAGTAAATATCACCCATCATGCTTACCCTGACCGTCCCTGAAAACTACGGGTATGTGCCAATTCTACAATT
CCTTGCAGACAATGCCATTCTCCCCATGAAGTCTGATGCTAACTATCCTGCAGCTCTGTCATTGCCGTCGCTCTGGGTGCCATCCCCGTCCTGAGCTTCGTCCATGGCGCCG
TCGTGTCTCGTCTCCGCAAGGAAGCTGATTGCCCCTACCCTCACTGCTATGCGACCGTAGAGCAGTGCAAGACCAACGTAAGCCAACCTCACACAAACAGGATTCCTCGA
GCTAACATACATTCCGAACCGTGCAGCCCAAGGCCGAGCAGTTCAACTGCGCTCAGCGCGCTCATGCCAACTTCCTTGAGAACTCCAGCCAAACTATGCTCTTCCTCCTGG
TAGCTGGACTGAAGTACCCCCAGTTGGCGACTGGCCTCGGAAGCATCTGGGTCCTCGGTCGCTCACTGTTCCTTTACGGATATGTGTACTCCGGCAAGCCGCGGGGTCGCG
GTCGTTTGTACGGCAGCTTCTACTTGCTTGCACAGGGAGCTCTCTGGGGCTTGACGTCTTTTGGAGTTGCGAGGGAGTTGATTTCCTACTTCTAAGTTTGGACTGAATCCGT
GGTGTGATTGAGGTGATTGGCGATGTTTGGCTATACCAGCTATATGTAATAATCTCTACTGTATACTACTATTCAACGCATTTTACTATGCGTGCTGCTAGGGTCGGCAATG
ACAATGGCAATCTGACTGACGTGGTCTATTTCTCCATGTGCAGCAGGGAATACGAGCTCCAATGGACCTCGGGAGTGGCACAGTCAATGGCAAGGAAACTGGAGcgcaaaaaa
ccccgcccctgacagggcggggttttttcgcGATCGGAGGTCTTTCAGGTTGTTGAGCTGCTCGACAGCCACCTCAAATATTTCAGGAACGAGTTTTCGACAGGTTTCGGCGTACTTGGCCGA
CGTCAAGGCCAACCGGGATTGCGTTTGAACGAGCATGTCTGCGAGTCGATGCTTCGGCTGTGCAGGGTCCAGGGCAAGAAATGCTCCACCGGCTTGGAGAACGGCAAGC
ATAGAGACGATGGTCCAGCATGACTTGTCGAAATACAATGGGACGATCTCGTCGGGACCGATGCCGTGGTTCACCAGATATCCGGCCAGGCGCCGGACGAGCCTGTCTAG
CTCGCCGTAGGTCAACTCGCCATCCCAAGCACTGATAGCCAGCGAGTGCGGCTGCGATGCCGCCCACCCCAAGATCAAACCATGGGCAGTCGCTCTGACCTCGGGAATGA
CGGGCTGACTCCAAGGCCATGTCTGGCAGGTTTCCTCGGTGTGGATTCGGTCAGACATGATAGAAAGTCAAATCCTAGGAGGCCTTGTTGGTATTTTCTCTGTGTATTGGG
TATTCCAGGACCCTTTTAAACACTTAAAAGAAAGCCTGACCCCTCAGCCTTCAACAGTACTCCCAGGGAGGGGCTGAGtCCTGAATGGAGAAATTGCAAAAGACAAAACA
AATTAATTACATACCTTACCAACAGATACACACTGGTGCGGCTCGAGAGGCTTGGTTGCATGCAACTACGATCTTTCGCAAAACCTCAAAAGGCGATCTGGCAGACTAGG
ACATGCTGCATTAGCCATTTCTCTTCTCTCCACTGTGGCCTGATCTTTGCGAGCTGAGACTTCTCGTGGTCAAGAGCTGCCATCATGTAATATGCCGCATAGCCGGTCCCCC
ACCTCAGCTTTATAAAGGGAGTTTGACATCACCCTGAAGTATAAACCCCTCTATCCCCTGGTAGATTTCGATTAAACAAACTGCGAACAAGTAGCAGAATCCAACCTGCA
TGTCcTCAACCACGTCcTCATCAAGATCAGACCTTGAAAAGGTCCCCGTACCACAGGTCACCCCTAGAGACAGTGACTCCGATAAGGGATCCCTCTCTCCGGAGCCTTCGA
CCCTCGAGGCTCAGTCATCCGAGAAGCCACCGCATCATATCTTCACACGaTCTCGCAAGCTGCAAATGGTTTGCATCGTCTCCCTCGCTGCCATATTTTCTCCGCTTTCGTC
GAACATTTACTTCCCTGCCCTGGATGATGTCTCGAAATCCCTCAACATCAGCATGTCGCTCGCAACACTCACCATCACGGTGTACATGATCGTCCAAGGCCTCGCTCCCAG
CTTCTGGGGTTCCATGTCAGACGCCACAGGTAGACGGCCTGTCTTTATTGGAACATTCATTGTTTACCTCGTAGCCAATATTGCTCTGGCCGAATCCAAGAACTATGGTGA
GCTCATGGCCTTCCGAGCCTTGCAGGCTGCTGGTAGCGCAGCCACCATCTCAATCGGAGCTGGAGTGATTGGTGATATCACAAACTCGGAAGAAAGAGGTAGCTTGGTGG
GTATCTTCGGTGGAGTTCGCATGCTTGGACAGGGAATCGGGCCGGTTTTCGGCGGCATTTTCACCCAGTATCTCGGATATCGATCTATCTTTTGGTTCCTCACGATTGCTGG
AGGCGTGAGTCTCCTGTCCATTCTGGTGCTTCTTCCGGAGACATTGAGgCCAATTGCTGGAAATGGAACTGTGAAGCTCAATGGCATTCATAAGCCCTTCATCTACACGAT
CACCGGCCAGACGGGGGTTGTCGAGGGAGCGCAACCGGAAGCGAAAAAGACCAAAACCAGCTGGAAGTCTGTTTTTGCTCCTTTGACATTCCTCGTCGAAAAGGACGTTT
TCATCACCCTGTTCTTTGGAAGTATCGTGTACACAGTGTGGAGCATGGTGACATCCAGTACCACCGACCTCTTCAGCGAAGTGTACGGCCTGTCATCCCTGGACATTGGAC
TCACTTTCCTAGGCAATGGCTTTGGATGTATGTCTGGCTCTTATCTGGTCGGCTACCTTATGGATTACAACCACCGTCTTACCGAACGCGAATATTGCGAGAAACACGGTT
ATCCGGCAGGCACACGTGTCAATCTGAAATCACACCCCGACTTCCCCATTGAGGTCGCCCGGATGCGCAATACCTGGTGGGTGATTGCGATCTTCATCGTGACAGTTGCTT
TGTACGGCGTGTCTTTGCGGACACATCTGGCGGTGCCTATCATTCTGCAGTACTTCATTGCGTTCTGCTCAACAGGACTCTTCACCATCAACAGCGCCCTGGTCATCGATCT
TTACCCAGGTGCTAGCGCCAGTGCGACAGCAGTGAACAATCTGATGCGGTGCCTGCTTGGAGCTGGCGGTGTGGCTATCGTGCAACCTATCCTGGACGCCTTGAAGCCGG
ATTATACTTTCCTCTTGCTTGCCGGCATCACCCTTGTGATGACTCCGTTGCTGTACGTCGAAGATCGATGGGGTCCTGGCTGGCGACATGCCCGCGAAAGGAGACTCAAGG
CCAAAGCCAACGGCAACTAGGCTTGGAGAGAAAGGACTTGAAAAAAAAAAGGTGAAGTGGGACTGGTGAAGTAAATGTTTGATTCTTCCCCACACTTTCTTGATATGGG
TTTTATTTTAGCGGCATTTGGCGATACCACTGTTTTGCAAGCGATACCAGTTAGATTTATAGAAGAATTGATCAGTTTCATCGGCTATCTCGCTATTACTTCCTCGTAGCTTT
TTAGCTTCATATATGGGTGAGTGGAAAGGTTTGACATGGTGGTTGAGATAGTATCATTTGGATGGAGATGGAAGTATAAAGCAAGAAGTTCTGTTGTTTGGTGTTTAGAC
TAGACTAGACGTGGCCGGTGAAGTCACGAGCCTCAGCTTAGATAATCTAATGCGGCTGATGTGACTGACCGATGGCTTTGTTATATGAgGACAGGTCGGTCAATTAGAAG
ATGTCGCCAGCAAAAGGGTATAATAATGGCTATTATAGAGATAAGAATGTAGAGTATCCCTGTTTTTAAGGATCAGTACGTAGTTTTACTATAAAGGTACCCATATATATA
TTTGCGGAACACGAGGAGGTCGGGAGTACATACTTGTAGTTACTGTTTGTTTGTGACGAGGAATGCCAGTTAGGTAAGCACCACGTGATGCGTGACCCAAACAAGCGCCC
GGTCGTTGCTTGAGGAACCAATTTCTCTGTTTATCCTCCTTCGAACACCACACTTCGACCTTCTGGACACTGCCGCTATGAGCCTTCCATAGCCAGCCTGTTTCAGCTCGAC
AACACCTCTTGATGCCTTGACGACAACTCATCAACTTCTAGCTCCGTTGGTTCACGTTTTTGCTGTCCTTCTTGCGGTGTGGACCTCGTGCGGTCGAGGGTGGCTCCGTGAA
GGATCAAAACAGACGCTATTGCCCGTGGTTACCGATCGTCTCTCACCCAGCAACAAGGAAACCCAGACAGACACTGCACAGCGTAGCAGATCCTAAAAGAGAAAGAGAT
GCCTCCTGAGATGGGTAAGAAGCTGCCCGCTCCGGGGAGACGGGCACAAGGTTCATGAACCGAAGAGGATGGGAACCCCGACCATGGCCTGCGGCCGCAGCTATCAGTC
CTCTCAGCTGACGCCCCTCACGAACCCTCGCACTCTCCCGAAATGCTCTTCGGTGTCCATGGCGCATCATCTCAGCAGCATCCCATGTCAAACCATGGCTTTGGGCCCACG
GACTCTGTGGCCCTGCCGCAACATCACCATCACCATCGACTCCCGCCCCATGCAGCGCTGCGCGCCCCAGGGCGTCATGGAGTGAATGTGGAATCTCCTCCTTTGCCCTCG
GGCCCGCCGAGTGTGGTGGGCCAGCCTGGAATGCCTGATCCAGCCCCCAGGCCTCGAGGACCAAAACTGAAGTTTACTCCCGAAGAGGACGCTCTACTGGTGGAGTTGA
AGGAAAACAAGAACTTGACGTGGAAGCAAATTGCAGACTTCTTCCCGGGCCGAACGAGCGGTACCTTGCAAGTCCGATACTGCACCAAGCTGAAGGCTAAGGATGTAGC
TTGGAGTGACGAAATGGTTCGATTTGCTCCTGATGTATTTCTACGCCTGTCTCACACATGCTAATGAAGGGAATAGGTACAAAGGCTGCAGCGGGCAATGCACGAGTACG
AGAACGATCGGTGGCGCATCATTGCAGGGAAGGTTGGAAATGGCTTCACCCCAGCTGCTTGCCGCGAGAAAGCCATGCAGCTCCATGAGTAAAAGCGTTGGGGAATTTTC
ATATTTATATCTACTGTCGCCAGATTCGGCCCTGCTTGGACCCTCTGATCTCCTTACTCTCCATATTGGTTCAAATGTCGGGTCACCGATAGGGCTGGTGGTGCAGGCTTGT
TGTAGGCACGGGAGGATGATCAGCATAACTCTGAGTCACTATAGGGACGGGTTGATGTAAGGTATTAAGTGATGTATGATAATTCATTTTAGCCCGGGGGAACATATGGC
GCCGGCATTTGTTCGTTCGCAATGAACCGACACTAGCGTCCGCTCTCGCAGTTTAGCACCGGCTGATCCCGGGCTGAACGCGGCCATTGCTCGGCCGGGGCATGTGTTCCT
TATCTACGGCAGACCGCAGATGACCACTGGAGCAGATTATAGACCCTAAGCCCTAAGCCGGACACCCAATCGAGTAGGTCTGCGGACCAGGTCACTGCGGGCAGCCGGA
GAAGCTCCGCAACCAATCAATCCCCGGCGCTGACTAAGGGCAGGCGACCACGGGCCGAAGCGGCTTCAAACTCACCTCAACCTCCAAACTCCCTCATCTCCAAACGTCCT
TGCCTTGTCTGCCGTCATTGCAACCCACCCACCAGGACACATGAGCAGCAAGTCCCAGCTCACCTACACTGCCCGTGCCAGCAAGCATCCCAATGCTCTGGCGAAGAGGC
TGTTCGAGATTGCCGAGGCCAAGAAaACCAATGTGACTGTCTCGGCTGACGTTACCACCACTAAGGAGCTACTAGATCTTGCTGACCGTCTCGGTCCCTACATTGCCGTGA
TCAAAACCCACATCGATATCCTCTCTGATTTCAGCAACGAGACtATTGAGGGACTTAAGGCTCTCGCGCAGAAGCACAACTTTCTCATCTTCGAGGACCGCAAGTTCATTG
ACATCGGCAACACGGTCCAGAAGCAATACCACGGCGGTACCCTCCGTATCTCGGAATGGGCCCACATCATCAACTGCAGCATTCTCCCTGGTGAGGGTATCGTCGAGGCT
CTCGCTCAGACGGCGTCTGCACCGGACTTCGCCTACGGCCCCGAACGCGGTCTGTTGATCTTGGCAGAGATGACCTCTAAGGGCTCCTTGGCTACCGGCCAGTACACTACT
TCCTCGGTCGATTATGCCCGGAAATACAAGAACTTCGTTATGGGATTCGTGTCGACGCGCGCGTTGGGTGAGGTGCAGTCGGAAGTCAGCTCTCCTTCGGATGAGGAGGA
CTTTGTGGTtTTCACGACTGGTGTGAACATTAACCTCCA

>pAH025_IC
TGGAGGaAGGGATCCGCCAAGGATGCTCTGGATCCGCATCCGGCCGCTCTTGCGCCCCATCAATCGCCCGACTATAAATCGAACTACTTTCGGCATCTTCTAGACTTCCTA
ATACCGCCTAGTCATAGCAGATTCAAGCTGAGAACACCACAAGTAAATATCACCCATCATGCTTACCCTGACCGTCCCTGAAAACTACGGGTATGTGCCAATTCTACAATT
CCTTGCAGACAATGCCATTCTCCCCATGAAGTCTGATGCTAACTATCCTGCAGCTCTGTCATTGCCGTCGCTCTGGGTGCCATCCCCGTCCTGAGCTTCGTCCATGGCGCCG
TCGTGTCTCGTCTCCGCAAGGAAGCTGATTGCCCCTACCCTCACTGCTATGCGACCGTAGAGCAGTGCAAGACCAACGTAAGCCAACCTCACACAAACAGGATTCCTCGA
GCTAACATACATTCCGAACCGTGCAGCCCAAGGCCGAGCAGTTCAACTGCGCTCAGCGCGCTCATGCCAACTTCCTTGAGAACTCCAGCCAAACTATGCTCTTCCTCCTGG
TAGCTGGACTGAAGTACCCCCAGTTGGCGACTGGCCTCGGAAGCATCTGGGTCCTCGGTCGCTCACTGTTCCTTTACGGATATGTGTACTCCGGCAAGCCGCGGGGTCGCG
GTCGTTTGTACGGCAGCTTCTACTTGCTTGCACAGGGAGCTCTCTGGGGCTTGACGTCTTTTGGAGTTGCGAGGGAGTTGATTTCCTACTTCTAAGTTTGGACTGAATCCGT
GGTGTGATTGAGGTGATTGGCGATGTTTGGCTATACCAGCTATATGTAATAATCTCTACTGTATACTACTATTCAACGCATTTTACTATGCGTGCTGCTAGGGTCGGCAATG
ACAATGGCAATCTGACTGACGTGGTCTATTTCTCCATGTGCAGCAGGGAATACGAGCTCCAATGGACCTCGGGAGTGGCACAGTCAATGGCAAGGAAACTGGAGcgcaaaaaa
ccccgcccctgacagggcggggttttttcgcGATCGGAGTTTTGTGAGTTcTCTTCAAACAATTTCTACACAGGAGGCCGATGCAAATTTACAATTTCGGTCTTGCAATTGTCGTTGTGCACG
AGTAAGTCAAGTACACAGTAGGAGGCTGAGAGGCAAGCGTTTTGCCATCGGATATCCGGGACCAGATGGAAAGAAGGGGCGGTTCCCCATGGATGGAGCGCGAAGGTGT
GGACTCCAAGAAAACGCTTTGGTTCGGTCAAGACGGGGAAATGGGAGAGGAATGGATATCTTACGAAGCCGAATTACGGAGAAAGGGCCCAAACATGTAAATTATGGCC
TGTAGGTCAATCAGCAAAGGGCCGAGCGGTGATTGCGAGTACACCGATCGGTAGACTACGGAAGGGATGAAAAAGACAGGAAAATAGCGAGAGCTACATGCCGTTTCA
GAGGCTATCGAAATGATATTCCAAAGTATCACCAGTAGCCATAACCACTATAATAAAGGACGAAGATGAGAGTGCCTTCGTTCTCTTTGACCAGAAATTCACTCATAGTA
TCAAAGGGTATTTCCCAATAATGTCAGCGGTCGGAGTTGGTTACTGGCGCGATCGGGAGATATCGGCTCTTCGTTGCCTGGGCCAGCATTAGCGCCGGGTCCAGGTTTTTT
TTTTGCAAATTTTTTTTTCTTTTCCTGGCTATGTTTTTTTTCGTTCCCCCTAACAATGGGAAGGACCTCCCTACTCCGTACCGGGCCAACCAATCCGGCCAATGGAAACTGG
GCCGGGACGCCCATCGCCGCCGCTGCCACTGCAAATTCAGGCCAGCGAAAAACCCAAGAGCGTCCTAGCGTCTCCGCTCGCTTCTTCCCGCTTACAAGAGCCCTTCGCTC
GCTATTTTTTCTTCCCTCCCTTCCCTTCTCTCTTCTTTTCTTTCCATCCCCTTTGAAGTGTCCTGTTTGACTGGCACTATCATCCATCTCCTCTCTTTCTTTCCTTAGTTTTCGTT
CATCACAGCCGTCAAACATGTCcTCAACCACGTCcTCATCAAGATCAGACCTTGAAAAGGTCCCCGTACCACAGGTCACCCCTAGAGACAGTGACTCCGATAAGGGATCCC
TCTCTCCGGAGCCTTCGACCCTCGAGGCTCAGTCATCCGAGAAGCCACCGCATCATATCTTCACACGaTCTCGCAAGCTGCAAATGGTTTGCATCGTCTCCCTCGCTGCCAT
ATTTTCTCCGCTTTCGTCGAACATTTACTTCCCTGCCCTGGATGATGTCTCGAAATCCCTCAACATCAGCATGTCGCTCGCAACACTCACCATCACGGTGTACATGATCGTC
CAAGGCCTCGCTCCCAGCTTCTGGGGTTCCATGTCAGACGCCACAGGTAGACGGCCTGTCTTTATTGGAACATTCATTGTTTACCTCGTAGCCAATATTGCTCTGGCCGAA
TCCAAGAACTATGGTGAGCTCATGGCCTTCCGAGCCTTGCAGGCTGCTGGTAGCGCAGCCACCATCTCAATCGGAGCTGGAGTGATTGGTGATATCACAAACTCGGAAGA
AAGAGGTAGCTTGGTGGGTATCTTCGGTGGAGTTCGCATGCTTGGACAGGGAATCGGGCCGGTTTTCGGCGGCATTTTCACCCAGTATCTCGGATATCGATCTATCTTTTG
GTTCCTCACGATTGCTGGAGGCGTGAGTCTCCTGTCCATTCTGGTGCTTCTTCCGGAGACATTGAGgCCAATTGCTGGAAATGGAACTGTGAAGCTCAATGGCATTCATAA
GCCCTTCATCTACACGATCACCGGCCAGACGGGGGTTGTCGAGGGAGCGCAACCGGAAGCGAAAAAGACCAAAACCAGCTGGAAGTCTGTTTTTGCTCCTTTGACATTCC
TCGTCGAAAAGGACGTTTTCATCACCCTGTTCTTTGGAAGTATCGTGTACACAGTGTGGAGCATGGTGACATCCAGTACCACCGACCTCTTCAGCGAAGTGTACGGCCTGT
CATCCCTGGACATTGGACTCACTTTCCTAGGCAATGGCTTTGGATGTATGTCTGGCTCTTATCTGGTCGGCTACCTTATGGATTACAACCACCGTCTTACCGAACGCGAATA
TTGCGAGAAACACGGTTATCCGGCAGGCACACGTGTCAATCTGAAATCACACCCCGACTTCCCCATTGAGGTCGCCCGGATGCGCAATACCTGGTGGGTGATTGCGATCT
TCATCGTGACAGTTGCTTTGTACGGCGTGTCTTTGCGGACACATCTGGCGGTGCCTATCATTCTGCAGTACTTCATTGCGTTCTGCTCAACAGGACTCTTCACCATCAACAG

CGCCCTGGTCATCGATCTTTACCCAGGTGCTAGCGCCAGTGCGACAGCAGTGAACAATCTGATGCGGTGCCTGCTTGGAGCTGGCGGTGTGGCTATCGTGCAACCTATCCT
GGACGCCTTGAAGCCGGATTATACTTTCCTCTTGCTTGCCGGCATCACCCTTGTGATGACTCCGTTGCTGTACGTCGAAGATCGATGGGGTCCTGGCTGGCGACATGCCCG
CGAAAGGAGACTCAAGGCCAAAGCCAACGGCAACTAGGCTTGGAGAGAAAGGACTTGAAAAAAAAAAGGTGAAGTGGGACTGGTGAAGTAAATGTTTGATTCTTCCCC
ACACTTTCTTGATATGGGTTTTATTTTAGCGGCATTTGGCGATACCACTGTTTTGCAAGCGATACCAGTTAGATTTATAGAAGAATTGATCAGTTTCATCGGCTATCTCGCT
ATTACTTCCTCGTAGCTTTTTAGCTTCATATATGGGTGAGTGGGAAAGGTTTGACATGGTGGTTGAGATAGTATCATTTGGATGGAGATGGAAGTATAAAGCAAGAAGTTC
TGTTGTTTGGTGTTTAGACTAGACTAGACGTGGCCGGTGAAGTCACGAGCCTCAGCTTAGATAATCTAATGCGGCTGATGTGACTGACCGATGGCTTTGTTATATGAgGAC
AGGTCGGTCAATTAGAAGATGTCGCCAGCAAAAGGGTATAATAATGGCTATTATAGAGATAAGAATGTAGAGTATCCCTGTTTTTAAGGATCAGTACGTAGTTTTACTAT
AAAGGTACCCATATATATATATTTGCGGAACACGAGGAGGTCGGGAGTACATACTTGTAGTTACTGTTTGTTTGTGACGAGGAATGCCAGTTAGGTAAGCACCACGTGATGC
GTGACCCAAACAAGCGCCCGGTCGTTGCTTGAGGAACCAATTTCTCTGTTTATCCTCCTTCGAACACCACACTTCGACCTTCTGGACACTGCCGCTATGAGCCTTCCATAG
CCAGCCTGTTTCAGCTCGACAACACCTCTTGATGCCTTGACGACAACTCATCAACTTCTAGCTCCGTTGGTTCACGTTTTTGCTGTCCTTCTTGCGGTGTGGACCTCGTGCG
GTCGAGGGTGGCTCCGTGAAGGATCAAAACAGACGCTATTGCCCGTGGTTACCGATCGTCTCTCACCCAGCAACAAGGAAACCCAGACAGACACTGCACAGCGTAGCAG
ATCCTAAAAGAGAAAGAGATGCCTCCTGAGATGGGTAAGAAGCTGCCCGCTCCGGGGAGACGGGCACAAGGTTCATGAACCGAAGAGGATGGAACCCCGACCATGGC
CTGCGGCCGCAGCTATCAGTCCTCTCAGCTGACGCCCCTCACGAACCCTCGCACTCTCCCGAAATGCTCTTCGGTGTCCATGGCGCATCATCTCAGCAGCATCCCATGTCA
AACCATGGCTTTGGGCCCACGGACTCTGTGGCCCTGCCGCAACATCACCATCACCATCGACTCCCGCCCCATGCAGCGCTGCGCGCCCCAGGGCGTCATGGAGTGAATGT
GGAATCTCCTCCTTTGCCCTCGGGCCCGCCGAGTGTGGTGGGCCAGCCTGGAATGCCTGATCCAGCCCCCAGGCCTCGAGGACCAAAACTGAAGTTTACTCCCGAAGAGG
ACGCTCTACTGGTGGAGTTGAAGGAAAACAAGAACTTGACGTGGAAGCAAATTGCAGACTTCTTCCCGGGCCGAACGAGCGGTACCTTGCAAGTCCGATACTGCACCAA
GCTGAAGGCTAAGGATGTAGCTTGGAGTGACGAAATGGTTCGATTTGCTCCTGATGTATTTCTACGCCTGTCTCACACATGCTAATGAAGGGAATAGGTACAAAGGCTGC
AGCGGGCAATGCACGAGTACGAGAACGATCGGTGGCGCATCATTGCAGGGAAGGTTGGAAATGGCTTCACCCCAGCTGCTTGCCGCGAGAAAGCCATGCAGCTCCATGA
GTAAAAGCGTTGGGGAATTTTCATATTTATATCTACTGTCGCCAGATTCGGCCCTGCTTGGACCCTCTGATCTCCTTACTCTCCATATTGGTTCAAATGTCGGGTCACCGAT
AGGGCTGGTGGTGCAGGCTTGTTGTAGGCACGGGAGGATGATCAGCATAACTCTGAGTCACTATAGGGACGGGTTGATGTAAGGTATTAAGTGATGTATGATAATTCATT
TTAGCCCGGGGGAACATATGGCGCCGGCATTTGTTCGTTCGCAATGAACCGACACTAGCGTCCGCTCTCGCAGTTTAGCACCGGCTGATCCCGGGCTGAACGCGGCCATT
GCTCGGCCGGGGCATGTGTTCCTTATCTACGGCAGACCGCAGATGACCACTGGAGCAGATTATAGACCCTAAGCCCTAAGCCGGACACCCAATCGAGTAGGTCTGCGGAC
CAGGTCACTGCGGGCAGCCGGAGAAGCTCCGCAACCAATCAATCCCCGGCGCTGACTAAGGGCAGGCGACCACGGGCCGAAGCGGCTTCAAACTCACCTCAACCTCCAA
ACTCCCTCATCTCCAAACGTCCTTGCCTTGTCTGCCGTCATTGCAACCCACCCACCAGGACACATGAGCAGCAAGTCCCAGCTCACCTACACTGCCCGTGCCAGCAAGCAT
CCCAATGCTCTGGCGAAGAGGCTGTTCGAGATTGCCGAGGCCAAGAAaACCAATGTGACTGTCTCGGCTGACGTTACCACCACTAAGGAGCTACTAGATCTTGCTGACCG
TCTCGGTCCCTACATTGCCGTGATCAAAACCCACATCGATATCCTCTCTGATTTCAGCAACGAGACtATTGAGGGACTTAAGGCTCTCGCGCAGAAGCACAACTTTCTCATC
TTCGAGGACCGCAAGTTCATTGACATCGGCAACACGGTCCAGAAGCAATACCACGGCGGTACCCTCCGTATCTCGGAATGGGCCCACATCATCAACTGCAGCATTCTCCC
TGGTGAGGGTATCGTCGAGGCTCTCGCTCAGACGGCGTCTGCACCGGACTTCGCCTACGGCCCCGAACGCGGTCTGTTGATCTTGGCAGAGATGACCTCTAAGGGCTCCTT
GGCTACCGGCCAGTACACTACTTCCTCGGTCGATTATGCCCGGAAATACAAGAACTTCGTTATGGGATTCGTGTCGACGCGCGCGTTGGGTGAGGTGCAGTCGGAAGTCA
GCTCTCCTTCGGATGAGGAGGACTTTGTGGTtTTCACGACTGGTGTGAACATTAACCTCCA

>pAH030_IC
TGGAGGaAGGGATCCGCCAAGGATGCTCTGGATCCGCATCCGGCCGCTCTTGCGCCCCATCAATCGCCCGACTATAAATCGAACTACTTTCGGCATCTTCTAGACTTCCTA
ATACCGCCTAGTCATAGCAGATTCAAGCTGAGAACACCACAAGTAAATATCACCCATCATGCTTACCCTGACCGTCCCTGAAAACTACGGGTATGTGCCAATTCTACAATT
CCTTGCAGACAATGCCATTCTCCCCATGAAGTCTGATGCTAACTATCCTGCAGCTCTGTCATTGCCGTCGCTCTGGGTGCCATCCCCGTCCTGAGCTTCGTCCATGGCGCCG
TCGTGTCTCGTCTCCGCAAGGAAGCTGATTGCCCCTACCCTCACTGCTATGCGACCGTAGAGCAGTGCAAGACCAACGTAAGCCAACCTCACACAAACAGGATTCCTCGA
GCTAACATACATTCCGAACCGTGCAGCCCAAGGCCGAGCAGTTCAACTGCGCTCAGCGCGCTCATGCCAACTTCCTTGAGAACTCCAGCCAAACTATGCTCTTCCTCCTGG
TAGCTGGACTGAAGTACCCCCAGTTGGCGACTGGCCTCGGAAGCATCTGGGTCCTCGGTCGCTCACTGTTCCTTTACGGATATGTGTACTCCGGCAAGCCGCGGGGTCGCG
GTCGTTTGTACGGCAGCTTCTACTTGCTTGCACAGGGAGCTCTCTGGGGCTTGACGTCTTTTGGAGTTGCGAGGGAGTTGATTTCCTACTTCTAAGTTTGGACTGAATCCGT
GGTGTGATTGAGGTGATTGGCGATGTTTGGCTATACCAGCTATATGTAATAATCTCTACTGTATACTACTATTCAACGCATTTTACTATGCGTGCTGCTAGGGTCGGCAATG
ACAATGGCAATCTGACTGACGTGGTCTATTTCTCCATGTGCAGCAGGGAATACGAGCTCCAATGGACCTCGGGAGTGGCACAGTCAATGGCAAGGAAACTGGAGcgcaaaaaa
ccccgcccctgacagggcggggttttttcgcGATCGGAGCCTCTCGTATGCAGAGGAAATCTCCCCTGATCTTCCGAACTGGTCGTACCTGGCGACCTATGACTATGGCACCCCAGTTCTGG
GGACCTTCCACGGAAGTGACCTGCTGCAGGTGTTCTATGGGATCAAGCCAAACTATGCAGCTAGTTCTAGCCACACGTACTATCTGAGCTTTGTGTATACGCTGGATCCGA
ACTCCAACCGGGGGGAGTACATTGAGTGGCCGCAGTGGAAGGAATCGCGGCAGTTGATGAATTTCGGAGCGAACGACGCCAGTCTCCTTACGGATGATTTCCGCAACGG
GACATATGAGTTCATCCTGCAGAATACCGCGGCGTTCCACATCTGATGCCATTGGCGGAGGGGTCCGGACGGTCAGGAACTTAGCCTTATGAGATGAATGATGGACGTGT
CTGGCCTCGGAAAAGGATATATGGGGATCATAATAGTACTAGCCATATTAATGAAGGGCATATACCACGCGTTGGACCTGCGTTATAGCTTCCCGTTAGTTATAGTACCAT
CGTTATACCAGCCAATCAAGTCACCACGCACGACCGGGGACGGCGAATCCCCGGGAATTGAAAGAAATTGCATCCCAGGCCAGTGAGGCCAGCGATTGGCCACCTCTCC
AAGGCACAGGGCCATTCTGCAGCGCTGGTGGATTCATCGCAATTTCCCCCGGCCCGGCCCGACACCGCTATAGGCTGGTTCTCCCACACCATCGGAGATTCGTCGCCTAAT
GTCTCGTCCGTTCACAAGCTGAAGAGCTTGAAGTGGCGAGATGTCTCTGCAGGAATTCAAGCTAGATGCTAAGCGATATTGCATGGCAATATGTGTTGATGCATGTGCTTC
TTCCTTCAGCTTCCCCTCGTGCAGATGAGGTTTGGCTATAAATTGAAGTGGTTGGTCGGGGTTCCGTGAGGGGCTGAAGTGCTTCCTCCCTTTTAGACGCAACTGAGAGCC
TGAGCTTCATCCCCAGCATCATTACACCTCAGCACATGTCcTCAACCACGTCcTCATCAAGATCAGACCTTGAAAAGGTCCCCGTACCACAGGTCACCCCTAGAGACAGTG
ACTCCGATAAGGGATCCCTCTCTCCGGAGCCTTCGACCCTCGAGGCTCAGTCATCCGAGAAGCCACCGCATCATATCTTCACACGaTCTCGCAAGCTGCAAATGGTTTGCA
TCGTCTCCCTCGCTGCCATATTTTCTCCGCTTTCGTCGAACATTTACTTCCCTGCCCTGGATGATGTCTCGAAATCCCTCAACATCAGCATGTCGCTCGCAACACTCACCATC
ACGGTGTACATGATCGTCCAAGGCCTCGCTCCCAGCTTCTGGGGTTCCATGTCAGACGCCACAGGTAGACGGCCTGTCTTTATTGGAACATTCATTGTTTACCTCGTAGCC
AATATTGCTCTGGCCGAATCCAAGAACTATGGTGAGCTCATGGCCTTCCGAGCCTTGCAGGCTGCTGGTAGCGCAGCCACCATCTCAATCGGAGCTGGAGTGATTGGTGA
TATCACAAACTCGGAAGAAAGAGGTAGCTTGGTGGGTATCTTCGGTGGAGTTCGCATGCTTGGACAGGGAATCGGGCCGGTTTTCGGCGGCATTTTCACCCAGTATCTCG
GATATCGATCTATCTTTTGGTTCCTCACGATTGCTGGAGGCGTGAGTCTCCTGTCCATTCTGGTGCTTCTTCCGGAGACATTGAGgCCAATTGCTGGAAATGGAACTGTGAA
GCTCAATGGCATTCATAAGCCCTTCATCTACACGATCACCGGCCAGACGGGGGTTGTCGAGGGAGCGCAACCGGAAGCGAAAAAGACCAAAACCAGCTGGAAGTCTGTT
TTTGCTCCTTTGACATTCCTCGTCGAAAAGGACGTTTTCATCACCCTGTTCTTTGGAAGTATCGTGTACACAGTGTGGAGCATGGTGACATCCAGTACCACCGACCTCTTCA
GCGAAGTGTACGGCCTGTCATCCCTGGACATTGGACTCACTTTCCTAGGCAATGGCTTTGGATGTATGTCTGGCTCTTATCTGGTCGGCTACCTTATGGATTACAACCACCG
TCTTACCGAACGCGAATATTGCGAGAAACACGGTTATCCGGCAGGCACACGTGTCAATCTGAAATCACACCCCGACTTCCCCATTGAGGTCGCCCGGATGCGCAATACCT
GGTGGGTGATTGCGATCTTCATCGTGACAGTTGCTTTGTACGGCGTGTCTTTGCGGACACATCTGGCGGTGCCTATCATTCTGCAGTACTTCATTGCGTTCTGCTCAACAGG
ACTCTTCACCATCAACAGCGCCCTGGTCATCGATCTTTACCCAGGTGCTAGCGCCAGTGCGACAGCAGTGAACAATCTGATGCGGTGCCTGCTTGGAGCTGGCGGTGTGG
CTATCGTGCAACCTATCCTGGACGCCTTGAAGCCGGATTATACTTTCCTCTTGCTTGCCGGCATCACCCTTGTGATGACTCCGTTGCTGTACGTCGAAGATCGATGGGGTCC
TGGCTGGCGACATGCCCGCGAAAGGAGACTCAAGGCCAAAGCCAACGGCAACTAGGCTTGGAGAGAAAGGACTTGAAAAAAAAAAGGTGAAGTGGGACTGGTGAAGTA
AATGTTTGATTCTTCCCCACACTTTCTTGATATGGGTTTTATTTTAGCGGCATTTGGCGATACCACTGTTTTGCAAGCGATACCAGTTAGATTTATAGAAGAATTGATCAGT
TTCATCGGCTATCTCGCTATTACTTCCTCGTAGCTTTTTAGCTTCATATATGGGTGAGTGGGAAAGGTTTGACATGGTGGTTGAGATAGTATCATTTGGATGGAGATGGAA
GTATAAAGCAAGAAGTTCTGTTGTTTGGTGTTTAGACTAGACTAGACGTGGCCGGTGAAGTCACGAGCCTCAGCTTAGATAATCTAATGCGGCTGATGTGACTGACCGAT
GGCTTTGTTATATGAgGACAGGTCGGTCAATTAGAAGATGTCGCCAGCAAAAGGGTATAATAATGGCTATTATAGAGATAAGAATGTAGAGTATCCCTGTTTTTAAGGATC
AGTACGTAGTTTTACTATAAAGGTACCCATATATATATATTTGCGGAACACGAGGAGGTCGGGAGTACATACTTGTAGTTACTGTTTGTTTGTGACGAGGAATGCCAGTTAGG
TAAGCACCACGTGATGCGTGACCCAAACAAGCGCCCGGTCGTTGCTTGAGGAACCAATTTCTCTGTTTATCCTCCTTCGAACACCACACTTCGACCTTCTGGACACTGCCG
CTATGAGCCTTCCATAGCCAGCCTGTTTCAGCTCGACAACACCTCTTGATGCCTTGACGACAACTCATCAACTTCTAGCTCCGTTGGTTCACGTTTTTGCTGTCCTTCTTGCG
GTGTGGACCTCGTGCGGTCGAGGGTGGCTCCGTGAAGGATCAAAACAGACGCTATTGCCCGTGGTTACCGATCGTCTCTCACCCAGCAACAAGGAAACCCAGACAGACA
CTGCACAGCGTAGCAGATCCTAAAAGAGAAAGAGATGCCTCCTGAGATGGGTAAGAAGCTGCCCGCTCCGGGGAGACGGGCACAAGGTTCATGAACCGAAGAGGATGG
GAACCCCGACCATGGCCTGCGGCCGCAGCTATCAGTCCTCTCAGCTGACGCCCCTCACGAACCCTCGCACTCTCCCGAAATGCTCTTCGGTGTCCATGGCGCATCATCTCA
GCAGCATCCCATGTCAAACCATGGCTTTGGGCCCACGGACTCTGTGGCCCTGCCGCAACATCACCATCACCATCGACTCCCGCCCCATGCAGCGCTGCGCGCCCCAGGGC
GTCATGGAGTGAATGTGGAATCTCCTCCTTTGCCCTCGGGCCCGCCGAGTGTGGTGGGCCAGCCTGGAATGCCTGATCCAGCCCCCAGGCCTCGAGGACCAAAACTGAAG
TTTACTCCCGAAGAGGACGCTCTACTGGTGGAGTTGAAGGAAAACAAGAACTTGACGTGGAAGCAAATTGCAGACTTCTTCCCGGGCCGAACGAGCGGTACCTTGCAAGT
CCGATACTGCACCAAGCTGAAGGCTAAGGATGTAGCTTGGAGTGACGAAATGGTTCGATTTGCTCCTGATGTATTTCTACGCCTGTCTCACACATGCTAATGAAGGGAAT
AGGTACAAAGGCTGCAGCGGGCAATGCACGAGTACGAGAACGATCGGTGGCGCATCATTGCAGGGAAGGTTGGAAATGGCTTCACCCCAGCTGCTTGCCGCGAGAAAGC
CATGCAGCTCCATGAGTAAAAGCGTTGGGGAATTTTCATATTTATATCTACTGTCGCCAGATTCGGCCCTGCTTGGACCCTCTGATCTCCTTACTCTCCATATTGGTTCAAA
TGTCGGGTCACCGATAGGGCTGGTGGTGCAGGCTTGTTGTAGGCACGGGAGGATGATCAGCATAACTCTGAGTCACTATAGGGACGGGTTGATGTAAGGTATTAAGTGAT
GTATGATAATTCATTTTAGCCCGGGGGAACATATGGCGCCGGCATTTGTTCGTTCGCAATGAACCGACACTAGCGTCCGCTCTCGCAGTTTAGCACCGGCTGATCCCGGGC
TGAACGCGGCCATTGCTCGGCCGGGGCATGTGTTCCTTATCTACGGCAGACCGCAGATGACCACTGGAGCAGATTATAGACCCTAAGCCCTAAGCCGGACACCCAATCGA
GTAGGTCTGCGGACCAGGTCACTGCGGGCAGCCGGAGAAGCTCCGCAACCAATCAATCCCCGGCGCTGACTAAGGGCAGGCGACCACGGGCCGAAGCGGCTTCAAACTC
ACCTCAACCTCCAAACTCCCTCATCTCCAAACGTCCTTGCCTTGTCTGCCGTCATTGCAACCCACCCACCAGGACACATGAGCAGCAAGTCCCAGCTCACCTACACTGCCC
GTGCCAGCAAGCATCCCAATGCTCTGGCGAAGAGGCTGTTCGAGATTGCCGAGGCCAAGAAaACCAATGTGACTGTCTCGGCTGACGTTACCACCACTAAGGAGCTACTA
GATCTTGCTGACCGTCTCGGTCCCTACATTGCCGTGATCAAAACCCACATCGATATCCTCTCTGATTTCAGCAACGAGACtATTGAGGGACTTAAGGCTCTCGCGCAGAAG
CACAACTTTCTCATCTTCGAGGACCGCAAGTTCATTGACATCGGCAACACGGTCCAGAAGCAATACCACGGCGGTACCCTCCGTATCTCGGAATGGGCCCACATCATCAA
CTGCAGCATTCTCCCTGGTGAGGGTATCGTCGAGGCTCTCGCTCAGACGGCGTCTGCACCGGACTTCGCCTACGGCCCCGAACGCGGTCTGTTGATCTTGGCAGAGATGAC
CTCTAAGGGCTCCTTGGCTACCGGCCAGTACACTACTTCCTCGGTCGATTATGCCCGGAAATACAAGAACTTCGTTATGGGATTCGTGTCGACGCGCGCGTTGGGTGAGGT
GCAGTCGGAAGTCAGCTCTCCTTCGGATGAGGAGGACTTTGTGGTtTTCACGACTGGTGTGAACATTAACCTCCA

>pAH036_IC
TGGAGGaAGGGATCCGCCAAGGATGCTCTGGATCCGCATCCGGCCGCTCTTGCGCCCCATCAATCGCCCGACTATAAATCGAACTACTTTCGGCATCTTCTAGACTTCCTA
ATACCGCCTAGTCATAGCAGATTCAAGCTGAGAACACCACAAGTAAATATCACCCATCATGCTTACCCTGACCGTCCCTGAAAACTACGGGTATGTGCCAATTCTACAATT
CCTTGCAGACAATGCCATTCTCCCCATGAAGTCTGATGCTAACTATCCTGCAGCTCTGTCATTGCCGTCGCTCTGGGTGCCATCCCCGTCCTGAGCTTCGTCCATGGCGCCG
TCGTGTCTCGTCTCCGCAAGGAAGCTGATTGCCCCTACCCTCACTGCTATGCGACCGTAGAGCAGTGCAAGACCAACGTAAGCCAACCTCACACAAACAGGATTCCTCGA
GCTAACATACATTCCGAACCGTGCAGCCCAAGGCCGAGCAGTTCAACTGCGCTCAGCGCGCTCATGCCAACTTCCTTGAGAACTCCAGCCAAACTATGCTCTTCCTCCTGG
TAGCTGGACTGAAGTACCCCCAGTTGGCGACTGGCCTCGGAAGCATCTGGGTCCTCGGTCGCTCACTGTTCCTTTACGGATATGTGTACTCCGGCAAGCCGCGGGGTCGCG
GTCGTTTGTACGGCAGCTTCTACTTGCTTGCACAGGGAGCTCTCTGGGGCTTGACGTCTTTTGGAGTTGCGAGGGAGTTGATTTCCTACTTCTAAGTTTGGACTGAATCCGT
GGTGTGATTGAGGTGATTGGCGATGTTTGGCTATACCAGCTATATGTAATAATCTCTACTGTATACTACTATTCAACGCATTTTACTATGCGTGCTGCTAGGGTCGGCAATG
ACAATGGCAATCTGACTGACGTGGTCTATTTCTCCATGTGCAGCAGGGAATACGAGCTCCAATGGACCTCGGGAGTGGCACAGTCAATGGCAAGGAAACTGGAGcgcaaaaaa

ccccgcccctgacagggcggggtttttcgcGATCGGAGTTTAAGCTTTCGCTTCAGCCATAGCTTATAAACAGTAAGATTATGAGAGGGGGAAAAGCTTCAACCAAGACTATTTTCACCCG
TCTTTAGGTTGTTCATAATGGGCTACGGCTTCATGGCTCTCATGCCTCCATCGTATAATCGTCCCTTGTAGACAGTAGCCAGCTACTTACTAGTTAGCACAGTGGACACAA
GCCAGCGAATCGGGGTGAAGCCAATTAGCATTGGATATCCACGAAGTATGATCGTCACCCTCCATGACTTCACACGGTTGCTGCATCGGTTCACCGGGTTGAACCTAGCC
GGGCAGGATCGGAGCAAGTCGCTGGATCCCTCACGGATCACAGAGCTCCAGCTCCACGAAGAAAAGCAGGATGCGATCCCCGCACATCGGAGAATTGCATACCGTTGAA
GCAATCCAAGCAATTGGCAGGTGTCATCTCCATTACGTACCGTGACCTTCATACCCTCCGTATTTATACCTGCCCCTCCCTCCCCCGCTTCTCCTGCTTTCTTCCTCTCTCTT
TCTCTCTTCCATCAACTCAACTTCCATCTACTTCACTACTTCCTGTCAAGTCaTCCATCGACTTTCATCACAAGTCATCCTCTCCCATGTTTGTATTCCCTGTATAGACCGTAC
TGACCTCTTCCCTAGAAACCCTCGTGCAATGTAATTCCACCTGCGTCGGCCCCTCCCCGGGGaCTCGTCTCAATACTTCATTACACACGATGGAAGTCATGCAATGCCTTTG
GCTGGACTCTCTCAATGATCAGGTATCTCAGGTAAATCTTGGGCGTGGACCGGTGTTCGTTCCTATCCGTTGGTTGTGCAGCATTGCTAGCATTGCTGCCTGCCATCGGCTC
TGGGTTCGTTCTGAGATTATACGGTTAAACTTGATCTGGATAATACCAGCGAAAGGATCATGCCTTCCCTCGTTTCCCCCCACTTGATGGAATGGCTAACAATTCCCAGTC
CCACCTACCACACCCTTAAAGCAACCATGTCcTCAACCACGTCcTCATCAAGATCAGACCTTGAAAAGGTCCCCGTACCACAGGTCACCCCTAGAGACAGTGACTCCGATA
AGGGATCCCTCTCTCCGGAGCCTTCGACCCTCGAGGCTCAGTCATCCGAGAAGCCACCGCATCATATCTTCACACGaTCTCGCAAGCTGCAAATGGTTTGCATCGTCTCCCT
CGCTGCCATATTTTCTCCGCTTTCGTCGAACATTTACTTCCCTGCCCTGGATGATGTCTCGAAATCCCTCAACATCAGCATGTCGCTCGCAACACTCACCATCACGGTGTAC
ATGATCGTCCAAGGCCTCGCTCCCAGCTTCTGGGGTTCCATGTCAGACGCCACAGGTAGACGGCCTGTCTTTATTGGAACATTCATTGTTTACCTCGTAGCCAATATTGCTC
TGGCCGAATCCAAGAACTATGGTGAGCTCATGGCCTTCCGAGCCTTGCAGGCTGCTGGTAGCGCAGCCACCATCTCAATCGGAGCTGGAGTGATTGGTGATATCACAAAC
TCGGAAGAAAGAGGTAGCTTGGTGGGTATCTTCGGTGGAGTTCGCATGCTTGGACAGGGAATCGGGCCGGTTTTCGGCGGCATTTTCACCCAGTATCTCGGATATCGATCT
ATCTTTTGGTTCCTCACGATTGCTGGAGGCGTGAGTCTCCTGTCCATTCTGGTGCTTCTTCCGGAGACATTGAGgCCAATTGCTGGAAATGGAACTGTGAAGCTCAATGGCA
TTCATAAGCCCTTCATCTACACGATCACCGGCCAGACGGGGGTTGTCGAGGGAGCGCAACCGGAAGCGAAAAAGACCAAAACCAGCTGGAAGTCTGTTTTTGCTCCTTTG
ACATTCCTCGTCGAAAAGGACGTTTTCATCACCCTGTTCTTTGGAAGTATCGTGTACACAGTGTGGAGCATGGTGACATCCAGTACCACCGACCTCTTCAGCGAAGTGTAC
GGCCTGTCATCCCTGGACATTGGACTCACTTTCCTAGGCAATGGCTTTGGATGTATGTCTGGCTCTTATCTGGTCGGCTACCTTATGGATTACAACCACCGTCTTACCGAAC
GCGAATATTGCGAGAAACACGGTTATCCGGCAGGCACACGTGTCAATCTGAAATCACACCCCGACTTCCCCATTGAGGTCGCCCGGATGCGCAATACCTGGTGGGTGATT
GCGATCTTCATCGTGACAGTTGCTTTGTACGGCGTGTCTTTGCGGACACATCTGGCGGTGCCTATCATTCTGCAGTACTTCATTGCGTTCTGCTCAACAGGACTCTTCACCA
TCAACAGCGCCCTGGTCATCGATCTTTACCCAGGTGCTAGCGCCAGTGCGACAGCAGTGAACAATCTGATGCGGTGCCTGCTTGGAGCTGGCGGTGTGGCTATCGTGCAA
CCTATCCTGGACGCCTTGAAGCCGGATTATACTTTCCTCTTGCTTGCCGGCATCACCCTTGTGATGACTCCGTTGCTGTACGTCGAAGATCGATGGGGTCCTGGCTGGCGAC
ATGCCCGCGAAAGGAGACTCAAGGCCAAAGCCAACGGCAACTAGGCTTGGAGAGAAAGGACTTGAAAAAAAAAAGGTGAAGTGGGACTGGTGAAGTAAATGTTTGATT
CTTCCCCACACTTTCTTGATATGGGTTTTATTTTAGCGGCATTTGGCGATACCACTGTTTTGCAAGCGATACCAGTTAGATTTATAGAAGAATTGATCAGTTTCATCGGCTA
TCTCGCTATTACTTCCTCGTAGCTTTTTAGCTTCATATATGGGTGAGTGGGAAAGGTTTGACATGGTGGTTGAGATAGTATCATTTGGATGGAGATGGAAGTATAAAGCAA
GAAGTTCTGTTGTTTGGTGTTTAGACTAGACTAGACGTGGCCGGTGAAGTCACGAGCCTCAGCTTAGATAATCTAATGCGGCTGATGTGACTGACCGATGGCTTTGTTATA
TGAgGACAGGTCGGTCAATTAGAAGATGTCGCCAGCAAAAGGGTATAATAATGGCTATTATAGAGATAAGAATGTAGAGTATCCCTGTTTTTAAGGATCAGTACGTAGTT
TTACTATAAAGGTACCCATATATATATTTGCGGAACACGAGGAGGTCGGGAGTACATACTTGTAGTTACTGTTTGTTTGTGACGAGGAATGCCAGTTAGGTAAGCACCAC
GTGATGCGTGACCCAAACAAGCGCCCGGTCGTTGCTTGAGGAACCAATTTCTCTGTTTATCCTCCTTCGAACACCACACTTCGACCTTCTGGACACTGCCGCTATGAGCCT
TCCATAGCCAGCCTGTTTCAGCTCGACAACACCTCTTGATGCCTTGACGACAACTCATCAACTTCTAGCTCCGTTGGTTCACGTTTTTGCTGTCCTTCTTGCGGTGTGGACC
TCGTGCGGTCGAGGGTGGCTCCGTGAAGGATCAAAACAGACGCTATTGCCCGTGGTTACCGATCGTCTCTCACCCAGCAACAAGGAAACCCAGACAGACACTGCACAGC
GTAGCAGATCCTAAAAGAGAAAGAGATGCCTCCTGAGATGGGTAAGAAGCTGCCCGCTCCGGGGAGACGGGCACAAGGTTCATGAACCGAAGAGGATGGGAACCCCGA
CCATGGCCTGCGGCCGCAGCTATCAGTCCTCTCAGCTGACGCCCCTCACGAACCCTCGCACTCTCCCGAAATGCTCTTCGGTGTCCATGGCGCATCATCTCAGCAGCATCC
CATGTCAAACCATGGCTTTGGGCCCACGGACTCTGTGGCCCTGCCGCAACATCACCATCACCATCGACTCCCGCCCCATGCAGCGCTGCGCGCCCCAGGGCGTCATGGAG
TGAATGTGGAATCTCCTCCTTTGCCCTCGGGCCCGCCGAGTGTGGTGGGCCAGCCTGGAATGCCTGATCCAGCCCCCAGGCCTCGAGGACCAAAACTGAAGTTTACTCCC
GAAGAGGACGCTCTACTGGTGGAGTTGAAGGAAAACAAGAACTTGACGTGGAAGCAAATTGCAGACTTCTTCCCGGGCCGAACGAGCGGTACCTTGCAAGTCCGATACT
GCACCAAGCTGAAGGCTAAGGATGTAGCTTGGAGTGACGAAATGGTTCGATTTGCTCCTGATGTATTTCTACGCCTGTCTCACACATGCTAATGAAGGGAATAGGTACAA
AGGCTGCAGCGGGCAATGCACGAGTACGAGAACGATCGGTGGCGCATCATTGCAGGGAAGGTTGGAAATGGCTTCACCCCAGCTGCTTGCCGCGAGAAAGCCATGCAGC
TCCATGAGTAAAAGCGTTGGGGAATTTTCATATTTATATCTACTGTCGCCAGATTCGGCCCTGCTTGGACCCTCTGATCTCCTTACTCTCCATATTGGTTCAAATGTCGGGT
CACCGATAGGGCTGGTGGTGCAGGCTTGTTGTAGGCACGGGAGGATGATCAGCATAACTCTGAGTCACTATAGGGACGGGTTGATGTAAGGTATTAAGTGATGTATGATA
ATTCATTTTAGCCCGGGGGAACATATGGCGCCGGCATTTGTTCGTTCGCAATGAACCGACACTAGCGTCCGCTCTCGCAGTTTAGCACCGGCTGATCCCGGGCTGAACGCG
GCCATTGCTCGGCCGGGGCATGTGTTCCTTATCTACGGCAGACCGCAGATGACCACTGGAGCAGATTATAGACCCTAAGCCCTAAGCCGGACACCCAATCGAGTAGGTCT
GCGGACCAGGTCACTGCGGGCAGCCGGAGAAGCTCCGCAACCAATCAATCCCCGGCGCTGACTAAGGGCAGGCGACCACGGGCCGAAGCGGCTTCAAACTCACCTCAAC
CTCCAAACTCCCTCATCTCCAAACGTCCTTGCCTTGTCTGCCGTCATTGCAACCCACCCACCAGGACACATGAGCAGCAAGTCCCAGCTCACCTACACTGCCCGTGCCAGC
AAGCATCCCAATGCTCTGGCGAAGAGGCTGTTCGAGATTGCCGAGGCCAAGAAaACCAATGTGACTGTCTCGGCTGACGTTACCACCACTAAGGAGCTACTAGATCTTGC
TGACCGTCTCGGTCCCTACATTGCCGTGATCAAAACCCACATCGATATCCTCTCTGATTTCAGCAACGAGACtATTGAGGGACTTAAGGCTCTCGCGCAGAAGCACAACTT
TCTCATCTTCGAGGACCGCAAGTTCATTGACATCGGCAACACGGTCCAGAAGCAATACCACGGCGGTACCCTCCGTATCTCGGAATGGGCCCACATCATCAACTGCAGCA
TTCTCCCTGGTGAGGGTATCGTCGAGGCTCTCGCTCAGACGGCGTCTGCACCGGACTTCGCCTACGGCCCCGAACGCGGTCTGTTGATCTTGGCAGAGATGACCTCTAAGG
GCTCCTTGGCTACCGGCCAGTACACTACTTCCTCGGTCGATTATGCCCGGAAATACAAGAACTTCGTTATGGGATTCGTGTCGACGCGCGCGTTGGGTGAGGTGCAGTCGG
AAGTCAGCTCTCCTTCGGATGAGGAGGACTTTGTGGTtTTCACGACTGGTGTGAACATTAACCTCCA

>pAH034_IC
TGGAGGaAGGGATCCGCCAAGGATGCTCTGGATCCGCATCCGGCCGCTCTTGCGCCCCATCAATCGCCCGACTATAAATCGAACTACTTTCGGCATCTTCTAGACTTCCTA
ATACCGCCTAGTCATAGCAGATTCAAGCTGAGAACACCACAAGTAAATATCACCCATCATGCTTACCCTGACCGTCCCTGAAAACTACGGGTATGTGCCAATTCTACAATT
CCTTGCAGACAATGCCATTCTCCCCATGAAGTCTGATGCTAACTATCCTGCAGCTCTGTCATTGCCGTCGCTCTGGGTGCCATCCCCGTCCTGAGCTTCGTCCATGGCGCCG
TCGTGTCTCGTCTCCGCAAGGAAGCTGATTGCCCCTACCCTCACTGCTATGCGACCGTAGAGCAGTGCAAGACCAACGTAAGCCAACCTCACACAAACAGGATTCCTCGA
GCTAACATACATTCCGAACCGTGCAGCCCAAGGCCGAGCAGTTCAACTGCGCTCAGCGCGCTCATGCCAACTTCCTTGAGAACTCCAGCCAAACTATGCTCTTCCTCCTGG
TAGCTGGACTGAAGTACCCCCAGTTGGCGACTGGCCTCGGAAGCATCTGGGTCCTCGGTCGCTCACTGTTCCTTTACGGATATGTGTACTCCGGCAAGCCGCGGGGTCGCG
GTCGTTTGTACGGCAGCTTCTACTTGCTTGCACAGGGAGCTCTCTGGGGCTTGACGTCTTTTGGAGTTGCGAGGGAGTTGATTTCCTACTTCTAAGTTTGGACTGAATCCGT
GGTGTGATTGAGGTGATTGGCGATGTTTGGCTATACCAGCTATATGTAATAATCTCTACTGTATACTACTATTCAACGCATTTTACTATGCGTGCTGCTAGGGTCGGCAATG
ACAATGGCAATCTGACTGACGTGGTCTATTTCTCCATGTGCAGCAGGGAATACGAGCTCCAATGGACCTCGGGAGTGGCACAGTCAATGGCAAGGAAACTGGAGcgcaaaaaa
ccccgcccctgacagggcggggtttttcgcGATCGGAGCTCTTGTAACAGGGCTAGAAGCACTGGGATTCATGGGGAGCGGAGGGGGTGCCCGTTCGGGGTCCGAACTGTCCCACCTTCGT
TTCCACAGTCCACGGTCAGCCTGACGCGGTTCATAGTTGTAGCGGCAGAGGGGGGCTCAACAATAGAGTTTACACATACATGGGCACCGATTGATTTCTGACGACCAGTT
CCCTGTCGCGGGACCCATCTCGTGTGTGCGTAGGCATCATGTAACTTAATCGTCGTTAAAGCAAGAAAACGGGGTATGTCTGTCGATGTTGGATATAAAGCAAGTCGATC
GAGGGATATCAGGAATGTGCGAGAGGAATGTTCGTCAAAATCAAAGCAATGGGGGTCGTAAAGGGCATATCAAATCGTCAAAACAAAAACCAAAAGGACGCAGGAGGG
GGGGAATTTGGAAGTCAATAGGCGACAAAAAAGCAGGGACAAGAAAAAGAATATAGACTGAAGATGATGATACAAATGCGCTTGCTATGAGAGATGAGGGGAAACAAG
GACGACGAAACACAATGCGACGAGACGGTTGCAAGAcACCAAGGAACACGAGCAGGCTCCAGAGGTTGAACAGTCAATATAAGAGACAATCAAACATCCAAGTGGAGT
TCCTGGCTGAAAGGAACGGTAGCTCATGGGGCATCAACGGAAGCCCGATCCAATGTGCGGCGTACACATCGAAGCTCCCGTCATAGCAACCGACGGTTCTTTGGTTGTCC
GGTAAACAAAGAGCCAAGGCCCGATCGAGGCGGTGAGTTTGTCCGACCTCCCCGACATCTCGGGCACTAGTCCGTTTGGAGAAACCTTCCCAGAACGGCTAGTAGCCTTT
TGTCGGTAAGATGACCAATGAGATTTTGGCGCACAAAGCCCTGAGCCCTCTTCGCTCAGAAAAATAGAACGTCCAACGCGGAGAACCTCACTTACTCGACCGAAGCACAT
CGACAACCACCCCATCGACCTGCCGCTCACCGGGACTTTCACACATGTCcTCAACCACGTCcTCATCAAGATCAGACCTTGAAAAGGTCCCCGTACCACAGGTCACCCCTA
GAGACAGTGACTCCGATAAGGGATCCCTCTCTCCGGAGCCTTCGACCCTCGAGGCTCAGTCATCCGAGAAGCCACCGCATCATATCTTCACACGaTCTCGCAAGCTGCAAA
TGGTTTGCATCGTCTCCCTCGCTGCCATATTTTCTCCGCTTTCGTCGAACATTTACTTCCCTGCCCTGGATGATGTCTCGAAATCCCTCAACATCAGCATGTCGCTCGCAACA
CTCACCATCACGGTGTACATGATCGTCCAAGGCCTCGCTCCCAGCTTCTGGGGTTCCATGTCAGACGCCACAGGTAGACGGCCTGTCTTTATTGGAACATTCATTGTTTAC
CTCGTAGCCAATATTGCTCTGGCCGAATCCAAGAACTATGGTGAGCTCATGGCCTTCCGAGCCTTGCAGGCTGCTGGTAGCGCAGCCACCATCTCAATCGGAGCTGGAGT
GATTGGTGATATCACAAACTCGGAAGAAAGAGGTAGCTTGGTGGGTATCTTCGGTGGAGTTCGCATGCTTGGACAGGGAATCGGGCCGGTTTTCGGCGGCATTTTCACCC
AGTATCTCGGATATCGATCTATCTTTTGGTTCCTCACGATTGCTGGAGGCGTGAGTCTCCTGTCCATTCTGGTGCTTCTTCCGGAGACATTGAGgCCAATTGCTGGAAATGG
AACTGTGAAGCTCAATGGCATTCATAAGCCCTTCATCTACACGATCACCGGCCAGACGGGGGTTGTCGAGGGAGCGCAACCGGAAGCGAAAAAGACCAAAACCAGCTGG
AAGTCTGTTTTTGCTCCTTTGACATTCCTCGTCGAAAAGGACGTTTTCATCACCCTGTTCTTTGGAAGTATCGTGTACACAGTGTGGAGCATGGTGACATCCAGTACCACCG
ACCTCTTCAGCGAAGTGTACGGCCTGTCATCCCTGGACATTGGACTCACTTTCCTAGGCAATGGCTTTGGATGTATGTCTGGCTCTTATCTGGTCGGCTACCTTATGGATTA
CAACCACCGTCTTACCGAACGCGAATATTGCGAGAAACACGGTTATCCGGCAGGCACACGTGTCAATCTGAAATCACACCCCGACTTCCCCATTGAGGTCGCCCGGATGC
GCAATACCTGGTGGGTGATTGCGATCTTCATCGTGACAGTTGCTTTGTACGGCGTGTCTTTGCGGACACATCTGGCGGTGCCTATCATTCTGCAGTACTTCATTGCGTTCTG
CTCAACAGGACTCTTCACCATCAACAGCGCCCTGGTCATCGATCTTTACCCAGGTGCTAGCGCCAGTGCGACAGCAGTGAACAATCTGATGCGGTGCCTGCTTGGAGCTG
GCGGTGTGGCTATCGTGCAACCTATCCTGGACGCCTTGAAGCCGGATTATACTTTCCTCTTGCTTGCCGGCATCACCCTTGTGATGACTCCGTTGCTGTACGTCGAAGATCG
ATGGGGTCCTGGCTGGCGACATGCCCGCGAAAGGAGACTCAAGGCCAAAGCCAACGGCAACTAGGCTTGGAGAGAAAGGACTTGAAAAAAAAAAGGTGAAGTGGGACT
GGTGAAGTAAATGTTTGATTCTTCCCCACACTTTCTTGATATGGGTTTTATTTTAGCGGCATTTGGCGATACCACTGTTTTGCAAGCGATACCAGTTAGATTTATAGAAGAA
TTGATCAGTTTCATCGGCTATCTCGCTATTACTTCCTCGTAGCTTTTTAGCTTCATATATGGGTGAGTGGGAAAGGTTTGACATGGTGGTTGAGATAGTATCATTTGGATGG
AGATGGAAGTATAAAGCAAGAAGTTCTGTTGTTTGGTGTTTAGACTAGACTAGACGTGGCCGGTGAAGTCACGAGCCTCAGCTTAGATAATCTAATGCGGCTGATGTGAC
TGACCGATGGCTTTGTTATATGAgGACAGGTCGGTCAATTAGAAGATGTCGCCAGCAAAAGGGTATAATAATGGCTATTATAGAGATAAGAATGTAGAGTATCCCTGTTTT
TAAGGATCAGTACGTAGTTTTACTATAAAGGTACCCATATATATATTTGCGGAACACGAGGAGGTCGGGAGTACATACTTGTAGTTACTGTTTGTTTGTGACGAGGAATGC
CAGTTAGGTAAGCACCACGTGATGCGTGACCCAAACAAGCGCCCGGTCGTTGCTTGAGGAACCAATTTCTCTGTTTATCCTCCTTCGAACACCACACTTCGACCTTCTGGA
CACTGCCGCTATGAGCCTTCCATAGCCAGCCTGTTTCAGCTCGACAACACCTCTTGATGCCTTGACGACAACTCATCAACTTCTAGCTCCGTTGGTTCACGTTTTTGCTGTC
CTTCTTGCGGTGTGGACCTCGTGCGGTCGAGGGTGGCTCCGTGAAGGATCAAAACAGACGCTATTGCCCGTGGTTACCGATCGTCTCTCACCCAGCAACAAGGAAACCCA
GACAGACACTGCACAGCGTAGCAGATCCTAAAAGAGAAAGAGATGCCTCCTGAGATGGGTAAGAAGCTGCCCGCTCCGGGGAGACGGGCACAAGGTTCATGAACCGAA
GAGGATGGGAACCCCGACCATGGCCTGCGGCCGCAGCTATCAGTCCTCTCAGCTGACGCCCCTCACGAACCCTCGCACTCTCCCGAAATGCTCTTCGGTGTCCATGGCGC
ATCATCTCAGCAGCATCCCATGTCAAACCATGGCTTTGGGCCCACGGACTCTGTGGCCCTGCCGCAACATCACCATCACCATCGACTCCCGCCCCATGCAGCGCTGCGCGC
CCCAGGGCGTCATGGAGTGAATGTGGAATCTCCTCCTTTGCCCTCGGGCCCGCCGAGTGTGGTGGGCCAGCCTGGAATGCCTGATCCAGCCCCCAGGCCTCGAGGACCAA
AACTGAAGTTTACTCCCGAAGAGGACGCTCTACTGGTGGAGTTGAAGGAAAACAAGAACTTGACGTGGAAGCAAATTGCAGACTTCTTCCCGGGCCGAACGAGCGGTAC
CTTGCAAGTCCGATACTGCACCAAGCTGAAGGCTAAGGATGTAGCTTGGAGTGACGAAATGGTTCGATTTGCTCCTGATGTATTTCTACGCCTGTCTCACACATGCTAATG
AAGGGAATAGGTACAAAGGCTGCAGCGGGCAATGCACGAGTACGAGAACGATCGGTGGCGCATCATTGCAGGGAAGGTTGGAAATGGCTTCACCCCAGCTGCTTGCCGC
GAGAAAGCCATGCAGCTCCATGAGTAAAAGCGTTGGGGAATTTTCATATTTATATCTACTGTCGCCAGATTCGGCCCTGCTTGGACCCTCTGATCTCCTTACTCTCCATATT
GGTTCAAATGTCGGGTCACCGATAGGGCTGGTGGTGCAGGCTTGTTGTAGGCACGGGAGGATGATCAGCATAACTCTGAGTCACTATAGGGACGGGTTGATGTAAGGTAT

TAAGTGATGTATGATAATTCATTTTAGCCCGGGGGAACATATGGCGCCGGCATTTGTTCGTTCGCAATGAACCGACACTAGCGTCCGCTCTCGCAGTTTAGCACCGGCTGA
TCCCGGGCTGAACGCGGCCATTGCTCGGCCGGGGCATGTGTTCCTTATCTACGGCAGACCGCAGATGACCACTGGAGCAGATTATAGACCCTAAGCCCTAAGCCGGACAC
CCAATCGAGTAGGTCTGCGGACCAGGTCACTGCGGGCAGCCGGAGAAGCTCCGCAACCAATCAATCCCCGGCGCTGACTAAGGGCAGGCGACCACGGGCCGAAGCGGCT
TCAAACTCACCTCAACCTCCAAACTCCCTCATCTCCAAACGTCCTTGCCTTGTCTGCCGTCATTGCAACCCACCCACCAGGACACATGAGCAGCAAGTCCCAGCTCACCTA
CACTGCCCGTGCCAGCAAGCATCCCAATGCTCTGGCGAAGAGGCTGTTCGAGATTGCCGAGGCCAAGAAaACCAATGTGACTGTCTCGGCTGACGTTACCACCACTAAGG
AGCTACTAGATCTTGCTGACCGTCTCGGTCCCTACATTGCCGTGATCAAAACCCACATCGATATCCTCTCTGATTTCAGCAACGAGACtATTGAGGGACTTAAGGCTCTCGC
GCAGAAGCACAACTTTCTCATCTTCGAGGACCGCAAGTTCATTGACATCGGCAACACGGTCCAGAAGCAATACCACGGCGGTACCCTCCGTATCTCGGAATGGGCCCACA
TCATCAACTGCAGCATTCTCCCTGGTGAGGGTATCGTCGAGGCTCTCGCTCAGACGGCGTCTGCACCGGACTTCGCCTACGGCCCCGAACGCGGTCTGTTGATCTTGGCAG
AGATGACCTCTAAGGGCTCCTTGGCTACCGGCCAGTACACTACTTCCTCGGTCGATTATGCCCGGAAATACAAGAACTTCGTTATGGGATTCGTGTCGACGCGCGCGTTGG
GTGAGGTGCAGTCGGAAGTCAGCTCTCCTTCGGATGAGGAGGACTTTGTGGTtTTCACGACTGGTGTGAACATTAACCTCCA

>pAH026_IC
TGGAGGaAGGGATCCGCCAAGGATGCTCTGGATCCGCATCCGGCCGCTCTTGCGCCCCATCAATCGCCCGACTATAAATCGAACTACTTTCGGCATCTTCTAGACTTCCTA
ATACCGCCTAGTCATAGCAGATTCAAGCTGAGAACACCACAAGTAAATATCACCCATCATGCTTACCCTGACCGTCCCTGAAAACTACGGGTATGTGCCAATTCTACAATT
CCTTGCAGACAATGCCATTCTCCCCATGAAGTCTGATGCTAACTATCCTGCAGCTCTGTCATTGCCGTCGCTCTGGGTGCCATCCCCGTCCTGAGCTTCGTCCATGGCGCCG
TCGTGTCTCGTCTCCGCAAGGAAGCTGATTGCCCCTACCCTCACTGCTATGCGACCGTAGAGCAGTGCAAGACCAACGTAAGCCAACCTCACACAAACAGGATTCCTCGA
GCTAACATACATTCCGAACCGTGCAGCCCAAGGCCGAGCAGTTCAACTGCGCTCAGCGCGCTCATGCCAACTTCCTTGAGAACTCCAGCCAAACTATGCTCTTCCTCCTGG
TAGCTGGACTGAAGTACCCCCAGTTGGCGACTGGCCTCGGAAGCATCTGGGTCCTCGGTCGCTCACTGTTCCTTTACGGATATGTGTACTCCGGCAAGCCGCGGGGTCGCG
GTCGTTTGTACGGCAGCTTCTACTTGCTTGCACAGGGAGCTCTCTGGGGCTTGACGTCTTTTGGAGTTGCGAGGGAGTTGATTTCCTACTTCTAAGTTTGGACTGAATCCGT
GGTGTGATTGAGGTGATTGGCGATGTTTGGCTATACCAGCTATATGTAATAATCTCTACTGTATACTACTATTCAACGCATTTTACTATGCGTGCTGCTAGGGTCGGCAATG
ACAATGGCAATCTGACTGACGTGGTCTATTTCTCCATGTGCAGCAGGGAATACGAGCTCCAATGGACCTCGGGAGTGGCACAGTCAATGGCAAGGAAACTGGAGcgcaaaaaa
ccccgcccctgacagggcggggtttttcgcGATCGGAGTGTGCTGTGGTTAGGGGGGGGAAAGGGAGGATTATTGAGTGCCTATTTTTGGGATCAATATGACTGACTATGGTCATGGCTAG
GACTATAGATTTGGTGGTGTAAGATTTCATTGTTCATATTGGATGGGATTGGCATGGAATGAAAGAACTGAAATGAGGGGAGAGAGATTCTAGACAAGTATCTATTATGT
ATCTAGTGGATGGATCGAGGTCTAGACGTAGTGAGTATACTAAGTGGACTAGGGACAAGGTAACTATGTAGTAGGTAGTGATGAGTGTATTCCAGAAGTACTGACTGCAG
GCGCTGTCAGCACCCACACGCACACGCACACACACTAAATGCACCACTTCCACAATACCCTAATGGAGTGACTACGATGGATGGCAGATGATGGCAGATGGCAGTGGAT
TCCGTCTCGTGGCGTCTCCAGGAACTTCTTCTCCACCTGCGCCCAACTTCCAGCACAACTTGGAAAAGGCGAAAAAGAAAAGAAAAAGAAAAAAAGAAAAAATGCAATTC
AATGGCTCGCCAGGGACACTCCAATCCACGTCAGTGGCCATGTGGACCCCGAAGCCAAACAAACTTTTTACCCCTGCGCCTCTGGCGGCGATTCGTTGAAACGGCCAGGA
ACAGCAGGAACAGCAGTATGAGCACTAGCCGATCTCTTCGCCGACGGTTGGATGCCTTGCCGCCGCAAAAAGCTATATAAGGATGCATCTCGCTGCACCTTTTGCTTCCTC
TTCAATCCATCAGCAAATCCACTGACACTCTCTTGATCAGCTTTGTCTGACCTGTCATTCTCTCTCTTTTTTGATACATCTGATTTTAGTCTGTCGATCAGACTAGTCTGCCT
TCCTTTTAGTCCTCTCTCTTTTGGACTTTACTACTACACATACAACAAACCACTTTCTCTTTTTCAGAGGAACAACAACAGTCTTTCTTGACAACCCAAAATCTCATCTTTTT
TGAGTCAATCTCTTCAAACAAATCTCATCAAACATGTCcTCAACCACGTCcTCATCAAGATCAGACCTTGAAAAGGTCCCCGTACCACAGGTCACCCCTAGAGACAGTGAC
TCCGATAAGGGATCCCTCTCTCCGGAGCCTTCGACCCTCGAGGCTCAGTCATCCGAGAAGCCACCGCATCATATCTTCACACGaTCTCGCAAGCTGCAAATGGTTTGCATC
GTCTCCCTCGCTGCCATATTTTCTCCGCTTTCGTCGAACATTTACTTCCCTGCCCTGGATGATGTCTCGAAATCCCTCAACATCAGCATGTCGCTCGCAACACTCACCATCA
CGGTGTACATGATCGTCCAAGGCCTCGCTCCCAGCTTCTGGGGTTCCATGTCAGACGCCACAGGTAGACGGCCTGTCTTTATTGGAACATTCATTGTTTACCTCGTAGCCA
ATATTGCTCTGGCCGAATCCAAGAACTATGGTGAGCTCATGGCCTTCCGAGCCTTGCAGGCTGCTGGTAGCGCAGCCACCATCTCAATCGGAGCTGGAGTGATTGGTGAT
ATCACAAACTCGGAAGAAAGAGGTAGCTTGGTGGGTATCTTCGGTGGAGTTCGCATGCTTGGACAGGGAATCGGGCCGGTTTTCGGCGGCATTTTCACCCAGTATCTCGG
ATATCGATCTATCTTTTGGTTCCTCACGATTGCTGGAGGCGTGAGTCTCCTGTCCATTCTGGTGCTTCTTCCGGAGACATTGAGgCCAATTGCTGGAAATGGAACTGTGAAG
CTCAATGGCATTCATAAGCCCTTCATCTACACGATCACCGGCCAGACGGGGGTTGTCGAGGGAGCGCAACCGGAAGCGAAAAAGACCAAAACCAGCTGGAAGTCTGTTT
TTGCTCCTTTGACATTCCTCGTCGAAAAGGACGTTTTCATCACCCTGTTCTTTGGAAGTATCGTGTACACAGTGTGGAGCATGGTGACATCCAGTACCACCGACCTCTTCAG
CGAAGTGTACGGCCTGTCATCCCTGGACATTGGACTCACTTTCCTAGGCAATGGCTTTGGATGTATGTCTGGCTCTTATCTGGTCGGCTACCTTATGGATTACAACCACCGT
CTTACCGAACGCGAATATTGCGAGAAACACGGTTATCCGGCAGGCACACGTGTCAATCTGAAATCACACCCCGACTTCCCCATTGAGGTCGCCCGGATGCGCAATACCTG
GTGGGTGATTGCGATCTTCATCGTGACAGTTGCTTTGTACGGCGTGTCTTTGCGGACACATCTGGCGGTGCCTATCATTCTGCAGTACTTCATTGCGTTCTGCTCAACAGGA
CTCTTCACCATCAACAGCGCCCTGGTCATCGATCTTTACCCAGGTGCTAGCGCCAGTGCGACAGCAGTGAACAATCTGATGCGGTGCCTGCTTGGAGCTGGCGGTGTGGCT
ATCGTGCAACCTATCCTGGACGCCTTGAAGCCGGATTATACTTTCCTCTTGCTTGCCGGCATCACCCTTGTGATGACTCCGTTGCTGTACGTCGAAGATCGATGGGGTCCTG
GCTGGCGACATGCCCGCGAAAGGAGACTCAAGGCCAAAGCCAACGGCAACTAGGCTTGGAGAGAAAGGACTTGAAAAAAAAAAGGTGAAGTGGGACTGGTGAAGTAAA
TGTTTGATTCTTCCCCACACTTTCTTGATATGGGTTTTATTTTAGCGGCATTTGGCGATACCACTGTTTTGCAAGCGATACCAGTTAGATTTATAGAAGAATTGATCAGTTTC
ATCGGCTATCTCGCTATTACTTCCTCGTAGCTTTTTAGCTTCATATATGGGTGAGTGGGAAAGGTTTGACATGGTGGTTGAGATAGTATCATTTGGATGGAGATGGAAGTA
TAAAGCAAGAAGTTCTGTTGTTTGGTGTTTAGACTAGACTAGACGTGGCCGGTGAAGTCACGAGCCTCAGCTTAGATAATCTAATGCGGCTGATGTGACTGACCGATGGC
TTTGTTATATGAgGACAGGTCGGTCAATTAGAAGATGTCGCCAGCAAAAGGGTATAATAATGGCTATTATAGAGATAAGAATGTAGAGTATCCCTGTTTTTAAGGATCAGT
ACGTAGTTTTACTATAAAGGTACCCATATATATATTTGCGGAACACGAGGAGGTCGGGAGTACATACTTGTAGTTACTGTTTGTTTGTGACGAGGAATGCCAGTTAGGTAA
GCACCACGTGATGCGTGACCCAAACAAGCGCCCGGTCGTTGCTTGAGGAACCAATTTCTCTGTTTATCCTCCTTCGAACACCACACTTCGACCTTCTGGACACTGCCGCTA
TGAGCCTTCCATAGCCAGCCTGTTTCAGCTCGACAACACCTCTTGATGCCTTGACGACAACTCATCAACTTCTAGCTCCGTTGGTTCACGTTTTTGCTGTCCTTCTTGCGGT
GTGGACCTCGTGCGGTCGAGGGTGGCTCCGTGAAGGATCAAAACAGACGCTATTGCCCGTGGTTACCGATCGTCTCTCACCCAGCAACAAGGAAACCCAGACAGACACT
GCACAGCGTAGCAGATCCTAAAAGAGAAAGAGATGCCTCCTGAGATGGGTAAGAAGCTGCCCGCTCCGGGGAGACGGGCACAAGGTTCATGAACCGAAGAGGATGGGA
ACCCCGACCATGGCCTGCGGCCGCAGCTATCAGTCCTCTCAGCTGACGCCCCTCACGAACCCTCGCACTCTCCCGAAATGCTCTTCGGTGTCCATGGCGCATCATCTCAGC
AGCATCCCATGTCAAACCATGGCTTTGGGCCCACGGACTCTGTGGCCCTGCCGCAACATCACCATCACCATCGACTCCCGCCCCATGCAGCGCTGCGCGCCCCAGGGCGT
CATGGAGTGAATGTGGAATCTCCTCCTTTGCCCTCGGGCCCGCCGAGTGTGGTGGGCCAGCCTGGAATGCCTGATCCAGCCCCCAGGCCTCGAGGACCAAAACTGAAGTT
TACTCCCGAAGAGGACGCTCTACTGGTGGAGTTGAAGGAAAACAAGAACTTGACGTGGAAGCAAATTGCAGACTTCTTCCCGGGCCGAACGAGCGGTACCTTGCAAGTC
CGATACTGCACCAAGCTGAAGGCTAAGGATGTAGCTTGGAGTGACGAAATGGTTCGATTTGCTCCTGATGTATTTCTACGCCTGTCTCACACATGCTAATGAAGGGAATA
GGTACAAAGGCTGCAGCGGGCAATGCACGAGTACGAGAACGATCGGTGGCGCATCATTGCAGGGAAGGTTGGAAATGGCTTCACCCCAGCTGCTTGCCGCGAGAAAGCC
ATGCAGCTCCATGAGTAAAAGCGTTGGGGAATTTTCATATTTATATCTACTGTCGCCAGATTCGGCCCTGCTTGGACCCTCTGATCTCCTTACTCTCCATATTGGTTCAAAT
GTCGGGTCACCGATAGGGCTGGTGGTGCAGGCTTGTTGTAGGCACGGGAGGATGATCAGCATAACTCTGAGTCACTATAGGGACGGGTTGATGTAAGGTATTAAGTGATG
TATGATAATTCATTTTAGCCCGGGGGAACATATGGCGCCGGCATTTGTTCGTTCGCAATGAACCGACACTAGCGTCCGCTCTCGCAGTTTAGCACCGGCTGATCCCGGGCT
GAACGCGGCCATTGCTCGGCCGGGGCATGTGTTCCTTATCTACGGCAGACCGCAGATGACCACTGGAGCAGATTATAGACCCTAAGCCCTAAGCCGGACACCCAATCGAG
TAGGTCTGCGGACCAGGTCACTGCGGGCAGCCGGAGAAGCTCCGCAACCAATCAATCCCCGGCGCTGACTAAGGGCAGGCGACCACGGGCCGAAGCGGCTTCAAACTCA
CCTCAACCTCCAAACTCCCTCATCTCCAAACGTCCTTGCCTTGTCTGCCGTCATTGCAACCCACCCACCAGGACACATGAGCAGCAAGTCCCAGCTCACCTACACTGCCCG
TGCCAGCAAGCATCCCAATGCTCTGGCGAAGAGGCTGTTCGAGATTGCCGAGGCCAAGAAaACCAATGTGACTGTCTCGGCTGACGTTACCACCACTAAGGAGCTACTAG
ATCTTGCTGACCGTCTCGGTCCCTACATTGCCGTGATCAAAACCCACATCGATATCCTCTCTGATTTCAGCAACGAGACtATTGAGGGACTTAAGGCTCTCGCGCAGAAGC
ACAACTTTCTCATCTTCGAGGACCGCAAGTTCATTGACATCGGCAACACGGTCCAGAAGCAATACCACGGCGGTACCCTCCGTATCTCGGAATGGGCCCACATCATCAAC
TGCAGCATTCTCCCTGGTGAGGGTATCGTCGAGGCTCTCGCTCAGACGGCGTCTGCACCGGACTTCGCCTACGGCCCCGAACGCGGTCTGTTGATCTTGGCAGAGATGACC
TCTAAGGGCTCCTTGGCTACCGGCCAGTACACTACTTCCTCGGTCGATTATGCCCGGAAATACAAGAACTTCGTTATGGGATTCGTGTCGACGCGCGCGTTGGGTGAGGTG
CAGTCGGAAGTCAGCTCTCCTTCGGATGAGGAGGACTTTGTGGTtTTCACGACTGGTGTGAACATTAACCTCCA

>pAH028_IC
TGGAGGaAGGGATCCGCCAAGGATGCTCTGGATCCGCATCCGGCCGCTCTTGCGCCCCATCAATCGCCCGACTATAAATCGAACTACTTTCGGCATCTTCTAGACTTCCTA
ATACCGCCTAGTCATAGCAGATTCAAGCTGAGAACACCACAAGTAAATATCACCCATCATGCTTACCCTGACCGTCCCTGAAAACTACGGGTATGTGCCAATTCTACAATT
CCTTGCAGACAATGCCATTCTCCCCATGAAGTCTGATGCTAACTATCCTGCAGCTCTGTCATTGCCGTCGCTCTGGGTGCCATCCCCGTCCTGAGCTTCGTCCATGGCGCCG
TCGTGTCTCGTCTCCGCAAGGAAGCTGATTGCCCCTACCCTCACTGCTATGCGACCGTAGAGCAGTGCAAGACCAACGTAAGCCAACCTCACACAAACAGGATTCCTCGA
GCTAACATACATTCCGAACCGTGCAGCCCAAGGCCGAGCAGTTCAACTGCGCTCAGCGCGCTCATGCCAACTTCCTTGAGAACTCCAGCCAAACTATGCTCTTCCTCCTGG
TAGCTGGACTGAAGTACCCCCAGTTGGCGACTGGCCTCGGAAGCATCTGGGTCCTCGGTCGCTCACTGTTCCTTTACGGATATGTGTACTCCGGCAAGCCGCGGGGTCGCG
GTCGTTTGTACGGCAGCTTCTACTTGCTTGCACAGGGAGCTCTCTGGGGCTTGACGTCTTTTGGAGTTGCGAGGGAGTTGATTTCCTACTTCTAAGTTTGGACTGAATCCGT
GGTGTGATTGAGGTGATTGGCGATGTTTGGCTATACCAGCTATATGTAATAATCTCTACTGTATACTACTATTCAACGCATTTTACTATGCGTGCTGCTAGGGTCGGCAATG
ACAATGGCAATCTGACTGACGTGGTCTATTTCTCCATGTGCAGCAGGGAATACGAGCTCCAATGGACCTCGGGAGTGGCACAGTCAATGGCAAGGAAACTGGAGcgcaaaaaa
ccccgcccctgacagggcggggtttttcgcGATCGGAGACATCACAGCGACGAGGACCACGGCAGTGGTTATCGGAATTAATTAATTATTACGAGGCATAGGCCAGGGTCAATTTGGGTTT
AGTTGGAACCACAGGAAAAGAATTCCCCGTGACGACTGCCGCTTCTTTCTCAAATGCTCCATCAATAGCTTCTTTTCAAACTCGCAACTATTCCTCTGCTGGGAGACTCTC
GaCTTCCCCCACACACGTTTCCTGTCTATCTGGTCACTATTGAGTACTATTGCCTTCTCTTCTCCTTCTTTTTTTCTGGTGATCCTTCCAAGGCCTTCCAAGAAAGGGAATCCT
CTCGATCGGCTAGTCAGCCATGATATCACTTGCCTGGCCGATCCCTCCGTCAGCCCCTCCACTTGCCGCCCCACGATGAGGGAATTCGCGCCTCCCTTTCGTCCTCGGTTTC
CCCTCTTTCGGACTTTTGGGTGAATCAGTCTCCATCTTGACTCCATTCCCATCGTGGCTGAGACACTTCCCACCGGTTGCCGACCGATCCAGGCCAAGCTTTCCTTAAGCTT
TAGCGCTGTGTCCCTGGTCACTGTCTGGTGATTCGCCCATCCGGCCACACGAGAGAGGGGTCCCAAACCTCGATACCCACTCGCTGCGTCCCTCCGTCACTGGCAATGGGC
GTTTACAGCACTAGTCTAGGATTTCGGACACTGTCTGAGCTACAGGAAAGGAGCTCCATGAGTGGCTGCCACTCCCCTCTCGGGGCTGAGGACAACAGCCACTCGTTGGC
TGTTCCCTCCTCCCGTGTTGTCTGAGCTGACGGATGTTGCGGCAGGCCAGAGCAGGGAGCAGGGGACCAGTTTCAATGAGCTAACTGGATTGACGGCCGTTGGCTGATGG
TGGGAGCCTGCTGTTGCGCCCCTCCTTTCTCTTATTTACATCACTGCGCTCCCCCTTTTTCCTGTGGCTGTTTTCTAGCATCACTTTCTTCTCTCTTCTCACTCTTCAATCACA
TCTACACATCCCAATTCGGCACACATGTCcTCAACCACGTCcTCATCAAGATCAGACCTTGAAAAGGTCCCCGTACCACAGGTCACCCCTAGAGACAGTGACTCCGATAAG
GGATCCCTCTCTCCGGAGCCTTCGACCCTCGAGGCTCAGTCATCCGAGAAGCCACCGCATCATATCTTCACACGaTCTCGCAAGCTGCAAATGGTTTGCATCGTCTCCCTCG
CTGCCATATTTTCTCCGCTTTCGTCGAACATTTACTTCCCTGCCCTGGATGATGTCTCGAAATCCCTCAACATCAGCATGTCGCTCGCAACACTCACCATCACGGTGTACAT
GATCGTCCAAGGCCTCGCTCCCAGCTTCTGGGGTTCCATGTCAGACGCCACAGGTAGACGGCCTGTCTTTATTGGAACATTCATTGTTTACCTCGTAGCCAATATTGCTCTG
GCCGAATCCAAGAACTATGGTGAGCTCATGGCCTTCCGAGCCTTGCAGGCTGCTGGTAGCGCAGCCACCATCTCAATCGGAGCTGGAGTGATTGGTGATATCACAAACTC
GGAAGAAAGAGGTAGCTTGGTGGGTATCTTCGGTGGAGTTCGCATGCTTGGACAGGGAATCGGGCCGGTTTTCGGCGGCATTTTCACCCAGTATCTCGGATATCGATCTA
TCTTTTGGTTCCTCACGATTGCTGGAGGCGTGAGTCTCCTGTCCATTCTGGTGCTTCTTCCGGAGACATTGAGgCCAATTGCTGGAAATGGAACTGTGAAGCTCAATGGCAT
TCATAAGCCCTTCATCTACACGATCACCGGCCAGACGGGGGTTGTCGAGGGAGCGCAACCGGAAGCGAAAAAGACCAAAACCAGCTGGAAGTCTGTTTTTGCTCCTTTGA
CATTCCTCGTCGAAAAGGACGTTTTCATCACCCTGTTCTTTGGAAGTATCGTGTACACAGTGTGGAGCATGGTGACATCCAGTACCACCGACCTCTTCAGCGAAGTGTACG
GCCTGTCATCCCTGGACATTGGACTCACTTTCCTAGGCAATGGCTTTGGATGTATGTCTGGCTCTTATCTGGTCGGCTACCTTATGGATTACAACCACCGTCTTACCGAACG
CGAATATTGCGAGAAACACGGTTATCCGGCAGGCACACGTGTCAATCTGAAATCACACCCCGACTTCCCCATTGAGGTCGCCCGGATGCGCAATACCTGGTGGGTGATTG

CGATCTTCATCGTGACAGTTGCTTTGTACGGCGTGTCTTTGCGGACACATCTGGCGGTGCCTATCATTCTGCAGTACTTCATTGCGTTCTGCTCAACAGGACTCTTCACCAT
CAACAGCGCCCTGGTCATCGATCTTTACCCAGGTGCTAGCGCCAGTGCGACAGCAGTGAACAATCTGATGCGGTGCCTGCTTGGAGCTGGCGGTGTGGCTATCGTGCAAC
CTATCCTGGACGCCTTGAAGCCGGATTATACTTTCCTCTTGCTTGCCGGCATCACCCTTGTGATGACTCCGTTGCTGTACGTCGAAGATCGATGGGGTCCTGGCTGGCGAC
ATGCCCGCGAAAGGAGACTCAAGGCCAAAGCCAACGGCAACTAGGCTTGGAGAGAAAGGACTTGAAAAAAAAAAGGTGAAGTGGGACTGGTGAAGTAAATGTTTGATT
CTTCCCCACACTTTCTTGATATGGGTTTTATTTTAGCGGCATTTGGCGATACCACTGTTTTGCAAGCGATACCAGTTAGATTTATAGAAGAATTGATCAGTTTCATCGGCTA
TCTCGCTATTACTTCCTCGTAGCTTTTTAGCTTCATATATGGGTGAGTGGGAAAGGTTTGACATGGTGGTTGAGATAGTATCATTTGGATGGAGATGGAAGTATAAAGCAA
GAAGTTCTGTTGTTTGGTGTTTAGACTAGACTAGACGTGGCCGGTGAAGTCACGAGCCTCAGCTTAGATAATCTAATGCGGCTGATGTGACTGACCGATGGCTTTGTTATA
TGAgGACAGGTCGGTCAATTAGAAGATGTCGCCAGCAAAAGGGTATAATAATGGCTATTATAGAGATAAGAATGTAGAGTATCCCTGTTTTTAAGGATCAGTACGTAGTT
TTACTATAAAGGTACCCATATATATATTTGCGGAACACGAGGAGGTCGGGAGTACATACTTGTAGTTACTGTTTGTTTGTGACGAGGAATGCCAGTTAGGTAAGCACCAC
GTGATGCGTGACCCAAACAAGCGCCCGGTCGTTGCTTGAGGAACCAATTTCTCTGTTTATCCTCCTTCGAACACCACACTTCGACCTTCTGGACACTGCCGCTATGAGCCT
TCCATAGCCAGCCTGTTTCAGCTCGACAACACCTCTTGATGCCTTGACGACAACTCATCAACTTCTAGCTCCGTTGGTTCACGTTTTTGCTGTCCTTCTTGCGGTGTGGACC
TCGTGCGGTCGAGGGTGGCTCCGTGAAGGATCAAAACAGACGCTATTGCCCGTGGTTACCGATCGTCTCTCACCCAGCAACAAGGAAACCCAGACAGACACTGCACAGC
GTAGCAGATCCTAAAAGAGAAAGAGATGCCTCCTGAGATGGGTAAGAAGCTGCCCGCTCCGGGGAGACGGGCACAAGGTTCATGAACCGAAGAGGATGGGAACCCCGA
CCATGGCCTGCGGCCGCAGCTATCAGTCCTCTCAGCTGACGCCCCTCACGAACCCTCGCACTCTCCCGAAATGCTCTTCGGTGTCCATGGCGCATCATCTCAGCAGCATCC
CATGTCAAACCATGGCTTTGGGCCCACGGACTCTGTGGCCCTGCCGCAACATCACCATCACCATCGACTCCCGCCCCATGCAGCGCTGCGCGCCCCAGGGCGTCATGGAG
TGAATGTGGAATCTCCTCCTTTGCCCTCGGGCCCGCCGAGTGTGGTGGGCCAGCCTGGAATGCCTGATCCAGCCCCCAGGCCTCGAGGACCAAAACTGAAGTTTACTCCC
GAAGAGGACGCTCTACTGGTGGAGTTGAAGGAAAACAAGAACTTGACGTGGAAGCAAATTGCAGACTTCTTCCCGGGCCGAACGAGCGGTACCTTGCAAGTCCGATACT
GCACCAAGCTGAAGGCTAAGGATGTAGCTTGGAGTGACGAAATGGTTCGATTTGCTCCTGATGTATTTCTACGCCTGTCTCACACATGCTAATGAAGGGAATAGGTACAA
AGGCTGCAGCGGGCAATGCACGAGTACGAGAACGATCGGTGGCGCATCATTGCAGGGAAGGTTGGAAATGGCTTCACCCCAGCTGCTTGCCGCGAGAAAGCCATGCAGC
TCCATGAGTAAAAGCGTTGGGGAATTTTCATATTTATATCTACTGTCGCCAGATTCGGCCCTGCTTGGACCCTCTGATCTCCTTACTCTCCATATTGGTTCAAATGTCGGGT
CACCGATAGGGCTGGTGGTGCAGGCTTGTTGTAGGCACGGGAGGATGATCAGCATAACTCTGAGTCACTATAGGGACGGGTTGATGTAAGGTATTAAGTGATGTATGATA
ATTCATTTTAGCCCGGGGGAACATATGGCGCCGGCATTTGTTCGTTCGCAATGAACCGACACTAGCGTCCGCTCTCGCAGTTTAGCACCGGCTGATCCCGGGCTGAACGCG
GCCATTGCTCGGCCGGGGCATGTGTTCCTTATCTACGGCAGACCGCAGATGACCACTGGAGCAGATTATAGACCCTAAGCCCTAAGCCGGACACCCAATCGAGTAGGTCT
GCGGACCAGGTCACTGCGGGCAGCCGGAGAAGCTCCGCAACCAATCAATCCCCGGCGCTGACTAAGGGCAGGCGACCACGGGCCGAAGCGGCTTCAAACTCACCTCAAC
CTCCAAACTCCCTCATCTCCAAACGTCCTTGCCTTGTCTGCCGTCATTGCAACCCACCCACCAGGACACATGAGCAGCAAGTCCCAGCTCACCTACACTGCCCGTGCCAGC
AAGCATCCCAATGCTCTGGCGAAGAGGCTGTTCGAGATTGCCGAGGCCAAGAAaACCAATGTGACTGTCTCGGCTGACGTTACCACCACTAAGGAGCTACTAGATCTTGC
TGACCGTCTCGGTCCCTACATTGCCGTGATCAAAACCCACATCGATATCCTCTCTGATTTCAGCAACGAGACtATTGAGGGACTTAAGGCTCTCGCGCAGAAGCACAACTT
TCTCATCTTCGAGGACCGCAAGTTCATTGACATCGGCAACACGGTCCAGAAGCAATACCACGGCGGTACCCTCCGTATCTCGGAATGGGCCCACATCATCAACTGCAGCA
TTCTCCCTGGTGAGGGTATCGTCGAGGCTCTCGCTCAGACGGCGTCTGCACCGGACTTCGCCTACGGCCCCGAACGCGGTCTGTTGATCTTGGCAGAGATGACCTCTAAGG
GCTCCTTGGCTACCGGCCAGTACACTACTTCCTCGGTCGATTATGCCCGGAAATACAAGAACTTCGTTATGGGATTCGTGTCGACGCGCGCGTTGGGTGAGGTGCAGTCGG
AAGTCAGCTCTCCTTCGGATGAGGAGGACTTTGTGGTtTTCACGACTGGTGTGAACATTAACCTCCA

>pAH029_IC
TGGAGGaAGGGATCCGCCAAGGATGCTCTGGATCCGCATCCGGCCGCTCTTGCGCCCCATCAATCGCCCGACTATAAATCGAACTACTTTCGGCATCTTCTAGACTTCCTA
ATACCGCCTAGTCATAGCAGATTCAAGCTGAGAACACCACAAGTAAATATCACCCATCATGCTTACCCTGACCGTCCCTGAAAACTACGGGTATGTGCCAATTCTACAATT
CCTTGCAGACAATGCCATTCTCCCCATGAAGTCTGATGCTAACTATCCTGCAGCTCTGTCATTGCCGTCGCTCTGGGTGCCATCCCCGTCCTGAGCTTCGTCCATGGCGCCG
TCGTGTCTCGTCTCCGCAAGGAAGCTGATTGCCCCTACCCTCACTGCTATGCGACCGTAGAGCAGTGCAAGACCAACGTAAGCCAACCTCACACAAACAGGATTCCTCGA
GCTAACATACATTCCGAACCGTGCAGCCCAAGGCCGAGCAGTTCAACTGCGCTCAGCGCGCTCATGCCAACTTCCTTGAGAACTCCAGCCAAACTATGCTCTTCCTCCTGG
TAGCTGGACTGAAGTACCCCCAGTTGGCGACTGGCCTCGGAAGCATCTGGGTCCTCGGTCGCTCACTGTTCCTTTACGGATATGTGTACTCCGGCAAGCCGCGGGGTCGCG
GTCGTTTGTACGGCAGCTTCTACTTGCTTGCACAGGGAGCTCTCTGGGGCTTGACGTCTTTTGGAGTTGCGAGGGAGTTGATTTCCTACTTCTAAGTTTGGACTGAATCCGT
GGTGTGATTGAGGTGATTGGCGATGTTTGGCTATACCAGCTATATGTAATAATCTCTACTGTATACTACTATTCAACGCATTTTACTATGCGTGCTGCTAGGGTCGGCAATG
ACAATGGCAATCTGACTGACGTGGTCTATTTCTCCATGTGCAGCAGGGAATACGAGCTCCAATGGACCTCGGGAGTGGCACAGTCAATGGCAAGGAAACTGGAGcgcaaaaaa
ccccgccccctgacagggcggggttttttcgcGATCGGAGTAAGAATGGGGAAGGCGAAGGTACCGCCTTTGGGGTCCAGCCACGCGACTCCAACATGGAGGGGCACTGGACTAACATTATT
CCAGCACCGGGATCACGGGCCGAAAGCGGCAAGGCCGCGCACTGCCCCTCTTTTTGGGTGAAAGAGCTGGCAGTAACTAAACTGTACTTTCTGGAGTGAATAATACTACT
ACTATGAAAGACCGCGATGGGCCGATAGTAGTAGTTACTTCCATTACATCATCTCATCCGCCCGGTTCCTCGCCTCCGCGGCAGTCTACGGGTAGGATCGTAGCAAAAAC
CCGGGGGATAGACCCGTCGTCCCGAGCTGGAGTTCCGTATAACCTAGGTAGAAGGTATCAATTGAACCCGAACAACTGGCAAAACATTCTCGAGATCGTAGGAGTGAGT
ACCCGGCGTGATGGAGGGGGAGCACGCTCATTGGTCCGTACGGCAGCTGCCGAGGGGGAGCAGGAGATCCAAATATCGTGAGTCTCCTGCTTTGCCCGGTGTATGAAAC
CGGAAAGGACTGCTGGGGAACTGGGGAGCGGCGCAAGCCGGGAATCCCAGCTGACAATTGACCCATCCTCATGCCGTGGCAGAGCTTGAGGTAGCTTTTGCCCCGTCTGT
CTCCCCGGTGTGCGCATTCGACTGGGCGCGGCATCTGTGCCTCCTCCAGGAGCGGAGGACCCAGTAGTAAGTAGGCCTGACCTGGTCGTTGCGTCAGTCCAGAGGTTCCC
TCCCCTACCCTTTTTCTACTTCCCCTCCCCCGCCGCTCAACTTTTCTTTCCCTTTTACTTTCTCTCTCTCTTCCTCTTCATCCATCCTCTCTTCATCACTTCCCTCTTCCCTTCAT
CCAATTCATCTTCCAAGTGAGaCTTCCTCCCCATCTGTCCCTCCATCTTTCCCATCATCATCTCCCTTCCCAGCTCCTCCCCTCCTCTCGTCTCCTCACGAAGCTTGACTAACC
ATTACCCCGCCACATAGACACATCTAAACACATGTCcTCAACCACGTCcTCATCAAGATCAGACCTTGAAAAGGTCCCCGTACCACAGGTCACCCCTAGAGACAGTGACTC
CGATAAGGGATCCCTCTCTCCGGAGCCTTCGACCCTCGAGGCTCAGTCATCCGAGAAGCCACCGCATCATATCTTCACACGaTCTCGCAAGCTGCAAATGGTTTGCATCGT
CTCCCTCGCTGCCATATTTTCTCCGCTTTCGTCGAACATTTACTTCCCTGCCCTGGATGATGTCTCGAAATCCCTCAACATCAGCATGTCGCTCGCAACACTCACCATCACG
GTGTACATGATCGTCCAAGGCCTCGCTCCCAGCTTCTGGGGTTCCATGTCAGACGCCACAGGTAGACGGCCTGTCTTTATTGGAACATTCATTGTTTACCTCGTAGCCAAT
ATTGCTCTGGCCGAATCCAAGAACTATGGTGAGCTCATGGCCTTCCGAGCCTTGCAGGCTGCTGGTAGCGCAGCCACCATCTCAATCGGAGCTGGAGTGATTGGTGATAT
CACAAACTCGGAAGAAAGAGGTAGCTTGGTGGGTATCTTCGGTGGAGTTCGCATGCTTGGACAGGGAATCGGGCCGGTTTTCGGCGGCATTTTCACCCAGTATCTCGGAT
ATCGATCTATCTTTTGGTTCCTCACGATTGCTGGAGGCGTGAGTCTCCTGTCCATTCTGGTGCTTCTTCCGGAGACATTGAGgCCAATTGCTGGAAATGGAACTGTGAAGCT
CAATGGCATTCATAAGCCCTTCATCTACACGATCACCGGCCAGACGGGGGTTGTCGAGGGAGCGCAACCGGAAGCGAAAAAGACCAAAACCAGCTGGAAGTCTGTTTTT
GCTCCTTTGACATTCCTCGTCGAAAAGGACGTTTTCATCACCCTGTTCTTTGGAAGTATCGTGTACACAGTGTGGAGCATGGTGACATCCAGTACCACCGACCTCTTCAGC
GAAGTGTACGGCCTGTCATCCCTGGACATTGGACTCACTTTCCTAGGCAATGGCTTTGGATGTATGTCTGGCTCTTATCTGGTCGGCTACCTTATGGATTACAACCACCGTC
TTACCGAACGCGAATATTGCGAGAAACACGGTTATCCGGCAGGCACACGTGTCAATCTGAAATCACACCCCGACTTCCCCATTGAGGTCGCCCGGATGCGCAATACCTGG
TGGGTGATTGCGATCTTCATCGTGACAGTTGCTTTGTACGGCGTGTCTTTGCGGACACATCTGGCGGTGCCTATCATTCTGCAGTACTTCATTGCGTTCTGCTCAACAGGAC
TCTTCACCATCAACAGCGCCCTGGTCATCGATCTTTACCCAGGTGCTAGCGCCAGTGCGACAGCAGTGAACAATCTGATGCGGTGCCTGCTTGGAGCTGGCGGTGTGGCTA
TCGTGCAACCTATCCTGGACGCCTTGAAGCCGGATTATACTTTCCTCTTGCTTGCCGGCATCACCCTTGTGATGACTCCGTTGCTGTACGTCGAAGATCGATGGGGTCCTGG
CTGGCGACATGCCCGCGAAAGGAGACTCAAGGCCAAAGCCAACGGCAACTAGGCTTGGAGAGAAAGGACTTGAAAAAAAAAAGGTGAAGTGGGACTGGTGAAGTAAAT
GTTTGATTCTTCCCCACACTTTCTTGATATGGGTTTTATTTTAGCGGCATTTGGCGATACCACTGTTTTGCAAGCGATACCAGTTAGATTTATAGAAGAATTGATCAGTTTC
ATCGGCTATCTCGCTATTACTTCCTCGTAGCTTTTTAGCTTCATATATGGGTGAGTGGGAAAGGTTTGACATGGTGGTTGAGATAGTATCATTTGGATGGAGATGGAAGTA
TAAAGCAAGAAGTTCTGTTGTTTGGTGTTTAGACTAGACTAGACGTGGCCGGTGAAGTCACGAGCCTCAGCTTAGATAATCTAATGCGGCTGATGTGACTGACCGATGGC
TTTGTTATATGAgGACAGGTCGGTCAATTAGAAGATGTCGCCAGCAAAAGGGTATAATAATGGCTATTATAGAGATAAGAATGTAGAGTATCCCTGTTTTTAAGGATCAGT
ACGTAGTTTTACTATAAAGGTACCCATATATATATTTGCGGAACACGAGGAGGTCGGGAGTACATACTTGTAGTTACTGTTTGTTTGTGACGAGGAATGCCAGTTAGGTAA
GCACCACGTGATGCGTGACCCAAACAAGCGCCCGGTCGTTGCTTGAGGAACCAATTTCTCTGTTTATCCTCCTTCGAACACCACACTTCGACCTTCTGGACACTGCCGCTA
TGAGCCTTCCATAGCCAGCCTGTTTCAGCTCGACAACACCTCTTGATGCCTTGACGACAACTCATCAACTTCTAGCTCCGTTGGTTCACGTTTTTGCTGTCCTTCTTGCGGT
GTGGACCTCGTGCGGTCGAGGGTGGCTCCGTGAAGGATCAAAACAGACGCTATTGCCCGTGGTTACCGATCGTCTCTCACCCAGCAACAAGGAAACCCAGACAGACACT
GCACAGCGTAGCAGATCCTAAAAGAGAAAGAGATGCCTCCTGAGATGGGTAAGAAGCTGCCCGCTCCGGGGAGACGGGCACAAGGTTCATGAACCGAAGAGGATGGGA
ACCCCGACCATGGCCTGCGGCCGCAGCTATCAGTCCTCTCAGCTGACGCCCCTCACGAACCCTCGCACTCTCCCGAAATGCTCTTCGGTGTCCATGGCGCATCATCTCAGC
AGCATCCCATGTCAAACCATGGCTTTGGGCCCACGGACTCTGTGGCCCTGCCGCAACATCACCATCACCATCGACTCCCGCCCCATGCAGCGCTGCGCGCCCCAGGGCGT
CATGGAGTGAATGTGGAATCTCCTCCTTTGCCCTCGGGCCCGCCGAGTGTGGTGGGCCAGCCTGGAATGCCTGATCCAGCCCCCAGGCCTCGAGGACCAAAACTGAAGTT
TACTCCCGAAGAGGACGCTCTACTGGTGGAGTTGAAGGAAAACAAGAACTTGACGTGGAAGCAAATTGCAGACTTCTTCCCGGGCCGAACGAGCGGTACCTTGCAAGTC
CGATACTGCACCAAGCTGAAGGCTAAGGATGTAGCTTGGAGTGACGAAATGGTTCGATTTGCTCCTGATGTATTTCTACGCCTGTCTCACACATGCTAATGAAGGGAATA
GGTACAAAGGCTGCAGCGGGCAATGCACGAGTACGAGAACGATCGGTGGCGCATCATTGCAGGGAAGGTTGGAAATGGCTTCACCCCAGCTGCTTGCCGCGAGAAAGCC
ATGCAGCTCCATGAGTAAAAGCGTTGGGGAATTTTCATATTTATATCTACTGTCGCCAGATTCGGCCCTGCTTGGACCCTCTGATCTCCTTACTCTCCATATTGGTTCAAAT
GTCGGGTCACCGATAGGGCTGGTGGTGCAGGCTTGTTGTAGGCACGGGAGGATGATCAGCATAACTCTGAGTCACTATAGGGACGGGTTGATGTAAGGTATTAAGTGATG
TATGATAATTCATTTTAGCCCGGGGGAACATATGGCGCCGGCATTTGTTCGTTCGCAATGAACCGACACTAGCGTCCGCTCTCGCAGTTTAGCACCGGCTGATCCCGGGCT
GAACGCGGCCATTGCTCGGCCGGGGCATGTGTTCCTTATCTACGGCAGACCGCAGATGACCACTGGAGCAGATTATAGACCCTAAGCCCTAAGCCGGACACCCAATCGAG
TAGGTCTGCGGACCAGGTCACTGCGGGCAGCCGGAGAAGCTCCGCAACCAATCAATCCCCGGCGCTGACTAAGGGCAGGCGACCACGGGCCGAAGCGGCTTCAAACTCA
CCTCAACCTCCAAACTCCCTCATCTCCAAACGTCCTTGCCTTGTCTGCCGTCATTGCAACCCACCCACCAGGACACATGAGCAGCAAGTCCCAGCTCACCTACACTGCCCG
TGCCAGCAAGCATCCCAATGCTCTGGCGAAGAGGCTGTTCGAGATTGCCGAGGCCAAGAAaACCAATGTGACTGTCTCGGCTGACGTTACCACCACTAAGGAGCTACTAG
ATCTTGCTGACCGTCTCGGTCCCTACATTGCCGTGATCAAAACCCACATCGATATCCTCTCTGATTTCAGCAACGAGACtATTGAGGGACTTAAGGCTCTCGCGCAGAAGC
ACAACTTTCTCATCTTCGAGGACCGCAAGTTCATTGACATCGGCAACACGGTCCAGAAGCAATACCACGGCGGTACCCTCCGTATCTCGGAATGGGCCCACATCATCAAC
TGCAGCATTCTCCCTGGTGAGGGTATCGTCGAGGCTCTCGCTCAGACGGCGTCTGCACCGGACTTCGCCTACGGCCCCGAACGCGGTCTGTTGATCTTGGCAGAGATGACC
TCTAAGGGCTCCTTGGCTACCGGCCAGTACACTACTTCCTCGGTCGATTATGCCCGGAAATACAAGAACTTCGTTATGGGATTCGTGTCGACGCGCGCGTTGGGTGAGGTG
CAGTCGGAAGTCAGCTCTCCTTCGGATGAGGAGGACTTTGTGGTtTTCACGACTGGTGTGAACATTAACCTCCA

>pAH032_IC
TGGAGGaAGGGATCCGCCAAGGATGCTCTGGATCCGCATCCGGCCGCTCTTGCGCCCCATCAATCGCCCGACTATAAATCGAACTACTTTCGGCATCTTCTAGACTTCCTA
ATACCGCCTAGTCATAGCAGATTCAAGCTGAGAACACCACAAGTAAATATCACCCATCATGCTTACCCTGACCGTCCCTGAAAACTACGGGTATGTGCCAATTCTACAATT
CCTTGCAGACAATGCCATTCTCCCCATGAAGTCTGATGCTAACTATCCTGCAGCTCTGTCATTGCCGTCGCTCTGGGTGCCATCCCCGTCCTGAGCTTCGTCCATGGCGCCG
TCGTGTCTCGTCTCCGCAAGGAAGCTGATTGCCCCTACCCTCACTGCTATGCGACCGTAGAGCAGTGCAAGACCAACGTAAGCCAACCTCACACAAACAGGATTCCTCGA
GCTAACATACATTCCGAACCGTGCAGCCCAAGGCCGAGCAGTTCAACTGCGCTCAGCGCGCTCATGCCAACTTCCTTGAGAACTCCAGCCAAACTATGCTCTTCCTCCTGG
TAGCTGGACTGAAGTACCCCCAGTTGGCGACTGGCCTCGGAAGCATCTGGGTCCTCGGTCGCTCACTGTTCCTTTACGGATATGTGTACTCCGGCAAGCCGCGGGGTCGCG
GTCGTTTGTACGGCAGCTTCTACTTGCTTGCACAGGGAGCTCTCTGGGGCTTGACGTCTTTTGGAGTTGCGAGGGAGTTGATTTCCTACTTCTAAGTTTGGACTGAATCCGT
GGTGTGATTGAGGTGATTGGCGATGTTTGGCTATACCAGCTATATGTAATAATCTCTACTGTATACTACTATTCAACGCATTTTACTATGCGTGCTGCTAGGGTCGGCAATG

ACAATGGCAATCTGACTGACGTGGTCTATTTCTCCATGTGCAGCAGGGAATACGAGCTCCAATGGACCTCGGGAGTGGCACAGTCAATGGCAAGGAAACTGGAGcgcaaaaaa
ccccgcccctgacagggcggggtttttcgcGATCGGAGACTCAAAAGTCAATCCGAAGAACAACTGCCCACTTCAGGCCCCAGTTCAGCCAGTCAGAAGTGTCTGTGCCATCCAGGCACG
GCTTGGTGAGACACTGGCGTCAACGCGGACCGCCAATCAGCCAAACTACTGGGCCTGACCCCGAGGATTCCGATCAGACCCTGCCCAGTTGGTGTGTGCCTGGAAAGTAG
AGTGATTTTTGACTTTCTTTTCCTGTTCTTTATTTGCTTCTTTCACAGTAACAGCTGTAAGATGAGATGGCTGCGTCAGGTACGAGGGAAGGCTCCCCAATCAGACCGAG
CAGGATGCTGAGCTGATTCCGGGCCGCGCCCGACCACATCCAACCATGGAGGTTGCCCTGGGAAGtCCGGTGAGGGTCGGGCAGAGAAGGGTTTAACGGGCATTGGCGG
AGATCCAGAGTTCACGAGCTGTTGGCGGTGGGGGGAGGAAAGAAAGGGAGTGGGTCTGGGGGGGGTCAAAAAAGTAATTGCACCAAGTGGAGTCTAGGCTTCATTCATG
AGAGCGTGAAAAGTGAGAGAAAGAGAGAGAGAGAGAGAGAAAGACAGAGAGAGGGTGATGTATTCTATGGTGATTGTTAGTATTGTTGGTGTGGTGGATCCCTCCTGCGAT
TCACCCCATACCAAATGATACCATACACACCATACATCCATCCATCCATACATACATACATACCACCGGCTCCAATTCTAATACAAATACTCTGCCCTCTCCTCCCTCGTTT
CTCGCCTGCTTCTTTCCCTCTCTTCTTCCACCTCACAGTCCGTCCTCTCTTTGACGCTCGGTCATTCCTTCTTTCCACCCTCATTCCTTGGGTTTTTTGCTCTATACTGCTGTGC
AGTACGCATTCCTTAGACTTCTCTGTCGACCAGAGCATCAATCCTTCCAAAAGAAAAAGTGACCCTACATCGTCCACCAAAATCAAATCACACCTACAACATCCCCTATAT
CAAAACCAAAACAACCTACATCCATATAACACATGTCcTCAACCACGTCcTCATCAAGATCAGACCTTGAAAAGGTCCCCGTACCACAGGTCACCCCTAGAGACAGTGACT
CCGATAAGGGATCCCTCTCTCCGGAGCCTTCGACCCTCGAGGCTCAGTCATCCGAGAAGCCACCGCATCATATCTTCACACGaTCTCGCAAGCTGCAAATGGTTTGCATCG
TCTCCCTCGCTGCCATATTTTCTCCGCTTTCGTCGAACATTTACTTCCCTGCCCTGGATGATGTCTCGAAATCCCTCAACATCAGCATGTCGCTCGCAACACTCACCATCAC
GGTGTACATGATCGTCCAAGGCCTCGCTCCCAGCTTCTGGGGTTCCATGTCAGACGCCACAGGTAGACGGCCTGTCTTTATTGGAACATTCATTGTTTACCTCGTAGCCAA
TATTGCTCTGGCCGAATCCAAGAACTATGGTGAGCTCATGGCCTTCCGAGCCTTGCAGGCTGCTGGTAGCGCAGCCACCATCTCAATCGGAGCTGGAGTGATTGGTGATAT
CACAAACTCGGAAGAAAGAGGTAGCTTGGTGGGTATCTTCGGTGGAGTTCGCATGCTTGGACAGGGAATCGGGCCGGTTTTCGGCGGCATTTTCACCCAGTATCTCGGAT
ATCGATCTATCTTTTGGTTCCTCACGATTGCTGGAGGCGTGAGTCTCCTGTCCATTCTGGTGCTTCTTCCGGAGACATTGAGgCCAATTGCTGGAAATGGAACTGTGAAGCT
CAATGGCATTCATAAGCCCTTCATCTACACGATCACCGGCCAGACGGGGGTTGTCGAGGGAGCGCAACCGGAAGCGAAAAAGACCAAAACCAGCTGGAAGTCTGTTTTT
GCTCCTTTGACATTCCTCGTCGAAAAGGACGTTTTCATCACCCTGTTCTTTGGAAGTATCGTGTACACAGTGTGGAGCATGGTGACATCCAGTACCACCGACCTCTTCAGC
GAAGTGTACGGCCTGTCATCCCTGGACATTGGACTCACTTTCCTAGGCAATGGCTTTGGATGTATGTCTGGCTCTTATCTGGTCGGCTACCTTATGGATTACAACCACCGTC
TTACCGAACGCGAATATTGCGAGAAACACGGTTATCCGGCAGGCACACGTGTCAATCTGAAATCACACCCCGACTTCCCCATTGAGGTCGCCCGGATGCGCAATACCTGG
TGGGTGATTGCGATCTTCATCGTGACAGTTGCTTTGTACGGCGTGTCTTTGCGGACACATCTGGCGGTGCCTATCATTCTGCAGTACTTCATTGCGTTCTGCTCAACAGGAC
TCTTCACCATCAACAGCGCCCTGGTCATCGATCTTTACCCAGGTGCTAGCGCCAGTGCGACAGCAGTGAACAATCTGATGCGGTGCCTGCTTGGAGCTGGCGGTGTGGCTA
TCGTGCAACCTATCCTGGACGCCTTGAAGCCGGATTATACTTTCCTCTTGCTTGCCGGCATCACCCTTGTGATGACTCCGTTGCTGTACGTCGAAGATCGATGGGGTCCTGG
CTGGCGACATGCCCGCGAAAGGAGACTCAAGGCCAAAGCCAACGGCAACTAGGCTTGGAGAGAAAGGACTTGAAAAAAAAAAGGTGAAGTGGGACTGGTGAAGTAAAT
GTTTGATTCTTCCCCACACTTTCTTGATATGGGTTTTATTTTAGCGGCATTTGGCGATACCACTGTTTTGCAAGCGATACCAGTTAGATTTATAGAAGAATTGATCAGTTTC
ATCGGCTATCTCGCTATTACTTCCTCGTAGCTTTTTAGCTTCATATATGGGTGAGTGGGAAAGGTTTGACATGGTGGTTGAGATAGTATCATTTGGATGGAGATGGAAGTA
TAAAGCAAGAAGTTCTGTTGTTTGGTGTTTAGACTAGACTAGACGTGGCCGGTGAAGTCACGAGCCTCAGCTTAGATAATCTAATGCGGCTGATGTGACTGACCGATGGC
TTTGTTATATGAgGACAGGTCGGTCAATTAGAAGATGTCGCCAGCAAAAGGGTATAATAATGGCTATTATAGAGATAAGAATGTAGAGTATCCCTGTTTTTAAGGATCAGT
ACGTAGTTTTACTATAAAGGTACCCATATATATATATTTGCGGAACACGAGGAGGTCGGGAGTACATACTTGTAGTTACTGTTTGTTTGTGACGAGGAATGCCAGTTAGGTAA
GCACCACGTGATGCGTGACCCAAACAAGCGCCCGGTCGTTGCTTGAGGAACCAATTTCTCTGTTTATCCTCCTTCGAACACCACACTTCGACCTTCTGGACACTGCCGCTA
TGAGCCTTCCATAGCCAGCCTGTTTCAGCTCGACAACACCTCTTGATGCCTTGACGACAACTCATCAACTTCTAGCTCCGTTGGTTCACGTTTTTGCTGTCCTTCTTGCGGT
GTGGACCTCGTGCGGTCGAGGGTGGCTCCGTGAAGGATCAAAACAGACGCTATTGCCCGTGGTTACCGATCGTCTCTCACCCAGCAACAAGGAAACCCAGACAGACACT
GCACAGCGTAGCAGATCCTAAAAGAGAAAGAGATGCCTCCTGAGATGGGTAAGAAGCTGCCCGCTCCGGGGAGACGGGCACAAGGTTCATGAACCGAAGAGGATGGGA
ACCCCGACCATGGCCTGCGGCCGCAGCTATCAGTCCTCTCAGCTGACGCCCCTCACGAACCCTCGCACTCTCCCGAAATGCTCTTCGGTGTCCATGGCGCATCATCTCAGC
AGCATCCCATGTCAAACCATGGCTTTGGGCCCACGGACTCTGTGGCCCTGCCGCAACATCACCATCACCATCGACTCCCGCCCCATGCAGCGCTGCGCGCCCCAGGGCGT
CATGGAGTGAATGTGGAATCTCCTCCTTTGCCCTCGGGCCCGCCGAGTGTGGTGGGCCAGCCTGGAATGCCTGATCCAGCCCCCAGGCCTCGAGGACCAAAACTGAAGTT
TACTCCCGAAGAGGACGCTCTACTGGTGGAGTTGAAGGAAAACAAGAACTTGACGTGGAAGCAAATTGCAGACTTCTTCCCGGGCCGAACGAGCGGTACCTTGCAAGTC
CGATACTGCACCAAGCTGAAGGCTAAGGATGTAGCTTGGAGTGACGAAATGGTTCGATTTGCTCCTGATGTATTTCTACGCCTGTCTCACACATGCTAATGAAGGGAATA
GGTACAAAGGCTGCAGCGGGCAATGCACGAGTACGAGAACGATCGGTGGCGCATCATTGCAGGGAAGGTTGGAAATGGCTTCACCCCAGCTGCTTGCCGCGAGAAAGCC
ATGCAGCTCCATGAGTAAAAGCGTTGGGGAATTTTCATATTTATATCTACTGTCGCCAGATTCGGCCCTGCTTGGACCCTCTGATCTCCTTACTCTCCATATTGGTTCAAAT
GTCGGGTCACCGATAGGGCTGGTGGTGCAGGCTTGTTGTAGGCACGGGAGGATGATCAGCATAACTCTGAGTCACTATAGGGACGGGTTGATGTAAGGTATTAAGTGATG
TATGATAATTCATTTTAGCCCGGGGGAACATATGGCGCCGGCATTTGTTCGTTCGCAATGAACCGACACTAGCGTCCGCTCTCGCAGTTTAGCACCGGCTGATCCCGGGCT
GAACGCGGCCATTGCTCGGCCGGGGCATGTGTTCCTTATCTACGGCAGACCGCAGATGACCACTGGAGCAGATTATAGACCCTAAGCCCTAAGCCGGACACCCAATCGAG
TAGGTCTGCGGACCAGGTCACTGCGGGCAGCCGGAGAAGCTCCGCAACCAATCAATCCCCGGCGCTGACTAAGGGCAGGCGACCACGGGCCGAAGCGGCTTCAAACTCA
CCTCAACCTCCAAACTCCCTCATCTCCAAACGTCCTTGCCTTGTCTGCCGTCATTGCAACCCACCCACCAGGACACATGAGCAGCAAGTCCCAGCTCACCTACACTGCCCG
TGCCAGCAAGCATCCCAATGCTCTGGCGAAGAGGCTGTTCGAGATTGCCGAGGCCAAGAAaACCAATGTGACTGTCTCGGCTGACGTTACCACCACTAAGGAGCTACTAG
ATCTTGCTGACCGTCTCGGTCCCTACATTGCCGTGATCAAAACCCACATCGATATCCTCTCTGATTTCAGCAACGAGACtATTGAGGGACTTAAGGCTCTCGCGCAGAAGC
ACAACTTTCTCATCTTCGAGGACCGCAAGTTCATTGACATCGGCAACACGGTCCAGAAGCAATACCACGGCGGTACCCTCCGTATCTCGGAATGGGCCCACATCATCAAC
TGCAGCATTCTCCCTGGTGAGGGTATCGTCGAGGCTCTCGCTCAGACGGCGTCTGCACCGGACTTCGCCTACGGCCCCGAACGCGGTCTGTTGATCTTGGCAGAGATGACC
TCTAAGGGCTCCTTGGCTACCGGCCAGTACACTACTTCCTCGGTCGATTATGCCCGGAAATACAAGAACTTCGTTATGGGATTCGTGTCGACGCGCGCGTTGGGTGAGGTG
CAGTCGGAAGTCAGCTCTCCTTCGGATGAGGAGGACTTTGTGGTtTTCACGACTGGTGTGAACATTAACCTCCA

>pAH031_IC
TGGAGGaAGGGATCCGCCAAGGATGCTCTGGATCCGCATCCGGCCGCTCTTGCGCCCCATCAATCGCCCGACTATAAATCGAACTACTTTCGGCATCTTCTAGACTTCCTA
ATACCGCCTAGTCATAGCAGATTCAAGCTGAGAACACCACAAGTAAATATCACCCATCATGCTTACCCTGACCGTCCCTGAAAACTACGGGTATGTGCCAATTCTACAATT
CCTTGCAGACAATGCCATTCTCCCCATGAAGTCTGATGCTAACTATCCTGCAGCTCTGTCATTGCCGTCGCTCTGGGTGCCATCCCCGTCCTGAGCTTCGTCCATGGCGCCG
TCGTGTCTCGTCTCCGCAAGGAAGCTGATTGCCCCTACCCTCACTGCTATGCGACCGTAGAGCAGTGCAAGACCAACGTAAGCCAACCTCACACAAACAGGATTCCTCGA
GCTAACATACATTCCGAACCGTGCAGCCCAAGGCCGAGCAGTTCAACTGCGCTCAGCGCGCTCATGCCAACTTCCTTGAGAACTCCAGCCAAACTATGCTCTTCCTCCTGG
TAGCTGGACTGAAGTACCCCCAGTTGGCGACTGGCCTCGGAAGCATCTGGGTCCTCGGTCGCTCACTGTTCCTTTACGGATATGTGTACTCCGGCAAGCCGCGGGGTCGCG
GTCGTTTGTACGGCAGCTTCTACTTGCTTGCACAGGGAGCTCTCTGGGGCTTGACGTCTTTTGGAGTTGCGAGGGAGTTGATTTCCTACTTCTAAGTTTGGACTGAATCCGT
GGTGTGATTGAGGTGATTGGCGATGTTTGGCTATACCAGCTATATGTAATAATCTCTACTGTATACTACTATTCAACGCATTTTACTATGCGTGCTGCTAGGGTCGGCAATG
ACAATGGCAATCTGACTGACGTGGTCTATTTCTCCATGTGCAGCAGGGAATACGAGCTCCAATGGACCTCGGGAGTGGCACAGTCAATGGCAAGGAAACTGGAGcgcaaaaaa
ccccgcccctgacagggcggggtttttcgcGATCGGAGAAGTTACTACTTTCGGGACAGATTCGGGGGTTTTTCTGAGGCATTAAACCGCCATCCCACACCAAGTGTCCCACTCTTCACACC
ACTTTCACTGCCGGCGCAGCATCGTGGCTGCAGATGGCAGCCTGAACTGCCCACCGCGGCGGGGCGGGAGGGCAACTGGGGAGTGGCATCACCGGATTCTTCGAGAGGT
GATTGGGCAGGCTGGAAATTTGTTCGCAGGACGAACCACCCAAGAAGGAAGAAGCTGCTGCAAGTTGAGTGGACAGGAGGGGGTGCCAGCGGGACTCTTGATGGGGAG
TGATTCGTCGCTACGGGCATTCCACTCACGAACGAGCCACAGATCGCTGGAGCCGATGACGATCGAGCGGGCGGCGGCGGGTCAAACGCTCGCGCCGTTTCGAAACCAA
TACACCCAGTGATGTCATGGGCTCCATGTTCTCGTTTTTTTGGTAATTGCAATTTCCCCGTCTGGATTGGGAAATCGGAATGAATTCAATGGATCCAGAAAAAAAGTGGGC
TTGGTGTCTCTTCAGGCCAAACCAAAAACGTGCCCATGCCGTCATGGATGGAAGCTTCACTGAGTAAAAAAAATATGCCACCAGGAGCTCCCCCAAGCTCGAGCTGGGAA
ATAAACCAAAACACCAAGGTCAAGTGTGGATGTTCATCCACAAGATGGCTGGTTAAAGTAAAAAAAAAAAAAGTACAAGTGTACATTCAGCAGCAAACCGAAAGTCAAA
GAAATGACCCTGCATAATAGAATTAGTAGCAGCAACAGCATCATACCCCTCATCAACACAACCCCCTCCACCCCACTTGCTGTTCTTGCCTGTAAAGTTGCCTTCTCAACG
AGAGCCCCTCCTCGCCGATACCACCAAGTGACTTCTTCCCCTCCCACCCGCTATTTGTACTCATCCCTCGGCTTCCTTAGCTATTCTTCTTCCTCCTCAGTTATACCCAATCC
CTTCTATACTCCATCACATCGATACCCCTATACAACCCATGTCcTCAACCACGTCcTCATCAAGATCAGACCTTGAAAAGGTCCCCGTACCACAGGTCACCCCTAGAGACA
GTGACTCCGATAAGGGATCCCTCTCTCCGGAGCCTTCGACCCTCGAGGCTCAGTCATCCGAGAAGCCACCGCATCATATCTTCACACGaTCTCGCAAGCTGCAAATGGTTT
GCATCGTCTCCCTCGCTGCCATATTTTCTCCGCTTTCGTCGAACATTTACTTCCCTGCCCTGGATGATGTCTCGAAATCCCTCAACATCAGCATGTCGCTCGCAACACTCAC
CATCACGGTGTACATGATCGTCCAAGGCCTCGCTCCCAGCTTCTGGGGTTCCATGTCAGACGCCACAGGTAGACGGCCTGTCTTTATTGGAACATTCATTGTTTACCTCGT
AGCCAATATTGCTCTGGCCGAATCCAAGAACTATGGTGAGCTCATGGCCTTCCGAGCCTTGCAGGCTGCTGGTAGCGCAGCCACCATCTCAATCGGAGCTGGAGTGATTG
GTGATATCACAAACTCGGAAGAAAGAGGTAGCTTGGTGGGTATCTTCGGTGGAGTTCGCATGCTTGGACAGGGAATCGGGCCGGTTTTCGGCGGCATTTTCACCCAGTAT
CTCGGATATCGATCTATCTTTTGGTTCCTCACGATTGCTGGAGGCGTGAGTCTCCTGTCCATTCTGGTGCTTCTTCCGGAGACATTGAGgCCAATTGCTGGAAATGGAACTG
TGAAGCTCAATGGCATTCATAAGCCCTTCATCTACACGATCACCGGCCAGACGGGGGTTGTCGAGGGAGCGCAACCGGAAGCGAAAAAGACCAAAACCAGCTGGAAGTC
TGTTTTTGCTCCTTTGACATTCCTCGTCGAAAAGGACGTTTTCATCACCCTGTTCTTTGGAAGTATCGTGTACACAGTGTGGAGCATGGTGACATCCAGTACCACCGACCTC
TTCAGCGAAGTGTACGGCCTGTCATCCCTGGACATTGGACTCACTTTCCTAGGCAATGGCTTTGGATGTATGTCTGGCTCTTATCTGGTCGGCTACCTTATGGATTACAACC
ACCGTCTTACCGAACGCGAATATTGCGAGAAACACGGTTATCCGGCAGGCACACGTGTCAATCTGAAATCACACCCCGACTTCCCCATTGAGGTCGCCCGGATGCGCAAT
ACCTGGTGGGTGATTGCGATCTTCATCGTGACAGTTGCTTTGTACGGCGTGTCTTTGCGGACACATCTGGCGGTGCCTATCATTCTGCAGTACTTCATTGCGTTCTGCTCAA
CAGGACTCTTCACCATCAACAGCGCCCTGGTCATCGATCTTTACCCAGGTGCTAGCGCCAGTGCGACAGCAGTGAACAATCTGATGCGGTGCCTGCTTGGAGCTGGCGGT
GTGGCTATCGTGCAACCTATCCTGGACGCCTTGAAGCCGGATTATACTTTCCTCTTGCTTGCCGGCATCACCCTTGTGATGACTCCGTTGCTGTACGTCGAAGATCGATGGG
GTCCTGGCTGGCGACATGCCCGCGAAAGGAGACTCAAGGCCAAAGCCAACGGCAACTAGGCTTGGAGAGAAAGGACTTGAAAAAAAAAAGGTGAAGTGGGACTGGTGA
AGTAAATGTTTGATTCTTCCCCACACTTTCTTGATATGGGTTTTATTTTAGCGGCATTTGGCGATACCACTGTTTTGCAAGCGATACCAGTTAGATTTATAGAAGAATTGAT
CAGTTTCATCGGCTATCTCGCTATTACTTCCTCGTAGCTTTTTAGCTTCATATATGGGTGAGTGGGAAAGGTTTGACATGGTGGTTGAGATAGTATCATTTGGATGGAGATG
GAAGTATAAAGCAAGAAGTTCTGTTGTTTGGTGTTTAGACTAGACTAGACGTGGCCGGTGAAGTCACGAGCCTCAGCTTAGATAATCTAATGCGGCTGATGTGACTGACC
GATGGCTTTGTTATATGAgGACAGGTCGGTCAATTAGAAGATGTCGCCAGCAAAAGGGTATAATAATGGCTATTATAGAGATAAGAATGTAGAGTATCCCTGTTTTTAAG
GATCAGTACGTAGTTTTACTATAAAGGTACCCATATATATATATTTGCGGAACACGAGGAGGTCGGGAGTACATACTTGTAGTTACTGTTTGTTTGTGACGAGGAATGCCAGT
TAGGTAAGCACCACGTGATGCGTGACCCAAACAAGCGCCCGGTCGTTGCTTGAGGAACCAATTTCTCTGTTTATCCTCCTTCGAACACCACACTTCGACCTTCTGGACACT
GCCGCTATGAGCCTTCCATAGCCAGCCTGTTTCAGCTCGACAACACCTCTTGATGCCTTGACGACAACTCATCAACTTCTAGCTCCGTTGGTTCACGTTTTTGCTGTCCTTC
TTGCGGTGTGGACCTCGTGCGGTCGAGGGTGGCTCCGTGAAGGATCAAAACAGACGCTATTGCCCGTGGTTACCGATCGTCTCTCACCCAGCAACAAGGAAACCCAGACA
GACACTGCACAGCGTAGCAGATCCTAAAAGAGAAAGAGATGCCTCCTGAGATGGGTAAGAAGCTGCCCGCTCCGGGGAGACGGGCACAAGGTTCATGAACCGAAGAGG
ATGGGAACCCCGACCATGGCCTGCGGCCGCAGCTATCAGTCCTCTCAGCTGACGCCCCTCACGAACCCTCGCACTCTCCCGAAATGCTCTTCGGTGTCCATGGCGCATCAT
CTCAGCAGCATCCCATGTCAAACCATGGCTTTGGGCCCACGGACTCTGTGGCCCTGCCGCAACATCACCATCACCATCGACTCCCGCCCCATGCAGCGCTGCGCGCCCCAG
GGCGTCATGGAGTGAATGTGGAATCTCCTCCTTTGCCCTCGGGCCCGCCGAGTGTGGTGGGCCAGCCTGGAATGCCTGATCCAGCCCCCAGGCCTCGAGGACCAAAACTG
AAGTTTACTCCCGAAGAGGACGCTCTACTGGTGGAGTTGAAGGAAAACAAGAACTTGACGTGGAAGCAAATTGCAGACTTCTTCCCGGGCCGAACGAGCGGTACCTTGC
AAGTCCGATACTGCACCAAGCTGAAGGCTAAGGATGTAGCTTGGAGTGACGAAATGGTTCGATTTGCTCCTGATGTATTTCTACGCCTGTCTCACACATGCTAATGAAGG
GAATAGGTACAAAGGCTGCAGCGGGCAATGCACGAGTACGAGAACGATCGGTGGCGCATCATTGCAGGGAAGGTTGGAAATGGCTTCACCCCAGCTGCTTGCCGCGAGA
AAGCCATGCAGCTCCATGAGTAAAAGCGTTGGGGAATTTTCATATTTATATCTACTGTCGCCAGATTCGGCCCTGCTTGGACCCTCTGATCTCCTTACTCTCCATATTGGTT

CAAATGTCGGGTCACCGATAGGGCTGGTGGTGCAGGCTTGTTGTAGGCACGGGAGGATGATCAGCATAACTCTGAGTCACTATAGGGACGGGTTGATGTAAGGTATTAAG
TGATGTATGATAATTCATTTTAGCCCGGGGGAACATATGGCGCCGGCATTTGTTCGTTCGCAATGAACCGACACTAGCGTCCGCTCTCGCAGTTTAGCACCGGCTGATCCC
GGGCTGAACGCGGCCATTGCTCGGCCGGGGCATGTGTTCCTTATCTACGGCAGACCGCAGATGACCACTGGAGCAGATTATAGACCCTAAGCCCTAAGCCGGACACCCAA
TCGAGTAGGTCTGCGGACCAGGTCACTGCGGGCAGCCGGAGAAGCTCCGCAACCAATCAATCCCCGGCGCTGACTAAGGGCAGGCGACCACGGGCCGAAGCGGCTTCAA
ACTCACCTCAACCTCCAAACTCCCTCATCTCCAAACGTCCTTGCCTTGTCTGCCGTCATTGCAACCCACCCACCAGGACACATGAGCAGCAAGTCCCAGCTCACCTACACT
GCCCGTGCCAGCAAGCATCCCAATGCTCTGGCGAAGAGGCTGTTCGAGATTGCCGAGGCCAAGAAaACCAATGTGACTGTCTCGGCTGACGTTACCACCACTAAGGAGCT
ACTAGATCTTGCTGACCGTCTCGGTCCCTACATTGCCGTGATCAAAACCCACATCGATATCCTCTCTGATTTCAGCAACGAGACtATTGAGGGACTTAAGGCTCTCGCGCAG
AAGCACAACTTTCTCATCTTCGAGGACCGCAAGTTCATTGACATCGGCAACACGGTCCAGAAGCAATACCACGGCGGTACCCTCCGTATCTCGGAATGGGCCCACATCAT
CAACTGCAGCATTCTCCCTGGTGAGGGTATCGTCGAGGCTCTCGCTCAGACGGCGTCTGCACCGGACTTCGCCTACGGCCCCGAACGCGGTCTGTTGATCTTGGCAGAGAT
GACCTCTAAGGGCTCCTTGGCTACCGGCCAGTACACTACTTCCTCGGTCGATTATGCCCGGAAATACAAGAACTTCGTTATGGGATTCGTGTCGACGCGCGCGTTGGGTGA
GGTGCAGTCGGAAGTCAGCTCTCCTTCGGATGAGGAGGACTTTGTGGTtTTCACGACTGGTGTGAACATTAACCTCCA

>pAH033_IC
TGGAGGaAGGGATCCGCCAAGGATGCTCTGGATCCGCATCCGGCCGCTCTTGCGCCCCATCAATCGCCCGACTATAAATCGAACTACTTTCGGCATCTTCTAGACTTCCTA
ATACCGCCTAGTCATAGCAGATTCAAGCTGAGAACACCACAAGTAAATATCACCCATCATGCTTACCCTGACCGTCCCTGAAAACTACGGGTATGTGCCAATTCTACAATT
CCTTGCAGACAATGCCATTCTCCCCATGAAGTCTGATGCTAACTATCCTGCAGCTCTGTCATTGCCGTCGCTCTGGGTGCCATCCCCGTCCTGAGCTTCGTCCATGGCGCCG
TCGTGTCTCGTCTCCGCAAGGAAGCTGATTGCCCCTACCCTCACTGCTATGCGACCGTAGAGCAGTGCAAGACCAACGTAAGCCAACCTCACACAAACAGGATTCCTCGA
GCTAACATACATTCCGAACCGTGCAGCCCAAGGCCGAGCAGTTCAACTGCGCTCAGCGCGCTCATGCCAACTTCCTTGAGAACTCCAGCCAAACTATGCTCTTCCTCCTGG
TAGCTGGACTGAAGTACCCCCAGTTGGCGACTGGCCTCGGAAGCATCTGGGTCCTCGGTCGCTCACTGTTCCTTTACGGATATGTGTACTCCGGCAAGCCGCGGGGTCGCG
GTCGTTTGTACGGCAGCTTCTACTTGCTTGCACAGGGAGCTCTCTGGGGCTTGACGTCTTTTGGAGTTGCGAGGGAGTTGATTTCCTACTTCTAAGTTTGGACTGAATCCGT
GGTGTGATTGAGGTGATTGGCGATGTTTGGCTATACCAGCTATATGTAATAATCTCTACTGTATACTACTATTCAACGCATTTTACTATGCGTGCTGCTAGGGTCGGCAATG
ACAATGGCAATCTGACTGACGTGGTCTATTTCTCCATGTGCAGCAGGGAATACGAGCTCCAATGGACCTCGGGAGTGGCACAGTCAATGGCAAGGAAACTGGAGcgcaaaaaa
ccccgcccctgacagggcggggtttttcgcGATCGGAGCTTTTGGCTTGTGATCTTGATTGCTAGAGATGTATATCCTCCCGGATACCGCGGAGTGCGCCATTTCTGGTTACCTTCTCTTTC
CCTTTTTGTCTCGATCGTGAGGCGGAACGCAGGATGAAGtCACGGCTTCTCCATCGCGGCCCACCAACCAACAATGTCCTTGGACGCCCAACTCTCCATCTACTGGTCATTG
GTCCAATGCAGAGACTCCGTCGAGCTCAAATGGGCCGGCCAACCCCGAGTCGTCAGGGGCAGCGGCAGCGACGAGCTAAATTAGACCACTGATAAGACGCGATAGTCCA
AAGTCTGACCGTCACATTGTGCCAGGCAGATAAGTTGAATCGTGTGACTGGATGTTGGCTAACGTATGGCGTCTCCGGAGGCCCGACGGACCCTGCGCGATCGGCGGTGG
AGCGCAATCTAAGGACATCCGCGCCTAAGATATCTACCCTTCAGCAGTTCAGCCTAGCCCTGCAGACTTGTCGGACCAGTGCTATCGTGATCGGCCCCCACGGTCGAATG
AGCTCTTGTCTCTTTCCGTCAGACCCTGCCAGTTAATCTGCTATCTACTCCGCGGTAACATCGTGCCTGTCTCCACTAAGGCAGGGTCCAGGGCTGTATGTCTTACTTGCA
CCGAGTCGGCCGCCGGTTGGCTCTGTCTTGGCAATTGCGAATATCCTCACGGGCGACGGACGACACGGATTTGGACGGACATGCGGAGATCTTCGTCGGTTTATTCCTGG
AAGGGACATCATCTCTTCCATCATGACGGCTGCCATAGCGGGGACTCTGAGACATTTTTGCTCTGAAGAGCATGGTCGACTTGGATGATGGAGGAGTTGATCGAGGTCA
ATGAGGAGAGGCTTGCAAGTATAAGAAGAGACTGCTCGACCAGCAGAATGGATCTTCTTGTTCATCAACCAAGAGTCCAAGGCTTCTTTGTCTGGTTCTATCTCTTCTCCG
AACTCTCTTGCTTGACATTCTCGTGGTCAAACATGTCcTCAACCACGTCcTCATCAAGATCAGACCTTGAAAAGGTCCCCGTACCACAGGTCACCCCTAGAGACAGTGACTC
CGATAAGGGATCCCTCTCTCCGGAGCCTTCGACCCTCGAGGCTCAGTCATCCGAGAAGCCACCGCATCATATCTTCACACGaTCTCGCAAGCTGCAAATGGTTTGCATCGT
CTCCCTCGCTGCCATATTTTCTCCGCTTTCGTCGAACATTTACTTCCCTGCCCTGGATGATGTCTCGAAATCCCTCAACATCAGCATGTCGCTCGCAACACTCACCATCACG
GTGTACATGATCGTCCAAGGCCTCGCTCCCAGCTTCTGGGGTTCCATGTCAGACGCCACAGGTAGACGGCCTGTCTTTATTGGAACATTCATTGTTTACCTCGTAGCCAAT
ATTGCTCTGGCCGAATCCAAGAACTATGGTGAGCTCATGGCCTTCCGAGCCTTGCAGGCTGCTGGTAGCGCAGCCACCATCTCAATCGGAGCTGGAGTGATTGGTGATAT
CACAAACTCGGAAGAAAGAGGTAGCTTGGTGGGTATCTTCGGTGGAGTTCGCATGCTTGGACAGGGAATCGGGCCGGTTTTCGGCGGCATTTTCACCCAGTATCTCGGAT
ATCGATCTATCTTTTGGTTCCTCACGATTGCTGGAGGCGTGAGTCTCCTGTCCATTCTGGTGCTTCTTCCGGAGACATTGAGgCCAATTGCTGGAAATGGAACTGTGAAGCT
CAATGGCATTCATAAGCCCTTCATCTACACGATCACCGGCCAGACGGGGGTTGTCGAGGGAGCGCAACCGGAAGCGAAAAAGACCAAAACCAGCTGGAAGTCTGTTTTT
GCTCCTTTGACATTCCTCGTCGAAAAGGACGTTTTCATCACCCTGTTCTTTGGAAGTATCGTGTACACAGTGTGGAGCATGGTGACATCCAGTACCACCGACCTCTTCAGC
GAAGTGTACGGCCTGTCATCCCTGGACATTGGACTCACTTTCCTAGGCAATGGCTTTGGATGTATGTCTGGCTCTTATCTGGTCGGCTACCTTATGGATTACAACCACCGTC
TTACCGAACGCGAATATTGCGAGAAACACGGTTATCCGGCAGGCACACGTGTCATCTGAAATCACACCCGACTTCCCCATTGAGGTCGCCCGGATGCGCAATACCTGG
TGGGTGATTGCGATCTTCATCGTGACAGTTGCTTTGTACGGCGTGTCTTTGCGGACACATCTGGCGGTGCCTATCATTCTGCAGTACTTCATTGCGTTCTGCTCAACAGGAC
TCTTCACCATCAACAGCGCCCTGGTCATCGATCTTTACCCAGGTGCTAGCGCCAGTGCGACAGCAGTGAACAATCTGATGCGGTGCCTGCTTGGAGCTGGCGGTGTGGCTA
TCGTGCAACCTATCCTGGACGCCTTGAAGCCGGATTATACTTTCCTCTTGCTTGCCGGCATCACCCTTGTGATGACTCCGTTGCTGTACGTCGAAGATCGATGGGGTCCTGG
CTGGCGACATGCCCGCGAAAGGAGACTCAAGGCCAAAGCCAACGGCAACTAGGCTTGGAGAGAAAGGACTTGAAAAAAAAAAGGTGAAGTGGGACTGGTGAAGTAAAT
GTTTGATTCTTCCCCACACTTTCTTGATATGGGTTTTATTTTAGCGGCATTTGGCGATACCACTGTTTTGCAAGCGATACCAGTTAGATTTATAGAAGAATTGATCAGTTTC
ATCGGCTATCTCGCTATTACTTCCTCGTAGCTTTTAGCTTCATATATGGGTGAGTGGGAAAGGTTTGACATGGTGGTTGAGATAGTATCATTTGGATGGAGATGGAAGTA
TAAAGCAAGAAGTTCTGTTGTTTGGTGTTTAGACTAGACTAGACGTGGCCGGTGAAGTCACGAGCCTCAGCTTAGATAATCTAATGCGGCTGATGTGACTGACCGATGGC
TTTGTTATATGAgGACAGGTCGGTCAATTAGAAGATGTCGCCAGCAAAAGGGTATAATAATGGCTATTATAGAGATAAGAATGTAGAGTATCCCTGTTTTTAAGGATCAGT
ACGTAGTTTTACTATAAAGGTACCCATATATATATTTGCGGAACACGAGGAGGTCGGGAGTACATACTTGTAGTTACTGTTGTTTGTGACGAGGAATGCCAGTTAGGTAA
GCACCACGTGATGCGTGACCCAAACAAGCGCCCGGTCGTTGCTTGAGGAACCAATTTCTCTGTTTATCCTCCTTCGAACACCACACTTCGACCTTCTGGACACTGCCGCTA
TGAGCCTTCCATAGCCAGCCTGTTTCAGCTCGACAACACCTCTTGATGCCTTGACGACAACTCATCAACTTCTAGCTCCGTTGGTTCACGTTTTTGCTGTCCTTCTTGCGGT
GTGGACCTCGTGCGGTCGAGGGTGGCTCCGTGAAGGATCAAAACAGACGCTATTGCCCGTGGTTACCGATCGTCTCTCACCCAGCAACAAGGAAACCCAGACAGACACT
GCACAGCGTAGCAGATCCTAAAAGAGAAAGAGATGCCTCCTGAGATGGGTAAGAAGCTGCCCGCTCCGGGGAGACGGGCACAAGGTTCATGAACCGAAGAGGATGGGA
ACCCCGACCATGGCCTGCGGCCGCAGCTATCAGTCCTCTCAGCTGACGCCCCTCACGAACCCTCGCACTCTCCCGAAATGCTCTTCGGTGTCCATGGCGCATCATCTCAGC
AGCATCCCATGTCAAACATGGCTTTGGGCCCACGGACTCTGTGGCCTGCCGCAACATCACCATCACCATCGACTCCCGCCCCATCAGGTCAGCAGCGCTGCGCGCCCCAGGGCGT
CATGGAGTGAATGTGGAATCTCCTCCTTTGCCCTCGGGCCCGCCGAGTGTGGTGGGCCAGCCTGGAATGCCTGATCCAGCCCCCAGGCCTCGAGGACCAAAACTGAAGTT
TACTCCCGAAGAGGACGCTCTACTGGTGGAGTTGAAGGAAAACAAGAACTTGACGTGGAAGCAAATTGCAGACTTCTTCCCGGGCCGAACGAGCGGTACCTTGCAAGTC
CGATACTGCACCAAGCTGAAGGCTAAGGATGTAGCTTGGGTGACGAAATGGTTCGATTTGCTCCTGATGTATTTCTACGCCTGTCTCACACATGCTAATGAAGGGAATA
GGTACAAAGGCTGCAGCGGGCAATGCACGAGTACGAGAACGATCGGTGGCGCATCATTGCAGGGAAGGTTGGAAATGGCTTCACCCCAGCTGCTTGCCGCGAGAAAGCC
ATGCAGCTCCATGAGTAAAAGCGTTGGGGAATTTTCATATTTATATCTACTGTCGCCAGATTCGGCCCTGCTTGGACCCTCTGATCTCCTTACTCTCCATATTGGTTCAAAT
GTCGGGTCACCGATAGGGCTGGTGGTGCAGGCTTGTTGTAGGCACGGGAGGATGATCAGCATAACTCTGAGTCACTATAGGGACGGGTTGATGTAAGGTATTAAGTGATG
TATGATAATTCATTTTAGCCCGGGGGAACATATGGCGCCGGCATTTGTTCGTTCGCAATGAACCGACACTAGCGTCCGCTCTCGCAGTTTAGCACCGGCTGATCCCGGGCT
GAACGCGGCCATTGCTCGGCCGGGGCATGTGTTCCTTATCTACGGCAGACCGCAGATGACCACTGGAGCAGATTATAGACCCTAAGCCCTAAGCCGGACACCCAATCGAG
TAGGTCTGCGGACCAGGTCACTGCGGGCAGCCGGAGAAGCTCCGCAACCAATCAATCCCCGGCGCTGACTAAGGGCAGGCGACCACGGGCCGAAGCGGCTTCAAACTCA
CCTCAACCTCCAAACTCCCTCATCTCCAAACGTCCTTGCCTTGTCTGCCGTCATTGCAACCCACCCACCAGGACACATGAGCAGCAAGTCCCAGCTCACCTACACTGCCCG
TGCCAGCAAGCATCCCAATGCTCTGGCGAAGAGGCTGTTCGAGATTGCCGAGGCCAAGAAaACCAATGTGACTGTCTCGGCTGACGTTACCACCACTAAGGAGCTACTAG
ATCTTGCTGACCGTCTCGGTCCCTACATTGCCGTGATCAAAACCCACATCGATATCCTCTCTGATTTCAGCAACGAGACtATTGAGGGACTTAAGGCTCTCGCGCAGAAGC
ACAACTTTCTCATCTTCGAGGACCGCAAGTTCATTGACATCGGCAACACGGTCCAGAAGCAATACCACGGCGGTACCCTCCGTATCTCGGAATGGGCCCACATCATCAAC
TGCAGCATTCTCCCTGGTGAGGGTATCGTCGAGGCTCTCGCTCAGACGGCGTCTGCACCGGACTTCGCCTACGGCCCCGAACGCGGTCTGTTGATCTTGGCAGAGATGACC
TCTAAGGGCTCCTTGGCTACCGGCCAGTACACTACTTCCTCGGTCGATTATGCCCGGAAATACAAGAACTTCGTTATGGGATTCGTGTCGACGCGCGCGTTGGGTGAGGTG
CAGTCGGAAGTCAGCTCTCCTTCGGATGAGGAGGACTTTGTGGTtTTCACGACTGGTGTGAACATTAACCTCCA

>pAH027_IC
TGGAGGaAGGGATCCGCCAAGGATGCTCTGGATCCGCATCCGGCCGCTCTTGCGCCCCATCAATCGCCCGACTATAAATCGAACTACTTTCGGCATCTTCTAGACTTCCTA
ATACCGCCTAGTCATAGCAGATTCAAGCTGAGAACACCACAAGTAAATATCACCCATCATGCTTACCCTGACCGTCCCTGAAAACTACGGGTATGTGCCAATTCTACAATT
CCTTGCAGACAATGCCATTCTCCCCATGAAGTCTGATGCTAACTATCCTGCAGCTCTGTCATTGCCGTCGCTCTGGGTGCCATCCCCGTCCTGAGCTTCGTCCATGGCGCCG
TCGTGTCTCGTCTCCGCAAGGAAGCTGATTGCCCCTACCCTCACTGCTATGCGACCGTAGAGCAGTGCAAGACCAACGTAAGCCAACCTCACACAAACAGGATTCCTCGA
GCTAACATACATTCCGAACCGTGCAGCCCAAGGCCGAGCAGTTCAACTGCGCTCAGCGCGCTCATGCCAACTTCCTTGAGAACTCCAGCCAAACTATGCTCTTCCTCCTGG
TAGCTGGACTGAAGTACCCCCAGTTGGCGACTGGCCTCGGAAGCATCTGGGTCCTCGGTCGCTCACTGTTCCTTTACGGATATGTGTACTCCGGCAAGCCGCGGGGTCGCG
GTCGTTTGTACGGCAGCTTCTACTTGCTTGCACAGGGAGCTCTCTGGGGCTTGACGTCTTTTGGAGTTGCGAGGGAGTTGATTTCCTACTTCTAAGTTTGGACTGAATCCGT
GGTGTGATTGAGGTGATTGGCGATGTTTGGCTATACCAGCTATATGTAATAATCTCTACTGTATACTACTATTCAACGCATTTTACTATGCGTGCTGCTAGGGTCGGCAATG
ACAATGGCAATCTGACTGACGTGGTCTATTTCTCCATGTGCAGCAGGGAATACGAGCTCCAATGGACCTCGGGAGTGGCACAGTCAATGGCAAGGAAACTGGAGcgcaaaaaa
ccccgcccctgacagggcggggtttttcgcGATCGGAGTGCAGCTACTATGAGACTACAGAGAAGAAAATACCGCAGCCTTAGTTGGATTGCGGCCCCCACTGACATGGCCCCATTACCCC
TGGGGACCAGAGAACAGTAATATGATAAACACATGATGTACTAGAGTCATTCCGTGGGACCCGAGCAATCCGCCCGACCCGAGCACCCCAAAACTACGCGCTCTGACCA
ACACGTCCCTGCCTGGCCACCACCACCACTACCACCACCAATCTTGCCAATTGTTGTCTAAATCAGTGGTGTTTGACTCAATTAAATGTCTAGACTCCGGCTAAGGCAGA
TATCAGATATCTAGAGACGTTGAAAAATGAGCAACGAAAATGACTCCTATCGTACTTCCTCGCCTGTGCGTTGAATTACTCCGCCACACACTACGTGAATCCCGCAAGAT
GCAAGAGTCTCGCGCTGTTCAACGCCAGGAGTTTAAGAAGGGCATGTTTATACGAGCAGATAGCAAGCTTCTTACCCGTGAGCTTCGTATCAGTCAAAACACCTAAGCCC
TGTTGATATTTCACCGACAAAAGGCCGGTCCCTAAGCCATGCTCATCTAAATTGAGTTGGTCTATCCTTCTAAATTAATATCGTCAGCACAAGCAGGACCCTTTTCTTGTTT
CCCATCAGATCCCGTTCTCCACGTTGTTCGGTTACCGACTGCCACAGACCATCACTAACTTTAAACGGAAAGTGCTCCGACATAGGGTgTCATTTTGAAACGGAGGATGTG
CATGTCTGTTGCAGATTGGCGTGAGATGGCGGCGCAACCCGCAATAGCTAGTTGGCTTACTTCTTCTTGAATGCCATATTGGACACGGAAATTCGGACAATCATAATGGCA
ATAGTAAGCATTGTTGATTAGATTCAGGTATTTAAGCTGCCTCATCTGCCGCTCCCTCCGTGTTGGTGTCTTATCCTGAACACTTATCCTCTTATTCCTTTCGAATTTCCCTT
GATTGCCGCTCCTCCGCTCTAACGCCCAACCATGTCcTCAACCACGTCcTCATCAAGATCAGACCTTGAAAAGGTCCCCGTACCACAGGTCACCCCTAGAGACAGTGACTC
CGATAAGGGATCCCTCTCTCCGGAGCCTTCGACCCTCGAGGCTCAGTCATCCGAGAAGCCACCGCATCATATCTTCACACGaTCTCGCAAGCTGCAAATGGTTTGCATCGT
CTCCCTCGCTGCCATATTTTCTCCGCTTTCGTCGAACATTTACTTCCCTGCCCTGGATGATGTCTCGAAATCCCTCAACATCAGCATGTCGCTCGCAACACTCACCATCACG
GTGTACATGATCGTCCAAGGCCTCGCTCCCAGCTTCTGGGGTTCCATGTCAGACGCCACAGGTAGACGGCCTGTCTTTATTGGAACATTCATTGTTTACCTCGTAGCCAAT
ATTGCTCTGGCCGAATCCAAGAACTATGGTGAGCTCATGGCCTTCCGAGCCTTGCAGGCTGCTGGTAGCGCAGCCACCATCTCAATCGGAGCTGGAGTGATTGGTGATAT
CACAAACTCGGAAGAAAGAGGTAGCTTGGTGGGTATCTTCGGTGGAGTTCGCATGCTTGGACAGGGAATCGGGCCGGTTTTCGGCGGCATTTTCACCCAGTATCTCGGAT
ATCGATCTATCTTTTGGTTCCTCACGATTGCTGGAGGCGTGAGTCTCCTGTCCATTCTGGTGCTTCTTCCGGAGACATTGAGgCCAATTGCTGGAAATGGAACTGTGAAGCT
CAATGGCATTCATAAGCCCTTCATCTACACGATCACCGGCCAGACGGGGGTTGTCGAGGGAGCGCAACCGGAAGCGAAAAAGACCAAAACCAGCTGGAAGTCTGTTTTT

GCTCCTTTGACATTCCTCGTCGAAAAGGACGTTTTCATCACCCTGTTCTTTGGAAGTATCGTGTACACAGTGTGGAGCATGGTGACATCCAGTACCACCGACCTCTTCAGC
GAAGTGTACGGCCTGTCATCCCTGGACATTGGACTCACTTTCCTAGGCAATGGCTTTGGATGTATGTCTGGCTCTTATCTGGTCGGCTACCTTATGGATTACAACCACCGTC
TTACCGAACGCGAATATTGCGAGAAACACGGTTATCCGGCAGGCACACGTGTCAATCTGAAATCACACCCCGACTTCCCCATTGAGGTCGCCCGGATGCGCAATACCTGG
TGGGTGATTGCGATCTTCATCGTGACAGTTGCTTTGTACGGCGTGTCTTTGCGGACACATCTGGCGGTGCCTATCATTCTGCAGTACTTCATTGCGTTCTGCTCAACAGGAC
TCTTCACCATCAACAGCGCCCTGGTCATCGATCTTTACCCAGGTGCTAGCGCCAGTGCGACAGCAGTGAACAATCTGATGCGGTGCCTGCTTGGAGCTGGCGGTGTGGCTA
TCGTGCAACCTATCCTGGACGCCTTGAAGCCGGATTATACTTTCCTCTTGCTTGCCGGCATCACCCTTGTGATGACTCCGTTGCTGTACGTCGAAGATCGATGGGGTCCTGG
CTGGCGACATGCCCGCGAAAGGAGACTCAAGGCCAAAGCCAACGGCAACTAGGCTTGGAGAGAAAGGACTTGAAAAAAAAAAGGTGAAGTGGGACTGGTGAAGTAAAT
GTTTGATTCTTCCCCACACTTTCTTGATATGGGTTTTATTTTAGCGGCATTTGGCGATACCACTGTTTTGCAAGCGATACCAGTTAGATTTATAGAAGAATTGATCAGTTTC
ATCGGCTATCTCGCTATTACTTCCTCGTAGCTTTTTAGCTTCATATATGGGTGAGTGGGAAAGGTTTGACATGGTGGTTGAGATAGTATCATTTGGATGGAGATGGAAGTA
TAAAGCAAGAAGTTCTGTTGTTTGGTGTTTAGACTAGACTAGACGTGGCCGGTGAAGTCACGAGCCTCAGCTTAGATAATCTAATGCGGCTGATGTGACTGACCGATGGC
TTTGTTATATGAgGACAGGTCGGTCAATTAGAAGATGTCGCCAGCAAAAGGGTATAATAATGGCTATTATAGAGATAAGAATGTAGAGTATCCCTGTTTTTAAGGATCAGT
ACGTAGTTTTACTATAAAGGTACCCATATATATATATTTGCGGAACACGAGGAGGTCGGGAGTACATACTTGTAGTTACTGTTTGTTTGTGACGAGGAATGCCAGTTAGGTAA
GCACCACGTGATGCGTGACCCAAACAAGCGCCCGGTCGTTGCTTGAGGAACCAATTTCTCTGTTTATCCTCCTTCGAACACCACACTTCGACCTTCTGGACACTGCCGCTA
TGAGCCTTCCATAGCCAGCCTGTTTCAGCTCGACAACACCTCTTGATGCCTTGACGACAACTCATCAACTTCTAGCTCCGTTGGTTCACGTTTTTGCTGTCCTTCTTGCGGT
GTGGACCTCGTGCGGTCGAGGGTGGCTCCGTGAAGGATCAAAACAGACGCTATTGCCCGTGGTTACCGATCGTCTCTCACCCAGCAACAAGGAAACCCAGACAGACACT
GCACAGCGTAGCAGATCCTAAAAGAGAAAGAGATGCCTCCTGAGATGGGTAAGAAGCTGCCCGCTCCGGGGAGACGGGCACAAGGTTCATGAACCGAAGAGGATGGGA
ACCCCGACCATGGCCTGCGGCCGCAGCTATCAGTCCTCTCAGCTGACGCCCCTCACGAACCCTCGCACTCTCCCGAAATGCTCTTCGGTGTCCATGGCGCATCATCTCAGC
AGCATCCCATGTCAAACCATGGCTTTGGGCCCACGGACTCTGTGGCCCTGCCGCAACATCACCATCACCATCGACTCCCGCCCCATGCAGCGCTGCGCGCCCCAGGGCGT
CATGGAGTGAATGTGGAATCTCCTCCTTTGCCCTCGGGCCCGCCGAGTGTGGTGGGCCAGCCTGGAATGCCTGATCCAGCCCCCAGGCCTCGAGGACCAAAACTGAAGTT
TACTCCCGAAGAGGACGCTCTACTGGTGGAGTTGAAGGAAAACAAGAACTTGACGTGGAAGCAAATTGCAGACTTCTTCCCGGGCCGAACGAGCGGTACCTTGCAAGTC
CGATACTGCACCAAGCTGAAGGCTAAGGATGTAGCTTGGAGTGACGAAATGGTTCGATTTGCTCCTGATGTATTTCTACGCCTGTCTCACACATGCTAATGAAGGGAATA
GGTACAAAGGCTGCAGCGGGCAATGCACGAGTACGAGAACGATCGGTGGCGCATCATTGCAGGGAAGGTTGGAAATGGCTTCACCCCAGCTGCTTGCCGCGAGAAAGCC
ATGCAGCTCCATGAGTAAAAGCGTTGGGGAATTTTCATATTTATATCTACTGTCGCCAGATTCGGCCCTGCTTGGACCCTCTGATCTCCTTACTCTCCATATTGGTTCAAAT
GTCGGGTCACCGATAGGGCTGGTGGTGCAGGCTTGTTGTAGGCACGGGAGGATGATCAGCATAACTCTGAGTCACTATAGGGACGGGTTGATGTAAGGTATTAAGTGATG
TATGATAATTCATTTTAGCCCGGGGGAACATATGGCGCCGGCATTTGTTCGTTCGCAATGAACCGACACTAGCGTCCGCTCTCGCAGTTTAGCACCGGCTGATCCCGGGCT
GAACGCGGCCATTGCTCGGCCGGGGCATGTGTTCCTTATCTACGGCAGACCGCAGATGACCACTGGAGCAGATTATAGACCCTAAGCCCTAAGCCGGACACCCAATCGAG
TAGGTCTGCGGACCAGGTCACTGCGGGCAGCCGGAGAAGCTCCGCAACCAATCAATCCCCGGCGCTGACTAAGGGCAGGCGACCACGGGCCGAAGCGGCTTCAAACTCA
CCTCAACCTCCAAACTCCCTCATCTCCAAACGTCCTTGCCTTGTCTGCCGTCATTGCAACCCACCCACCAGGACACATGAGCAGCAAGTCCCAGCTCACCTACACTGCCCG
TGCCAGCAAGCATCCCAATGCTCTGGCGAAGAGGCTGTTCGAGATTGCCGAGGCCAAGAAaACCAATGTGACTGTCTCGGCTGACGTTACCACCACTAAGGAGCTACTAG
ATCTTGCTGACCGTCTCGGTCCCTACATTGCCGTGATCAAAACCCACATCGATATCCTCTCTGATTTCAGCAACGAGACtATTGAGGGACTTAAGGCTCTCGCGCAGAAGC
ACAACTTTCTCATCTTCGAGGACCGCAAGTTCATTGACATCGGCAACACGGTCCAGAAGCAATACCACGGCGGTACCCTCCGTATCTCGGAATGGGCCCACATCATCAAC
TGCAGCATTCTCCCTGGTGAGGGTATCGTCGAGGCTCTCGCTCAGACGGCGTCTGCACCGGACTTCGCCTACGGCCCCGAACGCGGTCTGTTGATCTTGGCAGAGATGACC
TCTAAGGGCTCCTTGGCTACCGGCCAGTACACTACTTCCTCGGTCGATTATGCCCGGAAATACAAGAACTTCGTTATGGGATTCGTGTCGACGCGCGCGTTGGGTGAGGTG
CAGTCGGAAGTCAGCTCTCCTTCGGATGAGGAGGACTTTGTGGTtTTCACGACTGGTGTGAACATTAACCTCCA

>pSF516_IC
TGGAGGaAGGGATCCGCCAAGGATGCTCTGGATCCGCATCCGGCCGCTCTTGCGCCCCATCAATCGCCCGACTATAAATCGAACTACTTTCGGCATCTTCTAGACTTCCTAATA
CCGCCTAGTCATAGCAGATTCAAGCTGAGAACACCACAAGTAAATATCACCCATCATGCTTACCCTGACCGTCCCTGAAAACTACGGGTATGTGCCAATTCTACAATTCCTTG
CAGACAATGCCATTCTCCCCATGAAGTCTGATGCTAACTATCCTGCAGCTCTGTCATTGCCGTCGCTCTGGGTGCCATCCCCGTCCTGAGCTTCGTCCATGGCGCCGTCGTGT
CTCGTCTCCGCAAGGAAGCTGATTGCCCCTACCCTCACTGCTATGCGACCGTAGAGCAGTGCAAGACCAACGTAAGCCAACCTCACACAAACAGGATTCCTCGAGCTAACA
TACATTCCGAACCGTGCAGCCCAAGGCCGAGCAGTTCAACTGCGCTCAGCGCGCTCATGCCAACTTCCTTGAGAACTCCAGCCAAACTATGCTCTTCCTCCTGGTAGCTGGA
CTGAAGTACCCCCAGTTGGCGACTGGCCTCGGAAGCATCTGGGTCCTCGGTCGCTCACTGTTCCTTTACGGATATGTGTACTCCGGCAAGCCGCGGGGTCGCGGTCGTTTGT
ACGGCAGCTTCTACTTGCTTGCACAGGGAGCTCTCTGGGGCTTGACGTCTTTTGGAGTTGCGAGGGAGTTGATTTCCTACTTCTAAGTTTGGACTGAATCCGTGGTGTGATT
GAGGTGATTGGCGATGTTTGGCTATACCAGCTATATGTAATAATCTCTACTGTATACTACTATTCAACGCATTTTACTATGCGTGCTGCTAGGGTCGGCAATGACAATGGCAATC
TGACTGACGTGGTCTATTTCTCCATGTGCAGCAGGGAATACGAGCTCCAATGGACCTCGGGAGTGGCACAGTCAATGGCAAGGAAACTGGAGcgcaaaaaaccccgcccctgacagggcg
gggtttttcgcGATCGGAGTCGATAAGCTTCGGAGAATATGGAGCTTCATCGAATCACCGGCAGTAAGCGAAGGAGAATGTGAAGCCAGGGGTGTATAGCCGTCGGCGAAATAGC
ATGCCATTAACCTAGGTACAGAAGTCCAATTGCTTCCGATCTGGTAAAAGATTCACGAGATAGTACCTTCTCCGAAGTAGGTAGAGCGAGTACCCGGCGCGTAAGCTCCCTA
ATTGGCCCATCCGGCATCTGTAGGGCGTCCAAATATCGTGCCTCTCCTGCTTTGCCCGGTGTATGAAACCGGAAAGGCCGCTCAGGAGCTGGCCAGCGGCGCAGACCGGGA
ACACAAGCTGGCAGTCGACCCATCCGGTGCTCTGCACTCGACCTGCTGAGGTCCCTCAGTCCCTGGTAGGCAGCTTTGCCCCGTCTGTCCGCCCGGTGTGTCGGCGGGGTT
GACAAGGTCGTTGCGTCAGTCCAACATTTGTTGCCATATTTCCTGCTCTCCCCACCAGCTGCTCTTTTCTTTTCTCTTTCTTTTCCCATCTTCAGTATATTCATCTTCCCATCCA
AGAACCTTTATTTCCCCTAAGTAAGTACTTTGCTACATCCATACTCCATCCTTCCCATCCCTTATTCCTTTGAACCTTTCAGTTCGAGCTTTCCCACTTCATCGCAGCTTGACTA
ACAGCTACCCCGCTTGAGCAGACATCACCGAATTCACCATGTCTAGACTGGACAAGAGCAAAGTCATAAACGGCGCTCTGGAATTACTCAATGGAGTCGGTATCGAAGGCC
TGACGACAAGGAAACTCGCTCAAAAGCTGGGAGTTGAGCAGCCTACCCTGTACTGGCACGTGAAGAACAAGCGGGCCCTGCTCGATGCCCTGCCAATCGAGATGCTGGAC
AGGCATCATACCCACTTCTGCCCCCTGGAAGGCGAGTCATGGCAAGACTTTCTGCGGAACAACGCCAAGTCATTCCGCTGTGCTCTCCTCTCACATCGCGACGGGGCTAAA
GTGCATCTCGGCACCCGCCCAACAGAGAAACAGTACGAAACCCTGGAAAATCAGCTCGCGTTCCTGTGTCAGCAAGGCTTCTCCCTGGAGAACGCACTGTACGCTCTGTCC
GCCGTGGGCCACTTTACACTGGGCTGCGTATTGGAGGAACAGGAGCATCAAGTAGCAAAAGAGGAAAGAGAGACACCTACCACCGATTCTATGCCCCCACTTCTGAGACA
AGCAATTGAGCTGTTCGACCGGCAGGGAGCCGAACCTGCCTTCCTTTTCGGCCTGGAACTAATCATATGTGGCCTGGAGAAACAGCTAAAGTGCGAAAGCGGCGGGCCGGC
CGACGCCCTTGACGATTTTGACTTAGACATGCTCCCAGCCGATGCCCTTGACGACTTTGACCTTGATATGCTGCCTGCTGACGCTCTTGACGATTTTGACCTTGACATGCTCC
CCGGGTAACTAAGTAAGGATCCACTAGTACAGCAGAAGAATCTCTCTCCGCTGTTGCTTCAGTGTCTGCCATGCATTAACTTCATCCTACTGTCCTACCCGCAGTACCCATTC
ACATTTGCGGCAGATACCAGGTTGTTTTCTATTCCCTCGGTTCTTCAGTTCTTCAGATATTACATTACTCGAGAAATTGGCGGCGATGCAGGAGTTTGGTTGGTTTTAATTGTTT
CAGTCTCCTTGACCTGATTTGTATGACATGCAATGTTTCGACGGATGACTATCTCGGTGAATATACGTCATGAATCATGCGGCCGCGCGTATCACGAGGCCCTTTCGACTTCAC
TCGAGTTTACCACTCCCTATCAGTGATAGAGAAAAGTGAAAGTCGAGTTTACCACTCCCTATCAGTGATAGAGAAAAGTGAAAGTCGAGTTTACCACTCCCTATCAGTGATA
GAGAAAAGTGAAAGTCGAGTTTACCACTCCCTATCAGTGATAGAGAAAAGTGAAAGTCGAGTTTACCACTCCCTATCAGTGATAGAGAAAAGTGAAAGTCGAGTTTACCAC
TCCCTATCAGTGATAGAGAAAAGTGAAAGTCGAGTTTACCACTCCCTATCAGTGATAGAGAAAAGTGAAAGTCGAGCTCCCCATCTTCAGTATATTCATCTTCCCATCCAAGA
ACCTTTATTTCCCCTAAGTAAGTACTTTGCTACATCCATACTCCATCCTTCCCATCCCTTATTCCTTTGAACCTTTCAGTTCGAGCTTTCCCACTTCATCGCAGCTTGACTAACA
GCTACCCCGCTTGAGCAGACATCACCGTTTAAACACCATGCCTCCCAAAGCCGCTGAGAAGAAGCCCAGCACCGGTGGCAAGGCCCCGGCAGGCGGCAAGGCTCCCGCTG
AGAAGAAGGAAGCCGGCAAGAAaACTGCTGCCGCTGCCTCCGGCGACAAGAAGAAGCGTGGAAAGACCAGGAAGGAGACtTACTCGTCTTACATCTACAAGGTCTTGAAG
CAGGTCCACCCTGATACCGGTATCTCGACTCGTGCTATGTCCATCCTGAACTCCTTTGTCAATGATATCTTCGAGCGTGTCGCGACCGAGGCCTCGAAACTTGCCGCTTACAA
CAAGAAGTCCACCATCTCTTCACGGGAGATCCAGACCTCTGTGAGGCTCATCCTTCCCGGTGAATTGGCGAAGCACGCGGTGTCGGAAGGCACCAAGGCTGTCACGAAGTA
CTCGTCGTCTGCCAAAATGGCCTCCTCCGAGGACGTCATCAAGGAGTTCATGCGCTTCAAGGTGCGCATGGAGGGCTCCGTGAACGGCCACGAGTTCGAGATCGAGGGCG
AGGGCGAGGGCCGCCCCTACGAGGGCACCCAGACCGCCAAGCTGAAGGTGACCAAGGGCGGCCCCCTGCCCTTCGCCTGGGACATCCTGTCCCCCCAGTTCCAGTACGGC
TCCAAGGTGTACGTGAAGCACCCCGCCGACATCCCCGACTACAAGAAGCTGTCCTTCCCCGAGGGCTTCAAGTGGGAGCGCGTGATGAACTTCGAGGACGGCGGCGTGGT
GACCGTGACCCAGGACTCCTCCCTGCAGGACGGCTCCTTCATCTACAAGGTGAAGTTCATCGGCGTGAACTTCCCCTCCGACGGCCCCGTAATGCAGAAGAAAACTATGGG
CTGGGAGGCCTCCACCGAGCGCCTGTACCCCCGCGACGGCGTGCTGAAGGGCGAGATCCACAAGGCCCTGAAGCTGAAGGACGGCGGCCACTACCTGGTGGAGTTCAAG
TCCATCTACATGGCCAAGAAGCCCGTGCAGCTGCCCGGCTACTACTACGTGGACTCCAAGCTGGACATCACCTCCCACAACGAGGACTACACCATCGTGGAGCAGTACGAG
CGCGCCGAGGGCCGCCACCACCTGTTCCTGTAGGCTTGATCCACTTAACGTTACTGAAATCATCAAACAGCTTGACGAATCTGGATATAAGATCGTTGGTGTCGATGTCAGCT
CCGGAGTTGAGACAAATGGTGTTCAGGATCTCGATAAGATACGTTCATTTGTCCAAGCAGCAAAGAGTGCCTTCTAGTGATTTAATAGCTCCATGTCAACAAGAATAAAACG
CGTTTCGGGTTTACCTCTTCCAGATACAGCTCATCTGCAATGCATTAATGCATTGGACCTCGCAACCCTAGTACGCCCTTCAGGCTCCGGCGAAGCAGAAGAATAGCTTAGCA
GAGTCTATTTTCATTTTCGGGAGACGAGATCAAGCAGATCAACGGTCGTCAAGAGTCCTACGAGACTGAGGAATCCGCTCTTGGCTCCACGCGACTATATATTTGTCTCTAAT
TGTACTTTGACATGCTCCTCTTCTTTACTCTGATAGCTTGACTATGAAAATTCCGTCACCAGCCCCTGGGTTCGCAAAGATAATTGCACTGTTTCTTCCTTGAACTCTCAAGCC
TACAGGACACACATTCATCGTAGGTATAAACCTCGAAAATCATTCCTACTAAGATGGGTATACAATAGTAACCATGCATGGTTGCCTAGTGAATGCTCCGTAACACCCAATACG
CCGGCCGAAACTTTTTTACAACTCTCCTATGAGTCGTTTACCCAGAATGCACAGGTACACTTGTTTAGAGGTAATCCTTCTTTCTAGAAGTCCTCGTGTACTGTGTAAGCGCC
CACTCCACATCTCCACTCGCTCCGGGGAGACGGGCACAAGGTTCATGAACCGAAGAGGATGGGAACCCCGACCATGGCCTGCGGCCGCAGCTATCAGTCCTCTCAGCTGAC
GCCCCTCACGAACCCTCGCACTCTCCCGAAATGCTCTTCGGTGTCCATGGCGCATCATCTCAGCAGCATCCCATGTCAAACCATGGCTTTGGGCCCACGGACTCTGTGGCCC
TGCCGCAACATCACCATCACCATCGACTCCCGCCCCATGCAGCGCTGCGCGCCCCAGGGCGTCATGGAGTGAATGTGGAATCTCCTCCTTTGCCCTCGGGCCCGCCGAGTGT
GGTGGGCCAGCCTGGAATGCCTGATCCAGCCCCCAGGCCTCGAGGACCAAAACTGAAGTTTACTCCCGAAGAGGACGCTCTACTGGTGGAGTTGAAGGAAAACAAGAACT
TGACGTGGAAGCAAATTGCAGACTTCTTCCCGGGCCGAACGAGCGGTACCTTGCAAGTCCGATACTGCACCAAGCTGAAGGCTAAGGATGTAGCTTGGAGTGACGAAATG
GTTCGATTTGCTCCTGATGTATTTCTACGCCTGTCTCACACATGCTAATGAAGGGAATAGGTACAAAGGCTGCAGCGGGCAATGCACGAGTACGAGAACGATCGGTGGCGCA
TCATTGCAGGGAAGGTTGGAAATGGCTTCACCCCAGCTGCTTGCCGCGAGAAAGCCATGCAGCTCCATGAGTAAAAGCGTTGGGGAATTTTCATATTTATATCTACTGTCGCC
AGATTCGGCCCTGCTTGGACCCTCTGATCTCCTTACTCTCCATATTGGTTCAAATGTCGGGTCACCGATAGGGCTGGTGGTGCAGGCTTGTTGTAGGCACGGGAGGATGATCA
GCATAACTCTGAGTCACTATAGGGACGGGTTGATGTAAGGTATTAAGTGATGTATGATAATTCATTTTAGCCCGGGGGAACATATGGCGCCGGCATTTGTTCGTTCGCAATGAA
CCGACACTAGCGTCCGCTCTCGCAGTTTAGCACCGGCTGATCCCGGGCTGAACGCGGCCATTGCTCGGCCGGGGCATGTGTTCCTTATCTACGGCAGACCGCAGATGACCAC
TGGAGCAGATTATAGACCCTAAGCCCTAAGCCGGACACCCAATCGAGTAGGTCTGCGGACCAGGTCACTGCGGGCAGCCGGAGAAGCTCCGCAACCAATCAATCCCCGGCG
CTGACTAAGGGCAGGCGACCACGGGCCGAAGCGGCTTCAAACTCACCTCAACCTCCAAACTCCCTCATCTCCAAACGTCCTTGCCTTGTCTGCCGTCATTGCAACCCACCC
ACCAGGACACATGAGCAGCAAGTCCCAGCTCACCTACACTGCCCGTGCCAGCAAGCATCCCAATGCTCTGGCGAAGAGGCTGTTCGAGATTGCCGAGGCCAAGAAaACCA
ATGTGACTGTCTCGGCTGACGTTACCACCACTAAGGAGCTACTAGATCTTGCTGACCGTCTCGGTCCCTACATTGCCGTGATCAAAACCCACATCGATATCCTCTCTGATTTCA
GCAACGAGACtATTGAGGGACTTAAGGCTCTCGCGCAGAAGCACAACTTTCTCATCTTCGAGGACCGCAAGTTCATTGACATCGGCAACACGGTCCAGAAGCAATACCACG
GCGGTACCCTCCGTATCTCGGAATGGGCCCACATCATCAACTGCAGCATTCTCCCTGGTGAGGGTATCGTCGAGGCTCTCGCTCAGACGGCGTCTGCACCGGACTTCGCCTA
CGGCCCCGAACGCGGTCTGTTGATCTTGGCAGAGATGACCTCTAAGGGCTCCTTGGCTACCGGCCAGTACACTACTTCCTCGGTCGATTATGCCCGGAAATACAAGAACTTC
GTTATGGGATTCGTGTCGACGCGCGCGTTGGGTGAGGTGCAGTCGGAAGTCAGCTCTCCTTCGGATGAGGAGGACTTTGTGGTtTTCACGACTGGTGTGAACATTAACCTCC
A

>pSF515_IC

TGGAGGaAGGGATCCGCCAAGGATGCTCTGGATCCGCATCCGGCCGCTCTTGCGCCCCATCAATCGCCCGACTATAAATCGAACTACTTTCGGCATCTTCTAGACTTCCTA
ATACCGCCTAGTCATAGCAGATTCAAGCTGAGAACACCACAAGTAAATATCACCCATCATGCTTACCCTGACCGTCCCTGAAAACTACGGGTATGTGCCAATTCTACAATT
CCTTGCAGACAATGCCATTCTCCCCATGAAGTCTGATGCTAACTATCCTGCAGCTCTGTCATTGCCGTCGCTCTGGGTGCCATCCCCGTCCTGAGCTTCGTCCATGGCGCCG
TCGTGTCTCGTCTCCGCAAGGAAGCTGATTGCCCCTACCCTCACTGCTATGCGACCGTAGAGCAGTGCAAGACCAACGTAAGCCAACCTCACACAAACAGGATTCCTCGA
GCTAACATACATTCCGAACCGTGCAGCCCAAGGCCGAGCAGTTCAACTGCGCTCAGCGCGCTCATGCCAACTTCCTTGAGAACTCCAGCCAAACTATGCTCTTCCTCCTGG
TAGCTGGACTGAAGTACCCCCAGTTGGCGACTGGCCTCGGAAGCATCTGGGTCCTCGGTCGCTCACTGTTCCTTTACGGATATGTGTACTCCGGCAAGCCGCGGGGTCGCG
GTCGTTTGTACGGCAGCTTCTACTTGCTTGCACAGGGAGCTCTCTGGGGCTTGACGTCTTTTGGAGTTGCGAGGGAGTTGATTTCCTACTTCTAAGTTTGGACTGAATCCGT
GGTGTGATTGAGGTGATTGGCGATGTTTGGCTATACCAGCTATATGTAATAATCTCTACTGTATACTACTATTCAACGCATTTTACTATGCGTGCTGCTAGGGTCGGCAATG
ACAATGGCAATCTGACTGACGTGGTCTATTTCTCCATGTGCAGCAGGGAATACGAGCTCCAATGGACCTCGGGAGTGGCACAGTCAATGGCAAGGAAACTGGAGcgcaaaaaa
ccccgcccctgacagggcggggtttttcgcGATCGGAGTCGATAAGCTTCGGAGAATATGGAGCTTCATCGAATCACCGGCAGTAAGCGAAGGAGAATGTGAAGCCAGGGGTGTATAGCC
GTCGGCGAAATAGCATGCCATTAACCTAGGTACAGAAGTCCAATTGCTTCCGATCTGGTAAAAGATTCACGAGATAGTACCTTCTCCGAAGTAGGTAGAGCGAGTACCCG
GCGCGTAAGCTCCCTAATTGGCCCATCCGGCATCTGTAGGGCGTCCAAATATCGTGCCTCTCCTGCTTTGCCCGGTGTATGAAACCGGAAAGGCCGCTCAGGAGCTGGCC
AGCGGCGCAGACCGGGAACACAAGCTGGCAGTCGACCCATCCGGTGCTCTGCACTCGACCTGCTGAGGTCCCTCAGTCCCTGGTAGGCAGCTTTGCCCCGTCTGTCCGCC
CGGTGTGTCGGCGGGGTTGACAAGGTCGTTGCGTCAGTCCAACATTTGTTGCCATATTTTCCTGCTCTCCCCACCAGCTGCTCTTTTCTTTTCTCTTTCTTTTCCCATCTTCA
GTATATTCATCTTCCCATCCAAGAACCTTTATTTCCCCTAAGTAAGTACTTTGCTACATCCATACTCCATCCTTCCCATCCCTTATTCCTTTGAACCTTTCAGTTCGAGCTTTC
CCACTTCATCGCAGCTTGACTAACAGCTACCCCGCTTGAGCAGACATCACCGAATTCACCATGTCTAGACTGGACAAGAGCAAAGTCATAAACGGCGCTCTGGAATTACT
CAATGGAGTCGGTATCGAAGGCCTGACGACAAGGAAACTCGCTCAAAAGCTGGGAGTTGAGCAGCCTACCCTGTACTGGCACGTGAAGAACAAGCGGGCCCTGCTCGAT
GCCCTGCCAATCGAGATGCTGGACAGGCATCATACCCACTTCTGCCCCCTGGAAGGCGAGTCATGGCAAGACTTTCTGCGGAACAACGCCAAGTCATTCCGCTGTGCTCT
CCTCTCACATCGCGACGGGGCTAAAGTGCATCTCGGCACCCGCCCAACAGAGAAACAGTACGAAACCCTGGAAAATCAGCTCGCGTTCCTGTGTCAGCAAGGCTTCTCCC
TGGAGAACGCACTGTACGCTCTGTCCGCCGTGGGCCACTTTACACTGGGCTGCGTATTGGAGGAACAGGAGCATCAAGTAGCAAAAGAGGAAAGAGAGACACCTACCAC
CGATTCTATGCCCCCACTTCTGAGACAAGCAATTGAGCTGTTCGACCGGCAGGGAGCCGAACCTGCCTTCCTTTTCGGCCTGGAACTAATCATATGTGGCCTGGAGAAACA
GCTAAAGTGCGAAAGCGGCGGGCCGGCCGACGCCCTTGACGATTTTGACTTAGACATGCTCCCAGCCGATGCCCTTGACGACTTTGACCTTGATATGCTGCCTGCTGACGC
TCTTGACGATTTTGACCTTGACATGCTCCCCGGGTAACTAAGTAAGGATCCACTAGTACAGCAGAAGAATCTCTCTCCGCTGTTGCTTCAGTGTCTGCCATGCATTAACTTC
ATCCTACTGTCCTACCCGCAGTACCCATTCACATTTGCGGCAGATACCAGGTTGTTTTCTATTCCCTCGGTTCTTCAGTTCTTCAGATATTACATTACTCGAGAAATTGGCG
GCGATGCAGGAGTTTGGTTGGTTTTAATTGTTTCAGTCTCCTTGACCTGATTTGTATGACATGCAATGTTTCGACGGATGACTATCTCGGTGAATATACGTCATGAATCATG
CGGCCGCGCGTATCACGAGGCCCTTTCGACTTCACTCGAGTTTACCACTCCCTATCAGTGATAGAGAAAAGTGAAAGTCGAGTTTACCACTCCCTATCAGTGATAGAGAA
AAGTGAAAGTCGAGTTTACCACTCCCTATCAGTGATAGAGAAAAGTGAAAGTCGAGTTTACCACTCCCTATCAGTGATAGAGAAAAGTGAAAGTCGAGTTTACCACTCCC
TATCAGTGATAGAGAAAAGTGAAAGTCGAGTTTACCACTCCCTATCAGTGATAGAGAAAAGTGAAAGTCGAGTTTACCACTCCCTATCAGTGATAGAGAAAAGTGAAAG
TCGAGCTCCCCATCTTCAGTATATTCATCTTCCCATCCAAGAACCTTTATTTCCCCTAAGTAAGTACTTTGCTACATCCATACTCCATCCTTCCCATCCCTTATTCCTTTGAA
CCTTTCAGTTCGAGCTTTCCCACTTCATCGCAGCTTGACTAACAGCTACCCCGCTTGAGCAGACATCACCGTTTAAACACCATGACTGGAGGCAAATCTGGAGGCAAGGCC
AGCGGCAGCAAGAACGCCCAATCCCGTTCGTCCAAGGCCGGcCTCGCCTTTCCCGTTGGACGTGTTCATCGTCTCCTCCGCAAGGGCAACTACGCTCAGCGTGTTGGTGCC
GGTGCTCCCGTTTACCTCGCTGCCGTTCTTGAGTATCTCGCCGCCGAAATTCTTGAATTGGCTGGCAACGCTGCTCGCGATAACAAaAAGACCCGTATCATTCCCCGTCACC
TTCAGCTCGCCATCCGCAACGATGAGGAACTGAACAAGCTCCTGGGTCATGTCACCATTGCCCAGGGTGGTGTCCTCCCCAACATCCACCAGAACCTTCTGCCCAAaAAGA
CCCCCAAGAGTGGAAAGGGCCCGGGAAGCCAGGAGCTGATGGTGAGCAAGGGCGAGGAGCTGTTCACCGGGGTGGTGCCCATCCTGGTCGAGCTGGACGGCGACGTAA
ACGGCCACAAGTTCAGCGTGCGCGGCGAGGGCGAGGGCGATGCCACCAACGGCAAGCTGACCCTGAAGTTCATCTGCACCACCGGCAAGCTGCCCGTGCCCTGGCCCAC
CCTCGTGACCACCCTGACCTACGGCGTGCAGTGCTTCAGCCGCTACCCCGACCACATGAAGCAGCACGACTTCTTCAAGTCCGCCATGCCCGAAGGCTACGTCCAGGAGC
GCACCATCACCTTCAAGGACGACGGCACCTACAAGACCCGCGCCGAGGTGAAGTTCGAGGGCGACACCCTGGTGAACCGCATCGAGCTGAAGGGCATCGACTTCAAGGA
GGACGGCAACATCCTGGGGCACAAGCTGGAGTACAACTTCAACAGCCACAACGTCTATATCACCGCCGACAAGCAGAAGAACGGCATCAAGGCCAACTTCAAGATCCGC
CACAACGTGGAGGACGGCAGCGTGCAGCTCGCCGACCACTACCAGCAGAACACCCCCATCGGCGACGGCCCCGTGCTGCTGCCCGACAACCACTACCTGAGCACCCAGT
CCAAGCTGAGCAAAGACCCCAACGAGAAGCGCGATCACATGGTCCTGCTGGAGTTCGTGACCGCCGCCGGGATCACTCACGGCATGGACGAGCTGTACAAGTAAGCTTG
ATCCACTTAACGTTACTGAAATCATCAAACAGCTTGACGAATCTGGATATAAGATCGTTGGTGTCGATGTCAGCTCCGGAGTTGAGACAAATGGTGTTCAGGATCTCGAT
AAGATACGTTCATTTGTCCAAGCAGCAAAGAGTGCCTTCTAGTGATTTAATAGCTCCATGTCAACAAGAATAAAACGCGTTTCGGGTTTACCTCTTCCAGATACAGCTCAT
CTGCAATGCATTAATGCATTGGACCTCGCAACCCTAGTACGCCCTTCAGGCTCCGGCGAAGCAGAAGAATAGCTTAGCAGAGTCTATTTTCATTTTCGGGAGACGAGATC
AAGCAGATCAACGGTCGTCAAGAGTCCTACGAGACTGAGGAATCCGCTCTTGGCTCCACGCGACTATATATTTGTCTCTAATTGTACTTTGACATGCTCCTCTTCTTTACTC
TGATAGCTTGACTATGAAAATTCCGTCACCAGCCCCTGGGTTCGCAAAGATAATTGCACTGTTTCTTCCTTGAACTCTCAAGCCTACAGGACACACATTCATCGTAGGTAT
AAACCTCGAAAATCATTCCTACTAAGATGGGTATACAATAGTAACCATGCATGGTTGCCTAGTGAATGCTCCGTAACACCCAATACGCCGGCCGAAACTTTTTTACAACTC
TCCTATGAGTCGTTTACCCAGAATGCACAGGTACACTTGTTTAGAGGTAATCCTTCTTTCTAGAAGTCCTCGTGTACTGTGTAAGCGCCCACTCCACATCTCCACTCGCTCC
GGGGAGACGGGCACAAGGTTCATGAACCGAAGAGGATGGGAACCCCGACCATGGCCTGCGGCCGCAGCTATCAGTCCTCTCAGCTGACGCCCCTCACGAACCCTCGCAC
TCTCCCGAAATGCTCTTCGGTGTCCATGGCGCATCATCTCAGCAGCATCCCATGTCAAACCATGGCTTTGGGCCCACGGACTCTGTGGCCCTGCCGCAACATCACCATCAC
CATCGACTCCCGCCCCATGCAGCGCTGCGCGCCCCAGGGCGTCATGGAGTGAATGTGGAATCTCCTCCTTTGCCCTCGGGCCCGCCGAGTGTGGTGGGCCAGCCTGGAAT
GCCTGATCCAGCCCCCAGGCCTCGAGGACCAAAACTGAAGTTTACTCCCGAAGAGGACGCTCTACTGGTGGAGTTGAAGGAAAACAAGAACTTGACGTGGAAGCAAATT
GCAGACTTCTTCCCGGGCCGAACGAGCGGTACCTTGCAAGTCCGATACTGCACCAAGCTGAAGGCTAAGGATGTAGCTTGGAGTGACGAAATGGTTCGATTTGCTCCTGA
TGTATTTCTACGCCTGTCTCACACATGCTAATGAAGGGAATAGGTACAAAGGCTGCAGCGGGCAATGCACGAGTACGAGAACGATCGGTGGCGCATCATTGCAGGGAAG
GTTGGAAATGGCTTCACCCCAGCTGCTTGCCGCGAGAAAGCCATGCAGCTCCATGAGTAAAAGCGTTGGGGAATTTTCATATTTATATCTACTGTCGCCAGATTCGGCCCT
GCTTGGACCCTCTGATCTCCTTACTCTCCATATTGGTTCAAATGTCGGGTCACCGATAGGGCTGGTGGTGCAGGCTTGTTGTAGGCACGGGAGGATGATCAGCATAACTCT
GAGTCACTATAGGGACGGGTTGATGTAAGGTATTAAGTGATGTATGATAATTCATTTTAGCCCGGGGGAACATATGGCGCCGGCATTTGTTCGTTCGCAATGAACCGACA
CTAGCGTCCGCTCTCGCAGTTTAGCACCGGCTGATCCCGGGCTGAACGCGGCCATTGCTCGGCCGGGGCATGTGTTCCTTATCTACGGCAGACCGCAGATGACCACTGGA
GCAGATTATAGACCCTAAGCCCTAAGCCGGACACCCAATCGAGTAGGTCTGCGGACCAGGTCACTGCGGGCAGCCGGAGAAGCTCCGCAACCAATCAATCCCCGGCGCT
GACTAAGGGCAGGCGACCACGGGCCGAAGCGGCTTCAAACTCACCTCAACCTCCAAACTCCCTCATCTCCAAACGTCCTTGCCTTGTCTGCCGTCATTGCAACCCACCCAC
CAGGACACATGAGCAGCAAGTCCCAGCTCACCTACACTGCCCGTGCCAGCAAGCATCCCAATGCTCTGGCGAAGAGGCTGTTCGAGATTGCCGAGGCCAAGAAaACCAAT
GTGACTGTCTCGGCTGACGTTACCACCACTAAGGAGCTACTAGATCTTGCTGACCGTCTCGGTCCCTACATTGCCGTGATCAAAACCCACATCGATATCCTCTCTGATTTCA
GCAACGAGACtATTGAGGGACTTAAGGCTCTCGCGCAGAAGCACAACTTTCTCATCTTCGAGGACCGCAAGTTCATTGACATCGGCAACACGGTCCAGAAGCAATACCAC
GGCGGTACCCTCCGTATCTCGGAATGGGCCCACATCATCAACTGCAGCATTCTCCCTGGTGAGGGTATCGTCGAGGCTCTCGCTCAGACGGCGTCTGCACCGGACTTCGCC
TACGGCCCCGAACGCGGTCTGTTGATCTTGGCAGAGATGACCTCTAAGGGCTCCTTGGCTACCGGCCAGTACACTACTTCCTCGGTCGATTATGCCCGGAAATACAAGAA
CTTCGTTATGGGATTCGTGTCGACGCGCGCGTTGGGTGAGGTGCAGTCGGAAGTCAGCTCTCCTTCGGATGAGGAGGACTTTGTGGTtTTCACGACTGGTGTGAACATTAA
CCTCCA

>pSF525_IC
TGGAGGaAGGGATCCGCCAAGGATGCTCTGGATCCGCATCCGGCCGCTCTTGCGCCCCATCAATCGCCCGACTATAAATCGAACTACTTTCGGCATCTTCTAGACTTCCTA
ATACCGCCTAGTCATAGCAGATTCAAGCTGAGAACACCACAAGTAAATATCACCCATCATGCTTACCCTGACCGTCCCTGAAAACTACGGGTATGTGCCAATTCTACAATT
CCTTGCAGACAATGCCATTCTCCCCATGAAGTCTGATGCTAACTATCCTGCAGCTCTGTCATTGCCGTCGCTCTGGGTGCCATCCCCGTCCTGAGCTTCGTCCATGGCGCCG
TCGTGTCTCGTCTCCGCAAGGAAGCTGATTGCCCCTACCCTCACTGCTATGCGACCGTAGAGCAGTGCAAGACCAACGTAAGCCAACCTCACACAAACAGGATTCCTCGA
GCTAACATACATTCCGAACCGTGCAGCCCAAGGCCGAGCAGTTCAACTGCGCTCAGCGCGCTCATGCCAACTTCCTTGAGAACTCCAGCCAAACTATGCTCTTCCTCCTGG
TAGCTGGACTGAAGTACCCCCAGTTGGCGACTGGCCTCGGAAGCATCTGGGTCCTCGGTCGCTCACTGTTCCTTTACGGATATGTGTACTCCGGCAAGCCGCGGGGTCGCG
GTCGTTTGTACGGCAGCTTCTACTTGCTTGCACAGGGAGCTCTCTGGGGCTTGACGTCTTTTGGAGTTGCGAGGGAGTTGATTTCCTACTTCTAAGTTTGGACTGAATCCGT
GGTGTGATTGAGGTGATTGGCGATGTTTGGCTATACCAGCTATATGTAATAATCTCTACTGTATACTACTATTCAACGCATTTTACTATGCGTGCTGCTAGGGTCGGCAATG
ACAATGGCAATCTGACTGACGTGGTCTATTTCTCCATGTGCAGCAGGGAATACGAGCTCCAATGGACCTCGGGAGTGGCACAGTCAATGGCAAGGAAACTGGAGcgcaaaaaa
ccccgcccctgacagggcggggtttttcgcGATCGGAGTCGATAAGCTTCGGAGAATATGGAGCTTCATCGAATCACCGGCAGTAAGCGAAGGAGAATGTGAAGCCAGGGGTGTATAGCC
GTCGGCGAAATAGCATGCCATTAACCTAGGTACAGAAGTCCAATTGCTTCCGATCTGGTAAAAGATTCACGAGATAGTACCTTCTCCGAAGTAGGTAGAGCGAGTACCCG
GCGCGTAAGCTCCCTAATTGGCCCATCCGGCATCTGTAGGGCGTCCAAATATCGTGCCTCTCCTGCTTTGCCCGGTGTATGAAACCGGAAAGGCCGCTCAGGAGCTGGCC
AGCGGCGCAGACCGGGAACACAAGCTGGCAGTCGACCCATCCGGTGCTCTGCACTCGACCTGCTGAGGTCCCTCAGTCCCTGGTAGGCAGCTTTGCCCCGTCTGTCCGCC
CGGTGTGTCGGCGGGGTTGACAAGGTCGTTGCGTCAGTCCAACATTTGTTGCCATATTTTCCTGCTCTCCCCACCAGCTGCTCTTTTCTTTTCTCTTTCTTTTCCCATCTTCA
GTATATTCATCTTCCCATCCAAGAACCTTTATTTCCCCTAAGTAAGTACTTTGCTACATCCATACTCCATCCTTCCCATCCCTTATTCCTTTGAACCTTTCAGTTCGAGCTTTC
CCACTTCATCGCAGCTTGACTAACAGCTACCCCGCTTGAGCAGACATCACCGAATTCACCATGTCTAGACTGGACAAGAGCAAAGTCATAAACGGCGCTCTGGAATTACT
CAATGGAGTCGGTATCGAAGGCCTGACGACAAGGAAACTCGCTCAAAAGCTGGGAGTTGAGCAGCCTACCCTGTACTGGCACGTGAAGAACAAGCGGGCCCTGCTCGAT
GCCCTGCCAATCGAGATGCTGGACAGGCATCATACCCACTTCTGCCCCCTGGAAGGCGAGTCATGGCAAGACTTTCTGCGGAACAACGCCAAGTCATTCCGCTGTGCTCT
CCTCTCACATCGCGACGGGGCTAAAGTGCATCTCGGCACCCGCCCAACAGAGAAACAGTACGAAACCCTGGAAAATCAGCTCGCGTTCCTGTGTCAGCAAGGCTTCTCCC
TGGAGAACGCACTGTACGCTCTGTCCGCCGTGGGCCACTTTACACTGGGCTGCGTATTGGAGGAACAGGAGCATCAAGTAGCAAAAGAGGAAAGAGAGACACCTACCAC
CGATTCTATGCCCCCACTTCTGAGACAAGCAATTGAGCTGTTCGACCGGCAGGGAGCCGAACCTGCCTTCCTTTTCGGCCTGGAACTAATCATATGTGGCCTGGAGAAACA
GCTAAAGTGCGAAAGCGGCGGGCCGGCCGACGCCCTTGACGATTTTGACTTAGACATGCTCCCAGCCGATGCCCTTGACGACTTTGACCTTGATATGCTGCCTGCTGACGC
TCTTGACGATTTTGACCTTGACATGCTCCCCGGGTAACTAAGTAAGGATCCACTAGTACAGCAGAAGAATCTCTCTCCGCTGTTGCTTCAGTGTCTGCCATGCATTAACTTC
ATCCTACTGTCCTACCCGCAGTACCCATTCACATTTGCGGCAGATACCAGGTTGTTTTCTATTCCCTCGGTTCTTCAGTTCTTCAGATATTACATTACTCGAGAAATTGGCG
GCGATGCAGGAGTTTGGTTGGTTTTAATTGTTTCAGTCTCCTTGACCTGATTTGTATGACATGCAATGTTTCGACGGATGACTATCTCGGTGAATATACGTCATGAATCATG
CGGCCGCGCGTATCACGAGGCCCTTTCGACTTCACTCGAGTTTACCACTCCCTATCAGTGATAGAGAAAAGTGAAAGTCGAGTTTACCACTCCCTATCAGTGATAGAGAA
AAGTGAAAGTCGAGTTTACCACTCCCTATCAGTGATAGAGAAAAGTGAAAGTCGAGTTTACCACTCCCTATCAGTGATAGAGAAAAGTGAAAGTCGAGTTTACCACTCCC
TATCAGTGATAGAGAAAAGTGAAAGTCGAGTTTACCACTCCCTATCAGTGATAGAGAAAAGTGAAAGTCGAGTTTACCACTCCCTATCAGTGATAGAGAAAAGTGAAAG
TCGAGCTCCCCATCTTCAGTATATTCATCTTCCCATCCAAGAACCTTTATTTCCCCTAAGTAAGTACTTTGCTACATCCATACTCCATCCTTCCCATCCCTTATTCCTTTGAA
CCTTTCAGTTCGAGCTTTCCCACTTCATCGCAGCTTGACTAACAGCTACCCCGCTTGAGCAGACATCACCGTTTAAACACCATGTCGTTCCGATCTCTACTCGCCCTGAGCG
GCCTCGTCTGCACAGGGTTGGCAAATGTGATTTCCAAGCGCGCGACCCGTGCCTTCTTTGTCTTTGGTGACTCATTGGTGGATTCAGGTAATAATAACTATCTTGCTACTAC
TGCTCGTGCGGATTCCCCTCCTTACGGCATCGATTATCCCACCCGTCGCGCTACCGGTCGATTCTCCAACGGTTACAACATCCCGGATATCATCTCCCAGCAGATCGGTTCC
AGCGAGAGCCCCCTGCCTTATCTGGACCCTGCTCTGACCGGTCAGCGCCTGCTCGTCGGTGCTAACTTCGCCAGCGCTGGCATCGGTATCCTGAACGACACCGGCATCCAG
TTCATCAACATCATCCGCATGCCGCAGCAGCTCGCCTACTTCCGGCAGTACCAGAGCCGCGTGAGCGGCCTGATCGGCGAGGCCAACACCCAGCGCCTCGTCAACCAGGC
TCTGGTGCTCATGACCCTGGGCGGTAACGATTTCGTCAACAACTACTATCTGGTGCCCAACTCTGCTCGCAGCCGCCAGTTCTCCATCCAGGACTACGTCCCTTACCTCATC

CGCGAGTACCGCAAGATCCTGATGAACGTGTACAATCTGGGTGCTCGTCGCGTCATCGTGACCGGTACCGGTCCTCTGGGTTGCGTCCCTGCTGAACTGGCTCAGCGCTCC
CGCAATGGTGAATGCAGCCCTGAGCTGCAGCGAGCCGCTGGTCTGTTCAACCCCCAGCTCACCCAGATGCTGCAGGGCCTCAACTCCGAGCTCGGTAGCGATGTGTTCAT
CGCCGCTAACACCCAGCAGATGCACACCAACTTCATCACCAACCCGCAGGCCTACGGCTTCATCACCTCCAAGGTGGCTTGCTGCGGTCAGGGTCCTTACAATGGCCTGG
GCCTGTGCACCCCTCTGAGCAACCTCTGCCCTAACCGCGATGTCTACGCCTTCTGGGACCCTTTCCATCCTTCTGAGCGAGCTAACAAGATCATCGTGCAGCAGATCATGA
GCGGCACCACCGAGCTGATGAACCCCATGAATCTTTCGACAATCCTTGCGATGGACAGTCACGCCTGGAGCCACCCACAGTTTGAGAAGTAAGCTTGATCCACTTAACGT
TACTGAAATCATCAAACAGCTTGACGAATCTGGATATAAGATCGTTGGTGTCGATGTCAGCTCCGGAGTTGAGACAAATGGTGTTCAGGATCTCGATAAGATACGTTCATT
TGTCCAAGCAGCAAAGAGTGCCTTCTAGTGATTTAATAGCTCCATGTCAACAAGAATAAAACGCGTTTCGGGTTTACCTCTTCCAGATACAGCTCATCTGCAATGCATTAA
TGCATTGGACCTCGCAACCCTAGTACGCCCTTCAGGCTCCGGCGAAGCAGAAGAATAGCTTAGCAGAGTCTATTTTCATTTTCGGGAGACGAGATCAAGCAGATCAACGG
TCGTCAAGAGTCCTACGAGACTGAGGAATCCGCTCTTGGCTCCACGCGACTATATATTTGTCTCTAATTGTACTTTGACATGCTCCTCTTCTTTACTCTGATAGCTTGACTA
TGAAAATTCCGTCACCAGCCCCTGGGTTCGCAAAGATAATTGCACTGTTTCTTCCTTGAACTCTCAAGCCTACAGGACACACATTCATCGTAGGTATAAACCTCGAAAATC
ATTCCTACTAAGATGGGTATACAATAGTAACCATGCATGGTTGCCTAGTGAATGCTCCGTAACACCCAATACGCCGGCCGAAACTTTTTTACAACTCTCCTATGAGTCGTT
TACCCAGAATGCACAGGTACACTTGTTTAGAGGTAATCCTTCTTTCTAGAAGTCCTCGTGTACTGTGTAAGCGCCCACTCCACATCTCCACTCGCTCCGGGGAGACGGGCA
CAAGGTTCATGAACCGAAGAGGATGGGAACCCCGACCATGGCCTGCGGCCGCAGCTATCAGTCCTCTCAGCTGACGCCCCTCACGAACCCTCGCACTCTCCCGAAATGCT
CTTCGGTGTCCATGGCGCATCATCTCAGCAGCATCCCATGTCAAACCATGGCTTTGGGCCCACGGACTCTGTGGCCCTGCCGCAACATCACCATCACCATCGACTCCCGCC
CCATGCAGCGCTGCGCGCCCCAGGGCGTCATGGAGTGAATGTGGAATCTCCTCCTTTGCCCTCGGGCCCGCCGAGTGTGGTGGGCCAGCCTGGAATGCCTGATCCAGCCC
CCAGGCCTCGAGGACCAAAACTGAAGTTTACTCCCGAAGAGGACGCTCTACTGGTGGAGTTGAAGGAAAACAAGAACTTGACGTGGAAGCAAATTGCAGACTTCTTCCC
GGGCCGAACGAGCGGTACCTTGCAAGTCCGATACTGCACCAAGCTGAAGGCTAAGGATGTAGCTTGGAGTGACGAAATGGTTCGATTTGCTCCTGATGTATTTCTACGCC
TGTCTCACACATGCTAATGAAGGGAATAGGTACAAAGGCTGCAGCGGGCAATGCACGAGTACGAGAACGATCGGTGGCGCATCATTGCAGGGAAGGTTGGAAATGGCTT
CACCCCAGCTGCTTGCCGCGAGAAAGCCATGCAGCTCCATGAGTAAAAGCGTTGGGGAATTTTCATATTTATATCTACTGTCGCCAGATTCGGCCCTGCTTGGACCCTCTG
ATCTCCTTACTCTCCATATTGGTTCAAATGTCGGGTCACCGATAGGGCTGGTGGTGCAGGCTTGTTGTAGGCACGGGAGGATGATCAGCATAACTCTGAGTCACTATAGGG
ACGGGTTGATGTAAGGTATTAAGTGATGTATGATAATTCATTTTAGCCCGGGGGAACATATGGCGCCGGCATTTGTTCGTTCGCAATGAACCGACACTAGCGTCCGCTCTC
GCAGTTTAGCACCGGCTGATCCCGGGCTGAACGCGGCCATTGCTCGGCCGGGGCATGTGTTCCTTATCTACGGCAGACCGCAGATGACCACTGGAGCAGATTATAGACCC
TAAGCCCTAAGCCGGACACCCAATCGAGTAGGTCTGCGGACCAGGTCACTGCGGGCAGCCGGAGAAGCTCCGCAACCAATCAATCCCCGGCGCTGACTAAGGGCAGGCG
ACCACGGGCCGAAGCGGCTTCAAACTCACCTCAACCTCCAAACTCCCTCATCTCCAAACGTCCTTGCCTTGTCTGCCGTCATTGCAACCCACCCACCAGGACACATGAGCA
GCAAGTCCCAGCTCACCTACACTGCCCGTGCCAGCAAGCATCCCAATGCTCTGGCGAAGAGGCTGTTCGAGATTGCCGAGGCCAAGAAaACCAATGTGACTGTCTCGGCT
GACGTTACCACCACTAAGGAGCTACTAGATCTTGCTGACCGTCTCGGTCCCTACATTGCCGTGATCAAAACCCACATCGATATCCTCTCTGATTTCAGCAACGAGACtATTG
AGGGACTTAAGGCTCTCGCGCAGAAGCACAACTTTCTCATCTTCGAGGACCGCAAGTTCATTGACATCGGCAACACGGTCCAGAAGCAATACCACGGCGGTACCCTCCGT
ATCTCGGAATGGGCCCACATCATCAACTGCAGCATTCTCCCTGGTGAGGGTATCGTCGAGGCTCTCGCTCAGACGGCGTCTGCACCGGACTTCGCCTACGGCCCCGAACG
CGGTCTGTTGATCTTGGCAGAGATGACCTCTAAGGGCTCCTTGGCTACCGGCCAGTACACTACTTCCTCGGTCGATTATGCCCGGAAATACAAGAACTTCGTTATGGGATT
CGTGTCGACGCGCGCGTTGGGTGAGGTGCAGTCGGAAGTCAGCTCTCCTTCGGATGAGGAGGACTTTGTGGTtTTCACGACTGGTGTGAACATTAACCTCCA

>pSF529_IC
TGGAGGaAGGGATCCGCCAAGGATGCTCTGGATCCGCATCCGGCCGCTCTTGCGCCCCATCAATCGCCCGACTATAAATCGAACTACTTTCGGCATCTTCTAGACTTCCTA
ATACCGCCTAGTCATAGCAGATTCAAGCTGAGAACACCACAAGTAAATATCACCCATCATGCTTACCCTGACCGTCCCTGAAAACTACGGGTATGTGCCAATTCTACAATT
CCTTGCAGACAATGCCATTCTCCCCATGAAGTCTGATGCTAACTATCCTGCAGCTCTGTCATTGCCGTCGCTCTGGGTGCCATCCCCGTCCTGAGCTTCGTCCATGGCGCCG
TCGTGTCTCGTCTCCGCAAGGAAGCTGATTGCCCCTACCCTCACTGCTATGCGACCGTAGAGCAGTGCAAGACCAACGTAAGCCAACCTCACACAAACAGGATTCCTCGA
GCTAACATACATTCCGAACCGTGCAGCCCAAGGCCGAGCAGTTCAACTGCGCTCAGCGCGCTCATGCCAACTTCCTTGAGAACTCCAGCCAAACTATGCTCTTCCTCCTGG
TAGCTGGACTGAAGTACCCCCAGTTGGCGACTGGCCTCGGAAGCATCTGGGTCCTCGGTCGCTCACTGTTCCTTTACGGATATGTGTACTCCGGCAAGCCGCGGGGTCGCG
GTCGTTTGTACGGCAGCTTCTACTTGCTTGCACAGGGAGCTCTCTGGGGCTTGACGTCTTTTGGAGTTGCGAGGGAGTTGATTTCCTACTTCTAAGTTTGGACTGAATCCGT
GGTGTGATTGAGGTGATTGGCGATGTTTGGCTATACCAGCTATATGTAATAATCTCTACTGTATACTACTATTCAACGCATTTTACTATGCGTGCTGCTAGGGTCGGCAATG
ACAATGGCAATCTGACTGACGTGGTCTATTTCTCCATGTGCAGCAGGGAATACGAGCTCCAATGGACCTCGGGAGTGGCACAGTCAATGGCAAGGAAACTGGAGcgcaaaaaa
ccccgcccctgacagggcggggtttttcgcGATCGGAGCCTCTCGTATGCAGAGGAAATCTCCCCTGATCTTCCGAACTGGTCGTACCTGGCGACCTATGACTATGGCACCCCAGTTCTGG
GGACCTTCCACGGAAGTGACCTGCTGCAGGTGTTCTATGGGATCAAGCCAAACTATGCAGCTAGTTCTAGCCACACGTACTATCTGAGCTTTGTGTATACGCTGGATCCGA
ACTCCAACCGGGGGGAGTACATTGAGTGGCCGCAGTGGAAGGAATCGCGGCAGTTGATGAATTTCGGAGCGAACGACGCCAGTCTCCTTACGGATGATTTCCGCAACGG
GACATATGAGTTCATCCTGCAGAATACCGCGGCGTTCCACATCTGATGCCATTGGCGGAGGGGTCCGGACGGTCAGGAACTTAGCCTTATGAGATGAATGATGGACGTGT
CTGGCCTCGGAAAAGGATATATGGGGATCATAATAGTACTAGCCATATTAATGAAGGGCATATACCACGCGTTGGACCTGC GTTATAGCTTCCCGTTAGTTATAGTACCAT
CGTTATACCAGCCAATCAAGTCACCACGCACGACCGGGGACGGCGAATCCCCGGGAATTGAAAGAAATTGCATCCCAGGCCAGTGAGGCCAGCGATTGGCCACCTCTCC
AAGGCACAGGGCCATTCTGCAGCGCTGGTGGATTCATCGCAATTTCCCCCGGCCCGGCCCGACACCGCTATAGGCTGGTTCTCCCACACCATCGGAGATTCGTCGCCTAAT
GTCTCGTCCGTTCACAAGCTGAAGAGCTTGAAGTGGCGAGATGTCTCTGCAGGAATTCAAGCTAGATGCTAAGCGATATTGCATGGCAATATGTGTTGATGCATGTGCTTC
TTCCTTCAGCTTCCCCTCGTGCAGATGAGGTTTGGCTATAAATTGAAGTGGTTGGTCGGGGTTCCGTGAGGGGCTGAAGTGCTTCCTCCCTTTTAGACGCAACTGAGAGCC
TGAGCTTCATCCCCAGCATCATTACACCTCAGCACATGCCTGGAGAGGTCATCGACAGGCCAAATCCCAAGGCTGAGCCTTCACACATCCCCGATCTTGTCAATCAATTGC
AGGTCAAACTTCAAGAGACGCGTTTGGAGGAAACTGATTACAATGCCCTTCTGAAATTCCGCCGTGCAGCGGCCTACATTGCTGCTGCAATGATCTTTCTCCAAGACAATG
TGCTGCTGAAGCAGAATCTAAGGCACGAGGACATCAAGCCCAGGCTTCTTGGCCACTGGGGAACATGTCCCGGGTTGATTCTTGTATACTCTCACTTGAACTACATCATCA
GAAAGCAGAACCTGGATATGTTGTATGTCGTCGGGCCTGGCCACGGCGCGCCAGCTATTTTGGCCTCACTGTGGCTTGAGGGCTCTTTAGAGAAATTCTACCCCCACTACT
CACGAGACATGGATGGcCTCCATGAGCTCATCTCGACCTTCAGCACAAGTGCTGGATTACCAAGCCATATCAATGCGGAAACTCCCGGTGCAATCCATGAAGGTGGTGAA
TTGGGTTATGCGTTAGCTGTCTCTTTTGGTGCTGTTATGGACAATCCCGACATGATCGTCACCTGCGTGGTTGGTGACGGGGAAGCAGAAACTGGTCCTACCGCGACGTCC
TGGCATGCAATCAAGTACATTGACCCCGCAGAATCAGGTGCCGTCCTGCCGATTCTCCACGTTAATGGCTTTAAGATCAGCGAGCGCACCATTTATGGCTGCATGGACAA
CAAAGAGCTGGTgTCCCTCTTCACGGGTTATGGATACCAGGTGCGCATTGTTGAGAACCTGGATGACATCGACGCAGATCTCCATAGCTCTATGATGTGGGCAGTTGAGGA
GATCCACAAGATCCAAAAAGCGGCGCGTTCCGGCAAGCCAATTATGAAGCCTAGATGGCCAATGATTGTTTTGCGCACACCGAAGGGTTGGTCAGGACCTAAAGAGCTC
CACGGGTCATTCATAGAGGGATCTTTCCACTCACATCAGGTTCCTCTACCTAATGCAAAGAAGGATAAAGAGGAGCTTCAGGCTCTGCAGAAATGGCTGTCCTCGTATAA
TCCGCACGAACTTTTCACTGAGACGGGAGACATCATTGACGACATCAAGTCAGTGATCCCTCTGGAgGACACCAAGAAGCTTGGGCAGCGAGCAGAAGCCTACAAGGGCT
ATAGGGCACCCGATCTCCCAGACTGGCGCAAGTTTGGCGTAGAAAAGGGCTCCCAGCAGAGCGCTATGAAAACAATTGGAAAGTTCATTGACCAAGTGTTTACCCAAAA
TCCTCATGGCGTCCGTGTATTTTCGCCAGACGAGCTAGAGAGCAACAAGCTGGATGCAGCACTGGCGCACACGGGAAGGAACTTTCAGTGGGATCAATTCTCGAATGCCA
AAGGCGGCCGCGTCATCGAGGTGCTCAGTGAGCACCTGTGCCAGGGCTTTATGCAGGGATACACGTTGACGGGCCGGGTGGGCATTTTCCCATCGTACGAAAGCTTCTTG
GGAATCATCCATACCATGATGGTGCAATATGCCAAATTTAACAAAATGGCTCAAGAGACGACCTGGCATAAGCCGGTTAGTAGCATCAACTATATCGAAACGAGTACGTG
GGCTCGTCAGGAGCACAATGGATTCTCTCACCAGAACCCCTCCTTTATCGGAGCTGTGCTCAGGCTGAAGCCCACCGCCGCGCGAGTTTATCTGCCACCTGATGCTAACAC
ATTTTTGACCACCCTTCACCACTGTCTCAAGTCCAAGAATTATGTCAACCTCATGGTAGGTTCAAAACAGCCAACTCCCGTGTACTTGAGCCCCGAGGAAGCAGAGAGCC
ACTGCCGAGCCGGCGCATCGATCTGGAGATTCTGTAGTACCGACAATGGGCTGAACCCGGATGTCGTGCTGGTTGGCATTGGAGTAGAGGTGATGTTCGAGGTCATCTAC
GCGGCGGCCATCCTCCGCAAGCGTTGTCCAGAACTCCGGGTGCGTGTGGTCAATGTGACCGACTTGATGATTCTGGAGAAGGAAGGTCTACATCCACATGCATTGACGAC
CGAAGCTTTCGACAGTCTGTTTGGCTCGGACCGGCCGATACACTTCAACTACCACGGATACCCGGGCGAGCTCAAAGGTCTGCTCTTTGGGCGGCCCCGCCTGGACCGAG
TTTCAGTAGAAGGATACATGGAGGAAGGAAGCACGACGACGCCGTTCGATATGATGTTGCTGAACCGCGTCTCACGATACCACGTGGCGCAGGCAGCCGTGATCGGGGC
GTCCAGACGGAATGAGAAGGTTCAAGTTCGGCAGCACGAACTGGTCAGCGAATTCGGCCACAACATCGTGGAGACACGCAAATACATTCTGGCCAACCGCAAAGACCCG
GATGATACGTATGATATGCCCTCCTTTGAATAAGCTTGATCCACTTAACGTTACTGAAATCATCAAACAGCTTGACGAATCTGGATATAAGATCGTTGGTGTCGATGTCAG
CTCCGGAGTTGAGACAAATGGTGTTCAGGATCTCGATAAGATACGTTCATTTGTCCAAGCAGCAAAGAGTGCCTTCTAGTGATTTAATAGCTCCATGTCAACAAGAATAA
AACGCGTTTCGGGTTTACCTCTTCCAGATACAGCTCATCTGCAATGCATTAATGCATTGGACCTCGCAACCCTAGTACGCCCTTCAGGCTCCGGCGAAGCAGAAGAATAGC
TTAGCAGAGTCTATTTTCATTTTCGGGAGACGAGATCAAGCAGATCAACGGTCGTCAAGAGTCCTACGAGACTGAGGAATCCGCTCTTGGCTCCACGCGACTATATATTTG
TCTCTAATTGTACTTTGACATGCTCCTCTTCTTTACTCTGATAGCTTGACTATGAAAATTCCGTCACCAGCCCCTGGGTTCGCAAAGATAATTGCACTGTTTCTTCCTTGAAC
TCTCAAGCCTACAGGACACACATTCATCGTAGGTATAAACCTCGAAAATCATTCCTACTAAGATGGGTATACAATAGTAACCATGCATGGTTGCCTAGTGAATGCTCCGTA
ACACCCAATACGCCGGCCGAAACTTTTTTACAACTCTCCTATGAGTCGTTTACCCAGAATGCACAGGTACACTTGTTTAGAGGTAATCCTTCTTTCTAGAAGTCCTCGTGTA
CTGTGTAAGCGCCCACTCCACATCTCCACTCGCTCCGGGGAGACGGGCACAAGGTTCATGAACCGAAGAGGATGGGAACCCCGACCATGGCCTGCGGCCGCAGCTATCA
GTCCTCTCAGCTGACGCCCCTCACGAACCCTCGCACTCTCCCGAAATGCTCTTCGGTGTCCATGGCGCATCATCTCAGCAGCATCCCATGTCAAACCATGGCTTTGGGCCC
ACGGACTCTGTGGCCCTGCCGCAACATCACCATCACCATCGACTCCCGCCCCATGCAGCGCTGCGCGCCCCAGGGCGTCATGGAGTGAATGTGGAATCTCCTCCTTTGCCC
TCGGGCCCGCCGAGTGTGGTGGGCCAGCCTGGAATGCCTGATCCAGCCCCCAGGCCTCGAGGACCAAAACTGAAGTTTACTCCCGAAGAGGACGCTCTACTGGTGGAGTT
GAAGGAAAACAAGAACTTGACGTGGAAGCAAATTGCAGACTTCTTCCCGGGCCGAACGAGCGGTACCTTGCAAGTCCGATACTGCACCAAGCTGAAGGCTAAGGATGTA
GCTTGGAGTGACGAAATGGTTCGATTTGCTCCTGATGTATTTCTACGCCTGTCTCACACATGCTAATGAAGGGAATAGGTACAAAGGCTGCAGCGGGCAATGCACGAGTA
CGAGAACGATCGGTGGCGCATCATTGCAGGGAAGGTTGGAAATGGCTTCACCCCAGCTGCTTGCCGCGAGAAAGCCATGCAGCTCCATGAGTAAAAGCGTTGGGGAATT
TTCATATTTATATCTACTGTCGCCAGATTCGGCCCTGCTTGGACCCTCTGATCTCCTTACTCTCCATATTGGTTCAAATGTCGGGTCACCGATAGGGCTGGTGGTGCAGGCT
TGTTGTAGGCACGGGAGGATGATCAGCATAACTCTGAGTCACTATAGGGACGGGTTGATGTAAGGTATTAAGTGATGTATGATAATTCATTTTAGCCCGGGGGAACATAT
GGCGCCGGCATTTGTTCGTTCGCAATGAACCGACACTAGCGTCCGCTCTCGCAGTTTAGCACCGGCTGATCCCGGGCTGAACGCGGCCATTGCTCGGCCGGGGCATGTGTT
CCTTATCTACGGCAGACCGCAGATGACCACTGGAGCAGATTATAGACCCTAAGCCCTAAGCCGGACACCCAATCGAGTAGGTCTGCGGACCAGGTCACTGCGGGCAGCC
GGAGAAGCTCCGCAACCAATCAATCCCCGGCGCTGACTAAGGGCAGGCGACCACGGGCCGAAGCGGCTTCAAACTCACCTCAACCTCCAAACTCCCTCATCTCCAAACGT
CCTTGCCTTGTCTGCCGTCATTGCAACCCACCCACCAGGACACATGAGCAGCAAGTCCCAGCTCACCTACACTGCCCGTGCCAGCAAGCATCCCAATGCTCTGGCGAAGA
GGCTGTTCGAGATTGCCGAGGCCAAGAAaACCAATGTGACTGTCTCGGCTGACGTTACCACCACTAAGGAGCTACTAGATCTTGCTGACCGTCTCGGTCCCTACATTGCCG
TGATCAAAACCCACATCGATATCCTCTCTGATTTCAGCAACGAGACtATTGAGGGACTTAAGGCTCTCGCGCAGAAGCACAACTTTCTCATCTTCGAGGACCGCAAGTTCA
TTGACATCGGCAACACGGTCCAGAAGCAATACCACGGCGGTACCCTCCGTATCTCGGAATGGGCCCACATCATCAACTGCAGCATTCTCCCTGGTGAGGGTATCGTCGAG
GCTCTCGCTCAGACGGCGTCTGCACCGGACTTCGCCTACGGCCCCGAACGCGGTCTGTTGATCTTGGCAGAGATGACCTCTAAGGGCTCCTTGGCTACCGGCCAGTACACT
ACTTCCTCGGTCGATTATGCCCGGAAATACAAGAACTTCGTTATGGGATTCGTGTCGACGCGCGCGTTGGGTGAGGTGCAGTCGGAAGTCAGCTCTCCTTCGGATGAGGA
GGACTTTGTGGTtTTCACGACTGGTGTGAACATTAACCTCCA

>pSF530_IC
TGGAGGaAGGGATCCGCCAAGGATGCTCTGGATCCGCATCCGGCCGCTCTTGCGCCCCATCAATCGCCCGACTATAAATCGAACTACTTTCGGCATCTTCTAGACTTCCTA
ATACCGCCTAGTCATAGCAGATTCAAGCTGAGAACACCACAAGTAAATATCACCCATCATGCTTACCCTGACCGTCCCTGAAAACTACGGGTATGTGCCAATTCTACAATT
CCTTGCAGACAATGCCATTCTCCCCATGAAGTCTGATGCTAACTATCCTGCAGCTCTGTCATTGCCGTCGCTCTGGGTGCCATCCCCGTCCTGAGCTTCGTCCATGGCGCCG

TCGTGTCTCGTCTCCGCAAGGAAGCTGATTGCCCCTACCCTCACTGCTATGCGACCGTAGAGCAGTGCAAGACCAACGTAAGCCAACCTCACACAAACAGGATTCCTCGA
GCTAACATACATTCCGAACCGTGCAGCCCAAGGCCGAGCAGTTCAACTGCGCTCAGCGCGCTCATGCCAACTTCCTTGAGAACTCCAGCCAAACTATGCTCTTCCTCCTGG
TAGCTGGACTGAAGTACCCCCAGTTGGCGACTGGCCTCGGAAGCATCTGGGTCCTCGGTCGCTCACTGTTCCTTTACGGATATGTGTACTCCGGCAAGCCGCGGGGTCGCG
GTCGTTTGTACGGCAGCTTCTACTTGCTTGCACAGGGAGCTCTCTGGGGCTTGACGTCTTTTGGAGTTGCGAGGGAGTTGATTTCCTACTTCTAAGTTTGGACTGAATCCGT
GGTGTGATTGAGGTGATTGGCGATGTTTGGCTATACCAGCTATATGTAATAATCTCTACTGTATACTACTATTCAACGCATTTTACTATGCGTGCTGCTAGGGTCGGCAATG
ACAATGGCAATCTGACTGACGTGGTCTATTTCTCCATGTGCAGCAGGGAATACGAGCTCCAATGGACCTCGGGAGTGGCACAGTCAATGGCAAGGAAACTGGAGcgcaaaaaa
ccccgcccctgacagggcggggtttttcgcGATCGGAGTGTGCTGTGGTTAGGGGGGGGAAAGGGAGGATTATTGAGTGCCTATTTTTGGGATCAATATGACTGACTATGGTCATGGCTAG
GACTATAGATTTGGTGGTGTAAGATTTCATTGTTCATATTGGATGGGATTGGCATGGAATGAAAGAACTGAAATGAGGGGAGAGAGATTCTAGACAAGTATCTATTATGT
ATCTAGTGGATGGATCGAGGTCTAGACGTAGTGAGTATACTAAGTGGACTAGGGACAAGGTAACTATGTAGTAGGTAGTGATGAGTGTATTCCAGAAGTACTGACTGCAG
GCGCTGTCAGCACCCACACGCACACGCACACACACTAAATGCACCACTTCCACAATACCCTAATGGAGTGACTACGATGGATGGCAGATGATGGCAGATGGCAGTGGAT
TCCGTCTCGTGGCGTCTCCAGGAACTTCTTCTCCACCTGCGCCCAACTTCCAGCACAACTTGGAAAAGGCGAAAAAGAAAAGAAAAAGAAAAAAAGAAAAATGCAATTC
AATGGCTCGCCAGGGACACTCCAATCCACGTCAGTGGCCATGTGGACCCCGAAGCCAAACAAACTTTTTACCCCTGCGCCTCTGGCGGCGATTCGTTGAAACGGCCAGGA
ACAGCAGGAACAGCAGTATGAGCACTAGCCGATCTCTTCGCCGACGGTTGGATGCCTTGCCGCCGCAAAAAGCTATATAAGGATGCATCTCGCTGCACCTTTTGCTTCCTC
TTCAATCCATCAGCAAATCCACTGACACTCTCTTGATCAGCTTTGTCTGACCTGTCATTCTCTCTCTTTTTTGATACATCTGATTTTAGTCTGTCGATCAGACTAGTCTGCCT
TCCTTTTAGTCCTCTCTCTTTTGGACTTTACTACTACACATACAACAAACCACTTTCTCTTTTTCAGAGGAACAACAACAGTCTTTCTTGACAACCCAAAATCTCATCTTTTT
TGAGTCAATCTCTTCAAACAAATCTCATCAAACATGCCTGGAGAGGTCATCGACAGGCCAAATCCCAAGGCTGAGCCTTCACACATCCCCGATCTTGTCAATCAATTGCA
GGTCAAACTTCAAGAGACGCGTTTGGAGGAAACTGATTACAATGCCCTTCTGAAATTCCGCCGTGCAGCGGCCTACATTGCTGCTGCAATGATCTTTCTCCAAGACAATGT
GCTGCTGAAGCAGAATCTAAGGCACGAGGACATCAAGCCCAGGCTTCTTGGCCACTGGGGAACATGTCCCGGGTTGATTCTTGTATACTCTCACTTGAACTACATCATCA
GAAAGCAGAACCTGGATATGTTGTATGTCGTCGGGCCTGGCCACGGCGCGCCAGCTATTTTGGCCTCACTGTGGCTTGAGGGCTCTTTAGAGAAATTCTACCCCCACTACT
CACGAGACATGGATGGcCTCCATGAGCTCATCTCGACCTTCAGCACAAGTGCTGGATTACCAAGCCATATCAATGCGGAAACTCCCGGTGCAATCCATGAAGGTGGTGAA
TTGGGTTATGCGTTAGCTGTCTCTTTTGGTGCTGTTATGGACAATCCCGACATGATCGTCACCTGCGTGGTTGGTGACGGGGAAGCAGAAACTGGTCCTACCGCGACGTCC
TGGCATGCAATCAAGTACATTGACCCCGCAGAATCAGGTGCCGTCCTGCCGATTCTCCACGTTAATGGCTTTAAGATCAGCGAGCGCACCATTTATGGCTGCATGGACAA
CAAAGAGCTGGTgTCCCTCTTCACGGGTTATGGATACCAGGTGCGCATTGTTGAGAACCTGGATGACATCGACGCAGATCTCCATAGCTCTATGATGTGGGCAGTTGAGGA
GATCCACAAGATCCAAAAAGCGGCGCGTTCCGGCAAGCCAATTATGAAGCCTAGATGGCCAATGATTGTTTTGCGCACACCGAAGGGTTGGTCAGGACCTAAAGAGCTC
CACGGGTCATTCATAGAGGGATCTTTCCACTCACATCAGGTTCCTCTACCTAATGCAAAGAAGGATAAAGAGGAGCTTCAGGCTCTGCAGAAATGGCTGTCCTCGTATAA
TCCGCACGAACTTTTCACTGAGACGGGAGACATCATTGACGACATCAAGTCAGTGATCCCTCTGGAgGACACCAAGAAGCTTGGGCAGCGAGCAGAAGCCTACAAGGGCT
ATAGGGCACCCGATCTCCCAGACTGGCGCAAGTTTGGCGTAGAAAAGGGCTCCCAGCAGAGCGCTATGAAAACAATTGGAAAGTTCATTGACCAAGTGTTTACCCAAAA
TCCTCATGGCGTCCGTGTATTTTCGCCAGACGAGCTAGAGAGCAACAAGCTGGATGCAGCACTGGCGCACACGGGAAGGAACTTTCAGTGGGATCAATTCTCGAATGCCA
AAGGCGGCCGCGTCATCGAGGTGCTCAGTGAGCACCTGTGCCAGGGCTTTATGCAGGGATACACGTTGACGGGCCGGGTGGGCATTTTCCCATCGTACGAAAGCTTCTTG
GGAATCATCCATACCATGATGGTGCAATATGCCAAATTTAACAAAATGGCTCAAGAGACGACCTGGCATAAGCCGGTTAGTAGCATCAACTATATCGAAACGAGTACGTG
GGCTCGTCAGGAGCACAATGGATTCTCTCACCAGAACCCCTCCTTTATCGGAGCTGTGCTCAGGCTGAAGCCCACCGCCGCGCGAGTTTATCTGCCACCTGATGCTAACAC
ATTTTTGACCACCCTTCACCACTGTCTCAAGTCCAAGAATTATGTCAACCTCATGGTAGGTTCAAAACAGCCAACTCCCGTGTACTTGAGCCCCGAGGAAGCAGAGAGCC
ACTGCCGAGCCGGCGCATCGATCTGGAGATTCTGTAGTACCGACAATGGGCTGAACCCGGATGTCGTGCTGGTTGGCATTGGAGTAGAGGTGATGTTCGAGGTCATCTAC
GCGGCGGCCATCCTCCGCAAGCGTTGTCCAGAACTCCGGGTGCGTGTGGTCAATGTGACCGACTTGATGATTCTGGAGAAGGAAGGTCTACATCCACATGCATTGACGAC
CGAAGCTTTCGACAGTCTGTTTGGCTCGGACCGGCCGATACACTTCAACTACCACGGATACCCGGGCGAGCTCAAAGGTCTGCTCTTTGGGCGGCCCCGCCTGGACCGAG
TTTCAGTAGAAGGATACATGGAGGAAGGAAGCACGACGACGCCGTTCGATATGATGTTGCTGAACCGCGTCTCACGATACCACGTGGCGCAGGCAGCCGTGATCGGGGC
GTCCAGACGGAATGAGAAGGTTCAAGTTCGGCAGCACGAACTGGTCAGCGAATTCGGCCACAACATCGTGGAGACACGCAAATACATTCTGGCCAACCGCAAAGACCCG
GATGATACGTATGATATGCCCTCCTTTGAATAAGCTTGATCCACTTAACGTTACTGAAATCATCAAACAGCTTGACGAATCTGGATATAAGATCGTTGGTGTCGATGTCAG
CTCCGGAGTTGAGACAAATGGTGTTCAGGATCTCGATAAGATACGTTCATTTGTCCAAGCAGCAAAGAGTGCCTTCTAGTGATTTAATAGCTCCATGTCAACAAGAATAA
AACGCGTTTCGGGTTTACCTCTTCCAGATACAGCTCATCTGCAATGCATTAATGCATTGGACCTCGCAACCCTAGTACGCCCTTCAGGCTCCGGCGAAGCAGAAGAATAGC
TTAGCAGAGTCTATTTTCATTTTCGGGAGACGAGATCAAGCAGATCAACGGTCGTCAAGAGTCCTACGAGACTGAGGAATCCGCTCTTGGCTCCACGCGACTATATATTTG
TCTCTAATTGTACTTTGACATGCTCCTCTTCTTTACTCTGATAGCTTGACTATGAAAATTCCGTCACCAGCCCCTGGGTTCGCAAAGATAATTGCACTGTTTCTTCCTTGAAC
TCTCAAGCCTACAGGACACACATTCATCGTAGGTATAAACCTCGAAAATCATTCCTACTAAGATGGGTATACAATAGTAACCATGCATGGTTGCCTAGTGAATGCTCCGTA
ACACCCAATACGCCGGCCGAAACTTTTTTACAACTCTCCTATGAGTCGTTTACCCAGAATGCACAGGTACACTTGTTTAGAGGTAATCCTTCTTTCTAGAAGTCCTCGTGTA
CTGTGTAAGCGCCCACTCCACATCTCCACTCGCTCCGGGGAGACGGGCACAAGGTTCATGAACCGAAGAGGATGGGAACCCCGACCATGGCCTGCGGCCGCAGCTATCA
GTCCTCTCAGCTGACGCCCCTCACGAACCCTCGCACTCTCCCGAAATGCTCTTCGGTGTCCATGGCGCATCATCTCAGCAGCATCCCATGTCAAACCATGGCTTTGGGCCC
ACGGACTCTGTGGCCCTGCCGCAACATCACCATCACCATCGACTCCCGCCCCATGCAGCGCTGCGCGCCCCAGGGCGTCATGGAGTGAATGTGGAATCTCCTCCTTTGCCC
TCGGGCCCGCCGAGTGTGGTGGGCCAGCCTGGAATGCCTGATCCAGCCCCCAGGCCTCGAGGACCAAAACTGAAGTTTACTCCCGAAGAGGACGCTCTACTGGTGGAGTT
GAAGGAAAACAAGAACTTGACGTGGAAGCAAATTGCAGACTTCTTCCCGGGCCGAACGAGCGGTACCTTGCAAGTCCGATACTGCACCAAGCTGAAGGCTAAGGATGTA
GCTTGGAGTGACGAAATGGTTCGATTTGCTCCTGATGTATTTCTACGCCTGTCTCACACATGCTAATGAAGGGAATAGGTACAAAGGCTGCAGCGGGCAATGCACGAGTA
CGAGAACGATCGGTGGCGCATCATTGCAGGGAAGGTTGGAAATGGCTTCACCCCAGCTGCTTGCCGCGAGAAAGCCATGCAGCTCCATGAGTAAAAGCGTTGGGGAATT
TTCATATTTATATCTACTGTCGCCAGATTCGGCCCTGCTTGGACCCTCTGATCTCCTTACTCTCCATATTGGTTCAAATGTCGGGTCACCGATAGGGCTGGTGGTGCAGGCT
TGTTGTAGGCACGGGAGGATGATCAGCATAACTCTGAGTCACTATAGGGACGGGTTGATGTAAGGTATTAAGTGATGTATGATAATTCATTTTAGCCCGGGGGAACATAT
GGCGCCGGCATTTGTTCGTTCGCAATGAACCGACACTAGCGTCCGCTCTCGCAGTTTAGCACCGGCTGATCCCGGGCTGAACGCGGCCATTGCTCGGCCGGGGCATGTGTT
CCTTATCTACGGCAGACCGCAGATGACCACTGGAGCAGATTATAGACCCTAAGCCCTAAGCCGGACACCCAATCGAGTAGGTCTGCGGACCAGGTCACTGCGGGCAGCC
GGAGAAGCTCCGCAACCAATCAATCCCCGGCGCTGACTAAGGGCAGGCGACCACGGGCCGAAGCGGCTTCAAACTCACCTCAACCTCCAAACTCCCTCATCTCCAAACGT
CCTTGCCTTGTCTGCCGTCATTGCAACCCACCCACCAGGACACATGAGCAGCAAGTCCCAGCTCACCTACACTGCCCGTGCCAGCAAGCATCCCAATGCTCTGGCGAAGA
GGCTGTTCGAGATTGCCGAGGCCAAGAAaACCAATGTGACTGTCTCGGCTGACGTTACCACCACTAAGGAGCTACTAGATCTTGCTGACCGTCTCGGTCCCTACATTGCCG
TGATCAAAACCCACATCGATATCCTCTCTGATTTCAGCAACGAGACtATTGAGGGACTTAAGGCTCTCGCGCAGAAGCACAACTTTCTCATCTTCGAGGACCGCAAGTTCA
TTGACATCGGCAACACGGTCCAGAAGCAATACCACGGCGGTACCCTCCGTATCTCGGAATGGGCCCACATCATCAACTGCAGCATTCTCCCTGGTGAGGGTATCGTCGAG
GCTCTCGCTCAGACGGCGTCTGCACCGGACTTCGCCTACGGCCCCGAACGCGGTCTGTTGATCTTGGCAGAGATGACCTCTAAGGGCTCCTTGGCTACCGGCCAGTACACT
ACTTCCTCGGTCGATTATGCCCGGAAATACAAGAACTTCGTTATGGGATTCGTGTCGACGCGCGCGTTGGGTGAGGTGCAGTCGGAAGTCAGCTCTCCTTCGGATGAGGA
GGACTTTGTGGTtTTCACGACTGGTGTGAACATTAACCTCCA

>pSF534_IC
TGGAGGaAGGGATCCGCCAAGGATGCTCTGGATCCGCATCCGGCCGCTCTTGCGCCCCATCAATCGCCCGACTATAAATCGAACTACTTTCGGCATCTTCTAGACTTCCTAATA
CCGCCTAGTCATAGCAGATTCAAGCTGAGAACACCACAAGTAAATATCACCCATCATGCTTACCCTGACCGTCCCTGAAAACTACGGGTATGTGCCAATTCTACAATTCCTTG
CAGACAATGCCATTCTCCCCATGAAGTCTGATGCTAACTATCCTGCAGCTCTGTCATTGCCGTCGCTCTGGGTGCCATCCCCGTCCTGAGCTTCGTCCATGGCGCCGTCGTGT
CTCGTCTCCGCAAGGAAGCTGATTGCCCCTACCCTCACTGCTATGCGACCGTAGAGCAGTGCAAGACCAACGTAAGCCAACCTCACACAAACAGGATTCCTCGAGCTAACA
TACATTCCGAACCGTGCAGCCCAAGGCCGAGCAGTTCAACTGCGCTCAGCGCGCTCATGCCAACTTCCTTGAGAACTCCAGCCAAACTATGCTCTTCCTCCTGGTAGCTGGA
CTGAAGTACCCCCAGTTGGCGACTGGCCTCGGAAGCATCTGGGTCCTCGGTCGCTCACTGTTCCTTTACGGATATGTGTACTCCGGCAAGCCGCGGGGTCGCGGTCGTTTGT
ACGGCAGCTTCTACTTGCTTGCACAGGGAGCTCTCTGGGGCTTGACGTCTTTTGGAGTTGCGAGGGAGTTGATTTCCTACTTCTAAGTTTGGACTGAATCCGTGGTGTGATT
GAGGTGATTGGCGATGTTTGGCTATACCAGCTATATGTAATAATCTCTACTGTATACTACTATTCAACGCATTTTACTATGCGTGCTGCTAGGGTCGGCAATGACAATGGCAATC
TGACTGACGTGGTCTATTTCTCCATGTGCAGCAGGGAATACGAGCTCCAATGGACCTCGGGAGTGGCACAGTCAATGGCAAGGAAACTGGAGcgcaaaaaaccccgcccctgacagggcg
gggtttttcgcGATCGGAGCCTCTCGTATGCAGAGGAAATCTCCCCTGATCTTCCGAACTGGTCGTACCTGGCGACCTATGACTATGGCACCCCAGTTCTGGGGACCTTCCACGGA
AGTGACCTGCTGCAGGTGTTCTATGGGATCAAGCCAAACTATGCAGCTAGTTCTAGCCACACGTACTATCTGAGCTTTGTGTATACGCTGGATCCGAACTCCAACCGGGGGG
AGTACATTGAGTGGCCGCAGTGGAAGGAATCGCGGCAGTTGATGAATTTCGGAGCGAACGACGCCAGTCTCCTTACGGATGATTTCCGCAACGGGACATATGAGTTCATCCT
GCAGAATACCGCGGCGTTCCACATCTGATGCCATTGGCGGAGGGGTCCGGACGGTCAGGAACTTAGCCTTATGAGATGAATGATGGACGTGTCTGGCCTCGGAAAAGGATAT
ATGGGGATCATAATAGTACTAGCCATATTAATGAAGGGCATATACCACGCGTTGGACCTGCGTTATAGCTTCCCGTTAGTTATAGTACCATCGTTATACCAGCCAATCAAGTCAC
CACGCACGACCGGGGACGGCGAATCCCCGGGAATTGAAAGAAATTGCATCCCAGGCCAGTGAGGCCAGCGATTGGCCACCTCTCCAAGGCACAGGGCCATTCTGCAGCGC
TGGTGGATTCATCGCAATTTCCCCCGGCCCGGCCCGACACCGCTATAGGCTGGTTCTCCCACACCATCGGAGATTCGTCGCCTAATGTCTCGTCCGTTCACAAGCTGAAGAGC
TTGAAGTGGCGAGATGTCTCTGCAGGAATTCAAGCTAGATGCTAAGCGATATTGCATGGCAATATGTGTTGATGCATGTGCTTCTTCCTTCAGCTTCCCCTCGTGCAGATGAG
GTTTGGCTATAAATTGAAGTGGTTGGTCGGGGTTCCGTGAGGGGCTGAAGTGCTTCCTCCCTTTTAGACGCAACTGAGAGCCTGAGCTTCATCCCCAGCATCATTACACCTC
AGCACATGCCTGGAGAGGTCATCGACAGGCCAAATCCCAAGGCTGAGCCTTCACACATCCCCGATCTTGTCAATCAATTGCAGGTCAAACTTCAAGAGACGCGTTTGGAGG
AAACTGATTACAATGCCCTTCTGAAATTCCGCCGTGCAGCGGCCTACATTGCTGCTGCAATGATCTTTCTCCAAGACAATGTGCTGCTGAAGCAGAATCTAAGGCACGAGGA
CATCAAGCCCAGGCTTCTTGGCCACTGGGGAACATGTCCCGGGTTGATTCTTGTATACTCTCACTTGAACTACATCATCAGAAAGCAGAACCTGGATATGTTGTATGTCGTCG
GGCCTGGCCACGGCGCGCCAGCTATTTTGGCCTCACTGTGGCTTGAGGGCTCTTTAGAGAAATTCTACCCCCACTACTCACGAGACATGGATGGcCTCCATGAGCTCATCTCG
ACCTTCAGCACAAGTGCTGGATTACCAAGCCATATCAATGCGGAAACTCCCGGTGCAATCCATGAAGGTGGTGAATTGGGTTATGCGTTAGCTGTCTCTTTTGGTGCTGTTAT
GGACAATCCCGACATGATCGTCACCTGCGTGGTTGGTGACGGGGAAGCAGAAACTGGTCCTACCGCGACGTCCTGGCATGCAATCAAGTACATTGACCCCGCAGAATCAGG
TGCCGTCCTGCCGATTCTCCACGTTAATGGCTTTAAGATCAGCGAGCGCACCATTTATGGCTGCATGGACAACAAAGAGCTGGTgTCCCTCTTCACGGGTTATGGATACCAGG
TGCGCATTGTTGAGAACCTGGATGACATCGACGCAGATCTCCATAGCTCTATGATGTGGGCAGTTGAGGAGATCCACAAGATCCAAAAAGCGGCGCGTTCCGGCAAGCCAA
TTATGAAGCCTAGATGGCCAATGATTGTTTTGCGCACACCGAAGGGTTGGTCAGGACCTAAAGAGCTCCACGGGTCATTCATAGAGGGATCTTTCCACTCACATCAGGTTCC
TCTACCTAATGCAAAGAAGGATAAAGAGGAGCTTCAGGCTCTGCAGAAATGGCTGTCCTCGTATAATCCGCACGAACTTTTCACTGAGACGGGAGACATCATTGACGACATC
AAGTCAGTGATCCCTCTGGAgGACACCAAGAAGCTTGGGCAGCGAGCAGAAGCCTACAAGGGCTATAGGGCACCCGATCTCCCAGACTGGCGCAAGTTTGGCGTAGAAAA
GGGCTCCCAGCAGAGCGCTATGAAAACAATTGGAAAGTTCATTGACCAAGTGTTTACCCAAAATCCTCATGGCGTCCGTGTATTTTCGCCAGACGAGCTAGAGAGCAACAA
GCTGGATGCAGCACTGGCGCACACGGGAAGGAACTTTCAGTGGGATCAATTCTCGAATGCCAAAGGCGGCCGCGTCATCGAGGTGCTCAGTGAGCACCTGTGCCAGGGCT
TTATGCAGGGATACACGTTGACGGGCCGGGTGGGCATTTTCCCATCGTACGAAAGCTTCTTGGGAATCATCCATACCATGATGGTGCAATATGCCAAATTTAACAAAATGGCT
CAAGAGACGACCTGGCATAAGCCGGTTAGTAGCATCAACTATATCGAAACGAGTACGTGGGCTCGTCAGGAGCACAATGGATTCTCTCACCAGAACCCCTCCTTTATCGGAG
CTGTGCTCAGGCTGAAGCCCACCGCCGCGCGAGTTTATCTGCCACCTGATGCTAACACATTTTTGACCACCCTTCACCACTGTCTCAAGTCCAAGAATTATGTCAACCTCATG
GTAGGTTCAAAACAGCCAACTCCCGTGTACTTGAGCCCCGAGGAAGCAGAGAGCCACTGCCGAGCCGGCGCATCGATCTGGAGATTCTGTAGTACCGACAATGGGCTGAA
CCCGGATGTCGTGCTGGTTGGCATTGGAGTAGAGGTGATGTTCGAGGTCATCTACGCGGCGGCCATCCTCCGCAAGCGTTGTCCAGAACTCCGGGTGCGTGTGGTCAATGTG
ACCGACTTGATGATTCTGGAGAAGGAAGGTCTACATCCACATGCATTGACGACCGAAGCTTTCGACAGTCTGTTTGGCTCGGACCGGCCGATACACTTCAACTACCACGGAT
ACCCGGGCGAGCTCAAAGGTCTGCTCTTTGGGCGGCCCCGCCTGGACCGAGTTTCAGTAGAAGGATACATGGAGGAAGGAAGCACGACGACGCCGTTCGATATGATGTTGC

TGAACCGCGTCTCACGATACCACGTGGCGCAGGCAGCCGTGATCGGGGCGTCCAGACGGAATGAGAAGGTTCAAGTTCGGCAGCACGAACTGGTCAGCGAATTCGGCCAC
AACATCGTGGAGACACGCAAATACATTCTGGCCAACCGCAAAGACCCGGATGATACGTATGATATGCCCTCCTTTGAAcatcatcaccaccaccatTAAGCTTGATCCACTTAACGTTAC
TGAAATCATCAAACAGCTTGACGAATCTGGATATAAGATCGTTGGTGTCGATGTCAGCTCCGGAGTTGAGACAAATGGTGTTCAGGATCTCGATAAGATACGTTCATTTGTCC
AAGCAGCAAAGAGTGCCTTCTAGTGATTTAATAGCTCCATGTCAACAAGAATAAAACGCGTTTCGGGTTTACCTCTTCCAGATACAGCTCATCTGCAATGCATTAATGCATTG
GACCTCGCAACCCTAGTACGCCCTTCAGGCTCCGGCGAAGCAGAAGAATAGCTTAGCAGAGTCTATTTTCATTTTCGGGAGACGAGATCAAGCAGATCAACGGTCGTCAAG
AGTCCTACGAGACTGAGGAATCCGCTCTTGGCTCCACGCGACTATATATTTGTCTCTAATTGTACTTTGACATGCTCCTCTTCTTTACTCTGATAGCTTGACTATGAAAATTCCG
TCACCAGCCCCTGGGTTCGCAAAGATAATTGCACTGTTTCTTCCTTGAACTCTCAAGCCTACAGGACACACATTCATCGTAGGTATAAACCTCGAAAATCATTCCTACTAAGA
TGGGTATACAATAGTAACCATGCATGGTTGCCTAGTGAATGCTCCGTAACACCCAATACGCCGGCCGAAACTTTTTTACAACTCTCCTATGAGTCGTTTACCCAGAATGCACA
GGTACACTTGTTTAGAGGTAATCCTTCTTTCTAGAAGTCCTCGTGTACTGTGTAAGCGCCCACTCCACATCTCCACTCGCTCCGGGGAGACGGGCACAAGGTTCATGAACCG
AAGAGGATGGGAACCCCGACCATGGCCTGCGGCCGCAGCTATCAGTCCTCTCAGCTGACGCCCCTCACGAACCCTCGCACTCTCCCGAAATGCTCTTCGGTGTCCATGGCG
CATCATCTCAGCAGCATCCCATGTCAAACCATGGCTTTGGGCCCACGGACTCTGTGGCCCTGCCGCAACATCACCATCACCATCGACTCCCGCCCCATGCAGCGCTGCGCGC
CCCAGGGCGTCATGGAGTGAATGTGGAATCTCCTCCTTTGCCCTCGGGCCCGCCGAGTGTGGTGGGCCAGCCTGGAATGCCTGATCCAGCCCCCAGGCCTCGAGGACCAAA
ACTGAAGTTTACTCCCGAAGAGGACGCTCTACTGGTGGAGTTGAAGGAAAACAAGAACTTGACGTGGAAGCAAATTGCAGACTTCTTCCCGGGCCGAACGAGCGGTACCT
TGCAAGTCCGATACTGCACCAAGCTGAAGGCTAAGGATGTAGCTTGGAGTGACGAAATGGTTCGATTTGCTCCTGATGTATTTCTACGCCTGTCTCACACATGCTAATGAAGG
GAATAGGTACAAAGGCTGCAGCGGGCAATGCACGAGTACGAGAACGATCGGTGGCGCATCATTGCAGGGAAGGTTGGAAATGGCTTCACCCCAGCTGCTTGCCGCGAGAA
AGCCATGCAGCTCCATGAGTAAAAGCGTTGGGGAATTTTCATATTTATATCTACTGTCGCCAGATTCGGCCCTGCTTGGACCCTCTGATCTCCTTACTCTCCATATTGGTTCAAA
TGTCGGGTCACCGATAGGGCTGGTGGTGCAGGCTTGTTGTAGGCACGGGAGGATGATCAGCATAACTCTGAGTCACTATAGGGACGGGTTGATGTAAGGTATTAAGTGATGT
ATGATAATTCATTTTAGCCCGGGGGAACATATGGCGCCGGCATTTGTTCGTTCGCAATGAACCGACACTAGCGTCCGCTCTCGCAGTTTAGCACCGGCTGATCCCGGGCTGAA
CGCGGCCATTGCTCGGCCGGGGCATGTGTTCCTTATCTACGGCAGACCGCAGATGACCACTGGAGCAGATTATAGACCCTAAGCCCTAAGCCGGACACCCAATCGAGTAGGT
CTGCGGACCAGGTCACTGCGGGCAGCCGGAGAAGCTCCGCAACCAATCAATCCCCGGCGCTGACTAAGGGCAGGCGACCACGGGCCGAAGCGGCTTCAAACTCACCTCA
ACCTCCAAACTCCCTCATCTCCAAACGTCCTTGCCTTGTCTGCCGTCATTGCAACCCACCCACCAGGACACATGAGCAGCAAGTCCCAGCTCACCTACACTGCCCGTGCCAG
CAAGCATCCCAATGCTCTGGCGAAGAGGCTGTTCGAGATTGCCGAGGCCAAGAAaACCAATGTGACTGTCTCGGCTGACGTTACCACCACTAAGGAGCTACTAGATCTTGCT
GACCGTCTCGGTCCCTACATTGCCGTGATCAAAACCCACATCGATATCCTCTCTGATTTCAGCAACGAGACtATTGAGGGACTTAAGGCTCTCGCGCAGAAGCACAACTTTCT
CATCTTCGAGGACCGCAAGTTCATTGACATCGGCAACACGGTCCAGAAGCAATACCACGGCGGTACCCTCCGTATCTCGGAATGGGCCCACATCATCAACTGCAGCATTCTC
CCTGGTGAGGGTATCGTCGAGGCTCTCGCTCAGACGGCGTCTGCACCGGACTTCGCCTACGGCCCCGAACGCGGTCTGTTGATCTTGGCAGAGATGACCTCTAAGGGCTCC
TTGGCTACCGGCCAGTACACTACTTCCTCGGTCGATTATGCCCGGAAATACAAGAACTTCGTTATGGGATTCGTGTCGACGCGCGCGTTGGGTGAGGTGCAGTCGGAAGTCA
GCTCTCCTTCGGATGAGGAGGACTTTGTGGTtTTCACGACTGGTGTGAACATTAACCTCCA

>pSF535_IC
TGGAGGaAGGGATCCGCCAAGGATGCTCTGGATCCGCATCCGGCCGCTCTTGCGCCCCATCAATCGCCCGACTATAAATCGAACTACTTTCGGCATCTTCTAGACTTCCTA
ATACCGCCTAGTCATAGCAGATTCAAGCTGAGAACACCACAAGTAAATATCACCCATCATGCTTACCCTGACCGTCCCTGAAAACTACGGGTATGTGCCAATTCTACAATT
CCTTGCAGACAATGCCATTCTCCCCATGAAGTCTGATGCTAACTATCCTGCAGCTCTGTCATTGCCGTCGCTCTGGGTGCCATCCCCGTCCTGAGCTTCGTCCATGGCGCCG
TCGTGTCTCGTCTCCGCAAGGAAGCTGATTGCCCCTACCCTCACTGCTATGCGACCGTAGAGCAGTGCAAGACCAACGTAAGCCAACCTCACACAAACAGGATTCCTCGA
GCTAACATACATTCCGAACCGTGCAGCCCAAGGCCGAGCAGTTCAACTGCGCTCAGCGCGCTCATGCCAACTTCCTTGAGAACTCCAGCCAAACTATGCTCTTCCTCCTGG
TAGCTGGACTGAAGTACCCCCAGTTGGCGACTGGCCTCGGAAGCATCTGGGTCCTCGGTCGCTCACTGTTCCTTTACGGATATGTGTACTCCGGCAAGCCGCGGGGTCGCG
GTCGTTTGTACGGCAGCTTCTACTTGCTTGCACAGGGAGCTCTCTGGGGCTTGACGTCTTTTGGAGTTGCGAGGGAGTTGATTTCCTACTTCTAAGTTTGGACTGAATCCGT
GGTGTGATTGAGGTGATTGGCGATGTTTGGCTATACCAGCTATATGTAATAATCTCTACTGTATACTACTATTCAACGCATTTTACTATGCGTGCTGCTAGGGTCGGCAATG
ACAATGGCAATCTGACTGACGTGGTCTATTTCTCCATGTGCAGCAGGGAATACGAGCTCCAATGGACCTCGGGAGTGGCACAGTCAATGGCAAGGAAACTGGAGcgcaaaaaa
ccccgcccctgacagggcggggtttttcgcGATCGGAGTGTGCTGTGGTTAGGGGGGGGAAAGGGAGGATTATTGAGTGCCTATTTTTGGGATCAATATGACTGACTATGGTCATGGCTAG
GACTATAGATTTGGTGGTGTAAGATTTCATTGTTCATATTGGATGGGATTGGCATGGAATGAAAGAACTGAAATGAGGGGAGAGAGATTCTAGACAAGTATCTATTATGT
ATCTAGTGGATGGATCGAGGTCTAGACGTAGTGAGTATACTAAGTGGACTAGGGACAAGGTAACTATGTAGTAGGTAGTGATGAGTGTATTCCAGAAGTACTGACTGCAG
GCGCTGTCAGCACCCACACGCACACGCACACACACTAAATGCACCACTTCCACAATACCCTAATGGAGTGACTACGATGGATGGCAGATGATGGCAGATGGCAGTGGAT
TCCGTCTCGTGGCGTCTCCAGGAACTTCTTCTCCACCTGCGCCCAACTTCCAGCACAACTTGGAAAAGGCGAAAAAGAAAAGAAAAAGAAAAAAAGAAAAATGCAATTC
AATGGCTCGCCAGGGACACTCCAATCCACGTCAGTGGCCATGTGGACCCCGAAGCCAAACAAACTTTTTACCCCTGCGCCTCTGGCGGCGATTCGTTGAAACGGCCAGGA
ACAGCAGGAACAGCAGTATGAGCACTAGCCGATCTCTTCGCCGACGGTTGGATGCCTTGCCGCCGCAAAAAGCTATATAAGGATGCATCTCGCTGCACCTTTTGCTTCCTC
TTCAATCCATCAGCAAATCCACTGACACTCTCTTGATCAGCTTTGTCTGACCTGTCATTCTCTCTCTTTTTTGATACATCTGATTTTAGTCTGTCGATCAGACTAGTCTGCCT
TCCTTTTAGTCCTCTCTCTTTTGGACTTTACTACTACACATACAACAAACCACTTTCTCTTTTTCAGAGGAACAACAACAGTCTTTCTTGACAACCCAAAATCTCATCTTTTT
TGAGTCAATCTCTTCAAACAAATCTCATCAAACATGCCTGGAGAGGTCATCGACAGGCCAAATCCCAAGGCTGAGCCTTCACACATCCCCGATCTTGTCAATCAATTGCA
GGTCAAACTTCAAGAGACGCGTTTGGAGGAAACTGATTACAATGCCCTTCTGAAATTCCGCCGTGCAGCGGCCTACATTGCTGCTGCAATGATCTTTCTCCAAGACAATGT
GCTGCTGAAGCAGAATCTAAGGCACGAGGACATCAAGCCCAGGCTTCTTGGCCACTGGGGAACATGTCCCGGGTTGATTCTTGTATACTCTCACTTGAACTACATCATCA
GAAAGCAGAACCTGGATATGTTGTATGTCGTCGGGCCTGGCCACGGCGCGCCAGCTATTTTGGCCTCACTGTGGCTTGAGGGCTCTTTAGAGAAATTCTACCCCCACTACT
CACGAGACATGGATGGcCTCCATGAGCTCATCTCGACCTTCAGCACAAGTGCTGGATTACCAAGCCATATCAATGCGGAAACTCCCGGTGCAATCCATGAAGGTGGTGAA
TTGGGTTATGCGTTAGCTGTCTCTTTTGGTGCTGTTATGGACAATCCCGACATGATCGTCACCTGCGTGGTTGGTGACGGGGAAGCAGAAACTGGTCCTACCGCGACGTCC
TGGCATGCAATCAAGTACATTGACCCCGCAGAATCAGGTGCCGTCCTGCCGATTCTCCACGTTAATGGCTTTAAGATCAGCGAGCGCACCATTTATGGCTGCATGGACAA
CAAAGAGCTGGTgTCCCTCTTCACGGGTTATGGATACCAGGTGCGCATTGTTGAGAACCTGGATGACATCGACGCAGATCTCCATAGCTCTATGATGTGGGCAGTTGAGGA
GATCCACAAGATCCAAAAAGCGGCGCGTTCCGGCAAGCCAATTATGAAGCCTAGATGGCCAATGATTGTTTTGCGCACACCGAAGGGTTGGTCAGGACCTAAAGAGCTC
CACGGGTCATTCATAGAGGGATCTTTCCACTCACATCAGGTTCCTCTACCTAATGCAAAGAAGGATAAAGAGGAGCTTCAGGCTCTGCAGAAATGGCTGTCCTCGTATAA
TCCGCACGAACTTTTCACTGAGACGGGAGACATCATTGACGACATCAAGTCAGTGATCCCTCTGGAgGACACCAAGAAGCTTGGGCAGCGAGCAGAAGCCTACAAGGGCT
ATAGGGCACCCGATCTCCCAGACTGGCGCAAGTTTGGCGTAGAAAAGGGCTCCCAGCAGAGCGCTATGAAAACAATTGGAAAGTTCATTGACCAAGTGTTTACCCAAAA
TCCTCATGGCGTCCGTGTATTTTCGCCAGACGAGCTAGAGAGCAACAAGCTGGATGCAGCACTGGCGCACACGGGAAGGAACTTTCAGTGGGATCAATTCTCGAATGCCA
AAGGCGGCCGCGTCATCGAGGTGCTCAGTGAGCACCTGTGCCAGGGCTTTATGCAGGGATACACGTTGACGGGCCGGGTGGGCATTTTCCCATCGTACGAAAGCTTCTTG
GGAATCATCCATACCATGATGGTGCAATATGCCAAATTTAACAAAATGGCTCAAGAGACGACCTGGCATAAGCCGGTTAGTAGCATCAACTATATCGAAACGAGTACGTG
GGCTCGTCAGGAGCACAATGGATTCTCTCACCAGAACCCCTCCTTTATCGGAGCTGTGCTCAGGCTGAAGCCCACCGCCGCGCGAGTTTATCTGCCACCTGATGCTAACAC
ATTTTTGACCACCCTTCACCACTGTCTCAAGTCCAAGAATTATGTCAACCTCATGGTAGGTTCAAAACAGCCAACTCCCGTGTACTTGAGCCCCGAGGAAGCAGAGAGCC
ACTGCCGAGCCGGCGCATCGATCTGGAGATTCTGTAGTACCGACAATGGGCTGAACCCGGATGTCGTGCTGGTTGGCATTGGAGTAGAGGTGATGTTCGAGGTCATCTAC
GCGGCGGCCATCCTCCGCAAGCGTTGTCCAGAACTCCGGGTGCGTGTGGTCAATGTGACCGACTTGATGATTCTGGAGAAGGAAGGTCTACATCCACATGCATTGACGAC
CGAAGCTTTCGACAGTCTGTTTGGCTCGGACCGGCCGATACACTTCAACTACCACGGATACCCGGGCGAGCTCAAAGGTCTGCTCTTTGGGCGGCCCCGCCTGGACCGAG
TTTCAGTAGAAGGATACATGGAGGAAGGAAGCACGACGACGCCGTTCGATATGATGTTGCTGAACCGCGTCTCACGATACCACGTGGCGCAGGCAGCCGTGATCGGGGC
GTCCAGACGGAATGAGAAGGTTCAAGTTCGGCAGCACGAACTGGTCAGCGAATTCGGCCACAACATCGTGGAGACACGCAAATACATTCTGGCCAACCGCAAAGACCCG
GATGATACGTATGATATGCCCTCCTTTGAAcatcatcaccaccaccatTAAGCTTGATCCACTTAACGTTACTGAAATCATCAAACAGCTTGACGAATCTGGATATAAGATCGTTGGT
GTCGATGTCAGCTCCGGAGTTGAGACAAATGGTGTTCAGGATCTCGATAAGATACGTTCATTTGTCCAAGCAGCAAAGAGTGCCTTCTAGTGATTTAATAGCTCCATGTCA
ACAAGAATAAAACGCGTTTCGGGTTTACCTCTTCCAGATACAGCTCATCTGCAATGCATTAATGCATTGGACCTCGCAACCCTAGTACGCCCTTCAGGCTCCGGCGAAGCA
GAAGAATAGCTTAGCAGAGTCTATTTTCATTTTCGGGAGACGAGATCAAGCAGATCAACGGTCGTCAAGAGTCCTACGAGACTGAGGAATCCGCTCTTGGCTCCACGCGA
CTATATATTTGTCTCTAATTGTACTTTGACATGCTCCTCTTCTTTACTCTGATAGCTTGACTATGAAAATTCCGTCACCAGCCCCTGGGTTCGCAAAGATAATTGCACTGTTT
CTTCCTTGAACTCTCAAGCCTACAGGACACACATTCATCGTAGGTATAAACCTCGAAAATCATTCCTACTAAGATGGGTATACAATAGTAACCATGCATGGTTGCCTAGTG
AATGCTCCGTAACACCCAATACGCCGGCCGAAACTTTTTTACAACTCTCCTATGAGTCGTTTACCCAGAATGCACAGGTACACTTGTTTAGAGGTAATCCTTCTTTCTAGA
AGTCCTCGTGTACTGTGTAAGCGCCCACTCCACATCTCCACTCGCTCCGGGGAGACGGGCACAAGGTTCATGAACCGAAGAGGATGGGAACCCCGACCATGGCCTGCGGC
CGCAGCTATCAGTCCTCTCAGCTGACGCCCCTCACGAACCCTCGCACTCTCCCGAAATGCTCTTCGGTGTCCATGGCGCATCATCTCAGCAGCATCCCATGTCAAACCATG
GCTTTGGGCCCACGGACTCTGTGGCCCTGCCGCAACATCACCATCACCATCGACTCCCGCCCCATGCAGCGCTGCGCGCCCCAGGGCGTCATGGAGTGAATGTGGAATCT
CCTCCTTTGCCCTCGGGCCCGCCGAGTGTGGTGGGCCAGCCTGGAATGCCTGATCCAGCCCCCAGGCCTCGAGGACCAAAACTGAAGTTTACTCCCGAAGAGGACGCTCT
ACTGGTGGAGTTGAAGGAAAACAAGAACTTGACGTGGAAGCAAATTGCAGACTTCTTCCCGGGCCGAACGAGCGGTACCTTGCAAGTCCGATACTGCACCAAGCTGAAG
GCTAAGGATGTAGCTTGGAGTGACGAAATGGTTCGATTTGCTCCTGATGTATTTCTACGCCTGTCTCACACATGCTAATGAAGGGAATAGGTACAAAGGCTGCAGCGGGC
AATGCACGAGTACGAGAACGATCGGTGGCGCATCATTGCAGGGAAGGTTGGAAATGGCTTCACCCCAGCTGCTTGCCGCGAGAAAGCCATGCAGCTCCATGAGTAAAAG
CGTTGGGGAATTTTCATATTTATATCTACTGTCGCCAGATTCGGCCCTGCTTGGACCCTCTGATCTCCTTACTCTCCATATTGGTTCAAATGTCGGGTCACCGATAGGGCTG
GTGGTGCAGGCTTGTTGTAGGCACGGGAGGATGATCAGCATAACTCTGAGTCACTATAGGGACGGGTTGATGTAAGGTATTAAGTGATGTATGATAATTCATTTTAGCCC
GGGGGAACATATGGCGCCGGCATTTGTTCGTTCGCAATGAACCGACACTAGCGTCCGCTCTCGCAGTTTAGCACCGGCTGATCCCGGGCTGAACGCGGCCATTGCTCGGC
CGGGGCATGTGTTCCTTATCTACGGCAGACCGCAGATGACCACTGGAGCAGATTATAGACCCTAAGCCCTAAGCCGGACACCCAATCGAGTAGGTCTGCGGACCAGGTCA
CTGCGGGCAGCCGGAGAAGCTCCGCAACCAATCAATCCCCGGCGCTGACTAAGGGCAGGCGACCACGGGCCGAAGCGGCTTCAAACTCACCTCAACCTCCAAACTCCCT
CATCTCCAAACGTCCTTGCCTTGTCTGCCGTCATTGCAACCCACCCACCAGGACACATGAGCAGCAAGTCCCAGCTCACCTACACTGCCCGTGCCAGCAAGCATCCCAATG
CTCTGGCGAAGAGGCTGTTCGAGATTGCCGAGGCCAAGAAaACCAATGTGACTGTCTCGGCTGACGTTACCACCACTAAGGAGCTACTAGATCTTGCTGACCGTCTCGGTC
CCTACATTGCCGTGATCAAAACCCACATCGATATCCTCTCTGATTTCAGCAACGAGACtATTGAGGGACTTAAGGCTCTCGCGCAGAAGCACAACTTTCTCATCTTCGAGG
ACCGCAAGTTCATTGACATCGGCAACACGGTCCAGAAGCAATACCACGGCGGTACCCTCCGTATCTCGGAATGGGCCCACATCATCAACTGCAGCATTCTCCCTGGTGAG
GGTATCGTCGAGGCTCTCGCTCAGACGGCGTCTGCACCGGACTTCGCCTACGGCCCCGAACGCGGTCTGTTGATCTTGGCAGAGATGACCTCTAAGGGCTCCTTGGCTACC
GGCCAGTACACTACTTCCTCGGTCGATTATGCCCGGAAATACAAGAACTTCGTTATGGGATTCGTGTCGACGCGCGCGTTGGGTGAGGTGCAGTCGGAAGTCAGCTCTCCT
TCGGATGAGGAGGACTTTGTGGTtTTCACGACTGGTGTGAACATTAACCTCCA

>pSF527_IC
TGGAGGaAGGGATCCGCCAAGGATGCTCTGGATCCGCATCCGGCCGCTCTTGCGCCCCATCAATCGCCCGACTATAAATCGAACTACTTTCGGCATCTTCTAGACTTCCTA
ATACCGCCTAGTCATAGCAGATTCAAGCTGAGAACACCACAAGTAAATATCACCCATCATGCTTACCCTGACCGTCCCTGAAAACTACGGGTATGTGCCAATTCTACAATT
CCTTGCAGACAATGCCATTCTCCCCATGAAGTCTGATGCTAACTATCCTGCAGCTCTGTCATTGCCGTCGCTCTGGGTGCCATCCCCGTCCTGAGCTTCGTCCATGGCGCCG
TCGTGTCTCGTCTCCGCAAGGAAGCTGATTGCCCCTACCCTCACTGCTATGCGACCGTAGAGCAGTGCAAGACCAACGTAAGCCAACCTCACACAAACAGGATTCCTCGA
GCTAACATACATTCCGAACCGTGCAGCCCAAGGCCGAGCAGTTCAACTGCGCTCAGCGCGCTCATGCCAACTTCCTTGAGAACTCCAGCCAAACTATGCTCTTCCTCCTGG
TAGCTGGACTGAAGTACCCCCAGTTGGCGACTGGCCTCGGAAGCATCTGGGTCCTCGGTCGCTCACTGTTCCTTTACGGATATGTGTACTCCGGCAAGCCGCGGGGTCGCG

GTCGTTTGTACGGCAGCTTCTACTTGCTTGCACAGGGAGCTCTCTGGGGCTTGACGTCTTTTGGAGTTGCGAGGGAGTTGATTTCCTACTTCTAAGTTTGGACTGAATCCGT
GGTGTGATTGAGGTGATTGGCGATGTTTGGCTATACCAGCTATATGTAATAATCTCTACTGTATACTACTATTCAACGCATTTTACTATGCGTGCTGCTAGGGTCGGCAATG
ACAATGGCAATCTGACTGACGTGGTCTATTTCTCCATGTGCAGCAGGGAATACGAGCTCCAATGGACCTCGGGAGTGGCACAGTCAATGGCAAGGAAACTGGAGcgcaaaaaa
ccccgcccctgacagggcggggtttttcgcGATCGGAGTGTGCTGTGGTTAGGGGGGGGAAAGGGAGGATTATTGAGTGCCTATTTTTGGGATCAATATGACTGACTATGGTCATGGCTAG
GACTATAGATTTGGTGGTGTAAGATTTCATTGTTCATATTGGATGGGATTGGCATGGAATGAAAGAACTGAAATGAGGGGAGAGAGATTCTAGACAAGTATCTATTATGT
ATCTAGTGGATGGATCGAGGTCTAGACGTAGTGAGTATACTAAGTGGACTAGGGACAAGGTAACTATGTAGTAGGTAGTGATGAGTGTATTCCAGAAGTACTGACTGCAG
GCGCTGTCAGCACCCACACGCACACGCACACACACTAAATGCACCACTTCCACAATACCCTAATGGAGTGACTACGATGGATGGCAGATGATGGCAGATGGCAGTGGAT
TCCGTCTCGTGGCGTCTCCAGGAACTTCTTCTCCACCTGCGCCCAACTTCCAGCACAACTTGGAAAAGGCGAAAAAGAAAAGAAAAAGAAAAAAAGAAAAATGCAATTC
AATGGCTCGCCAGGGACACTCCAATCCACGTCAGTGGCCATGTGGACCCCGAAGCCAAACAAACTTTTTACCCCTGCGCCTCTGGCGGCGATTCGTTGAAACGGCCAGGA
ACAGCAGGAACAGCAGTATGAGCACTAGCCGATCTCTTCGCCGACGGTTGGATGCCTTGCCGCCGCAAAAAGCTATATAAGGATGCATCTCGCTGCACCTTTTGCTTCCTC
TTCAATCCATCAGCAAATCCACTGACACTCTCTTGATCAGCTTTGTCTGACCTGTCATTCTCTCTCTTTTTTGATACATCTGATTTTAGTCTGTCGATCAGACTAGTCTGCCT
TCCTTTTAGTCCTCTCTCTTTTGGACTTTACTACTACACATACAACAAACCACTTTCTCTTTTTCAGAGGAACAACAACAGTCTTTCTTGACAACCCAAAATCTCATCTTTTT
TGAGTCAATCTCTTCAAACAAATCTCATCAAACATGCCATCGGATTCGAATGATCAGAGCATCTCCGCTTATGGAGCTGCTCGCTCCACGGTCAAGGGCCAGAACCTTGAC
CCGGAAGAAGTCCGGAAAATGGACGCCTACTTTCGCGCCAGCATGTACCTATGCTTAGGCATGCTCTACCTGCGCGAAAATGTGCTTCTGAAACAGCCATTGAAGGTGGA
GCATCTCAAAGCACGCCTACTCGGCCACTGGGGCTCAGACGCAGGACAATCCTTCACCTGGATTCACATGAACCGGCTGATCAAGAAGTATGATCTGGACGTTCTCTTCA
TCTCAGGCCCGGGTCACGGTGCCCCTGGCATTCTTTCACAGTCCTACCTGGAGGGTGTATACTCCGAAGTCTACCCGGACAAGTCGGAGGACGAGCGAGGAATGCAAAGG
TTCTTCAAGCAGTTTTCTTTCCCCGGCGGTATCGGCAGCCACGCTACCCCAGAAACCCCCGGTAGTCTcCACGAGGGCGGGGAGCTGGGTTATTCCATCTCGCACGCCTTT
GGCACCGTgTTCGATCATCCCAATCTGATTACCCTTACCATGGTGGGCGATGGTGAGGCCGAGACTGGGCCACTAGCTACCAGCTGGCACAGCACCAAGTACCTTAATCCC
TGCACCGACGGTGCCGTTCTGCCCGTGCTCCATCTCAACGGCTACAAGATCAACAATCCGACACTCCTCGCACGCATCTCCCATGATGAGCTTTCTGCTCTCATGAAGGGT
TACGGCTGGACCCCCTACTTTGTAGAGGGCAGTGACCGCGAAACCATGCATCAAGCCATGGCAGCCACGCTCGAGCACTGTGTTCTTGAAATCAGGAAATTCCAGAAGAA
AGCCCGCGAGTCGAAGGAGCCCTTCCGCCCACACTGGCCCATGATCATACTGCGCAGCCCAAAGGGTTGGTCCGCCCCGCGTGAAGTCGATGGCAAACTCCTTGAGGGCT
TTTGGCGTGCCCACCAAATCCCTATCACCGACGTCCTCACCAATCCTTCACATCTTCAACTTCTTGAATCGTGGATGAAGAGCTATAAGCCCGAGGAGCTCTTCACTCACG
ACGGCCGTTTGATCTCCGAACTCAAAGCCCTCGCTCCAACTGGCAATTCCCGTATGAGTGCCAATCCGGTGGGCAATGGTGGGCTGCTACGGCGACCGCTGGACCTACCC
GACTTCCGCAAGTACGCACTCACATCCATCGATCCGGGAGCCACCATCCGCGGCAGCATGGTCAACATGTCACACTACCTCCGTGATGTGGTCGCCTTCAATCAGACCAA
CTTCCGTGTATTCGGGCCGGACGAAACTGAATCCAACAAGCTCTCCGAGATCTACAAAGCCGGCAAGAAAGTCTGGCTGGCCGAATACTTCCCTGAAGATAACAACGGTG
GAAACCTTTCCATGGCCGGTCGAGTGATGGAGATGCTTAGTGAACACACATGTGAAGGCTGGCTAGAGGGCTACGTCCTCTCAGGCCGCCATGGGCTCCTTAATAGCTAC
GAGCCCTTCATCCACATCATCGACTCTATGGTGAACCAGCACTGCAAGTGGATCGAGAAATGCCTCGAAGTCGAATGGCGCGCCAAAGTGGCATCTTTGAACATCCTCCT
CACCGCGACCGTCTGGCGCCAAGACCACAACGGATTCACTCACCAAGACCCAGGCTTCCTCGATGTCGTGGCTAACAAGTCTCCCGAAGTCGTGCGCATCTACCTCCCGC
CCGACGGAAACTCTCTCCTCTCCGTAATGGACCACTGCTTCCGCAGCGCCAACTACGTTAACGTCATTGTCGCCGATAAACAAGACCACATCCAATTCATGGACATGGAC
GCAGCAATTGCCCACTGCACCAAGGGCGTCGGGATCTGGGACTGGGCCAGTAACGACCAGGGCGCGGAACCAGACGTTGTCATGGCCGCATGCGGCGACGTTCCAACCC
ACGAGGCACTAGCCGCCACAGCGCTCCTACGCGAACACCTTCCCCAGCTGAAAGTGCGCTTCGTCAACGTGGTCGATCTTTTCAAACTCATGTCCAAGATCCATCACCCGC
ATGGAATGTCCGATCGGGAATGGAAGGCTATCTTCACGGCTGATAGGCCGATTGTgTTCAATTTCCACTCGTACCCGTGGCTTATTCACCGGTTGACGTATAAGcGACCCG
GACAGGAGAATATCCATGTGAGGGGGTACAAGGAGAAGGGGAACATCGATACGCCATTCGAGCTGGCGGTGAGGAATCAGACGGATCGGTATAGTTTGGCCGTTGATGC
GATTGATCATGCCCGCGGACTGGGCAATACGGCGTCCGGAGTGAGAGAGAAGTTCCTGAATATGCAGTTATTGGCCAAGCAGAAGGCATACGATGACGGCATTGATCCG
GACTATATTCGGAACTGGACCTGGCAGTATCCGCGGAAGAAGGGGGAGGGCGTCcatcatcaccaccaccatTGAGCTTGATCCACTTAACGTTACTGAAATCATCAAACAGCTTGA
CGAATCTGGATATAAGATCGTTGGTGTCGATGTCAGCTCCGGAGTTGAGACAAATGGTGTTCAGGATCTCGATAAGATACGTTCATTTGTCCAAGCAGCAAAGAGTGCCT
TCTAGTGATTTAATAGCTCCATGTCAACAAGAATAAAACGCGTTTCGGGTTTACCTCTTCCAGATACAGCTCATCTGCAATGCATTAATGCATTGGACCTCGCAACCCTAG
TACGCCCTTCAGGCTCCGGCGAAGCAGAAGAATAGCTTAGCAGAGTCTATTTTCATTTTCGGGAGACGAGATCAAGCAGATCAACGGTCGTCAAGAGTCCTACGAGACTG
AGGAATCCGCTCTTGGCTCCACGCGACTATATATTTGTCTCTAATTGTACTTTGACATGCTCCTCTTCTTTACTCTGATAGCTTGACTATGAAAATTCCGTCACCAGCCCCTG
GGTTCGCAAAGATAATTGCACTGTTTCTTCCTTGAACTCTCAAGCCTACAGGACACACATTCATCGTAGGTATAAACCTCGAAAATCATTCCTACTAAGATGGGTATACAA
TAGTAACCATGCATGGTTGCCTAGTGAATGCTCCGTAACACCCAATACGCCGGCCGAAACTTTTTTACAACTCTCCTATGAGTCGTTTACCCAGAATGCACAGGTACACTT
GTTTAGAGGTAATCCTTCTTTCTAGAAGTCCTCGTGTACTGTGTAAGCGCCCACTCCACATCTCCACTCGCTCCGGGGAGACGGGCACAAGGTTCATGAACCGAAGAGGAT
GGGAACCCCGACCATGGCCTGCGGCCGCAGCTATCAGTCCTCTCAGCTGACGCCCCTCACGAACCCTCGCACTCTCCCGAAATGCTCTTCGGTGTCCATGGCGCATCATCT
CAGCAGCATCCCATGTCAAACCATGGCTTTGGGCCCACGGACTCTGTGGCCCTGCCGCAACATCACCATCACCATCGACTCCCGCCCCATGCAGCGCTGCGCGCCCCAGG
GCGTCATGGAGTGAATGTGGAATCTCCTCCTTTGCCCTCGGGCCCGCCGAGTGTGGTGGGCCAGCCTGGAATGCCTGATCCAGCCCCCAGGCCTCGAGGACCAAAACTGA
AGTTTACTCCCGAAGAGGACGCTCTACTGGTGGAGTTGAAGGAAAACAAGAACTTGACGTGGAAGCAAATTGCAGACTTCTTCCCGGGCCGAACGAGCGGTACCTTGCA
AGTCCGATACTGCACCAAGCTGAAGGCTAAGGATGTAGCTTGGAGTGACGAAATGGTTCGATTTGCTCCTGATGTATTTCTACGCCTGTCTCACACATGCTAATGAAGGG
AATAGGTACAAAGGCTGCAGCGGGCAATGCACGAGTACGAGAACGATCGGTGGCGCATCATTGCAGGGAAGGTTGGAAATGGCTTCACCCCAGCTGCTTGCCGCGAGAA
AGCCATGCAGCTCCATGAGTAAAAGCGTTGGGGAATTTTCATATTTATATCTACTGTCGCCAGATTCGGCCCTGCTTGGACCCTCTGATCTCCTTACTCTCCATATTGGTTC
AAATGTCGGGTCACCGATAGGGCTGGTGGTGCAGGCTTGTTGTAGGCACGGGAGGATGATCAGCATAACTCTGAGTCACTATAGGGACGGGTTGATGTAAGGTATTAAGT
GATGTATGATAATTCATTTTAGCCCGGGGGAACATATGGCGCCGGCATTTGTTCGTTCGCAATGAACCGACACTAGCGTCCGCTCTCGCAGTTTAGCACCGGCTGATCCCG
GGCTGAACGCGGCCATTGCTCGGCCGGGGCATGTGTTCCTTATCTACGGCAGACCGCAGATGACCACTGGAGCAGATTATAGACCCTAAGCCCTAAGCCGGACACCCAAT
CGAGTAGGTCTGCGGACCAGGTCACTGCGGGCAGCCGGAGAAGCTCCGCAACCAATCAATCCCCGGCGCTGACTAAGGGCAGGCGACCACGGGCCGAAGCGGCTTCAAA
CTCACCTCAACCTCCAAACTCCCTCATCTCCAAACGTCCTTGCCTTGTCTGCCGTCATTGCAACCCACCCACCAGGACACATGAGCAGCAAGTCCCAGCTCACCTACACTG
CCCGTGCCAGCAAGCATCCCAATGCTCTGGCGAAGAGGCTGTTCGAGATTGCCGAGGCCAAGAAaACCAATGTGACTGTCTCGGCTGACGTTACCACCACTAAGGAGCTA
CTAGATCTTGCTGACCGTCTCGGTCCCTACATTGCCGTGATCAAAACCCACATCGATATCCTCTCTGATTTCAGCAACGAGACtATTGAGGGACTTAAGGCTCTCGCGCAGA
AGCACAACTTTCTCATCTTCGAGGACCGCAAGTTCATTGACATCGGCAACACGGTCCAGAAGCAATACCACGGCGGTACCCTCCGTATCTCGGAATGGGCCCACATCATC
AACTGCAGCATTCTCCCTGGTGAGGGTATCGTCGAGGCTCTCGCTCAGACGGCGTCTGCACCGGACTTCGCCTACGGCCCCGAACGCGGTCTGTTGATCTTGGCAGAGATG
ACCTCTAAGGGCTCCTTGGCTACCGGCCAGTACACTACTTCCTCGGTCGATTATGCCCGGAAATACAAGAACTTCGTTATGGGATTCGTGTCGACGCGCGCGTTGGGTGAG
GTGCAGTCGGAAGTCAGCTCTCCTTCGGATGAGGAGGACTTTGTGGTtTTCACGACTGGTGTGAACATTAACCTCCA

>pSF526_IC
TGGAGGaAGGGATCCGCCAAGGATGCTCTGGATCCGCATCCGGCCGCTCTTGCGCCCCATCAATCGCCCGACTATAAATCGAACTACTTTCGGCATCTTCTAGACTTCCTA
ATACCGCCTAGTCATAGCAGATTCAAGCTGAGAACACCACAAGTAAATATCACCCATCATGCTTACCCTGACCGTCCCTGAAAACTACGGGTATGTGCCAATTCTACAATT
CCTTGCAGACAATGCCATTCTCCCCATGAAGTCTGATGCTAACTATCCTGCAGCTCTGTCATTGCCGTCGCTCTGGGTGCCATCCCCGTCCTGAGCTTCGTCCATGGCGCCG
TCGTGTCTCGTCTCCGCAAGGAAGCTGATTGCCCCTACCCTCACTGCTATGCGACCGTAGAGCAGTGCAAGACCAACGTAAGCCAACCTCACACAAACAGGATTCCTCGA
GCTAACATACATTCCGAACCGTGCAGCCCAAGGCCGAGCAGTTCAACTGCGCTCAGCGCGCTCATGCCAACTTCCTTGAGAACTCCAGCCAAACTATGCTCTTCCTCCTGG
TAGCTGGACTGAAGTACCCCCAGTTGGCGACTGGCCTCGGAAGCATCTGGGTCCTCGGTCGCTCACTGTTCCTTTACGGATATGTGTACTCCGGCAAGCCGCGGGGTCGCG
GTCGTTTGTACGGCAGCTTCTACTTGCTTGCACAGGGAGCTCTCTGGGGCTTGACGTCTTTTGGAGTTGCGAGGGAGTTGATTTCCTACTTCTAAGTTTGGACTGAATCCGT
GGTGTGATTGAGGTGATTGGCGATGTTTGGCTATACCAGCTATATGTAATAATCTCTACTGTATACTACTATTCAACGCATTTTACTATGCGTGCTGCTAGGGTCGGCAATG
ACAATGGCAATCTGACTGACGTGGTCTATTTCTCCATGTGCAGCAGGGAATACGAGCTCCAATGGACCTCGGGAGTGGCACAGTCAATGGCAAGGAAACTGGAGcgcaaaaaa
ccccgcccctgacagggcggggtttttcgcGATCGGAGCCTCTCGTATGCAGAGGAAATCTCCCCTGATCTTCCGAACTGGTCGTACCTGGCGACCTATGACTATGGCACCCCAGTTCTGG
GGACCTTCCACGGAAGTGACCTGCTGCAGGTGTTCTATGGGATCAAGCCAAACTATGCAGCTAGTTCTAGCCACACGTACTATCTGAGCTTTGTGTATACGCTGGATCCGA
ACTCCAACCGGGGGGAGTACATTGAGTGGCCGCAGTGGAAGGAATCGCGGCAGTTGATGAATTTCGGAGCGAACGACGCCAGTCTCCTTACGGATGATTTCCGCAACGG
GACATATGAGTTCATCCTGCAGAATACCGCGGCGTTCCACATCTGATGCCATTGGCGGAGGGGTCCGGACGGTCAGGAACTTAGCCTTATGAGATGAATGATGGACGTGT
CTGGCCTCGGAAAAGGATATATGGGGATCATAATAGTACTAGCCATATTAATGAAGGGCATATACCACGCGTTGGACCTGCGTTATAGCTTCCCGTTAGTTATAGTACCAT
CGTTATACCAGCCAATCAAGTCACCACGCACGACCGGGGACGGCGAATCCCCGGGAATTGAAAGAAATTGCATCCCAGGCCAGTGAGGCCAGCGATTGGCCACCTCTCC
AAGGCACAGGGCCATTCTGCAGCGCTGGTGGATTCATCGCAATTTCCCCCGGCCCGGCCCGACACCGCTATAGGCTGGTTCTCCCACACCATCGGAGATTCGTCGCCTAAT
GTCTCGTCCGTTCACAAGCTGAAGAGCTTGAAGTGGCGAGATGTCTCTGCAGGAATTCAAGCTAGATGCTAAGCGATATTGCATGGCAATATGTGTTGATGCATGTGCTTC
TTCCTTCAGCTTCCCCTCGTGCAGATGAGGTTTGGCTATAAATTGAAGTGGTTGGTCGGGGTTCCGTGAGGGGCTGAAGTGCTTCCTCCCTTTTAGACGCAACTGAGAGCC
TGAGCTTCATCCCCAGCATCATTACACCTCAGCACATGCCATCGGATTCGAATGATCAGAGCATCTCCGCTTATGGAGCTGCTCGCTCCACGGTCAAGGGCCAGAACCTTG
ACCCGGAAGAAGTCCGGAAAATGGACGCCTACTTTCGCGCCAGCATGTACCTATGCTTAGGCATGCTCTACCTGCGCGAAAATGTGCTTCTGAAACAGCCATTGAAGGTG
GAGCATCTCAAAGCACGCCTACTCGGCCACTGGGGCTCAGACGCAGGACAATCCTTCACCTGGATTCACATGAACCGGCTGATCAAGAAGTATGATCTGGACGTTCTCTT
CATCTCAGGCCCGGGTCACGGTGCCCCTGGCATTCTTTCACAGTCCTACCTGGAGGGTGTATACTCCGAAGTCTACCCGGACAAGTCGGAGGACGAGCGAGGAATGCAAA
GGTTCTTCAAGCAGTTTTCTTTCCCCGGCGGTATCGGCAGCCACGCTACCCCAGAAACCCCCGGTAGTCTcCACGAGGGCGGGGAGCTGGGTTATTCCATCTCGCACGCCT
TTGGCACCGTgTTCGATCATCCCAATCTGATTACCCTTACCATGGTGGGCGATGGTGAGGCCGAGACTGGGCCACTAGCTACCAGCTGGCACAGCACCAAGTACCTTAATC
CCTGCACCGACGGTGCCGTTCTGCCCGTGCTCCATCTCAACGGCTACAAGATCAACAATCCGACACTCCTCGCACGCATCTCCCATGATGAGCTTTCTGCTCTCATGAAGG
GTTACGGCTGGACCCCCTACTTTGTAGAGGGCAGTGACCGCGAAACCATGCATCAAGCCATGGCAGCCACGCTCGAGCACTGTGTTCTTGAAATCAGGAAATTCCAGAAG
AAAGCCCGCGAGTCGAAGGAGCCCTTCCGCCCACACTGGCCCATGATCATACTGCGCAGCCCAAAGGGTTGGTCCGCCCCGCGTGAAGTCGATGGCAAACTCCTTGAGGG
CTTTTGGCGTGCCCACCAAATCCCTATCACCGACGTCCTCACCAATCCTTCACATCTTCAACTTCTTGAATCGTGGATGAAGAGCTATAAGCCCGAGGAGCTCTTCACTCA
CGACGGCCGTTTGATCTCCGAACTCAAAGCCCTCGCTCCAACTGGCAATTCCCGTATGAGTGCCAATCCGGTGGGCAATGGTGGGCTGCTACGGCGACCGCTGGACCTAC
CCGACTTCCGCAAGTACGCACTCACATCCATCGATCCGGGAGCCACCATCCGCGGCAGCATGGTCAACATGTCACACTACCTCCGTGATGTGGTCGCCTTCAATCAGACC
AACTTCCGTGTATTCGGGCCGGACGAAACTGAATCCAACAAGCTCTCCGAGATCTACAAAGCCGGCAAGAAAGTCTGGCTGGCCGAATACTTCCCTGAAGATAACAACG
GTGGAAACCTTTCCATGGCCGGTCGAGTGATGGAGATGCTTAGTGAACACACATGTGAAGGCTGGCTAGAGGGCTACGTCCTCTCAGGCCGCCATGGGCTCCTTAATAGC
TACGAGCCCTTCATCCACATCATCGACTCTATGGTGAACCAGCACTGCAAGTGGATCGAGAAATGCCTCGAAGTCGAATGGCGCGCCAAAGTGGCATCTTTGAACATCCT
CCTCACCGCGACCGTCTGGCGCCAAGACCACAACGGATTCACTCACCAAGACCCAGGCTTCCTCGATGTCGTGGCTAACAAGTCTCCCGAAGTCGTGCGCATCTACCTCCC
GCCCGACGGAAACTCTCTCCTCTCCGTAATGGACCACTGCTTCCGCAGCGCCAACTACGTTAACGTCATTGTCGCCGATAAACAAGACCACATCCAATTCATGGACATGG
ACGCAGCAATTGCCCACTGCACCAAGGGCGTCGGGATCTGGGACTGGGCCAGTAACGACCAGGGCGCGGAACCAGACGTTGTCATGGCCGCATGCGGCGACGTTCCAAC
CCACGAGGCACTAGCCGCCACAGCGCTCCTACGCGAACACCTTCCCCAGCTGAAAGTGCGCTTCGTCAACGTGGTCGATCTTTTCAAACTCATGTCCAAGATCCATCACCC
GCATGGAATGTCCGATCGGGAATGGAAGGCTATCTTCACGGCTGATAGGCCGATTGTgTTCAATTTCCACTCGTACCCGTGGCTTATTCACCGGTTGACGTATAAGcGACC
CGGACAGGAGAATATCCATGTGAGGGGGTACAAGGAGAAGGGGAACATCGATACGCCATTCGAGCTGGCGGTGAGGAATCAGACGGATCGGTATAGTTTGGCCGTTGAT
GCGATTGATCATGCCCGCGGACTGGGCAATACGGCGTCCGGAGTGAGAGAGAAGTTCCTGAATATGCAGTTATTGGCCAAGCAGAAGGCATACGATGACGGCATTGATC
CGGACTATATTCGGAACTGGACCTGGCAGTATCCGCGGAAGAAGGGGGAGGGCGTCcatcatcaccaccaccatTGAGCTTGATCCACTTAACGTTACTGAAATCATCAAACAGCTT

GACGAATCTGGATATAAGATCGTTGGTGTCGATGTCAGCTCCGGAGTTGAGACAAATGGTGTTCAGGATCTCGATAAGATACGTTCATTTGTCCAAGCAGCAAAGAGTGC
CTTCTAGTGATTTAATAGCTCCATGTCAACAAGAATAAAACGCGTTTCGGGTTTACCTCTTCCAGATACAGCTCATCTGCAATGCATTAATGCATTGGACCTCGCAACCCT
AGTACGCCCTTCAGGCTCCGGCGAAGCAGAAGAATAGCTTAGCAGAGTCTATTTTCATTTTCGGGAGACGAGATCAAGCAGATCAACGGTCGTCAAGAGTCCTACGAGAC
TGAGGAATCCGCTCTTGGCTCCACGCGACTATATATTTGTCTCTAATTGTACTTTGACATGCTCCTCTTCTTTACTCTGATAGCTTGACTATGAAAATTCCGTCACCAGCCCC
TGGGTTCGCAAAGATAATTGCACTGTTTCTTCCTTGAACTCTCAAGCCTACAGGACACACATTCATCGTAGGTATAAACCTCGAAAATCATTCCTACTAAGATGGGTATAC
AATAGTAACCATGCATGGTTGCCTAGTGAATGCTCCGTAACACCCAATACGCCGGCCGAAACTTTTTTACAACTCTCCTATGAGTCGTTTACCCAGAATGCACAGGTACAC
TTGTTTAGAGGTAATCCTTCTTTCTAGAAGTCCTCGTGTACTGTGTAAGCGCCCACTCCACATCTCCACTCGCTCCGGGGAGACGGGCACAAGGTTCATGAACCGAAGAGG
ATGGGAACCCCGACCATGGCCTGCGGCCGCAGCTATCAGTCCTCTCAGCTGACGCCCCTCACGAACCCTCGCACTCTCCCGAAATGCTCTTCGGTGTCCATGGCGCATCAT
CTCAGCAGCATCCCATGTCAAACCATGGCTTTGGGCCCACGGACTCTGTGGCCCTGCCGCAACATCACCATCACCATCGACTCCCGCCCCATGCAGCGCTGCGCGCCCCAG
GGCGTCATGGAGTGAATGTGGAATCTCCTCCTTTGCCCTCGGGCCCGCCGAGTGTGGTGGGCCAGCCTGGAATGCCTGATCCAGCCCCCAGGCCTCGAGGACCAAAACTG
AAGTTTACTCCCGAAGAGGACGCTCTACTGGTGGAGTTGAAGGAAAACAAGAACTTGACGTGGAAGCAAATTGCAGACTTCTTCCCGGGCCGAACGAGCGGTACCTTGC
AAGTCCGATACTGCACCAAGCTGAAGGCTAAGGATGTAGCTTGGAGTGACGAAATGGTTCGATTTGCTCCTGATGTATTTCTACGCCTGTCTCACACATGCTAATGAAGG
GAATAGGTACAAAGGCTGCAGCGGGCAATGCACGAGTACGAGAACGATCGGTGGCGCATCATTGCAGGGAAGGTTGGAAATGGCTTCACCCCAGCTGCTTGCCGCGAGA
AAGCCATGCAGCTCCATGAGTAAAAGCGTTGGGGAATTTTCATATTTATATCTACTGTCGCCAGATTCGGCCCTGCTTGGACCCTCTGATCTCCTTACTCTCCATATTGGTT
CAAATGTCGGGTCACCGATAGGGCTGGTGGTGCAGGCTTGTTGTAGGCACGGGAGGATGATCAGCATAACTCTGAGTCACTATAGGGACGGGTTGATGTAAGGTATTAAG
TGATGTATGATAATTCATTTTAGCCCGGGGGAACATATGGCGCCGGCATTTGTTCGTTCGCAATGAACCGACACTAGCGTCCGCTCTCGCAGTTTAGCACCGGCTGATCCC
GGGCTGAACGCGGCCATTGCTCGGCCGGGGCATGTGTTCCTTATCTACGGCAGACCGCAGATGACCACTGGAGCAGATTATAGACCCTAAGCCCTAAGCCGGACACCCAA
TCGAGTAGGTCTGCGGACCAGGTCACTGCGGGCAGCCGGAGAAGCTCCGCAACCAATCAATCCCCGGCGCTGACTAAGGGCAGGCGACCACGGGCCGAAGCGGCTTCAA
ACTCACCTCAACCTCCAAACTCCCTCATCTCCAAACGTCCTTGCCTTGTCTGCCGTCATTGCAACCCACCCACCAGGACACATGAGCAGCAAGTCCCAGCTCACCTACACT
GCCCGTGCCAGCAAGCATCCCAATGCTCTGGCGAAGAGGCTGTTCGAGATTGCCGAGGCCAAGAAaACCAATGTGACTGTCTCGGCTGACGTTACCACCACTAAGGAGCT
ACTAGATCTTGCTGACCGTCTCGGTCCCTACATTGCCGTGATCAAAACCCACATCGATATCCTCTCTGATTTCAGCAACGAGACtATTGAGGGACTTAAGGCTCTCGCGCAG
AAGCACAACTTTCTCATCTTCGAGGACCGCAAGTTCATTGACATCGGCAACACGGTCCAGAAGCAATACCACGGCGGTACCCTCCGTATCTCGGAATGGGCCCACATCAT
CAACTGCAGCATTCTCCCTGGTGAGGGTATCGTCGAGGCTCTCGCTCAGACGGCGTCTGCACCGGACTTCGCCTACGGCCCCGAACGCGGTCTGTTGATCTTGGCAGAGAT
GACCTCTAAGGGCTCCTTGGCTACCGGCCAGTACACTACTTCCTCGGTCGATTATGCCCGGAAATACAAGAACTTCGTTATGGGATTCGTGTCGACGCGCGCGTTGGGTGA
GGTGCAGTCGGAAGTCAGCTCTCCTTCGGATGAGGAGGACTTTGTGGTtTTCACGACTGGTGTGAACATTAACCTCCA

>pSF533_IC
TGGAGGaAGGGATCCGCCAAGGATGCTCTGGATCCGCATCCGGCCGCTCTTGCGCCCCATCAATCGCCCGACTATAAATCGAACTACTTTCGGCATCTTCTAGACTTCCTA
ATACCGCCTAGTCATAGCAGATTCAAGCTGAGAACACCACAAGTAAATATCACCCATCATGCTTACCCTGACCGTCCCTGAAAACTACGGGTATGTGCCAATTCTACAATT
CCTTGCAGACAATGCCATTCTCCCCATGAAGTCTGATGCTAACTATCCTGCAGCTCTGTCATTGCCGTCGCTCTGGGTGCCATCCCCGTCCTGAGCTTCGTCCATGGCGCCG
TCGTGTCTCGTCTCCGCAAGGAAGCTGATTGCCCCTACCCTCACTGCTATGCGACCGTAGAGCAGTGCAAGACCAACGTAAGCCAACCTCACACAAACAGGATTCCTCGA
GCTAACATACATTCCGAACCGTGCAGCCCAAGGCCGAGCAGTTCAACTGCGCTCAGCGCGCTCATGCCAACTTCCTTGAGAACTCCAGCCAAACTATGCTCTTCCTCCTGG
TAGCTGGACTGAAGTACCCCCAGTTGGCGACTGGCCTCGGAAGCATCTGGGTCCTCGGTCGCTCACTGTTCCTTTACGGATATGTGTACTCCGGCAAGCCGCGGGGTCGCG
GTCGTTTGTACGGCAGCTTCTACTTGCTTGCACAGGGAGCTCTCTGGGGCTTGACGTCTTTTGGAGTTGCGAGGGAGTTGATTTCCTACTTCTAAGTTTGGACTGAATCCGT
GGTGTGATTGAGGTGATTGGCGATGTTTGGCTATACCAGCTATATGTAATAATCTCTACTGTATACTACTATTCAACGCATTTTACTATGCGTGCTGCTAGGGTCGGCAATG
ACAATGGCAATCTGACTGACGTGGTCTATTTCTCCATGTGCAGCAGGGAATACGAGCTCCAATGGACCTCGGGAGTGGCACAGTCAATGGCAAGGAAACTGGAGcgcaaaaaa
ccccgcccctgacagggcggggtttttcgcGATCGGAGTGTGCTGTGGTTAGGGGGGGGAAAGGGAGGATTATTGAGTGCCTATTTTTGGGATCAATATGACTGACTATGGTCATGGCTAG
GACTATAGATTTGGTGGTGTAAGATTTCATTGTTCATATTGGATGGGATTGGCATGGAATGAAAGAACTGAAATGAGGGGAGAGAGATTCTAGACAAGTATCTATTATGT
ATCTAGTGGATGGATCGAGGTCTAGACGTAGTGAGTATACTAAGTGGACTAGGGACAAGGTAACTATGTAGTAGGTAGTGATGAGTGTATTCCAGAAGTACTGACTGCAG
GCGCTGTCAGCACCCACACGCACACGCACACACACTAAATGCACCACTTCCACAATACCCTAATGGAGTGACTACGATGGATGGCAGATGATGGCAGATGGCAGTGGAT
TCCGTCTCGTGGCGTCTCCAGGAACTTCTTCTCCACCTGCGCCCAACTTCCAGCACAACTTGGAAAAGGCGAAAAAGAAAAGAAAAAGAAAAAAAGAAAAATGCAATTC
AATGGCTCGCCAGGGACACTCCAATCCACGTCAGTGGCCATGTGGACCCCGAAGCCAAACAAACTTTTTACCCCTGCGCCTCTGGCGGCGATTCGTTGAAACGGCCAGGA
ACAGCAGGAACAGCAGTATGAGCACTAGCCGATCTCTTCGCCGACGGTTGGATGCCTTGCCGCCGCAAAAAGCTATATAAGGATGCATCTCGCTGCACCTTTTGCTTCCTC
TTCAATCCATCAGCAAATCCACTGACACTCTCTTGATCAGCTTTGTCTGACCTGTCATTCTCTCTCTTTTTTGATACATCTGATTTTAGTCTGTCGATCAGACTAGTCTGCCT
TCCTTTTAGTCCTCTCTCTTTTGGACTTTACTACTACACATACAACAAACCACTTTCTCTTTTTCAGAGGAACAACAACAGTCTTTCTTGACAACCCAAAATCTCATCTTTTT
TGAGTCAATCTCTTCAAACAAATCTCATCAAACATGACATCCCCTGTAATTGGTACTCCTTGGAAGAAGTTGAACGCCCCAGTCTCAGAAGAAGCATTGGAAGGTGTTGA
TAAGTATTGGAGAGTTGCAAATTACTTGTCAATTGGTCAAATTTACTTGAGATCAAATCCATTGATGAAAGAACCATTCACTAGAGAAGATGTTAAACATAGATTGGTTGG
TCATTGGGGTACAACTCCAGGTTTAAATTTCTTGATTGGTCATATTAATAGATTCATTGCTGATCATGGTCAAAATACAGTTATTATTATGGGTCCAGGTCATGGTGGTCCA
GCTGGTACTTCACAATCTTACTTGGATGGTACTTATACAGAAACTTTTCCAAAAATTACTAAAGATGAAGCAGGTTTGCAAAAATTTTTCAGACAATTTTCTTATCCAGGT
GGTATTCCATCTCATTTTGCACCAGAAACACCAGGTTCAATTCATGAAGGTGGTGAATTGGGTTACGCTTTGTCTCATGCTTATGGTGCAATTATGGATAATCCATCTTTGT
TTGTTCCAGCTATTGTTGGTGACGGTGAAGCTGAAACTGGTCCATTGGCTACTGGTTGGCAATCAAATAAGTTGGTTAATCCAAGAACTGATGGTATTGTTTTACCAATTTT
GCATTTGAATGGTTACAAAATTGCTAATCCAACAATTTTATCAAGAATTTCTGATGAAGAATTGCATGAATTTTTCCATGGTATGGGTTACGAACCATACGAATTTGTTGC
AGGTTTTGATGATGAAGATCATATGTCAATTCATAGAAGATTTGCTGAATTATGGGAAACAATTTGGGATGAAATTTGTGATATTAAAGCAACAGCTCAAACTGATAATG
TTCATAGACCATTTTATCCAATGTTGATTTTTAGAACACCAAAAGGTTGGACTTGTCCAAAATATATTGATGGTAAAAAGACTGAAGGTTCATGGAGATCACATCAAGTTC
CATTAGCATCTGCTAGAGATACTGAAGCACATTTTGAAGTTTTGAAAAATTGGTTGGAATCTTATAAACCAGAAGAATTGTTTGATGCAAATGGTGCAGTTAAAGATGAT
GTTTTAGCTTTTATGCCAAAAGGTGAATTGAGAATTGGTGCTAATCCAAATGCTAATGGTGGTGTTATTAGAAATGATTTGAAATTACCAAATTTGGAAGATTACGAAGTT
AAAGAAGTTGCAGAATACGGTCATGGTTGGGGTCAATTGGAAGCTACTAGAACTTTAGGTGCTTATACTAGAGATATTATTAAAAATAATCCAAGAGATTTTAGAATTTTT
GGTCCAGATGAAACTGCTTCTAATAGATTGCAAGCATCTTATGAAGTTACTAATAAGCAATGGGATGCTGGTTATATTTCAGATGAAGTTGATGAACATATGCATGTTTCA
GGTCAAGTTGTTGAACAATTGTCAGAACATCAAATGGAAGGTTTCTTGGAAGCATACTTGTTAACAGGTAGACATGGTATTTGGTCATCTTATGAATCTTTTGTTCATGTTA
TTGATTCAATGTTAAATCAACATGCAAAATGGTTGGAAGCTACAGTTAGAGAAATTCCATGGAGAAAACCAATTGCATCTATGAATTTGTTAGTTTCTTCACATGTTTGGA
GACAAGATCATAATGGTTTTTCACATCAAGATCCAGGTGTTACTTCTGTTTTGTTGAATAAGTGTTTTCATAATGATCATGTTATTGGTATTTACTTTGCAACTGATGCTAAT
ATGTTGTTAGCTATTGCTGAAAAATGTTACAAATCAACTAATAAGATTAATGCAATTATTGCTGGTAAACAACCAGCAGCTACTTGGTTAACATTGGATGAAGCTAGAGC
AGAATTAGAAAAAGGTGCAGCTGCTTGGGATTGGGCATCTACTGCTAAAAATAATGATGAAGCTGAAGTTGTTTTAGCAGCAGCTGGTGACGTTCCAACTCAAGAAATTA
TGGCAGCTTCAGATAAATTGAAAGAATTGGGTATTAAATTCAAAGTTGTTAATGTTGCAGATTTGTTATCATTGCAATCTGCTAAAGAAAATGATGAAGCATTAACTGATG
AAGAATTTGCTGATATTTTTACAGCTGATAAACCAGTTTTATTTGCTTACCATTCTTATGCTCATGATGTTAGAGGTTTGATTTACGATAGACCAAATCATGATAATTTTAA
TGTTCATGGTTATGAAGAAGAAGGTTCAACTACAACTCCATACGATATGGTTAGAGTTAATAGAATTGATAGATACGAATTGACTGCTGAAGCATTGAGAATGATTGATG
CAGATAAATACGCAGATAAAATTGATGAATTGGAAAAATTCAGAGATGAAGCATTTCAATTTGCAGTTGATAATGGTTATGATCATCCAGATTATACAGATTGGGTTTAC
TCAGGTGTAAATACCGACAAAAAGGGTGCTGTAACCGCTACCGCTGCTACCGCTGGTGACAACGAAcatcatcaccaccaccatTAAGCTTGATCCACTTAACGTTACTGAAATCAT
CAAACAGCTTGACGAATCTGGATATAAGATCGTTGGTGTCGATGTCAGCTCCGGAGTTGAGACAAATGGTGTTCAGGATCTCGATAAGATACGTTCATTTGTCCAAGCAG
CAAAGAGTGCCTTCTAGTGATTTAATAGCTCCATGTCAACAAGAATAAAACGCGTTTCGGGTTTACCTCTTCCAGATACAGCTCATCTGCAATGCATTAATGCATTGGACC
TCGCAACCCTAGTACGCCCTTCAGGCTCCGGCGAAGCAGAAGAATAGCTTAGCAGAGTCTATTTTCATTTTCGGGAGACGAGATCAAGCAGATCAACGGTCGTCAAGAGT
CCTACGAGACTGAGGAATCCGCTCTTGGCTCCACGCGACTATATATTTGTCTCTAATTGTACTTTGACATGCTCCTCTTCTTTACTCTGATAGCTTGACTATGAAAATTCCG
TCACCAGCCCCTGGGTTCGCAAAGATAATTGCACTGTTTCTTCCTTGAACTCTCAAGCCTACAGGACACACATTCATCGTAGGTATAAACCTCGAAAATCATTCCTACTAA
GATGGGTATACAATAGTAACCATGCATGGTTGCCTAGTGAATGCTCCGTAACACCCAATACGCCGGCCGAAACTTTTTTACAACTCTCCTATGAGTCGTTTACCCAGAATG
CACAGGTACACTTGTTTAGAGGTAATCCTTCTTTCTAGAAGTCCTCGTGTACTGTGTAAGCGCCCACTCCACATCTCCACTCGCTCCGGGGAGACGGGCACAAGGTTCATG
AACCGAAGAGGATGGGAACCCCGACCATGGCCTGCGGCCGCAGCTATCAGTCCTCTCAGCTGACGCCCCTCACGAACCCTCGCACTCTCCCGAAATGCTCTTCGGTGTCC
ATGGCGCATCATCTCAGCAGCATCCCATGTCAAACCATGGCTTTGGGCCCACGGACTCTGTGGCCCTGCCGCAACATCACCATCACCATCGACTCCCGCCCCATGCAGCGC
TGCGCGCCCCAGGGCGTCATGGAGTGAATGTGGAATCTCCTCCTTTGCCCTCGGGCCCGCCGAGTGTGGTGGGCCAGCCTGGAATGCCTGATCCAGCCCCCAGGCCTCGA
GGACCAAAACTGAAGTTTACTCCCGAAGAGGACGCTCTACTGGTGGAGTTGAAGGAAAACAAGAACTTGACGTGGAAGCAAATTGCAGACTTCTTCCCGGGCCGAACGA
GCGGTACCTTGCAAGTCCGATACTGCACCAAGCTGAAGGCTAAGGATGTAGCTTGGAGTGACGAAATGGTTCGATTTGCTCCTGATGTATTTCTACGCCTGTCTCACACAT
GCTAATGAAGGGAATAGGTACAAAGGCTGCAGCGGGCAATGCACGAGTACGAGAACGATCGGTGGCGCATCATTGCAGGGAAGGTTGGAAATGGCTTCACCCCAGCTGC
TTGCCGCGAGAAAGCCATGCAGCTCCATGAGTAAAAGCGTTGGGGAATTTTCATATTTATATCTACTGTCGCCAGATTCGGCCCTGCTTGGACCCTCTGATCTCCTTACTCT
CCATATTGGTTCAAATGTCGGGTCACCGATAGGGCTGGTGGTGCAGGCTTGTTGTAGGCACGGGAGGATGATCAGCATAACTCTGAGTCACTATAGGGACGGGTTGATGT
AAGGTATTAAGTGATGTATGATAATTCATTTTAGCCCGGGGGAACATATGGCGCCGGCATTTGTTCGTTCGCAATGAACCGACACTAGCGTCCGCTCTCGCAGTTTAGCAC
CGGCTGATCCCGGGCTGAACGCGGCCATTGCTCGGCCGGGGCATGTGTTCCTTATCTACGGCAGACCGCAGATGACCACTGGAGCAGATTATAGACCCTAAGCCCTAAGC
CGGACACCCAATCGAGTAGGTCTGCGGACCAGGTCACTGCGGGCAGCCGGAGAAGCTCCGCAACCAATCAATCCCCGGCGCTGACTAAGGGCAGGCGACCACGGGCCGA
AGCGGCTTCAAACTCACCTCAACCTCCAAACTCCCTCATCTCCAAACGTCCTTGCCTTGTCTGCCGTCATTGCAACCCACCCACCAGGACACATGAGCAGCAAGTCCCAGC
TCACCTACACTGCCCGTGCCAGCAAGCATCCCAATGCTCTGGCGAAGAGGCTGTTCGAGATTGCCGAGGCCAAGAAaACCAATGTGACTGTCTCGGCTGACGTTACCACC
ACTAAGGAGCTACTAGATCTTGCTGACCGTCTCGGTCCCTACATTGCCGTGATCAAAACCCACATCGATATCCTCTCTGATTTCAGCAACGAGACtATTGAGGGACTTAAG
GCTCTCGCGCAGAAGCACAACTTTCTCATCTTCGAGGACCGCAAGTTCATTGACATCGGCAACACGGTCCAGAAGCAATACCACGGCGGTACCCTCCGTATCTCGGAATG
GGCCCACATCATCAACTGCAGCATTCTCCCTGGTGAGGGTATCGTCGAGGCTCTCGCTCAGACGGCGTCTGCACCGGACTTCGCCTACGGCCCCGAACGCGGTCTGTTGAT
CTTGGCAGAGATGACCTCTAAGGGCTCCTTGGCTACCGGCCAGTACACTACTTCCTCGGTCGATTATGCCCGGAAATACAAGAACTTCGTTATGGGATTCGTGTCGACGCG
CGCGTTGGGTGAGGTGCAGTCGGAAGTCAGCTCTCCTTCGGATGAGGAGGACTTTGTGGTtTTCACGACTGGTGTGAACATTAACCTCCA

>pSF532_IC
TGGAGGaAGGGATCCGCCAAGGATGCTCTGGATCCGCATCCGGCCGCTCTTGCGCCCCATCAATCGCCCGACTATAAATCGAACTACTTTCGGCATCTTCTAGACTTCCTA
ATACCGCCTAGTCATAGCAGATTCAAGCTGAGAACACCACAAGTAAATATCACCCATCATGCTTACCCTGACCGTCCCTGAAAACTACGGGTATGTGCCAATTCTACAATT
CCTTGCAGACAATGCCATTCTCCCCATGAAGTCTGATGCTAACTATCCTGCAGCTCTGTCATTGCCGTCGCTCTGGGTGCCATCCCCGTCCTGAGCTTCGTCCATGGCGCCG
TCGTGTCTCGTCTCCGCAAGGAAGCTGATTGCCCCTACCCTCACTGCTATGCGACCGTAGAGCAGTGCAAGACCAACGTAAGCCAACCTCACACAAACAGGATTCCTCGA
GCTAACATACATTCCGAACCGTGCAGCCCAAGGCCGAGCAGTTCAACTGCGCTCAGCGCGCTCATGCCAACTTCCTTGAGAACTCCAGCCAAACTATGCTCTTCCTCCTGG
TAGCTGGACTGAAGTACCCCCAGTTGGCGACTGGCCTCGGAAGCATCTGGGTCCTCGGTCGCTCACTGTTCCTTTACGGATATGTGTACTCCGGCAAGCCGCGGGGTCGCG
GTCGTTTGTACGGCAGCTTCTACTTGCTTGCACAGGGAGCTCTCTGGGGCTTGACGTCTTTTGGAGTTGCGAGGGAGTTGATTTCCTACTTCTAAGTTTGGACTGAATCCGT
GGTGTGATTGAGGTGATTGGCGATGTTTGGCTATACCAGCTATATGTAATAATCTCTACTGTATACTACTATTCAACGCATTTTACTATGCGTGCTGCTAGGGTCGGCAATG

ACAATGGCAATCTGACTGACGTGGTCTATTTCTCCATGTGCAGCAGGGAATACGAGCTCCAATGGACCTCGGGAGTGGCACAGTCAATGGCAAGGAAACTGGAGcgcaaaaaa
ccccgcccctgacagggcggggtttttcgcGATCGGAGCCTCTCGTATGCAGAGGAAATCTCCCCTGATCTTCCGAACTGGTCGTACCTGGCGACCTATGACTATGGCACCCCAGTTCTGG
GGACCTTCCACGGAAGTGACCTGCTGCAGGTGTTCTATGGGATCAAGCCAAACTATGCAGCTAGTTCTAGCCACACGTACTATCTGAGCTTTGTGTATACGCTGGATCCGA
ACTCCAACCGGGGGGAGTACATTGAGTGGCCGCAGTGGAAGGAATCGCGGCAGTTGATGAATTTCGGAGCGAACGACGCCAGTCTCCTTACGGATGATTTCCGCAACGG
GACATATGAGTTCATCCTGCAGAATACCGCGGCGTTCCACATCTGATGCCATTGGCGGAGGGGTCCGGACGGTCAGGAACTTAGCCTTATGAGATGAATGATGGACGTGT
CTGGCCTCGGAAAAGGATATATGGGGATCATAATAGTACTAGCCATATTAATGAAGGGCATATACCACGCGTTGGACCTGCGTTATAGCTTCCCGTTAGTTATAGTACCAT
CGTTATACCAGCCAATCAAGTCACCACGCACGACCGGGGACGGCGAATCCCCGGGAATTGAAAGAAATTGCATCCCAGGCCAGTGAGGCCAGCGATTGGCCACCTCTCC
AAGGCACAGGGCCATTCTGCAGCGCTGGTGGATTCATCGCAATTTCCCCCGGCCCGGCCCGACACCGCTATAGGCTGGTTCTCCCACACCATCGGAGATTCGTCGCCTAAT
GTCTCGTCCGTTCACAAGCTGAAGAGCTTGAAGTGGCGAGATGTCTCTGCAGGAATTCAAGCTAGATGCTAAGCGATATTGCATGGCAATATGTGTTGATGCATGTGCTTC
TTCCTTCAGCTTCCCCTCGTGCAGATGAGGTTTGGCTATAAATTGAAGTGGTTGGTCGGGGTTCCGTGAGGGGCTGAAGTGCTTCCTCCCTTTTAGACGCAACTGAGAGCC
TGAGCTTCATCCCCAGCATCATTACACCTCAGCACATGACATCCCCTGTAATTGGTACTCCTTGGAAGAAGTTGAACGCCCCAGTCTCAGAAGAAGCATTGGAAGGTGTTG
ATAAGTATTGGAGAGTTGCAAATTACTTGTCAATTGGTCAAATTTACTTGAGATCAAATCCATTGATGAAAGAACCATTCACTAGAGAAGATGTTAAACATAGATTGGTTG
GTCATTGGGGTACAACTCCAGGTTTAAATTTCTTGATTGGTCATATTAATAGATTCATTGCTGATCATGGTCAAAATACAGTTATTATTATGGGTCCAGGTCATGGTGGTCC
AGCTGGTACTTCACAATCTTACTTGGATGGTACTTATACAGAAACTTTTCCAAAAATTACTAAAGATGAAGCAGGTTTGCAAAAATTTTTCAGACAATTTTCTTATCCAGG
TGGTATTCCATCTCATTTTGCACCAGAAACACCAGGTTCAATTCATGAAGGTGGTGAATTGGGTTACGCTTTGTCTCATGCTTATGGTGCAATTATGGATAATCCATCTTTG
TTTGTTCCAGCTATTGTTGGTGACGGTGAAGCTGAAACTGGTCCATTGGCTACTGGTTGGCAATCAAATAAGTTGGTTAATCCAAGAACTGATGGTATTGTTTTACCAATTT
TGCATTTGAATGGTTACAAAATTGCTAATCCAACAATTTTATCAAGAATTTCTGATGAAGAATTGCATGAATTTTTCCATGGTATGGGTTACGAACCATACGAATTTGTTGC
AGGTTTTGATGATGAAGATCATATGTCAATTCATAGAAGATTTGCTGAATTATGGGAAACAATTTGGGATGAAATTTGTGATATTAAAGCAACAGCTCAAACTGATAATG
TTCATAGACCATTTTATCCAATGTTGATTTTTAGAACACCAAAAGGTTGGACTTGTCCAAAATATATTGATGGTAAAAAGACTGAAGGTTCATGGAGATCACATCAAGTTC
CATTAGCATCTGCTAGAGATACTGAAGCACATTTTGAAGTTTTGAAAAATTGGTTGGAATCTTATAAACCAGAAGAATTGTTTGATGCAAATGGTGCAGTTAAAGATGAT
GTTTTAGCTTTTATGCCAAAAGGTGAATTGAGAATTGGTGCTAATCCAAATGCTAATGGTGGTGTTATTAGAAATGATTTGAAATTACCAAATTTGGAAGATTACGAAGTT
AAAGAAGTTGCAGAATACGGTCATGGTTGGGGTCAATTGGAAGCTACTAGAACTTTAGGTGCTTATACTAGAGATATTATTAAAAATAATCCAAGAGATTTTAGAATTTTT
GGTCCAGATGAAACTGCTTCTAATAGATTGCAAGCATCTTATGAAGTTACTAATAAGCAATGGGATGCTGGTTATATTTCAGATGAAGTTGATGAACATATGCATGTTTCA
GGTCAAGTTGTTGAACAATTGTCAGAACATCAAATGGAAGGTTTCTTGGAAGCATACTTGTTAACAGGTAGACATGGTATTTGGTCATCTTATGAATCTTTTGTTCATGTTA
TTGATTCAATGTTAAATCAACATGCAAAATGGTTGGAAGCTACAGTTAGAGAAATTCCATGGAGAAAACCAATTGCATCTATGAATTTGTTAGTTTCTTCACATGTTTGGA
GACAAGATCATAATGGTTTTTCACATCAAGATCCAGGTGTTACTTCTGTTTTGTTGAATAAGTGTTTTCATAATGATCATGTTATTGGTATTTACTTTGCAACTGATGCTAAT
ATGTTGTTAGCTATTGCTGAAAAATGTTACAAATCAACTAATAAGATTAATGCAATTATTGCTGGTAAACAACCAGCAGCTACTTGGTTAACATTGGATGAAGCTAGAGC
AGAATTAGAAAAAGGTGCAGCTGCTTGGGATTGGGCATCTACTGCTAAAAATAATGATGAAGCTGAAGTTGTTTTAGCAGCAGCTGGTGACGTTCCAACTCAAGAAATTA
TGGCAGCTTCAGATAAATTGAAAGAATTGGGTATTAAATTCAAAGTTGTTAATGTTGCAGATTTGTTATCATTGCAATCTGCTAAAGAAAATGATGAAGCATTAACTGATG
AAGAATTTGCTGATATTTTTACAGCTGATAAACCAGTTTTATTTGCTTACCATTCTTATGCTCATGATGTTAGAGGTTTGATTTACGATAGACCAAATCATGATAATTTTAA
TGTTCATGGTTATGAAGAAGAAGGTTCAACTACAACTCCATACGATATGGTTAGAGTTAATAGAATTGATAGATACGAATTGACTGCTGAAGCATTGAGAATGATTGATG
CAGATAAATACGCAGATAAAATTGATGAATTGGAAAAATTCAGAGATGAAGCATTTCAATTTGCAGTTGATAATGGTTATGATCATCCAGATTATACAGATTGGGTTTAC
TCAGGTGTAAATACCGACAAAAAGGGTGCTGTAACCGCTACCGCTGCTACCGCTGGTGACAACGAAcatcatcaccaccaccatTAAGCTTGATCCACTTAACGTTACTGAAATCAT
CAAACAGCTTGACGAATCTGGATATAAGATCGTTGGTGTCGATGTCAGCTCCGGAGTTGAGACAAATGGTGTTCAGGATCTCGATAAGATACGTTCATTTGTCCAAGCAG
CAAAGAGTGCCTTCTAGTGATTTAATAGCTCCATGTCAACAAGAATAAAACGCGTTTCGGGTTTACCTCTTCCAGATACAGCTCATCTGCAATGCATTAATGCATTGGACC
TCGCAACCCTAGTACGCCCTTCAGGCTCCGGCGAAGCAGAAGAATAGCTTAGCAGAGTCTATTTTCATTTTCGGGAGACGAGATCAAGCAGATCAACGGTCGTCAAGAGT
CCTACGAGACTGAGGAATCCGCTCTTGGCTCCACGCGACTATATATTTGTCTCTAATTGTACTTTGACATGCTCCTCTTCTTTACTCTGATAGCTTGACTATGAAAATTCCG
TCACCAGCCCCTGGGTTCGCAAAGATAATTGCACTGTTTCTTCCTTGAACTCTCAAGCCTACAGGACACACATTCATCGTAGGTATAAACCTCGAAAATCATTCCTACTAA
GATGGGTATACAATAGTAACCATGCATGGTTGCCTAGTGAATGCTCCGTAACACCCAATACGCCGGCCGAAACTTTTTTACAACTCTCCTATGAGTCGTTTACCCAGAATG
CACAGGTACACTTGTTTAGAGGTAATCCTTCTTTCTAGAAGTCCTCGTGTACTGTGTAAGCGCCCACTCCACATCTCCACTCGCTCCGGGGAGACGGGCACAAGGTTCATG
AACCGAAGAGGATGGGAACCCCGACCATGGCCTGCGGCCGCAGCTATCAGTCCTCTCAGCTGACGCCCCTCACGAACCCTCGCACTCTCCCGAAATGCTCTTCGGTGTCC
ATGGCGCATCATCTCAGCAGCATCCCATGTCAAACCATGGCTTTGGGCCCACGGACTCTGTGGCCCTGCCGCAACATCACCATCACCATCGACTCCCGCCCCATGCAGCGC
TGCGCGCCCCAGGGCGTCATGGAGTGAATGTGGAATCTCCTCCTTTGCCCTCGGGCCCGCCGAGTGTGGTGGGCCAGCCTGGAATGCCTGATCCAGCCCCCAGGCCTCGA
GGACCAAAACTGAAGTTTACTCCCGAAGAGGACGCTCTACTGGTGGAGTTGAAGGAAAACAAGAACTTGACGTGGAAGCAAATTGCAGACTTCTTCCCGGGCCGAACGA
GCGGTACCTTGCAAGTCCGATACTGCACCAAGCTGAAGGCTAAGGATGTAGCTTGGAGTGACGAAATGGTTCGATTTGCTCCTGATGTATTTCTACGCCTGTCTCACACAT
GCTAATGAAGGGAATAGGTACAAAGGCTGCAGCGGGCAATGCACGAGTACGAGAACGATCGGTGGCGCATCATTGCAGGGAAGGTTGGAAATGGCTTCACCCCAGCTGC
TTGCCGCGAGAAAGCCATGCAGCTCCATGAGTAAAAGCGTTGGGGAATTTTCATATTTATATCTACTGTCGCCAGATTCGGCCCTGCTTGGACCCTCTGATCTCCTTACTCT
CCATATTGGTTCAAATGTCGGGTCACCGATAGGGCTGGTGGTGCAGGCTTGTTGTAGGCACGGGAGGATGATCAGCATAACTCTGAGTCACTATAGGGACGGGTTGATGT
AAGGTATTAAGTGATGTATGATAATTCATTTTAGCCCGGGGGAACATATGGCGCCGGCATTTGTTCGTTCGCAATGAACCGACACTAGCGTCCGCTCTCGCAGTTTAGCAC
CGGCTGATCCCGGGCTGAACGCGGCCATTGCTCGGCCGGGGCATGTGTTCCTTATCTACGGCAGACCGCAGATGACCACTGGAGCAGATTATAGACCCTAAGCCCTAAGC
CGGACACCCAATCGAGTAGGTCTGCGGACCAGGTCACTGCGGGCAGCCGGAGAAGCTCCGCAACCAATCAATCCCCGGCGCTGACTAAGGGCAGGCGACCACGGGCCGA
AGCGGCTTCAAACTCACCTCAACCTCCAAACTCCCTCATCTCCAAACGTCCTTGCCTTGTCTGCCGTCATTGCAACCCACCCACCAGGACACATGAGCAGCAAGTCCCAGC
TCACCTACACTGCCCGTGCCAGCAAGCATCCCAATGCTCTGGCGAAGAGGCTGTTCGAGATTGCCGAGGCCAAGAAaACCAATGTGACTGTCTCGGCTGACGTTACCACC
ACTAAGGAGCTACTAGATCTTGCTGACCGTCTCGGTCCCTACATTGCCGTGATCAAAACCCACATCGATATCCTCTCTGATTTCAGCAACGAGACtATTGAGGGACTTAAG
GCTCTCGCGCAGAAGCACAACTTTCTCATCTTCGAGGACCGCAAGTTCATTGACATCGGCAACACGGTCCAGAAGCAATACCACGGCGGTACCCTCCGTATCTCGGAATG
GGCCCACATCATCAACTGCAGCATTCTCCCTGGTGAGGGTATCGTCGAGGCTCTCGCTCAGACGGCGTCTGCACCGGACTTCGCCTACGGCCCCGAACGCGGTCTGTTGAT
CTTGGCAGAGATGACCTCTAAGGGCTCCTTGGCTACCGGCCAGTACACTACTTCCTCGGTCGATTATGCCCGGAAATACAAGAACTTCGTTATGGGATTCGTGTCGACGCG
CGCGTTGGGTGAGGTGCAGTCGGAAGTCAGCTCTCCTTCGGATGAGGAGGACTTTGTGGTtTTCACGACTGGTGTGAACATTAACCTCCA

>pSF522_IC
TGGAGGaAGGGATCCGCCAAGGATGCTCTGGATCCGCATCCGGCCGCTCTTGCGCCCCATCAATCGCCCGACTATAAATCGAACTACTTTCGGCATCTTCTAGACTTCCTA
ATACCGCCTAGTCATAGCAGATTCAAGCTGAGAACACCACAAGTAAATATCACCCATCATGCTTACCCTGACCGTCCCTGAAAACTACGGGTATGTGCCAATTCTACAATT
CCTTGCAGACAATGCCATTCTCCCCATGAAGTCTGATGCTAACTATCCTGCAGCTCTGTCATTGCCGTCGCTCTGGGTGCCATCCCCGTCCTGAGCTTCGTCCATGGCGCCG
TCGTGTCTCGTCTCCGCAAGGAAGCTGATTGCCCCTACCCTCACTGCTATGCGACCGTAGAGCAGTGCAAGACCAACGTAAGCCAACCTCACACAAACAGGATTCCTCGA
GCTAACATACATTCCGAACCGTGCAGCCCAAGGCCGAGCAGTTCAACTGCGCTCAGCGCGCTCATGCCAACTTCCTTGAGAACTCCAGCCAAACTATGCTCTTCCTCCTGG
TAGCTGGACTGAAGTACCCCCAGTTGGCGACTGGCCTCGGAAGCATCTGGGTCCTCGGTCGCTCACTGTTCCTTTACGGATATGTGTACTCCGGCAAGCCGCGGGGTCGCG
GTCGTTTGTACGGCAGCTTCTACTTGCTTGCACAGGGAGCTCTCTGGGGCTTGACGTCTTTTGGAGTTGCGAGGGAGTTGATTTCCTACTTCTAAGTTTGGACTGAATCCGT
GGTGTGATTGAGGTGATTGGCGATGTTTGGCTATACCAGCTATATGTAATAATCTCTACTGTATACTACTATTCAACGCATTTTACTATGCGTGCTGCTAGGGTCGGCAATG
ACAATGGCAATCTGACTGACGTGGTCTATTTCTCCATGTGCAGCAGGGAATACGAGCTCCAATGGACCTCGGGAGTGGCACAGTCAATGGCAAGGAAACTGGAGcgcaaaaaa
ccccgcccctgacagggcggggtttttcgcGATCGGAGTCGATAAGCTTCGGAGAATATGGAGCTTCATCGAATCACCGGCAGTAAGCGAAGGAGAATGTGAAGCCAGGGGTGTATAGCC
GTCGGCGAAATAGCATGCCATTAACCTAGGTACAGAAGTCCAATTGCTTCCGATCTGGTAAAAGATTCACGAGATAGTACCTTCTCCGAAGTAGGTAGAGCGAGTACCCG
GCGCGTAAGCTCCCTAATTGGCCCATCCGGCATCTGTAGGGCGTCCAAATATCGTGCCTCTCCTGCTTTGCCCGGTGTATGAAACCGGAAAGGCCGCTCAGGAGCTGGCC
AGCGGCGCAGACCGGGAACACAAGCTGGCAGTCGACCCATCCGGTGCTCTGCACTCGACCTGCTGAGGTCCCTCAGTCCCTGGTAGGCAGCTTTGCCCCGTCTGTCCGCC
CGGTGTGTCGGCGGGGTTGACAAGGTCGTTGCGTCAGTCCAACATTTGTTGCCATATTTTCCTGCTCTCCCCACCAGCTGCTCTTTTCTTTTCTCTTTCTTTTCCCATCTTCA
GTATATTCATCTTCCCATCCAAGAACCTTTATTTCCCCTAAGTAAGTACTTTGCTACATCCATACTCCATCCTTCCCATCCCTTATTCCTTTGAACCTTTCAGTTCGAGCTTTC
CCACTTCATCGCAGCTTGACTAACAGCTACCCCGCTTGAGCAGACATCACCGAATTCACCATGTCTAGACTGGACAAGAGCAAAGTCATAAACGGCGCTCTGGAATTACT
CAATGGAGTCGGTATCGAAGGCCTGACGACAAGGAAACTCGCTCAAAAGCTGGGAGTTGAGCAGCCTACCCTGTACTGGCACGTGAAGAACAAGCGGGCCCTGCTCGAT
GCCCTGCCAATCGAGATGCTGGACAGGCATCATACCCACTTCTGCCCCCTGGAAGGCGAGTCATGGCAAGACTTTCTGCGGAACAACGCCAAGTCATTCCGCTGTGCTCT
CCTCTCACATCGCGACGGGGCTAAAGTGCATCTCGGCACCCGCCCAACAGAGAAACAGTACGAAACCCTGGAAAATCAGCTCGCGTTCCTGTGTCAGCAAGGCTTCTCCC
TGGAGAACGCACTGTACGCTCTGTCCGCCGTGGGCCACTTTACACTGGGCTGCGTATTGGAGGAACAGGAGCATCAAGTAGCAAAAGAGGAAAGAGAGACACCTACCAC
CGATTCTATGCCCCCACTTCTGAGACAAGCAATTGAGCTGTTCGACCGGCAGGGAGCCGAACCTGCCTTCCTTTTCGGCCTGGAACTAATCATATGTGGCCTGGAGAAACA
GCTAAAGTGCGAAAGCGGCGGGCCGGCCGACGCCCTTGACGATTTTGACTTAGACATGCTCCCAGCCGATGCCCTTGACGACTTTGACCTTGATATGCTGCCTGCTGACGC
TCTTGACGATTTTGACCTTGACATGCTCCCCGGGTAACTAAGTAAGGATCCACTAGTACAGCAGAAGAATCTCTCTCCGCTGTTGCTTCAGTGTCTGCCATGCATTAACTTC
ATCCTACTGTCCTACCCGCAGTACCCATTCACATTTGCGGCAGATACCAGGTTGTTTTCTATTCCCTCGGTTCTTCAGTTCTTCAGATATTACATTACTCGAGAAATTGGCG
GCGATGCAGGAGTTTGGTTGGTTTTAATTGTTTCAGTCTCCTTGACCTGATTTGTATGACATGCAATGTTTCGACGGATGACTATCTCGGTGAATATACGTCATGAATCATG
CGGCCGCGCGTATCACGAGGCCCTTTCGACTTCACTCGAGTTTACCACTCCCTATCAGTGATAGAGAAAAGTGAAAGTCGAGTTTACCACTCCCTATCAGTGATAGAGAA
AAGTGAAAGTCGAGTTTACCACTCCCTATCAGTGATAGAGAAAAGTGAAAGTCGAGTTTACCACTCCCTATCAGTGATAGAGAAAAGTGAAAGTCGAGTTTACCACTCCC
TATCAGTGATAGAGAAAAGTGAAAGTCGAGTTTACCACTCCCTATCAGTGATAGAGAAAAGTGAAAGTCGAGTTTACCACTCCCTATCAGTGATAGAGAAAAGTGAAAG
TCGAGCTCCCCATCTTCAGTATATTCATCTTCCCATCCAAGAACCTTTATTTCCCCTAAGTAAGTACTTTGCTACATCCATACTCCATCCTTCCCATCCCTTATTCCTTTGAA
CCTTTCAGTTCGAGCTTTCCCACTTCATCGCAGCTTGACTAACAGCTACCCCGCTTGAGCAGACATCACCGTTTAAACACCATGACCGATACGAAAGCCGTAGAATTTGTG
GGCCACACAGCCATTGTAGTCTTTGGAGCTTCAGGGGACCTGGCTAAGAAGAAAACTTTCCCTGCCCTCTTCGGACTTTACCGTGAGGGATACCTGTCCAACAAGGTGAA
GATTATTGGCTATGCTAGATCAAAGCTGGATGACAAGGAGTTCAAGGATAGAATTGTGGGCTATTTCAAGACAAAGAACAAGGGCGACGAGGACAAAGTTCAAGAATTC
TTAAAGTTGTGCTCATATATTTCAGCTCCTTATGACAAACCAGATGGGTATGAAAAGTTGAATGAAACTATTAACGAATTCGAAAAGGAAAACAACGTCGAACAGTCTCA
CAGGTTGTTCTACTTAGCTTTGCCCCCTTCTGTTTTCATACCTGTTGCTACGGAGGTCAAGAAGTATGTTCATCCAGGTTCTAAAGGGATTGCTCGGATTATCGTGGAAAAA
CCTTTCGGGCACGACTTGCAGTCAGCAGAAGAGCTTTTGAATGCTTTGAAGCCGATCTGGAAAGAAGAGGAATTGTTTAGAATCGACCACTATCTAGGTAAGGAGATGGT
TAAGAATTTGTTGGCCTTCCGTTTTGGAAACGCATTCATCAATGCTTCTTGGGACAACAGACATATCAGCTGTATCCAAATCTCGTTCAAGGAGCCTTTTGGAACAGAAGG
TCGTGGTGGCTATTTTGACTCAATTGGTATAATAAGAGACGTCATTCAGAACCACTTGCTTCAAGTGTTAACCCTCTTAACCATGGAAAGACCCGTCTCTAATGACCCTGA
GGCTGTTAGAGATGAAAAGGTTCGCATTCTGAAGTCAATTTCTGAGCTAGATTTGAACGACGTTTTGGTGGGTCAATACGGCAAATCTGAGGATGGAAAGAAGCCAGCTT
ATGTGGATGATGAAACTGTTAAGCCAGGTTCTAAATGTGTCACATTTGCAGCCATTGGCTTGCACATCAACACAGAAAGGTGGGAAGGTGTCCCAATCATTTTAAGAGCT
GGTAAGGCTTTGAACGAAGGTAAAGTTGAGATTAGAGTGCAATACAAACAGTCTACTGGATTTCTCAATGATATTCAGCGAAATGAATTGGTCATCCGTGTGCAGCCTAA
CGAAGCCATGTACATGAAACTGAACTCCAAAGTCCCAGGTGTTTCCCAAAAGACTACTGTCACTGAGCTAGACCTCACTTACAAAGACCGTTACGAAAACTTTTACATTC
CAGAGGCATATGAATCACTTATCAGAGATGCTATGAAGGGAGATCACTCTAATTTTGTCAGAGATGACGAGTTGATACAAAGTTGGAAGATTTTCACTCCTTTACTGTATC
ACTTGGAGGGCCCTGATGCACCGGCTCCAGAAATCTATCCCTACGGATCCAGAGGTCCAGCTTCATTGACCAAATTCTTGCAAGATCATGATTACTTCTTTGAATCACGCG

ACAATTACCAATGGCCAGTGACAAGACCCGATGTGCTGCACAAGATGTAAGCTTGATCCACTTAACGTTACTGAAATCATCAAACAGCTTGACGAATCTGGATATAAGAT
CGTTGGTGTCGATGTCAGCTCCGGAGTTGAGACAAATGGTGTTCAGGATCTCGATAAGATACGTTCATTTGTCCAAGCAGCAAAGAGTGCCTTCTAGTGATTTAATAGCTC
CATGTCAACAAGAATAAAACGCGTTTCGGGTTTACCTCTTCCAGATACAGCTCATCTGCAATGCATTAATGCATTGGACCTCGCAACCCTAGTACGCCCTTCAGGCTCCGG
CGAAGCAGAAGAATAGCTTAGCAGAGTCTATTTTCATTTTCGGGAGACGAGATCAAGCAGATCAACGGTCGTCAAGAGTCCTACGAGACTGAGGAATCCGCTCTTGGCTC
CACGCGACTATATATTTGTCTCTAATTGTACTTTGACATGCTCCTCTTCTTTACTCTGATAGCTTGACTATGAAAATTCCGTCACCAGCCCCTGGGTTCGCAAAGATAATTG
CACTGTTTCTTCCTTGAACTCTCAAGCCTACAGGACACACATTCATCGTAGGTATAAACCTCGAAAATCATTCCTACTAAGATGGGTATACAATAGTAACCATGCATGGTT
GCCTAGTGAATGCTCCGTAACACCCAATACGCCGGCCGAAACTTTTTTACAACTCTCCTATGAGTCGTTTACCCAGAATGCACAGGTACACTTGTTTAGAGGTAATCCTTC
TTTCTAGAAGTCCTCGTGTACTGTGTAAGCGCCCACTCCACATCTCCACTCGCTCCGGGGAGACGGGCACAAGGTTCATGAACCGAAGAGGATGGGAACCCCGACCATGG
CCTGCGGCCGCAGCTATCAGTCCTCTCAGCTGACGCCCCTCACGAACCCTCGCACTCTCCCGAAATGCTCTTCGGTGTCCATGGCGCATCATCTCAGCAGCATCCCATGTC
AAACCATGGCTTTGGGCCCACGGACTCTGTGGCCCTGCCGCAACATCACCATCACCATCGACTCCCGCCCCATGCAGCGCTGCGCGCCCCAGGGCGTCATGGAGTGAATG
TGGAATCTCCTCCTTTGCCCTCGGGCCCGCCGAGTGTGGTGGGCCAGCCTGGAATGCCTGATCCAGCCCCCAGGCCTCGAGGACCAAAACTGAAGTTTACTCCCGAAGAG
GACGCTCTACTGGTGGAGTTGAAGGAAAACAAGAACTTGACGTGGAAGCAAATTGCAGACTTCTTCCCGGGCCGAACGAGCGGTACCTTGCAAGTCCGATACTGCACCA
AGCTGAAGGCTAAGGATGTAGCTTGGAGTGACGAAATGGTTCGATTTGCTCCTGATGTATTTCTACGCCTGTCTCACACATGCTAATGAAGGGAATAGGTACAAAGGCTG
CAGCGGGCAATGCACGAGTACGAGAACGATCGGTGGCGCATCATTGCAGGGAAGGTTGGAAATGGCTTCACCCCAGCTGCTTGCCGCGAGAAAGCCATGCAGCTCCATG
AGTAAAAGCGTTGGGGAATTTTCATATTTATATCTACTGTCGCCAGATTCGGCCCTGCTTGGACCCTCTGATCTCCTTACTCTCCATATTGGTTCAAATGTCGGGTCACCGA
TAGGGCTGGTGGTGCAGGCTTGTTGTAGGCACGGGAGGATGATCAGCATAACTCTGAGTCACTATAGGGACGGGTTGATGTAAGGTATTAAGTGATGTATGATAATTCAT
TTTAGCCCGGGGGAACATATGGCGCCGGCATTTGTTCGTTCGCAATGAACCGACACTAGCGTCCGCTCTCGCAGTTTAGCACCGGCTGATCCCGGGCTGAACGCGGCCATT
GCTCGGCCGGGGCATGTGTTCCTTATCTACGGCAGACCGCAGATGACCACTGGAGCAGATTATAGACCCTAAGCCCTAAGCCGGACACCCAATCGAGTAGGTCTGCGGAC
CAGGTCACTGCGGGCAGCCGGAGAAGCTCCGCAACCAATCAATCCCCGGCGCTGACTAAGGGCAGGCGACCACGGGCCGAAGCGGCTTCAAACTCACCTCAACCTCCAA
ACTCCCTCATCTCCAAACGTCCTTGCCTTGTCTGCCGTCATTGCAACCCACCCACCAGGACACATGAGCAGCAAGTCCCAGCTCACCTACACTGCCCGTGCCAGCAAGCAT
CCCAATGCTCTGGCGAAGAGGCTGTTCGAGATTGCCGAGGCCAAGAAaACCAATGTGACTGTCTCGGCTGACGTTACCACCACTAAGGAGCTACTAGATCTTGCTGACCG
TCTCGGTCCCTACATTGCCGTGATCAAAACCCACATCGATATCCTCTCTGATTTCAGCAACGAGACtATTGAGGGACTTAAGGCTCTCGCGCAGAAGCACAACTTTCTCATC
TTCGAGGACCGCAAGTTCATTGACATCGGCAACACGGTCCAGAAGCAATACCACGGCGGTACCCTCCGTATCTCGGAATGGGCCCACATCATCAACTGCAGCATTCTCCC
TGGTGAGGGTATCGTCGAGGCTCTCGCTCAGACGGCGTCTGCACCGGACTTCGCCTACGGCCCCGAACGCGGTCTGTTGATCTTGGCAGAGATGACCTCTAAGGGCTCCTT
GGCTACCGGCCAGTACACTACTTCCTCGGTCGATTATGCCCGGAAATACAAGAACTTCGTTATGGGATTCGTGTCGACGCGCGCGTTGGGTGAGGTGCAGTCGGAAGTCA
GCTCTCCTTCGGATGAGGAGGACTTTGTGGTtTTCACGACTGGTGTGAACATTAACCTCCA

>pSF544_IC
TGGAGGaAGGGATCCGCCAAGGATGCTCTGGATCCGCATCCGGCCGCTCTTGCGCCCCATCAATCGCCCGACTATAAATCGAACTACTTTCGGCATCTTCTAGACTTCCTAATA
CCGCCTAGTCATAGCAGATTCAAGCTGAGAACACCACAAGTAAATATCACCCATCATGCTTACCCTGACCGTCCCTGAAAACTACGGGTATGTGCCAATTCTACAATTCCTTG
CAGACAATGCCATTCTCCCCATGAAGTCTGATGCTAACTATCCTGCAGCTCTGTCATTGCCGTCGCTCTGGGTGCCATCCCCGTCCTGAGCTTCGTCCATGGCGCCGTCGTGT
CTCGTCTCCGCAAGGAAGCTGATTGCCCCTACCCTCACTGCTATGCGACCGTAGAGCAGTGCAAGACCAACGTAAGCCAACCTCACACAAACAGGATTCCTCGAGCTAACA
TACATTCCGAACCGTGCAGCCCAAGGCCGAGCAGTTCAACTGCGCTCAGCGCGCTCATGCCAACTTCCTTGAGAACTCCAGCCAAACTATGCTCTTCCTCCTGGTAGCTGGA
CTGAAGTACCCCCAGTTGGCGACTGGCCTCGGAAGCATCTGGGTCCTCGGTCGCTCACTGTTCCTTTACGGATATGTGTACTCCGGCAAGCCGCGGGGTCGCGGTCGTTTGT
ACGGCAGCTTCTACTTGCTTGCACAGGGAGCTCTCTGGGGCTTGACGTCTTTTGGAGTTGCGAGGGAGTTGATTTCCTACTTCTAAGTTTGGACTGAATCCGTGGTGTGATT
GAGGTGATTGGCGATGTTTGGCTATACCAGCTATATGTAATAATCTCTACTGTATACTACTATTCAACGCATTTTACTATGCGTGCTGCTAGGGTCGGCAATGACAATGGCAATC
TGACTGACGTGGTCTATTTCTCCATGTGCAGCAGGGAATACGAGCTCCAATGGACCTCGGGAGTGGCACAGTCAATGGCAAGGAAACTGGAGcgcaaaaaaccccgcccctgacagggcg
gggtttttcgcGATCGGAGTCGATAAGCTTCGGAGAATATGGAGCTTCATCGAATCACCGGCAGTAAGCGAAGGAGAATGTGAAGCCAGGGGTGTATAGCCGTCGGCGAAATAGC
ATGCCATTAACCTAGGTACAGAAGTCCAATTGCTTCCGATCTGGTAAAAGATTCACGAGATAGTACCTTCTCCGAAGTAGGTAGAGCGAGTACCCGGCGCGTAAGCTCCCTA
ATTGGCCCATCCGGCATCTGTAGGGCGTCCAAATATCGTGCCTCTCCTGCTTTGCCCGGTGTATGAAACCGGAAAGGCCGCTCAGGAGCTGGCCAGCGGCGCAGACCGGGA
ACACAAGCTGGCAGTCGACCCATCCGGTGCTCTGCACTCGACCTGCTGAGGTCCCTCAGTCCCTGGTAGGCAGCTTTGCCCCGTCTGTCCGCCCGGTGTGTCGGCGGGGTT
GACAAGGTCGTTGCGTCAGTCCAACATTTGTTGCCATATTTTCCTGCTCTCCCCACCAGCTGCTCTTTTCTTTTCTCTTTCTTTTCCCATCTTCAGTATATTCATCTTCCCATCCA
AGAACCTTTATTTCCCCTAAGTAAGTACTTTGCTACATCCATACTCCATCCTTCCCATCCCTTATTCCTTTGAACCTTTCAGTTCGAGCTTTCCCACTTCATCGCAGCTTGACTA
ACAGCTACCCCGCTTGAGCAGACATCACCGAATTCACCATGTCTAGACTGGACAAGAGCAAAGTCATAAACGGCGCTCTGGAATTACTCAATGGAGTCGGTATCGAAGGCC
TGACGACAAGGAAACTCGCTCAAAAGCTGGGAGTTGAGCAGCCTACCCTGTACTGGCACGTGAAGAACAAGCGGGCCCTGCTCGATGCCCTGCCAATCGAGATGCTGGAC
AGGCATCATACCCACTTCTGCCCCCTGGAAGGCGAGTCATGGCAAGACTTTCTGCGGAACAACGCCAAGTCATTCCGCTGTGCTCTCCTCTCACATCGCGACGGGGCTAAA
GTGCATCTCGGCACCCGCCCAACAGAGAAACAGTACGAAACCCTGGAAAATCAGCTCGCGTTCCTGTGTCAGCAAGGCTTCTCCCTGGAGAACGCACTGTACGCTCTGTCC
GCCGTGGGCCACTTTACACTGGGCTGCGTATTGGAGGAACAGGAGCATCAAGTAGCAAAAGAGGAAAGAGAGACACCTACCACCGATTCTATGCCCCCACTTCTGAGACA
AGCAATTGAGCTGTTCGACCGGCAGGGAGCCGAACCTGCCTTCCTTTTCGGCCTGGAACTAATCATATGTGGCCTGGAGAAACAGCTAAAGTGCGAAAGCGGCGGGCCGGC
CGACGCCCTTGACGATTTTGACTTAGACATGCTCCCAGCCGATGCCCTTGACGACTTTGACCTTGATATGCTGCCTGCTGACGCTCTTGACGATTTTGACCTTGACATGCTCC
CCGGGTAACTAAGTAAGGATCCACTAGTACAGCAGAAGAATCTCTCTCCGCTGTTGCTTCAGTGTCTGCCATGCATTAACTTCATCCTACTGTCCTACCCGCAGTACCCATTC
ACATTTGCGGCAGATACCAGGTTGTTTTCTATTCCCTCGGTTCTTCAGTTCTTCAGATATTACATTACTCGAGAAATTGGCGGCGATGCAGGAGTTTGGTTGGTTTTAATTGTTT
CAGTCTCCTTGACCTGATTTGTATGACATGCAATGTTTCGACGGATGACTATCTCGGTGAATATACGTCATGAATCATGCGGCCGCGCGTATCACGAGGCCCTTTCGACTTCAC
TCGAGTTTACCACTCCCTATCAGTGATAGAGAAAAGTGAAAGTCGAGTTTACCACTCCCTATCAGTGATAGAGAAAAGTGAAAGTCGAGTTTACCACTCCCTATCAGTGATA
GAGAAAAGTGAAAGTCGAGTTTACCACTCCCTATCAGTGATAGAGAAAAGTGAAAGTCGAGTTTACCACTCCCTATCAGTGATAGAGAAAAGTGAAAGTCGAGTTTACCAC
TCCCTATCAGTGATAGAGAAAAGTGAAAGTCGAGTTTACCACTCCCTATCAGTGATAGAGAAAAGTGAAAGTCGAGCTCCCCATCTTCAGTATATTCATCTTCCCATCCAAGA
ACCTTTATTTCCCCTAAGTAAGTACTTTGCTACATCCATACTCCATCCTTCCCATCCCTTATTCCTTTGAACCTTTCAGTTCGAGCTTTCCCACTTCATCGCAGCTTGACTAACA
GCTACCCCGCTTGAGCAGACATCACCGTTTAAACACCATGGTGAGCAAGGGCGAGGAGCTGTTCACCGGGGTGGTGCCCATCCTGGTCGAGCTGGACGGCGACGTAAACG
GCCACAAGTTCAGCGTGCGCGGCGAGGGCGAGGGCGATGCCACCAACGGCAAGCTGACCCTGAAGTTCATCTGCACCACCGGCAAGCTGCCCGTGCCCTGGCCCACCCTC
GTGACCACCCTGACCTACGGCGTGCAGTGCTTCAGCCGCTACCCCGACCACATGAAGCAGCACGACTTCTTCAAGTCCGCCATGCCCGAAGGCTACGTCCAGGAGCGCACC
ATCACCTTCAAGGACGACGGCACCTACAAGACCCGCGCCGAGGTGAAGTTCGAGGGCGACACCCTGGTGAACCGCATCGAGCTGAAGGGCATCGACTTCAAGGAGGACG
GCAACATCCTGGGGCACAAGCTGGAGTACAACTTCAACAGCCACAACGTCTATATCACCGCCGACAAGCAGAAGAACGGCATCAAGGCCAACTTCAAGATCCGCCACAAC
GTGGAGGACGGCAGCGTGCAGCTCGCCGACCACTACCAGCAGAACACCCCCATCGGCGACGGCCCCGTGCTGCTGCCCGACAACCACTACCTGAGCACCCAGTCCAAGCT
GAGCAAAGACCCCAACGAGAAGCGCGATCACATGGTCCTGCTGGAGTTCGTGACCGCCGCCGGGATCACTCACGGCATGGACGAGCTGTACAAGgccgccgccATGGAGAACT
ACCAGAAGATCGAGAAGATTGGAGAAGGAACtTATGGCGTTGTCTACAAGGCTCGCGAGCTCACCCATCCCAATCGGATTGTCGCCCTCAAGAAGATTCGGCTAGAGGCGG
AGGATGAAGGCGTTCCTAGCACAGCCATCCGTGAAATCTCCCTACTCAAAGAGATGAGCGATCCCAATATTGTGCGACTCCTGAATATTGTGCATGCCGATGGCCACAAGCT
CTATCTCGTtTTCGAATTCCTCGATCTCGACCTCAAGAAGTATATGGAGGCTCTTCCTGTCAGCGAAGGTGGACGTGGCAAAGCTCTTCCGGAAGGCTCTGCTTTGAGCAAGA
ACATGGGCCTTGGCGATGCCATGGTCAAGAAGTTCATGGCTCAGCTAGTGGAGGGTATTCGTTACTGCCACAGCCACCGTATTTTACACCGTGATCTCAAGCCCCAGAACCT
TCTGATCGACCGCGATGGCAATCTTAAGCTGGCGGACTTTGGATTGGCTAGAGCTTTCGGTGTTCCTCTGAGAACCTACACCCATGAGGTTGTCACCCTTTGGTACCGCTCCC
CTGAGATTCTCTTGGGTGGTCGTCAGTACTCGACCGGCGTCGACATGTGGTCCGCTGGTGCCATTTTCGCGGAGATGTGCACTCGCAAGCCTTTGTTCCCTGGTGATTCGGA
GATTGATGAGATCTTCAAGATCTTCCGTCTCCTTGGTACCCCAGATGAAGCTATCTGGCCCGGCGTCACCTCTTTCCCCGACTTCAAACCCACTTTCCCTAAATGGAAACGTG
ATGAAACCCGTGCACTCGTCCCTGACCTTGAAGAaGATGGTCTGGACCTCCTCGATGCTCTGCTCGAGTATGACCCTGCACGACGTATCTCCGCCAAACAGGCCTGCATGCA
TCCCTACTTCCGGAACGGCAGTGCCTACTACTCAGGACGTACCCGGAGGAATGGTTTTAACTAGGCTTGATCCACTTAACGTTACTGAAATCATCAAACAGCTTGACGAATCT
GGATATAAGATCGTTGGTGTCGATGTCAGCTCCGGAGTTGAGACAAATGGTGTTCAGGATCTCGATAAGATACGTTCATTTGTCCAAGCAGCAAAGAGTGCCTTCTAGTGATT
TAATAGCTCCATGTCAACAAGAATAAAACGCGTTTCGGGTTTACCTCTTCCAGATACAGCTCATCTGCAATGCATTAATGCATTGGACCTCGCAACCCTAGTACGCCCTTCAG
GCTCCGGCGAAGCAGAAGAATAGCTTAGCAGAGTCTATTTTCATTTTCGGGAGACGAGATCAAGCAGATCAACGGTCGTCAAGAGTCCTACGAGACTGAGGAATCCGCTCT
TGGCTCCACGCGACTATATATTTGTCTCTAATTGTACTTTGACATGCTCCTCTTCTTTACTCTGATAGCTTGACTATGAAAATTCCGTCACCAGCCCCTGGGTTCGCAAAGATAA
TTGCACTGTTTCTTCCTTGAACTCTCAAGCCTACAGGACACACATTCATCGTAGGTATAAACCTCGAAAATCATTCCTACTAAGATGGGTATACAATAGTAACCATGCATGGTT
GCCTAGTGAATGCTCCGTAACACCCAATACGCCGGCCGAAACTTTTTTACAACTCTCCTATGAGTCGTTTACCCAGAATGCACAGGTACACTTGTTTAGAGGTAATCCTTCTT
TCTAGAAGTCCTCGTGTACTGTGTAAGCGCCCACTCCACATCTCCACTCGCTCCGGGGAGACGGGCACAAGGTTCATGAACCGAAGAGGATGGGAACCCCGACCATGGCCT
GCGGCCGCAGCTATCAGTCCTCTCAGCTGACGCCCCTCACGAACCCTCGCACTCTCCCGAAATGCTCTTCGGTGTCCATGGCGCATCATCTCAGCAGCATCCCATGTCAAAC
CATGGCTTTGGGCCCACGGACTCTGTGGCCCTGCCGCAACATCACCATCACCATCGACTCCCGCCCCATGCAGCGCTGCGCGCCCCAGGGCGTCATGGAGTGAATGTGGAAT
CTCCTCCTTTGCCCTCGGGCCCGCCGAGTGTGGTGGGCCAGCCTGGAATGCCTGATCCAGCCCCCAGGCCTCGAGGACCAAAACTGAAGTTTACTCCCGAAGAGGACGCTC
TACTGGTGGAGTTGAAGGAAAACAAGAACTTGACGTGGAAGCAAATTGCAGACTTCTTCCCGGGCCGAACGAGCGGTACCTTGCAAGTCCGATACTGCACCAAGCTGAAG
GCTAAGGATGTAGCTTGGAGTGACGAAATGGTTCGATTTGCTCCTGATGTATTTCTACGCCTGTCTCACACATGCTAATGAAGGGAATAGGTACAAAGGCTGCAGCGGGCAAT
GCACGAGTACGAGAACGATCGGTGGCGCATCATTGCAGGGAAGGTTGGAAATGGCTTCACCCCAGCTGCTTGCCGCGAGAAAGCCATGCAGCTCCATGAGTAAAAGCGTT
GGGGAATTTTCATATTTATATCTACTGTCGCCAGATTCGGCCCTGCTTGGACCCTCTGATCTCCTTACTCTCCATATTGGTTCAAATGTCGGGTCACCGATAGGGCTGGTGGTGC
AGGCTTGTTGTAGGCACGGGAGGATGATCAGCATAACTCTGAGTCACTATAGGGACGGGTTGATGTAAGGTATTAAGTGATGTATGATAATTCATTTAGCCCGGGGGAACAT
ATGGCGCCGGCATTTGTTCGTTCGCAATGAACCGACACTAGCGTCCGCTCTCGCAGTTTAGCACCGGCTGATCCCGGGCTGAACGCGGCCATTGCTCGGCCGGGGCATGTGT
TCCTTATCTACGGCAGACCGCAGATGACCACTGGAGCAGATTATAGACCCTAAGCCCTAAGCCGGACACCCAATCGAGTAGGTCTGCGGACCAGGTCACTGCGGGCAGCCG
GAGAAGCTCCGCAACCAATCAATCCCCGGCGCTGACTAAGGGCAGGCGACCACGGGCCGAAGCGGCTTCAAACTCACCTCAACCTCCAAACTCCCTCATCTCCAAACGTC
CTTGCCTTGTCTGCCGTCATTGCAACCCACCCACCAGGACACATGAGCAGCAAGTCCCAGCTCACCTACACTGCCCGTGCCAGCAAGCATCCCAATGCTCTGGCGAAGAGG
CTGTTCGAGATTGCCGAGGCCAAGAAaACCAATGTGACTGTCTCGGCTGACGTTACCACCACTAAGGAGCTACTAGATCTTGCTGACCGTCTCGGTCCCTACATTGCCGTGAT
CAAAACCCACATCGATATCCTCTCTGATTTCAGCAACGAGACtATTGAGGGACTTAAGGCTCTCGCGCAGAAGCACAACTTTCTCATCTTCGAGGACCGCAAGTTCATTGAC
ATCGGCAACACGGTCCAGAAGCAATACCACGGCGGTACCCTCCGTATCTCGGAATGGGCCCACATCATCAACTGCAGCATTCTCCCTGGTGAGGGTATCGTCGAGGCTCTCG
CTCAGACGGCGTCTGCACCGGACTTCGCCTACGGCCCCGAACGCGGTCTGTTGATCTTGGCAGAGATGACCTCTAAGGGCTCCTTGGCTACCGGCCAGTACACTACTTCCTC
GGTCGATTATGCCCGGAAATACAAGAACTTCGTTATGGGATTCGTGTCGACGCGCGCGTTGGGTGAGGTGCAGTCGGAAGTCAGCTCTCCTTCGGATGAGGAGGACTTTGTG
GTtTTCACGACTGGTGTGAACATTAACCTCCA

>pSF543_IC
TGGAGGaAGGGATCCGCCAAGGATGCTCTGGATCCGCATCCGGCCGCTCTTGCGCCCCATCAATCGCCCGACTATAAATCGAACTACTTTCGGCATCTTCTAGACTTCCTA
ATACCGCCTAGTCATAGCAGATTCAAGCTGAGAACACCACAAGTAAATATCACCCATCATGCTTACCCTGACCGTCCCTGAAAACTACGGGTATGTGCCAATTCTACAATT
CCTTGCAGACAATGCCATTCTCCCCATGAAGTCTGATGCTAACTATCCTGCAGCTCTGTCATTGCCGTCGCTCTGGGTGCCATCCCCGTCCTGAGCTTCGTCCATGGCGCCG

TCGTGTCTCGTCTCCGCAAGGAAGCTGATTGCCCCTACCCTCACTGCTATGCGACCGTAGAGCAGTGCAAGACCAACGTAAGCCAACCTCACACAAACAGGATTCCTCGA
GCTAACATACATTCCGAACCGTGCAGCCCAAGGCCGAGCAGTTCAACTGCGCTCAGCGCGCTCATGCCAACTTCCTTGAGAACTCCAGCCAAACTATGCTCTTCCTCCTGG
TAGCTGGACTGAAGTACCCCCAGTTGGCGACTGGCCTCGGAAGCATCTGGGTCCTCGGTCGCTCACTGTTCCTTTACGGATATGTGTACTCCGGCAAGCCGCGGGGTCGCG
GTCGTTTGTACGGCAGCTTCTACTTGCTTGCACAGGGAGCTCTCTGGGGCTTGACGTCTTTTGGAGTTGCGAGGGAGTTGATTTCCTACTTCTAAGTTTGGACTGAATCCGT
GGTGTGATTGAGGTGATTGGCGATGTTTGGCTATACCAGCTATATGTAATAATCTCTACTGTATACTACTATTCAACGCATTTTACTATGCGTGCTGCTAGGGTCGGCAATG
ACAATGGCAATCTGACTGACGTGGTCTATTTCTCCATGTGCAGCAGGGAATACGAGCTCCAATGGACCTCGGGAGTGGCACAGTCAATGGCAAGGAAACTGGAGcgcaaaaaa
ccccgcccctgacagggcggggttttttcgcGATCGGAGTCGATAAGCTTCGGAGAATATGGAGCTTCATCGAATCACCGGCAGTAAGCGAAGGAGAATGTGAAGCCAGGGGTGTATAGCC
GTCGGCGAAATAGCATGCCATTAACCTAGGTACAGAAGTCCAATTGCTTCCGATCTGGTAAAAGATTCACGAGATAGTACCTTCTCCGAAGTAGGTAGAGCGAGTACCCG
GCGCGTAAGCTCCCTAATTGGCCCATCCGGCATCTGTAGGGCGTCCAAATATCGTGCCTCTCCTGCTTTGCCCGGTGTATGAAACCGGAAAGGCCGCTCAGGAGCTGGCC
AGCGGCGCAGACCGGGAACACAAGCTGGCAGTCGACCCATCCGGTGCTCTGCACTCGACCTGCTGAGGTCCCTCAGTCCCTGGTAGGCAGCTTTGCCCCGTCTGTCCGCC
CGGTGTGTCGGCGGGGTTGACAAGGTCGTTGCGTCAGTCCAACATTTGTTGCCATATTTTCCTGCTCTCCCCACCAGCTGCTCTTTTCTTTTCTCTTTCTTTTCCCATCTTCA
GTATATTCATCTTCCCATCCAAGAACCTTTATTTCCCCTAAGTAAGTACTTTGCTACATCCATACTCCATCCTTCCCATCCCTTATTCCTTTGAACCTTTCAGTTCGAGCTTTC
CCACTTCATCGCAGCTTGACTAACAGCTACCCCGCTTGAGCAGACATCACCGAATTCACCATGTCTAGACTGGACAAGAGCAAAGTCATAAACGGCGCTCTGGAATTACT
CAATGGAGTCGGTATCGAAGGCCTGACGACAAGGAAACTCGCTCAAAAGCTGGGAGTTGAGCAGCCTACCCTGTACTGGCACGTGAAGAACAAGCGGGCCCTGCTCGAT
GCCCTGCCAATCGAGATGCTGGACAGGCATCATACCCACTTCTGCCCCCTGGAAGGCGAGTCATGGCAAGACTTTCTGCGGAACAACGCCAAGTCATTCCGCTGTGCTCT
CCTCTCACATCGCGACGGGGCTAAAGTGCATCTCGGCACCCGCCCAACAGAGAAACAGTACGAAACCCTGGAAAATCAGCTCGCGTTCCTGTGTCAGCAAGGCTTCTCCC
TGGAGAACGCACTGTACGCTCTGTCCGCCGTGGGCCACTTTACACTGGGCTGCGTATTGGAGGAACAGGAGCATCAAGTAGCAAAAGAGGAAAGAGAGACACCTACCAC
CGATTCTATGCCCCCACTTCTGAGACAAGCAATTGAGCTGTTCGACCGGCAGGGAGCCGAACCTGCCTTCCTTTTCGGCCTGGAACTAATCATATGTGGCCTGGAGAAACA
GCTAAAGTGCGAAAGCGGCGGGCCGGCCGACGCCCTTGACGATTTTGACTTAGACATGCTCCCAGCCGATGCCCTTGACGACTTTGACCTTGATATGCTGCCTGCTGACGC
TCTTGACGATTTTGACCTTGACATGCTCCCCGGGTAACTAAGTAAGGATCCACTAGTACAGCAGAAGAATCTCTCTCCGCTGTTGCTTCAGTGTCTGCCATGCATTAACTTC
ATCCTACTGTCCTACCCGCAGTACCCATTCACATTTGCGGCAGATACCAGGTTGTTTTCTATTCCCTCGGTTCTTCAGTTCTTCAGATATTACATTACTCGAGAAATTGGCG
GCGATGCAGGAGTTTGGTTGGTTTTAATTGTTTCAGTCTCCTTGACCTGATTTGTATGACATGCAATGTTTCGACGGATGACTATCTCGGTGAATATACGTCATGAATCATG
CGGCCGCGCGTATCACGAGGCCCTTTCGACTTCACTCGAGTTTACCACTCCCTATCAGTGATAGAGAAAAGTGAAAGTCGAGTTTACCACTCCCTATCAGTGATAGAGAA
AAGTGAAAGTCGAGTTTACCACTCCCTATCAGTGATAGAGAAAAGTGAAAGTCGAGTTTACCACTCCCTATCAGTGATAGAGAAAAGTGAAAGTCGAGTTTACCACTCCC
TATCAGTGATAGAGAAAAGTGAAAGTCGAGTTTACCACTCCCTATCAGTGATAGAGAAAAGTGAAAGTCGAGTTTACCACTCCCTATCAGTGATAGAGAAAAGTGAAAG
TCGAGCTCCCCATCTTCAGTATATTCATCTTCCCATCCAAGAACCTTTATTTCCCCTAAGTAAGTACTTTGCTACATCCATACTCCATCCTTCCCATCCCTTATTCCTTTGAA
CCTTTCAGTTCGAGCTTTCCCACTTCATCGCAGCTTGACTAACAGCTACCCCGCTTGAGCAGACATCACCGTTTAAACACCATGGTGAGCAAGGGCGAGGAGCTGTTCACC
GGGGTGGTGCCCATCCTGGTCGAGCTGGACGGCGACGTAAACGGCCACAAGTTCAGCGTGCGCGGCGAGGGCGAGGGCGATGCCACCAACGGCAAGCTGACCCTGAAGT
TCATCTGCACCACCGGCAAGCTGCCCGTGCCCTGGCCCACCCTCGTGACCACCCTGACCTACGGCGTGCAGTGCTTCAGCCGCTACCCCGACCACATGAAGCAGCACGAC
TTCTTCAAGTCCGCCATGCCCGAAGGCTACGTCCAGGAGCGCACCATCACCTTCAAGGACGACGGCACCTACAAGACCCGCGCCGAGGTGAAGTTCGAGGGCGACACCCT
GGTGAACCGCATCGAGCTGAAGGGCATCGACTTCAAGGAGGACGGCAACATCCTGGGGCACAAGCTGGAGTACAACTTCAACAGCCACAACGTCTATATCACCGCCGAC
AAGCAGAAGAACGGCATCAAGGCCAACTTCAAGATCCGCCACAACGTGGAGGACGGCAGCGTGCAGCTCGCCGACCACTACCAGCAGAACACCCCCATCGGCGACGGC
CCCGTGCTGCTGCCCGACAACCACTACCTGAGCACCCAGTCCAAGCTGAGCAAAGACCCCAACGAGAAGCGCGATCACATGGTCCTGCTGGAGTTCGTGACCGCCGCCGG
GATCACTCACGGCATGGACGAGCTGTACAAGgccgccgccATGAGAGAAGTTATTTCTTTGAACGTTGGTCAGGCTGGTTGCCAGATTGCCAACTCCTGCTGGGAGCTCTACT
GCCTTGAGCACGGTATCCAGGCCGACGGTTACTTGACCGAGGAGCGCAAGAAGGATGACCCCGACCACGGATTCAGCACCTTCTTCTCCGAGACTGGTCAGGGCAAGTAT
GTCCCTCGTACCATTTACTGCGATCTGGAACCCAATGTTGTGGATGAGGTCCGCACCGGTACCTACCGCAGTCTTTTCCACCCCGAGAACATGATCACTGGCAAGGAGGAT
GCCTCGAACAACTATGCCCGTGGTCACTACACCGTCGGCAAGGAGATGATCGACCAGGTCCTCGACAAGGTCCGCCGTGTTGCCGACAACTGCGCTGGTCTaCAGGGTTTC
CTGGTtTTCCACTCCTTCGGTGGTGGTACTGGTTCCGGTTTCGGTGCTCTCCTGATGGAGCGTCTGTCCGTTGACTACGGCAAGAAGTCCAAGCTGGAATTCTGCGTTTACC
CTGCTCCCCAGAACGCCACCTCCGTTGTTGAGCCCTACAACTCTATCCTCACCACCCACACCACCCTTGAGCACTCCGACTGCAGCTTCATGGTTGACAACGAGGCCATCT
ACGACATCTGCCGCCGCAACCTCGGCATCGAGCGCCCTAGCTATGAGAACCTCAACCGCCTGATCGCCCAGGTTGTCTCTTCCATCACCGCTTCCCTCCGTTTCGACGGTT
CCCTCAACGTCGATCTCAACGAGTTCCAGACCAACCTGGTCCCCTACCCCCGTATTCACTTCCCTCTGGTGGCCTACGCCCCCGTCATCTCCGCGGACAAGGCCTCCCACG
AGTCCAACTCCGTCAGCGAGATCACCATGAGCTGCTTTGAGCCCAATAACCAGATGGTCAAGTGTGATCCCCGCAACGGCAAGTACATGGCCACTTGCCTGCTGTACCGT
GGTGATGTCGTCCCCAAGGAGACtCACGCTGCCGTGGCCACCCTtAAGACTAAGCGCACCATCCAGTTCGTCGACTGGTGCCCTACTGGTTTCAAGATCGGTATCTGCTACC
AGCCTCCCCAGCAGGTCCCCAACGGCGACCTTGCCAACCTCAAGCGCGCTGTCTGCATGCTGTCCAACACCACCGCCATCTCCGAGGCCTGGTCCGCTCTCGACCACAAGT
TCGACCTCATGTACTCCAAGCGTGCTTTCGTCCACTGGTACGTTGGTGAGGGTATGGAGGAGGGTGAATTCTCCGAGGCCCGTGAGGACCTGGCTGCCCTTGAGCGCGAC
TACGAGGAGGTTGCCAGCGACTCCCTGGAGGAGGAGGTTGAGGCCGAGTACTAGGCTTGATCCACTTAACGTTACTGAAATCATCAAACAGCTTGACGAATCTGGATATA
AGATCGTTGGTGTCGATGTCAGCTCCGGAGTTGAGACAAATGGTGTTCAGGATCTCGATAAGATACGTTCATTTGTCCAAGCAGCAAAGAGTGCCTTCTAGTGATTTAATA
GCTCCATGTCAACAAGAATAAAACGCGTTTCGGGTTTACCTCTTCCAGATACAGCTCATCTGCAATGCATTAATGCATTGGACCTCGCAACCCTAGTACGCCCTTCAGGCT
CCGGCGAAGCAGAAGAATAGCTTAGCAGAGTCTATTTTCATTTTCGGGAGACGAGATCAAGCAGATCAACGGTCGTCAAGAGTCCTACGAGACTGAGGAATCCGCTCTTG
GCTCCACGCGACTATATATTTGTCTCTAATTGTACTTTGACATGCTCCTCTTCTTTACTCTGATAGCTTGACTATGAAAATTCCGTCACCAGCCCCTGGGTTCGCAAAGATA
ATTGCACTGTTTCTTCCTTGAACTCTCAAGCCTACAGGACACACATTCATCGTAGGTATAAACCTCGAAAATCATTCCTACTAAGATGGGTATACAATAGTAACCATGCAT
GGTTGCCTAGTGAATGCTCCGTAACACCCAATACGCCGGCCGAAACTTTTTTACAACTCTCCTATGAGTCGTTTACCCAGAATGCACAGGTACACTTGTTTAGAGGTAATC
CTTCTTTCTAGAAGTCCTCGTGTACTGTGTAAGCGCCCACTCCACATCTCCACTCGCTCCGGGGAGACGGGCACAAGGTTCATGAACCGAAGAGGATGGGAACCCCGACC
ATGGCCTGCGGCCGCAGCTATCAGTCCTCTCAGCTGACGCCCCTCACGAACCCTCGCACTCTCCCGAAATGCTCTTCGGTGTCCATGGCGCATCATCTCAGCAGCATCCCA
TGTCAAACCATGGCTTTGGGCCCACGGACTCTGTGGCCCTGCCGCAACATCACCATCACCATCGACTCCCGCCCCATGCAGCGCTGCGCGCCCCAGGGCGTCATGGAGTG
AATGTGGAATCTCCTCCTTTGCCCTCGGCCCGCCGAGTGTGGTGGGCCAGCCTGGAATGCCTGATCCAGCCCCCAGGCCTCGAGGACCAAAACTGAAGTTTACTCCCGA
AGAGGACGCTCTACTGGTGGAGTTGAAGGAAAACAAGAACTTGACGTGGAAGCAAATTGCAGACTTCTTCCCGGGCCGAACGAGCGGTACCTTGCAAGTCCGATACTGC
ACCAAGCTGAAGGCTAAGGATGTAGCTTGGAGTGACGAAATGGTTCGATTTGCTCCTGATGTATTTCTACGCCTGTCTCACACATGCTAATGAAGGGAATAGGTACAAAG
GCTGCAGCGGGCAATGCACGAGTACGAGAACGATCGGTGGCGCATCATTGCAGGGAAGGTTGGAAATGGCTTCACCCCAGCTGCTTGCCGCGAGAAAGCCATGCAGCTC
CATGAGTAAAAGCGTTGGGGAATTTTCATATTTATATCTACTGTCGCCAGATTCGGCCCTGCTTGGACCCTCTGATCTCCTTACTCTCCATATTGGTTCAAATGTCGGGTCA
CCGATAGGGCTGGTGGTGCAGGCTTGTTGTAGGCACGGGAGGATGATCAGCATAACTCTGAGTCACTATAGGGACGGGTTGATGTAAGGTATTAAGTGATGTATGATAAT
TCATTTTAGCCCGGGGGAACATATGGCGCCGGCATTTGTTCGTTCGCAATGAACCGACACTAGCGTCCGCTCTCGCAGTTTAGCACCGGCTGATCCCGGGCTGAACGCGGC
CATTGCTCGGCCGGGGCATGTGTTCCTTATCTACGGCAGACCGCAGATGACCACTGGAGCAGATTATAGACCCTAAGCCCTAAGCCGGACACCCAATCGAGTAGGTCTGC
GGACCAGGTCACTGCGGGCAGCCGGAGAAGCTCCGCAACCAATCAATCCCCGGCGCTGACTAAGGGCAGGCGACCACGGGCCGAAGCGGCTTCAAACTCACCTCAACCT
CCAAACTCCCTCATCTCCAAACGTCCTTGCCTTGTCTGCCGTCATTGCAACCCACCCACCAGGACACATGAGCAGCAAGTCCCAGCTCACCTACACTGCCCGTGCCAGCAA
GCATCCCAATGCTCTGGCGAAGAGGCTGTTCGAGATTGCCGAGGCCAAGAAaACCAATGTGACTGTCTCGGCTGACGTTACCACCACTAAGGAGCTACTAGATCTTGCTG
ACCGTCTCGGTCCCTACATTGCCGTGATCAAAACCCACATCGATATCCTCTCTGATTTCAGCAACGAGACtATTGAGGGACTTAAGGCTCTCGCGCAGAAGCACAACTTTC
TCATCTTCGAGGACCGCAAGTTCATTGACATCGGCAACACGGTCCAGAAGCAATACCACGGCGGTACCCTCCGTATCTCGGAATGGGCCCACATCATCAACTGCAGCATT
CTCCCTGGTGAGGGTATCGTCGAGGCTCTCGCTCAGACGGCGTCTGCACCGGACTTCGCCTACGGCCCCGAACGCGGTCTGTTGATCTTGGCAGAGATGACCTCTAAGGG
CTCCTTGGCTACCGGCCAGTACACTACTTCCTCGGTCGATTATGCCCGGAAATACAAGAACTTCGTTATGGGATTCGTGTCGACGCGCGCGTTGGGTGAGGTGCAGTCGGA
AGTCAGCTCTCCTTCGGATGAGGAGGACTTTGTGGTtTTCACGACTGGTGTGAACATTAACCTCCA

>pSF536
TGGAGGaAGGGATCCGCCAAGGATGCTCTGGATCCGCATCCGGCCGCTCTTGCGCCCCATCAATCGCCCGACTATAAATCGAACTACTTTCGGCATCTTCTAGACTTCCTA
ATACCGCCTAGTCATAGCAGATTCAAGCTGAGAACACCACAAGTAAATATCACCCATCATGCTTACCCTGACCGTCCCTGAAAACTACGGGTATGTGCCAATTCTACAATT
CCTTGCAGACAATGCCATTCTCCCCATGAAGTCTGATGCTAACTATCCTGCAGCTCTGTCATTGCCGTCGCTCTGGGTGCCATCCCCGTCCTGAGCTTCGTCCATGGCGCCG
TCGTGTCTCGTCTCCGCAAGGAAGCTGATTGCCCCTACCCTCACTGCTATGCGACCGTAGAGCAGTGCAAGACCAACGTAAGCCAACCTCACACAAACAGGATTCCTCGA
GCTAACATACATTCCGAACCGTGCAGCCCAAGGCCGAGCAGTTCAACTGCGCTCAGCGCGCTCATGCCAACTTCCTTGAGAACTCCAGCCAAACTATGCTCTTCCTCCTGG
TAGCTGGACTGAAGTACCCCCAGTTGGCGACTGGCCTCGGAAGCATCTGGGTCCTCGGTCGCTCACTGTTCCTTTACGGATATGTGTACTCCGGCAAGCCGCGGGGTCGCG
GTCGTTTGTACGGCAGCTTCTACTTGCTTGCACAGGGAGCTCTCTGGGGCTTGACGTCTTTTGGAGTTGCGAGGGAGTTGATTTCCTACTTCTAAGTTTGGACTGAATCCGT
GGTGTGATTGAGGTGATTGGCGATGTTTGGCTATACCAGCTATATGTAATAATCTCTACTGTATACTACTATTCAACGCATTTTACTATGCGTGCTGCTAGGGTCGGCAATG
ACAATGGCAATCTGACTGACGTGGTCTATTTCTCCATGTGCAGCAGGGAATACGAGCTCCAATGGACCTCGGGAGTGGCACAGTCAATGGCAAGGAAACTGGAGcgcaaaaaa
ccccgcccctgacagggcggggttttttcgcGATCGGAGTCGATAAGCTTCGGAGAATATGGAGCTTCATCGAATCACCGGCAGTAAGCGAAGGAGAATGTGAAGCCAGGGGTGTATAGCC
GTCGGCGAAATAGCATGCCATTAACCTAGGTACAGAAGTCCAATTGCTTCCGATCTGGTAAAAGATTCACGAGATAGTACCTTCTCCGAAGTAGGTAGAGCGAGTACCCG
GCGCGTAAGCTCCCTAATTGGCCCATCCGGCATCTGTAGGGCGTCCAAATATCGTGCCTCTCCTGCTTTGCCCGGTGTATGAAACCGGAAAGGCCGCTCAGGAGCTGGCC
AGCGGCGCAGACCGGGAACACAAGCTGGCAGTCGACCCATCCGGTGCTCTGCACTCGACCTGCTGAGGTCCCTCAGTCCCTGGTAGGCAGCTTTGCCCCGTCTGTCCGCC
CGGTGTGTCGGCGGGGTTGACAAGGTCGTTGCGTCAGTCCAACATTTGTTGCCATATTTTCCTGCTCTCCCCACCAGCTGCTCTTTTCTTTTCTCTTTCTTTTCCCATCTTCA
GTATATTCATCTTCCCATCCAAGAACCTTTATTTCCCCTAAGTAAGTACTTTGCTACATCCATACTCCATCCTTCCCATCCCTTATTCCTTTGAACCTTTCAGTTCGAGCTTTC
CCACTTCATCGCAGCTTGACTAACAGCTACCCCGCTTGAGCAGACATCACCGAATTCACCATGTCTAGACTGGACAAGAGCAAAGTCATAAACGGCGCTCTGGAATTACT
CAATGGAGTCGGTATCGAAGGCCTGACGACAAGGAAACTCGCTCAAAAGCTGGGAGTTGAGCAGCCTACCCTGTACTGGCACGTGAAGAACAAGCGGGCCCTGCTCGAT
GCCCTGCCAATCGAGATGCTGGACAGGCATCATACCCACTTCTGCCCCCTGGAAGGCGAGTCATGGCAAGACTTTCTGCGGAACAACGCCAAGTCATTCCGCTGTGCTCT
CCTCTCACATCGCGACGGGGCTAAAGTGCATCTCGGCACCCGCCCAACAGAGAAACAGTACGAAACCCTGGAAAATCAGCTCGCGTTCCTGTGTCAGCAAGGCTTCTCCC
TGGAGAACGCACTGTACGCTCTGTCCGCCGTGGGCCACTTTACACTGGGCTGCGTATTGGAGGAACAGGAGCATCAAGTAGCAAAAGAGGAAAGAGAGACACCTACCAC
CGATTCTATGCCCCCACTTCTGAGACAAGCAATTGAGCTGTTCGACCGGCAGGGAGCCGAACCTGCCTTCCTTTTCGGCCTGGAACTAATCATATGTGGCCTGGAGAAACA
GCTAAAGTGCGAAAGCGGCGGGCCGGCCGACGCCCTTGACGATTTTGACTTAGACATGCTCCCAGCCGATGCCCTTGACGACTTTGACCTTGATATGCTGCCTGCTGACGC
TCTTGACGATTTTGACCTTGACATGCTCCCCGGGTAACTAAGTAAGGATCCACTAGTACAGCAGAAGAATCTCTCTCCGCTGTTGCTTCAGTGTCTGCCATGCATTAACTTC
ATCCTACTGTCCTACCCGCAGTACCCATTCACATTTGCGGCAGATACCAGGTTGTTTTCTATTCCCTCGGTTCTTCAGTTCTTCAGATATTACATTACTCGAGAAATTGGCG
GCGATGCAGGAGTTTGGTTGGTTTTAATTGTTTCAGTCTCCTTGACCTGATTTGTATGACATGCAATGTTTCGACGGATGACTATCTCGGTGAATATACGTCATGAATCATG
CGGCCGCGCGTATCACGAGGCCCTTTCGACTTCACTCGAGTTTACCACTCCCTATCAGTGATAGAGAAAAGTGAAAGTCGAGTTTACCACTCCCTATCAGTGATAGAGAA
AAGTGAAAGTCGAGTTTACCACTCCCTATCAGTGATAGAGAAAAGTGAAAGTCGAGTTTACCACTCCCTATCAGTGATAGAGAAAAGTGAAAGTCGAGTTTACCACTCCC
TATCAGTGATAGAGAAAAGTGAAAGTCGAGTTTACCACTCCCTATCAGTGATAGAGAAAAGTGAAAGTCGAGTTTACCACTCCCTATCAGTGATAGAGAAAAGTGAAAG
TCGAGCTCCCCATCTTCAGTATATTCATCTTCCCATCCAAGAACCTTTATTTCCCCTAAGTAAGTACTTTGCTACATCCATACTCCATCCTTCCCATCCCTTATTCCTTTGAA
CCTTTCAGTTCGAGCTTTCCCACTTCATCGCAGCTTGACTAACAGCTACCCCGCTTGAGCAGACATCACCGTTTAAACACCATGCCTGGAGAGGTCATCGACAGGCCAAAT

CCCAAGGCTGAGCCTTCACACATCCCCGATCTTGTCAATCAATTGCAGGTCAAACTTCAAGAGACGCGTTTGGAGGAAACTGATTACAATGCCCTTCTGAAATTCCGCCGT
GCAGCGGCCTACATTGCTGCTGCAATGATCTTTCTCCAAGACAATGTGCTGCTGAAGCAGAATCTAAGGCACGAGGACATCAAGCCCAGGCTTCTTGGCCACTGGGGAAC
ATGTCCCGGGTTGATTCTTGTATACTCTCACTTGAACTACATCATCAGAAAGCAGAACCTGGATATGTTGTATGTCGTCGGGCCTGGCCACGGCGCGCCAGCTATTTTGGC
CTCACTGTGGCTTGAGGGCTCTTTAGAGAAATTCTACCCCCACTACTCACGAGACATGGATGGcCTCCATGAGCTCATCTCGACCTTCAGCACAAGTGCTGGATTACCAAG
CCATATCAATGCGGAAACTCCCGGTGCAATCCATGAAGGTGGTGAATTGGGTTATGCGTTAGCTGTCTCTTTTGGTGCTGTTATGGACAATCCCGACATGATCGTCACCTG
CGTGGTTGGTGACGGGGAAGCAGAAACTGGTCCTACCGCGACGTCCTGGCATGCAATCAAGTACATTGACCCCGCAGAATCAGGTGCCGTCCTGCCGATTCTCCACGTTA
ATGGCTTTAAGATCAGCGAGCGCACCATTTATGGCTGCATGGACAACAAAGAGCTGGTgTCCCTCTTCACGGGTTATGGATACCAGGTGCGCATTGTTGAGAACCTGGATG
ACATCGACGCAGATCTCCATAGCTCTATGATGTGGGCAGTTGAGGAGATCCACAAGATCCAAAAAGCGGCGCGTTCCGGCAAGCCAATTATGAAGCCTAGATGGCCAAT
GATTGTTTTGCGCACACCGAAGGGTTGGTCAGGACCTAAAGAGCTCCACGGGTCATTCATAGAGGGATCTTTCCACTCACATCAGGTTCCTCTACCTAATGCAAAGAAGG
ATAAAGAGGAGCTTCAGGCTCTGCAGAAATGGCTGTCCTCGTATAATCCGCACGAACTTTTCACTGAGACGGGAGACATCATTGACGACATCAAGTCAGTGATCCCTCTG
GAgGACACCAAGAAGCTTGGGCAGCGAGCAGAAGCCTACAAGGGCTATAGGGCACCCGATCTCCCAGACTGGCGCAAGTTTGGCGTAGAAAAGGGCTCCCAGCAGAGCG
CTATGAAAACAATTGGAAAGTTCATTGACCAAGTGTTTACCCAAAATCCTCATGGCGTCCGTGTATTTTCGCCAGACGAGCTAGAGAGCAACAAGCTGGATGCAGCACTG
GCGCACACGGGAAGGAACTTTCAGTGGGATCAATTCTCGAATGCCAAAGGCGGCCGCGTCATCGAGGTGCTCAGTGAGCACCTGTGCCAGGGCTTTATGCAGGGATACA
CGTTGACGGGCCGGGTGGGCATTTTCCCATCGTACGAAAGCTTCTTGGGAATCATCCATACCATGATGGTGCAATATGCCAAATTTAACAAAATGGCTCAAGAGACGACC
TGGCATAAGCCGGTTAGTAGCATCAACTATATCGAAACGAGTACGTGGGCTCGTCAGGAGCACAATGGATTCTCTCACCAGAACCCCTCCTTTATCGGAGCTGTGCTCAG
GCTGAAGCCCACCGCCGCGCGAGTTTATCTGCCACCTGATGCTAACACATTTTTGACCACCCTTCACCACTGTCTCAAGTCCAAGAATTATGTCAACCTCATGGTAGGTTC
AAAACAGCCAACTCCCGTGTACTTGAGCCCCGAGGAAGCAGAGAGCCACTGCCGAGCCGGCGCATCGATCTGGAGATTCTGTAGTACCGACAATGGGCTGAACCCGGAT
GTCGTGCTGGTTGGCATTGGAGTAGAGGTGATGTTCGAGGTCATCTACGCGGCGGCCATCCTCCGCAAGCGTTGTCCAGAACTCCGGGTGCGTGTGGTCAATGTGACCGA
CTTGATGATTCTGGAGAAGGAAGGTCTACATCCACATGCATTGACGACCGAAGCTTTCGACAGTCTGTTTGGCTCGGACCGGCCGATACACTTCAACTACCACGGATACC
CGGGCGAGCTCAAAGGTCTGCTCTTTGGGCGGCCCCGCCTGGACCGAGTTTCAGTAGAAGGATACATGGAGGAAGGAAGCACGACGACGCCGTTCGATATGATGTTGCT
GAACCGCGTCTCACGATACCACGTGGCGCAGGCAGCCGTGATCGGGGCGTCCAGACGGAATGAGAAGGTTCAAGTTCGGCAGCACGAACTGGTCAGCGAATTCGGCCAC
AACATCGTGGAGACACGCAAATACATTCTGGCCAACCGCAAAGACCCGGATGATACGTATGATATGCCCTCCTTTGAAcatcatcaccaccatTAAGCTTGATCCACTTAACGT
TACTGAAATCATCAAACAGCTTGACGAATCTGGATATAAGATCGTTGGTGTCGATGTCAGCTCCGGAGTTGAGACAAATGGTGTTCAGGATCTCGATAAGATACGTTCATT
TGTCCAAGCAGCAAAGAGTGCCTTCTAGTGATTTAATAGCTCCATGTCAACAAGAATAAAACGCGTTTCGGGTTTACCTCTTCCAGATACAGCTCATCTGCAATGCATTAA
TGCATTGGACCTCGCAACCCTAGTACGCCCTTCAGGCTCCGGCGAAGCAGAAGAATAGCTTAGCAGAGTCTATTTTCATTTTCGGGAGACGAGATCAAGCAGATCAACGG
TCGTCAAGAGTCCTACGAGACTGAGGAATCCGCTCTTGGCTCCACGCGACTATATATTTGTCTCTAATTGTACTTTGACATGCTCCTCTTCTTTACTCTGATAGCTTGACTA
TGAAAATTCCGTCACCAGCCCCTGGGTTCGCAAAGATAATTGCACTGTTTCTTCCTTGAACTCTCAAGCCTACAGGACACACATTCATCGTAGGTATAAACCTCGAAAATC
ATTCCTACTAAGATGGGTATACAATAGTAACCATGCATGGTTGCCTAGTGAATGCTCCGTAACACCCAATACGCCGGCCGAAACTTTTTTACAACTCTCCTATGAGTCGTT
TACCCAGAATGCACAGGTACACTTGTTTAGAGGTAATCCTTCTTTCTAGAAGTCCTCGTGTACTGTGTAAGCGCCCACTCCACATCTCCACTCGCTCCGGGGAGACGGGCA
CAAGGTTCATGAACCGAAGAGGATGGGAACCCCGACCATGGCCTGCGGCCGCAGCTATCAGTCCTCTCAGCTGACGCCCCTCACGAACCCTCGCACTCTCCCGAAATGCT
CTTCGGTGTCCATGGCGCATCATCTCAGCAGCATCCCATGTCAAACCATGGCTTTGGGCCCACGGACTCTGTGGCCCTGCCGCAACATCACCATCACCATCGACTCCCGCC
CCATGCAGCGCTGCGCGCCCCAGGGCGTCATGGAGTGAATGTGGAATCTCCTCCTTTGCCCTCGGGCCCGCCGAGTGTGGTGGGCCAGCCTGGAATGCCTGATCCAGCCC
CCAGGCCTCGAGGACCAAAACTGAAGTTTACTCCCGAAGAGGACGCTCTACTGGTGGAGTTGAAGGAAAACAAGAACTTGACGTGGAAGCAAATTGCAGACTTCTTCCC
GGGCCGAACGAGCGGTACCTTGCAAGTCCGATACTGCACCAAGCTGAAGGCTAAGGATGTAGCTTGGAGTGACGAAATGGTTCGATTTGCTCCTGATGTATTTCTACGCC
TGTCTCACACATGCTAATGAAGGGAATAGGTACAAAGGCTGCAGCGGGCAATGCACGAGTACGAGAACGATCGGTGGCGCATCATTGCAGGGAAGGTTGGAAATGGCTT
CACCCCAGCTGCTTGCCGCGAGAAAGCCATGCAGCTCCATGAGTAAAAGCGTTGGGGAATTTTCATATTTATATCTACTGTCGCCAGATTCGGCCCTGCTTGGACCCTCTG
ATCTCCTTACTCTCCATATTGGTTCAAATGTCGGGTCACCGATAGGGCTGGTGGTGCAGGCTTGTTGTAGGCACGGGAGGATGATCAGCATAACTCTGAGTCACTATAGGG
ACGGGTTGATGTAAGGTATTAAGTGATGTATGATAATTCATTTTAGCCCGGGGGAACATATGGCGCCGGCATTTGTTCGTTCGCAATGAACCGACACTAGCGTCCGCTCTC
GCAGTTTAGCACCGGCTGATCCCGGGCTGAACGCGGCCATTGCTCGGCCGGGGCATGTGTTCCTTATCTACGGCAGACCGCAGATGACCACTGGAGCAGATTATAGACCC
TAAGCCCTAAGCCGGACACCCAATCGAGTAGGTCTGCGGACCAGGTCACTGCGGGCAGCCGGAGAAGCTCCGCAACCAATCAATCCCCGGCGCTGACTAAGGGCAGGCG
ACCACGGGCCGAAGCGGCTTCAAACTCACCTCAACCTCCAAACTCCCTCATCTCCAAACGTCCTTGCCTTGTCTGCCGTCATTGCAACCCACCCACCAGGACACATGAGCA
GCAAGTCCCAGCTCACCTACACTGCCCGTGCCAGCAAGCATCCCAATGCTCTGGCGAAGAGGCTGTTCGAGATTGCCGAGGCCAAGAAaACCAATGTGACTGTCTCGGCT
GACGTTACCACCACTAAGGAGCTACTAGATCTTGCTGACCGTCTCGGTCCCTACATTGCCGTGATCAAAACCCACATCGATATCCTCTCTGATTTCAGCAACGAGACtATTG
AGGGACTTAAGGCTCTCGCGCAGAAGCACAACTTTCTCATCTTCGAGGACCGCAAGTTCATTGACATCGGCAACACGGTCCAGAAGCAATACCACGGCGGTACCCTCCGT
ATCTCGGAATGGGCCCACATCATCAACTGCAGCATTCTCCCTGGTGAGGGTATCGTCGAGGCTCTCGCTCAGACGGCGTCTGCACCGGACTTCGCCTACGGCCCCGAACG
CGGTCTGTTGATCTTGGCAGAGATGACCTCTAAGGGCTCCTTGGCTACCGGCCAGTACACTACTTCCTCGGTCGATTATGCCCGGAAATACAAGAACTTCGTTATGGGATT
CGTGTCGACGCGCGCGTTGGGTGAGGTGCAGTCGGAAGTCAGCTCTCCTTCGGATGAGGAGGACTTTGTGGTtTTCACGACTGGTGTGAACATTAACCTCCA

>pSF503_IC
TGGAGGaAGGGATCCGCCAAGGATGCTCTGGATCCGCATCCGGCCGCTCTTGCGCCCCATCAATCGCCCGACTATAAATCGAACTACTTTCGGCATCTTCTAGACTTCCTA
ATACCGCCTAGTCATAGCAGATTCAAGCTGAGAACACCACAAGTAAATATCACCCATCATGCTTACCCTGACCGTCCCTGAAAACTACGGGTATGTGCCAATTCTACAATT
CCTTGCAGACAATGCCATTCTCCCCATGAAGTCTGATGCTAACTATCCTGCAGCTCTGTCATTGCCGTCGCTCTGGGTGCCATCCCCGTCCTGAGCTTCGTCCATGGCGCCG
TCGTGTCTCGTCTCCGCAAGGAAGCTGATTGCCCCTACCCTCACTGCTATGCGACCGTAGAGCAGTGCAAGACCAACGTAAGCCAACCTCACACAAACAGGATTCCTCGA
GCTAACATACATTCCGAACCGTGCAGCCCAAGGCCGAGCAGTTCAACTGCGCTCAGCGCGCTCATGCCAACTTCCTTGAGAACTCCAGCCAAACTATGCTCTTCCTCCTGG
TAGCTGGACTGAAGTACCCCCAGTTGGCGACTGGCCTCGGAAGCATCTGGGTCCTCGGTCGCTCACTGTTCCTTTACGGATATGTGTACTCCGGCAAGCCGCGGGGTCGCG
GTCGTTTGTACGGCAGCTTCTACTTGCTTGCACAGGGAGCTCTCTGGGGCTTGACGTCTTTTGGAGTTGCGAGGGAGTTGATTTCCTACTTCTAAGTTTGGACTGAATCCGT
GGTGTGATTGAGGTGATTGGCGATGTTTGGCTATACCAGCTATATGTAATAATCTCTACTGTATACTACTATTCAACGCATTTTACTATGCGTGCTGCTAGGGTCGGCAATG
ACAATGGCAATCTGACTGACGTGGTCTATTTCTCCATGTGCAGCAGGGAATACGAGCTCCAATGGACCTCGGGAGTGGCACAGTCAATGGCAAGGAAACTGGAGcgcaaaaaa
ccccgcccctgacagggcggggtttttcgcGATCGGAGTCGATAAGCTTCGGAGAATATGGAGCTTCATCGAATCACCGGCAGTAAGCGAAGGAGAATGTGAAGCCAGGGGTGTATAGCC
GTCGGCGAAATAGCATGCCATTAACCTAGGTACAGAAGTCCAATTGCTTCCGATCTGGTAAAAGATTCACGAGATAGTACCTTCTCCGAAGTAGGTAGAGCGAGTACCCG
GCGCGTAAGCTCCCTAATTGGCCCATCCGGCATCTGTAGGGCGTCCAAATATCGTGCCTCTCCTGCTTTGCCCGGTGTATGAAACCGGAAAGGCCGCTCAGGAGCTGGCC
AGCGGCGCAGACCGGGAACACAAGCTGGCAGTCGACCCATCCGGTGCTCTGCACTCGACCTGCTGAGGTCCCTCAGTCCCTGGTAGGCAGCTTTGCCCCGTCTGTCCGCC
CGGTGTGTCGGCGGGGTTGACAAGGTCGTTGCGTCAGTCCAACATTTGTTGCCATATTTTCCTGCTCTCCCCACCAGCTGCTCTTTTCTTTTCTCTTTCTTTTCCCATCTTCA
GTATATTCATCTTCCCATCCAAGAACCTTTATTTCCCCTAAGTAAGTACTTTGCTACATCCATACTCCATCCTTCCCATCCCTTATTCCTTTGAACCTTTCAGTTCGAGCTTTC
CCACTTCATCGCAGCTTGACTAACAGCTACCCCGCTTGAGCAGACATCACCGAATTCACCATGTCTAGACTGGACAAGAGCAAAGTCATAAACGGCGCTCTGGAATTACT
CAATGGAGTCGGTATCGAAGGCCTGACGACAAGGAAACTCGCTCAAAAGCTGGGAGTTGAGCAGCCTACCCTGTACTGGCACGTGAAGAACAAGCGGGCCCTGCTCGAT
GCCCTGCCAATCGAGATGCTGGACAGGCATCATACCCACTTCTGCCCCCTGGAAGGCGAGTCATGGCAAGACTTTCTGCGGAACAACGCCAAGTCATTCCGCTGTGCTCT
CCTCTCACATCGCGACGGGGCTAAAGTGCATCTCGGCACCCGCCCAACAGAGAAACAGTACGAAACCCTGGAAAATCAGCTCGCGTTCCTGTGTCAGCAAGGCTTCTCCC
TGGAGAACGCACTGTACGCTCTGTCCGCCGTGGGCCACTTTACACTGGGCTGCGTATTGGAGGAACAGGAGCATCAAGTAGCAAAAGAGGAAAGAGAGACACCTACCAC
CGATTCTATGCCCCCACTTCTGAGACAAGCAATTGAGCTGTTCGACCGGCAGGGAGCCGAACCTGCCTTCCTTTTCGGCCTGGAACTAATCATATGTGGCCTGGAGAAACA
GCTAAAGTGCGAAAGCGGCGGGCCGGCCGACGCCCTTGACGATTTTGACTTAGACATGCTCCCAGCCGATGCCCTTGACGACTTTGACCTTGATATGCTGCCTGCTGACGC
TCTTGACGATTTTGACCTTGACATGCTCCCCGGGTAACTAAGTAAGGATCCACTAGTACAGCAGAAGAATCTCTCTCCGCTGTTGCTTCAGTGTCTGCCATGCATTAACTTC
ATCCTACTGTCCTACCCGCAGTACCCATTCACATTTGCGGCAGATACCAGGTTGTTTTCTATTCCCTCGGTTCTTCAGTTCTTCAGATATTACATTACTCGAGAAATTGGCG
GCGATGCAGGAGTTTGGTTGGTTTTAATTGTTTCAGTCTCCTTGACCTGATTTGTATGACATGCAATGTTTCGACGGATGACTATCTCGGTGAATATACGTCATGAATCATG
CGGCCGCGCGTATCACGAGGCCCTTTCGACTTCACTCGAGTTTACCACTCCCTATCAGTGATAGAGAAAAGTGAAAGTCGAGTTTACCACTCCCTATCAGTGATAGAGAA
AAGTGAAAGTCGAGTTTACCACTCCCTATCAGTGATAGAGAAAAGTGAAAGTCGAGTTTACCACTCCCTATCAGTGATAGAGAAAAGTGAAAGTCGAGTTTACCACTCCC
TATCAGTGATAGAGAAAAGTGAAAGTCGAGTTTACCACTCCCTATCAGTGATAGAGAAAAGTGAAAGTCGAGTTTACCACTCCCTATCAGTGATAGAGAAAAGTGAAAG
TCGAGCTCCCCATCTTCAGTATATTCATCTTCCCATCCAAGAACCTTTATTTCCCCTAAGTAAGTACTTTGCTACATCCATACTCCATCCTTCCCATCCCTTATTCCTTTGAA
CCTTTCAGTTCGAGCTTTCCCACTTCATCGCAGCTTGACTAACAGCTACCCCGCTTGAGCAGACATCACCGTTTAAACACCATGGCCAGCACAATAGCACGCACTGAGGAA
CGCCAGAATGCTGGGTGAGTTTTGCGGTCTGCCTCTCTGACATCACACCCCTCCTCCCACTCCCGTCCCTCCTGCCCGCCGCCCAGACGTGAGGATTCACCACCCACGACT
CTCCATAACAAGCCCCGTCGCCCAAACATTCACTGGCAGGCTTCCCGCTTTCCATTATTCTTCAATTCGTCACCAGGATTACTCCTTCGGGCTTAACGAAAGGACTATCCTT
CTGACTCACCACCAACCCTCACTGCCCCTCCTGCATGCTGTAGCGGACTGCGGGCACCGACCTGCATCATCGATCTACACCCGATCCCTGTGACATTATATTCGTCAAGCT
ATAGCCTAGCTAACATGGATGTTTTACGTAGCACCATGGAGCTCAAAGATGACACTGTCATCATAGTACTGGGTGCCTCCGGAGATCTTGCAAAGAAGAAaACCGTCAGT
GACGACCCCCTGATTCATGTTGACCTGACAGAAAGCTAACCTTTTACAGTTCCCGGCCCTTTTCGGCCTTGTATGTCCTCTCCCAGATCCAATTGCAGTTTGACTCACCAGT
ATGGTTGCTGATTTGCGCTTCCAGTATCGCAACAAGTTCCTCCCCAAGGGAATCAAGATCGTCGGATATGCCCGGACAAACATGGACCATGAGGAGTACCTGAGGCGTGT
GCGCTCATACATCAAGACCCCTACCAAGGAAATCGAAGAGCAGCTGGACAGCTTCTGCCAGTTCTGCACCTACATTTCCGGTCAATATGACAAGGATGACTCGTTCATCA
ACCTCAACAAGCACCTCGAGGAGATTGAGAAGGGCCAGAAGGAGCAGAACAGAATCTACTACATGGCCCTCCCtCCCAGCGTTTTCACCACCGTTTCCGACCAACTTAAG
CGCAACTGCTACCCCAAGAACGGCGTTGCCCGTATCATCGTGAGTCAATCCTGGGCTGGTATCACCCTGCCATTGGTCATTATTCTTACTCGCTTGTTTTCCTATTTCACAG
GTAGAGAAGCCTTTCGGCAAGGACCTTCAGAGCTCGCGCGATCTCCAAAAAGCCCTGGAGCCTAACTGGAAGGAAGAGGAGATCTTCCGTATCGACCACTACCTGGGTA
AGGAGATGGTCAAGAACATCCTTATCATGCGCTTCGGAAACGAATTCTTCAACGCCACCTGGAACCGTCACCACATCGATAACGTTCAGGTACGACCTTGCGCTATCCAA
TTGGCCTATTGATTTACTTGCTAAATTGTCGCTTCTATCATTAGATCACATTCAAGGAGCCCTTCGGCACTGAGGGACGTGGTGGTTACTTCGATGAATTCGGCATCATCCG
TGATGTCATGCAGAACCGTACGTTCAAAGTCACGCTCGACATCTCCGACATGATGCTGATAAAAATCTCTCCTAGACCTTCTCCAGGTGTTGACGCTGCTCGCTATGGAGC
GCCCCATTTCCTTCTCCGCCGAGGACATCCGTGACGAGAAGGTACAGTGTGCGCTTGACTATTGGTTGTGCTGGGTTACTGACACTTAACCAGGTTCGTGTCCTCCGTGCG
ATGGACGCCATTGAGCCCAAGAACGTCATTATTGGCCAGTACGGAAAGTCTCTGGATGGCAGCAAGCCCGCCTACAAGGAGGACGAaACCGTTCCCCAGGATTCCCGCTG
CCCCACCTTCTGCGCTATGGTCGCCTACATCAAGAACGAGAGGTGGGACGGTGTTCCTTTCATCATGAAGGCTGGCAAGGGTATGTACCTCTTTCCAAGCGATCATAGCAC
CGATTGGTATACTAATAATTCGCAGCCTTGAACGAGCAGAAaACCGAGATCCGTATCCAGTTCCGTGACGTTACCTCCGGAATTTTCAAGGACATCCCTCGCAACGAGCTC
GTTATCCGCGTCCAGCCCAACGAGTCCGTGTACATCAAGATGAACTCCAAGCTGCCTGGCCTGTCCATGCAGACGGTTGTGACTGAGCTCGACCTCACCTACCGCCGCCGC
TTCTCCGACCTCAAGATCCCCGAAGCCTACGAGTCTCTGATCCTGGATGCTCTGAAGGGCGACCACTCCAACTTCGTCCGTGACGATGAGCTGGATGCCAGCTGGAGGAT
CTTCACCCCTCTCCTGCACTACCTGGATGACAACAAGGAGATCATCCCCATGGAATACCCCTACGGTACGTGCACTTCTTGCAATTTGTCTAAATCGCTTACATACTGACC
AACGCGCAGGCTCCCGCGGACCCGCCGTCCTTGATGACTTCACCGCGTCCTTCGGCTACAAGTTCAGCGATGCTGCTGGCTACCAGTGGCCCTTGACTTCCACCCCCAACC
GTCTGTAAGCTTGATCCACTTAACGTTACTGAAATCATCAAACAGCTTGACGAATCTGGATATAAGATCGTTGGTGTCGATGTCAGCTCCGGAGTTGAGACAAATGGTGTT
CAGGATCTCGATAAGATACGTTCATTTGTCCAAGCAGCAAAGAGTGCCTTCTAGTGATTTAATAGCTCCATGTCAACAAGAATAAAACGCGTTTCGGGTTTACCTCTTCCA
GATACAGCTCATCTGCAATGCATTAATGCATTGGACCTCGCAACCCTAGTACGCCCTTCAGGCTCCGGCGAAGCAGAAGAATAGCTTAGCAGAGTCTATTTTCATTTTCGG

GAGACGAGATCAAGCAGATCAACGGTCGTCAAGAGTCCTACGAGACTGAGGAATCCGCTCTTGGCTCCACGCGACTATATATTTGTCTCTAATTGTACTTTGACATGCTCC
TCTTCTTTACTCTGATAGCTTGACTATGAAAATTCCGTCACCAGCCCCTGGGTTCGCAAAGATAATTGCACTGTTTCTTCCTTGAACTCTCAAGCCTACAGGACACACATTC
ATCGTAGGTATAAACCTCGAAAATCATTCCTACTAAGATGGGTATACAATAGTAACCATGCATGGTTGCCTAGTGAATGCTCCGTAACACCCAATACGCCGGCCGAAACT
TTTTTACAACTCTCCTATGAGTCGTTTACCCAGAATGCACAGGTACACTTGTTTAGAGGTAATCCTTCTTTCTAGAAGTCCTCGTGTACTGTGTAAGCGCCCACTCCACATC
TCCACTCGCTCCGGGGAGACGGGCACAAGGTTCATGAACCGAAGAGGATGGGAACCCCGACCATGGCCTGCGGCCGCAGCTATCAGTCCTCTCAGCTGACGCCCCTCACG
AACCCTCGCACTCTCCCGAAATGCTCTTCGGTGTCCATGGCGCATCATCTCAGCAGCATCCCATGTCAAACCATGGCTTTGGGCCCACGGACTCTGTGGCCCTGCCGCAAC
ATCACCATCACCATCGACTCCCGCCCCATGCAGCGCTGCGCGCCCCAGGGCGTCATGGAGTGAATGTGGAATCTCCTCCTTTGCCCTCGGGCCCGCCGAGTGTGGTGGGCC
AGCCTGGAATGCCTGATCCAGCCCCCAGGCCTCGAGGACCAAAACTGAAGTTTACTCCCGAAGAGGACGCTCTACTGGTGGAGTTGAAGGAAAACAAGAACTTGACGTG
GAAGCAAATTGCAGACTTCTTCCCGGGCCGAACGAGCGGTACCTTGCAAGTCCGATACTGCACCAAGCTGAAGGCTAAGGATGTAGCTTGGAGTGACGAAATGGTTCGAT
TTGCTCCTGATGTATTTCTACGCCTGTCTCACACATGCTAATGAAGGGAATAGGTACAAAGGCTGCAGCGGGCAATGCACGAGTACGAGAACGATCGGTGGCGCATCATT
GCAGGGAAGGTTGGAAATGGCTTCACCCCAGCTGCTTGCCGCGAGAAAGCCATGCAGCTCCATGAGTAAAAGCGTTGGGGAATTTTCATATTTATATCTACTGTCGCCAG
ATTCGGCCCTGCTTGGACCCTCTGATCTCCTTACTCTCCATATTGGTTCAAATGTCGGGTCACCGATAGGGCTGGTGGTGCAGGCTTGTTGTAGGCACGGGAGGATGATCA
GCATAACTCTGAGTCACTATAGGGACGGGTTGATGTAAGGTATTAAGTGATGTATGATAATTCATTTTAGCCCGGGGGAACATATGGCGCCGGCATTTGTTCGTTCGCAAT
GAACCGACACTAGCGTCCGCTCTCGCAGTTTAGCACCGGCTGATCCCGGGCTGAACGCGGCCATTGCTCGGCCGGGGCATGTGTTCCTTATCTACGGCAGACCGCAGATG
ACCACTGGAGCAGATTATAGACCCTAAGCCCTAAGCCGGACACCCAATCGAGTAGGTCTGCGGACCAGGTCACTGCGGGCAGCCGGAGAAGCTCCGCAACCAATCAATC
CCCGGCGCTGACTAAGGGCAGGCGACCACGGGCCGAAGCGGCTTCAAACTCACCTCAACCTCCAAACTCCCTCATCTCCAAACGTCCTTGCCTTGTCTGCCGTCATTGCAA
CCCACCCACCAGGACACATGAGCAGCAAGTCCCAGCTCACCTACACTGCCCGTGCCAGCAAGCATCCCAATGCTCTGGCGAAGAGGCTGTTCGAGATTGCCGAGGCCAA
GAAaACCAATGTGACTGTCTCGGCTGACGTTACCACCACTAAGGAGCTACTAGATCTTGCTGACCGTCTCGGTCCCTACATTGCCGTGATCAAAACCCACATCGATATCCT
CTCTGATTTCAGCAACGAGACtATTGAGGGACTTAAGGCTCTCGCGCAGAAGCACAACTTTCTCATCTTCGAGGACCGCAAGTTCATTGACATCGGCAACACGGTCCAGAA
GCAATACCACGGCGGTACCCTCCGTATCTCGGAATGGGCCCACATCATCAACTGCAGCATTCTCCCTGGTGAGGGTATCGTCGAGGCTCTCGCTCAGACGGCGTCTGCACC
GGACTTCGCCTACGGCCCCGAACGCGGTCTGTTGATCTTGGCAGAGATGACCTCTAAGGGCTCCTTGGCTACCGGCCAGTACACTACTTCCTCGGTCGATTATGCCCGGAA
ATACAAGAACTTCGTTATGGGATTCGTGTCGACGCGCGCGTTGGGTGAGGTGCAGTCGGAAGTCAGCTCTCCTTCGGATGAGGAGGACTTTGTGGTtTTCACGACTGGTGT
GAACATTAACCTCCA

>pSF531_IC

TGGAGGaAGGGATCCGCCAAGGATGCTCTGGATCCGCATCCGGCCGCTCTTGCGCCCCATCAATCGCCCGACTATAAATCGAACTACTTTCGGCATCTTCTAGACTTCCTA
ATACCGCCTAGTCATAGCAGATTCAAGCTGAGAACACCACAAGTAAATATCACCCATCATGCTTACCCTGACCGTCCCTGAAAACTACGGGTATGTGCCAATTCTACAATT
CCTTGCAGACAATGCCATTCTCCCCATGAAGTCTGATGCTAACTATCCTGCAGCTCTGTCATTGCCGTCGCTCTGGGTGCCATCCCCGTCCTGAGCTTCGTCCATGGCGCCG
TCGTGTCTCGTCTCCGCAAGGAAGCTGATTGCCCCTACCCTCACTGCTATGCGACCGTAGAGCAGTGCAAGACCAACGTAAGCCAACCTCACACAAACAGGATTCCTCGA
GCTAACATACATTCCGAACCGTGCAGCCCAAGGCCGAGCAGTTCAACTGCGCTCAGCGCGCTCATGCCAACTTCCTTGAGAACTCCAGCCAAACTATGCTCTTCCTCCTGG
TAGCTGGACTGAAGTACCCCCAGTTGGCGACTGGCCTCGGAAGCATCTGGGTCCTCGGTCGCTCACTGTTCCTTTACGGATATGTGTACTCCGGCAAGCCGCGGGGTCGCG
GTCGTTTGTACGGCAGCTTCTACTTGCTTGCACAGGGAGCTCTCTGGGGCTTGACGTCTTTTGGAGTTGCGAGGGAGTTGATTTCCTACTTCTAAGTTTGGACTGAATCCGT
GGTGTGATTGAGGTGATTGGCGATGTTTGGCTATACCAGCTATATGTAATAATCTCTACTGTATACTACTATTCAACGCATTTTACTATGCGTGCTGCTAGGGTCGGCAATG
ACAATGGCAATCTGACTGACGTGGTCTATTTCTCCATGTGCAGCAGGGAATACGAGCTCCAATGGACCTCGGGAGTGGCACAGTCAATGGCAAGGAAACTGGAGcgcaaaaaa
ccccgcccctgacagggcggggttttttcgcGATCGGAGTCGATAAGCTTCGGAGAATATGGAGCTTCATCGAATCACCGGCAGTAAGCGAAGGAGAATGTGAAGCCAGGGGTGTATAGCC
GTCGGCGAAATAGCATGCCATTAACCTAGGTACAGAAGTCCAATTGCTTCCGATCTGGTAAAAGATTCACGAGATAGTACCTTCTCCGAAGTAGGTAGAGCGAGTACCCG
GCGCGTAAGCTCCCTAATTGGCCCATCCGGCATCTGTAGGGCGTCCAAATATCGTGCCTCTCCTGCTTTGCCCGGTGTATGAAACCGGAAAGGCCGCTCAGGAGCTGGCC
AGCGGCGCAGACCGGGAACACAAGCTGGCAGTCGACCCATCCGGTGCTCTGCACTCGACCTGCTGAGGTCCCTCAGTCCCTGGTAGGCAGCTTTGCCCCGTCTGTCCGCC
CGGTGTGTCGGCGGGGTTGACAAGGTCGTTGCGTCAGTCCAACATTTGTTGCCATATTTTCCTGCTCTCCCCACCAGCTGCTCTTTTCTTTTCTCTTTCTTTTCCCATCTTCA
GTATATTCATCTTCCCATCCAAGAACCTTTATTTCCCCTAAGTAAGTACTTTGCTACATCCATACTCCATCCTTCCCATCCCTTATTCCTTTGAACCTTTCAGTTCGAGCTTTC
CCACTTCATCGCAGCTTGACTAACAGCTACCCCGCTTGAGCAGACATCACCGAATTCACCATGTCTAGACTGGACAAGAGCAAAGTCATAAACGGCGCTCTGGAATTACT
CAATGGAGTCGGTATCGAAGGCCTGACGACAAGGAAACTCGCTCAAAAGCTGGGAGTTGAGCAGCCTACCCTGTACTGGCACGTGAAGAACAAGCGGGCCCTGCTCGAT
GCCCTGCCAATCGAGATGCTGGACAGGCATCATACCCACTTCTGCCCCCTGGAAGGCGAGTCATGGCAAGACTTTCTGCGGAACAACGCCAAGTCATTCCGCTGTGCTCT
CCTCTCACATCGCGACGGGGCTAAAGTGCATCTCGGCACCCGCCCAACAGAGAAACAGTACGAAACCCTGGAAAATCAGCTCGCGTTCCTGTGTCAGCAAGGCTTCTCCC
TGGAGAACGCACTGTACGCTCTGTCCGCCGTGGGCCACTTTACACTGGGCTGCGTATTGGAGGAACAGGAGCATCAAGTAGCAAAAGAGGAAAGAGAGACACCTACCAC
CGATTCTATGCCCCCACTTCTGAGACAAGCAATTGAGCTGTTCGACCGGCAGGGAGCCGAACCTGCCTTCCTTTTCGGCCTGGAACTAATCATATGTGGCCTGGAGAAACA
GCTAAAGTGCGAAAGCGGCGGGCCGGCCGACGCCCTTGACGATTTTGACTTAGACATGCTCCCAGCCGATGCCCTTGACGACTTTGACCTTGATATGCTGCCTGCTGACGC
TCTTGACGATTTTGACCTTGACATGCTCCCCGGGTAACTAAGTAAGGATCCACTAGTACAGCAGAAGAATCTCTCTCCGCTGTTGCTTCAGTGTCTGCCATGCATTAACTTC
ATCCTACTGTCCTACCCGCAGTACCCATTCACATTTGCGGCAGATACCAGGTTGTTTTCTATTCCCTCGGTTCTTCAGTTCTTCAGATATTACATTACTCGAGAAATTGGCG
GCGATGCAGGAGTTTGGTTGGTTTTAATTGTTTCAGTCTCCTTGACCTGATTTGTATGACATGCAATGTTTCGACGGATGACTATCTCGGTGAATATACGTCATGAATCATG
CGGCCGCGCGTATCACGAGGCCCTTTCGACTTCACTCGAGTTTACCACTCCCTATCAGTGATAGAGAAAAGTGAAAGTCGAGTTTACCACTCCCTATCAGTGATAGAGAA
AAGTGAAAGTCGAGTTTACCACTCCCTATCAGTGATAGAGAAAAGTGAAAGTCGAGTTTACCACTCCCTATCAGTGATAGAGAAAAGTGAAAGTCGAGTTTACCACTCCC
TATCAGTGATAGAGAAAAGTGAAAGTCGAGTTTACCACTCCCTATCAGTGATAGAGAAAAGTGAAAGTCGAGTTTACCACTCCCTATCAGTGATAGAGAAAAGTGAAAG
TCGAGCTCCCCATCTTCAGTATATTCATCTTCCCATCCAAGAACCTTTATTTCCCCTAAGTAAGTACTTTGCTACATCCATACTCCATCCTTCCCATCCCTTATTCCTTTGAA
CCTTTCAGTTCGAGCTTTCCCACTTCATCGCAGCTTGACTAACAGCTACCCCGCTTGAGCAGACATCACCGTTTAAACACCATGCCTGGAGAGGTCATCGACAGGCCAAAT
CCCAAGGCTGAGCCTTCACACATCCCCGATCTTGTCAATCAATTGCAGGTCAAACTTCAAGAGACGCGTTTGGAGGAAACTGATTACAATGCCCTTCTGAAATTCCGCCGT
GCAGCGGCCTACATTGCTGCTGCAATGATCTTTCTCCAAGACAATGTGCTGCTGAAGCAGAATCTAAGGCACGAGGACATCAAGCCCAGGCTTCTTGGCCACTGGGGAAC
ATGTCCCGGGTTGATTCTTGTATACTCTCACTTGAACTACATCATCAGAAAGCAGAACCTGGATATGTTGTATGTCGTCGGGCCTGGCCACGGCGCGCCAGCTATTTTGGC
CTCACTGTGGCTTGAGGGCTCTTTAGAGAAATTCTACCCCCACTACTCACGAGACATGGATGGcCTCCATGAGCTCATCTCGACCTTCAGCACAAGTGCTGGATTACCAAG
CCATATCAATGCGGAAACTCCCGGTGCAATCCATGAAGGTGGTGAATTGGGTTATGCGTTAGCTGTCTCTTTTGGTGCTGTTATGGACAATCCCGACATGATCGTCACCTG
CGTGGTTGGTGACGGGGAAGCAGAAACTGGTCCTACCGCGACGTCCTGGCATGCAATCAAGTACATTGACCCCGCAGAATCAGGTGCCGTCCTGCCGATTCTCCACGTTA
ATGGCTTTAAGATCAGCGAGCGCACCATTTATGGCTGCATGGACAACAAAGAGCTGGTgTCCCTCTTCACGGGTTATGGATACCAGGTGCGCATTGTTGAGAACCTGGATG
ACATCGACGCAGATCTCCATAGCTCTATGATGTGGGCAGTTGAGGAGATCCACAAGATCCAAAAAGCGGCGCGTTCCGGCAAGCCAATTATGAAGCCTAGATGGCCAAT
GATTGTTTTGCGCACACCGAAGGGTTGGTCAGGACCTAAAGAGCTCCACGGGTCATTCATAGAGGGATCTTTCCACTCACATCAGGTTCCTCTACCTAATGCAAAGAAGG
ATAAAGAGGAGCTTCAGGCTCTGCAGAAATGGCTGTCCTCGTATAATCCGCACGAACTTTTCACTGAGACGGGAGACATCATTGACGACATCAAGTCAGTGATCCCTCTG
GAgGACACCAAGAAGCTTGGGCAGCGAGCAGAAGCCTACAAGGGCTATAGGGCACCCGATCTCCCAGACTGGCGCAAGTTTGGCGTAGAAAAGGGCTCCCAGCAGAGCG
CTATGAAAACAATTGGAAAGTTCATTGACCAAGTGTTTACCCAAAATCCTCATGGCGTCCGTGTATTTTCGCCAGACGAGCTAGAGAGCAACAAGCTGGATGCAGCACTG
GCGCACACGGGAAGGAACTTTCAGTGGGATCAATTCTCGAATGCCAAAGGCGGCCGCGTCATCGAGGTGCTCAGTGAGCACCTGTGCCAGGGCTTTATGCAGGGATACA
CGTTGACGGGCCGGGTGGGCATTTTCCCATCGTACGAAAGCTTCTTGGGAATCATCCATACCATGATGGTGCAATATGCCAAATTTAACAAA ATGGCTCAAGAGACGACC
TGGCATAAGCCGGTTAGTAGCATCAACTATATCGAAACGAGTACGTGGGCTCGTCAGGAGCACAATGGATTCTCTCACCAGAACCCCTCCTTTATCGGAGCTGTGCTCAG
GCTGAAGCCCACCGCCGCGCGAGTTTATCTGCCACCTGATGCTAACACATTTTTGACCACCCTTCACCACTGTCTCAAGTCCAAGAATTATGTCAACCTCATGGTAGGTTC
AAAACAGCCAACTCCCGTGTACTTGAGCCCCGAGGAAGCAGAGAGCCACTGCCGAGCCGGCGCATCGATCTGGAGATTCTGTAGTACCGACAATGGGCTGAACCCGGAT
GTCGTGCTGGTTGGCATTGGAGTAGAGGTGATGTTCGAGGTCATCTACGCGGCGGCCATCCTCCGCAAGCGTTGTCCAGAACTCCGGGTGCGTGTGGTCAATGTGACCGA
CTTGATGATTCTGGAGAAGGAAGGTCTACATCCACATGCATTGACGACCGAAGCTTTCGACAGTCTGTTTGGCTCGGACCGGCCGATACACTTCAACTACCACGGATACC
CGGGCGAGCTCAAAGGTCTGCTCTTTGGGCGGCCCCGCCTGGACCGAGTTTCAGTAGAAGGATACATGGAGGAAGGAAGCACGACGACGCCGTTCGATATGATGTTGCT
GAACCGCGTCTCACGATACCACGTGGCGCAGGCAGCCGTGATCGGGGCGTCCAGACGGAATGAGAAGGTTCAAGTTCGGCAGCACGAACTGGTCAGCGAATTCGGCCAC
AACATCGTGGAGACACGCAAATACATTCTGGCCAACCGCAAAGACCCGGATGATACGTATGATATGCCCTCCTTTGAATAAGCTTGATCCACTTAACGTTACTGAAATCA
TCAAACAGCTTGACGAATCTGGATATAAGATCGTTGGTGTCGATGTCAGCTCCGGAGTTGAGACAAATGGTGTTCAGGATCTCGATAAGATACGTTCATTTGTCCAAGCA
GCAAAGAGTGCCTTCTAGTGATTTAATAGCTCCATGTCAACAAGAATAAAACGCGTTTCGGGTTTACCTCTTCCAGATACAGCTCATCTGCAATGCATTAATGCATTGGAC
CTCGCAACCCTAGTACGCCCTTCAGGCTCCGGCGAAGCAGAAGAATAGCTTAGCAGAGTCTATTTTCATTTTCGGGAGACGAGATCAAGCAGATCAACGGTCGTCAAGAG
TCCTACGAGACTGAGGAATCCGCTCTTGGCTCCACGCGACTATATATTTGTCTCTAATTGTACTTTGACATGCTCCTCTTCTTTACTCTGATAGCTTGACTATGAAAATTCC
GTCACCAGCCCCTGGGTTCGCAAAGATAATTGCACTGTTTCTTCCTTGAACTCTCAAGCCTACAGGACACACATTCATCGTAGGTATAAACCTCGAAAATCATTCCTACTA
AGATGGGTATACAATAGTAACCATGCATGGTTGCCTAGTGAATGCTCCGTAACACCCAATACGCCGGCCGAAACTTTTTTACAACTCTCCTATGAGTCGTTTACCCAGAAT
GCACAGGTACACTTGTTTAGAGGTAATCCTTCTTTCTAGAAGTCCTCGTGTACTGTGTAAGCGCCCACTCCACATCTCCACTCGCTCCGGGGAGACGGGCACAAGGTTCAT
GAACCGAAGAGGATGGGAACCCCGACCATGGCCTGCGGCCGCAGCTATCAGTCCTCTCAGCTGACGCCCCTCACGAACCCTCGCACTCTCCCGAAATGCTCTTCGGTGTC
CATGGCGCATCATCTCAGCAGCATCCCATGTCAAACCATGGCTTTGGGCCCACGGACTCTGTGGCCCTGCCGCAACATCACCATCACCATCGACTCCCGCCCCATGCAGCG
CTGCGCGCCCCAGGGCGTCATGGAGTGAATGTGGAATCTCCTCCTTTGCCCTCGGGCCCGCCGAGTGTGGTGGGCCAGCCTGGAATGCCTGATCCAGCCCCCAGGCCTCG
AGGACCAAAACTGAAGTTTACTCCCGAAGAGGACGCTCTACTGGTGGAGTTGAAGGAAAACAAGAACTTGACGTGGAAGCAAATTGCAGACTTCTTCCCGGGCCGAACG
AGCGGTACCTTGCAAGTCCGATACTGCACCAAGCTGAAGGCTAAGGATGTAGCTTGGAGTGACGAAATGGTTCGATTTGCTCCTGATGTATTTCTACGCCTGTCTCACACA
TGCTAATGAAGGGAATAGGTACAAAGGCTGCAGCGGGCAATGCACGAGTACGAGAACGATCGGTGGCGCATCATTGCAGGGAAGGTTGGAAATGGCTTCACCCCAGCTG
CTTGCCGCGAGAAAGCCATGCAGCTCCATGAGTAAAAGCGTTGGGGAATTTTCATATTTATATCTACTGTCGCCAGATTCGGCCCTGCTTGGACCCTCTGATCTCCTTACTC
TCCATATTGGTTCAAATGTCGGGTCACCGATAGGGCTGGTGGTGCAGGCTTGTTGTAGGCACGGGAGGATGATCAGCATAACTCTGAGTCACTATAGGGACGGGTTGATG
TAAGGTATTAAGTGATGTATGATAATTCATTTTAGCCCGGGGGAACATATGGCGCCGGCATTTGTTCGTTCGCAATGAACCGACACTAGCGTCCGCTCTCGCAGTTTAGCA
CCGGCTGATCCCGGGCTGAACGCGGCCATTGCTCGGCCGGGGCATGTGTTCCTTATCTACGGCAGACCGCAGATGACCACTGGAGCAGATTATAGACCCTAAGCCCTAAG
CCGGACACCCAATCGAGTAGGTCTGCGGACCAGGTCACTGCGGGCAGCCGGAGAAGCTCCGCAACCAATCAATCCCCGGCGCTGACTAAGGGCAGGCGACCACGGGCCG
AAGCGGCTTCAAACTCACCTCAACCTCCAAACTCCCTCATCTCCAAACGTCCTTGCCTTGTCTGCCGTCATTGCAACCCACCCACCAGGACACATGAGCAGCAAGTCCCAG
CTCACCTACACTGCCCGTGCCAGCAAGCATCCCAATGCTCTGGCGAAGAGGCTGTTCGAGATTGCCGAGGCCAAGAAaACCAATGTGACTGTCTCGGCTGACGTTACCAC
CACTAAGGAGCTACTAGATCTTGCTGACCGTCTCGGTCCCTACATTGCCGTGATCAAAACCCACATCGATATCCTCTCTGATTTCAGCAACGAGACtATTGAGGGACTTAA
GGCTCTCGCGCAGAAGCACAACTTTCTCATCTTCGAGGACCGCAAGTTCATTGACATCGGCAACACGGTCCAGAAGCAATACCACGGCGGTACCCTCCGTATCTCGGAAT
GGGCCCACATCATCAACTGCAGCATTCTCCCTGGTGAGGGTATCGTCGAGGCTCTCGCTCAGACGGCGTCTGCACCGGACTTCGCCTACGGCCCCGAACGCGGTCTGTTGA
TCTTGGCAGAGATGACCTCTAAGGGCTCCTTGGCTACCGGCCAGTACACTACTTCCTCGGTCGATTATGCCCGGAAATACAAGAACTTCGTTATGGGATTCGTGTCGACGC
GCGCGTTGGGTGAGGTGCAGTCGGAAGTCAGCTCTCCTTCGGATGAGGAGGACTTTGTGGTtTTCACGACTGGTGTGAACATTAACCTCCA

>pSF528_IC

TGGAGGaAGGGATCCGCCAAGGATGCTCTGGATCCGCATCCGGCCGCTCTTGCGCCCCATCAATCGCCCGACTATAAATCGAACTACTTTCGGCATCTTCTAGACTTCCTA
ATACCGCCTAGTCATAGCAGATTCAAGCTGAGAACACCACAAGTAAATATCACCCATCATGCTTACCCTGACCGTCCCTGAAAACTACGGGTATGTGCCAATTCTACAATT
CCTTGCAGACAATGCCATTCTCCCCATGAAGTCTGATGCTAACTATCCTGCAGCTCTGTCATTGCCGTCGCTCTGGGTGCCATCCCCGTCCTGAGCTTCGTCCATGGCGCCG
TCGTGTCTCGTCTCCGCAAGGAAGCTGATTGCCCCTACCCTCACTGCTATGCGACCGTAGAGCAGTGCAAGACCAACGTAAGCCAACCTCACACAAACAGGATTCCTCGA
GCTAACATACATTCCGAACCGTGCAGCCCAAGGCCGAGCAGTTCAACTGCGCTCAGCGCGCTCATGCCAACTTCCTTGAGAACTCCAGCCAAACTATGCTCTTCCTCCTGG
TAGCTGGACTGAAGTACCCCCAGTTGGCGACTGGCCTCGGAAGCATCTGGGTCCTCGGTCGCTCACTGTTCCTTTACGGATATGTGTACTCCGGCAAGCCGCGGGGTCGCG
GTCGTTTGTACGGCAGCTTCTACTTGCTTGCACAGGGAGCTCTCTGGGGCTTGACGTCTTTTGGAGTTGCGAGGGAGTTGATTTCCTACTTCTAAGTTTGGACTGAATCCGT
GGTGTGATTGAGGTGATTGGCGATGTTTGGCTATACCAGCTATATGTAATAATCTCTACTGTATACTACTATTCAACGCATTTTACTATGCGTGCTGCTAGGGTCGGCAATG
ACAATGGCAATCTGACTGACGTGGTCTATTTCTCCATGTGCAGCAGGGAATACGAGCTCCAATGGACCTCGGGAGTGGCACAGTCAATGGCAAGGAAACTGGAGcgcaaaaaa
ccccgcccctgacagggcggggtttttcgcGATCGGAGTCGATAAGCTTCGGAGAATATGGAGCTTCATCGAATCACCGGCAGTAAGCGAAGGAGAATGTGAAGCCAGGGGTGTATAGCC
GTCGGCGAAATAGCATGCCATTAACCTAGGTACAGAAGTCCAATTGCTTCCGATCTGGTAAAAGATTCACGAGATAGTACCTTCTCCGAAGTAGGTAGAGCGAGTACCCG
GCGCGTAAGCTCCCTAATTGGCCCATCCGGCATCTGTAGGGCGTCCAAATATCGTGCCTCTCCTGCTTTGCCCGGTGTATGAAACCGGAAAGGCCGCTCAGGAGCTGGCC
AGCGGCGCAGACCGGGAACACAAGCTGGCAGTCGACCCATCCGGTGCTCTGCACTCGACCTGCTGAGGTCCCTCAGTCCCTGGTAGGCAGCTTTGCCCCGTCTGTCCGCC
CGGTGTGTCGGCGGGGTTGACAAGGTCGTTGCGTCAGTCCAACATTTGTTGCCATATTTTCCTGCTCTCCCCACCAGCTGCTCTTTTCTTTTCTCTTTCTTTTCCCATCTTCA
GTATATTCATCTTCCCATCCAAGAACCTTTATTTCCCCTAAGTAAGTACTTTGCTACATCCATACTCCATCCTTCCCATCCCTTATTCCTTTGAACCTTTCAGTTCGAGCTTTC
CCACTTCATCGCAGCTTGACTAACAGCTACCCCGCTTGAGCAGACATCACCGAATTCACCATGTCTAGACTGGACAAGAGCAAAGTCATAAACGGCGCTCTGGAATTACT
CAATGGAGTCGGTATCGAAGGCCTGACGACAAGGAAACTCGCTCAAAAGCTGGGAGTTGAGCAGCCTACCCTGTACTGGCACGTGAAGAACAAGCGGGCCCTGCTCGAT
GCCCTGCCAATCGAGATGCTGGACAGGCATCATACCCACTTCTGCCCCCTGGAAGGCGAGTCATGGCAAGACTTTCTGCGGAACAACGCCAAGTCATTCCGCTGTGCTCT
CCTCTCACATCGCGACGGGGCTAAAGTGCATCTCGGCACCCGCCCAACAGAGAAACAGTACGAAACCCTGGAAAATCAGCTCGCGTTCCTGTGTCAGCAAGGCTTCTCCC
TGGAGAACGCACTGTACGCTCTGTCCGCCGTGGGCCACTTTACACTGGGCTGCGTATTGGAGGAACAGGAGCATCAAGTAGCAAAAGAGGAAAGAGAGACACCTACCAC
CGATTCTATGCCCCCACTTCTGAGACAAGCAATTGAGCTGTTCGACCGGCAGGGAGCCGAACCTGCCTTCCTTTTCGGCCTGGAACTAATCATATGTGGCCTGGAGAAACA
GCTAAAGTGCGAAAGCGGCGGGCCGGCCGACGCCCTTGACGATTTTGACTTAGACATGCTCCCAGCCGATGCCCTTGACGACTTTGACCTTGATATGCTGCCTGCTGACGC
TCTTGACGATTTTGACCTTGACATGCTCCCCGGGTAACTAAGTAAGGATCCACTAGTACAGCAGAAGAATCTCTCTCCGCTGTTGCTTCAGTGTCTGCCATGCATTAACTTC
ATCCTACTGTCCTACCCGCAGTACCCATTCACATTTGCGGCAGATACCAGGTTGTTTTCTATTCCCTCGGTTCTTCAGTTCTTCAGATATTACATTACTCGAGAAATTGGCG
GCGATGCAGGAGTTTGGTTGGTTTTAATTGTTTCAGTCTCCTTGACCTGATTTGTATGACATGCAATGTTTCGACGGATGACTATCTCGGTGAATATACGTCATGAATCATG
CGGCCGCGCGTATCACGAGGCCCTTTCGACTTCACTCGAGTTTACCACTCCCTATCAGTGATAGAGAAAAGTGAAAGTCGAGTTTACCACTCCCTATCAGTGATAGAGAA
AAGTGAAAGTCGAGTTTACCACTCCCTATCAGTGATAGAGAAAAGTGAAAGTCGAGTTTACCACTCCCTATCAGTGATAGAGAAAAGTGAAAGTCGAGTTTACCACTCCC
TATCAGTGATAGAGAAAAGTGAAAGTCGAGTTTACCACTCCCTATCAGTGATAGAGAAAAGTGAAAGTCGAGTTTACCACTCCCTATCAGTGATAGAGAAAAGTGAAAG
TCGAGCTCCCCATCTTCAGTATATTCATCTTCCCATCCAAGAACCTTTATTTCCCCTAAGTAAGTACTTTGCTACATCCATACTCCATCCTTCCCATCCCTTATTCCTTTGAA
CCTTTCAGTTCGAGCTTTCCCACTTCATCGCAGCTTGACTAACAGCTACCCCGCTTGAGCAGACATCACCGTTTAAACACCATGCCATCGGATTCGAATGATCAGAGCATC
TCCGCTTATGGAGCTGCTCGCTCCACGGTCAAGGGCCAGAACCTTGACCCGGAAGAAGTCCGGAAAATGGACGCCTACTTTCGCGCCAGCATGTACCTATGCTTAGGCAT
GCTCTACCTGCGCGAAAATGTGCTTCTGAAACAGCCATTGAAGGTGGAGCATCTCAAAGCACGCCTACTCGGCCACTGGGGCTCAGACGCAGGACAATCCTTCACCTGGA
TTCACATGAACCGGCTGATCAAGAAGTATGATCTGGACGTTCTCTTCATCTCAGGCCCGGGTCACGGTGCCCCTGGCATTCTTTCACAGTCCTACCTGGAGGGTGTATACT
CCGAAGTCTACCCGGACAAGTCGGAGGACGAGCGAGGAATGCAAAGGTTCTTCAAGCAGTTTTCTTTCCCCGGCGGTATCGGCAGCCACGCTACCCCAGAAACCCCCGGT
AGTCTcCACGAGGGCGGGGAGCTGGGTTATTCCATCTCGCACGCCTTTGGCACCGTgTTCGATCATCCCAATCTGATTACCCTTACCATGGTGGGCGATGGTGAGGCCGAG
ACTGGGCCACTAGCTACCAGCTGGCACAGCACCAAGTACCTTAATCCCTGCACCGACGGTGCCGTTCTGCCCGTGCTCCATCTCAACGGCTACAAGATCAACAATCCGAC
ACTCCTCGCACGCATCTCCCATGATGAGCTTTCTGCTCTCATGAAGGGTTACGGCTGGACCCCCTACTTTGTAGAGGGCAGTGACCGCGAAACCATGCATCAAGCCATGGC
AGCCACGCTCGAGCACTGTGTTCTTGAAATCAGGAAATTCCAGAAGAAAGCCCGCGAGTCGAAGGAGCCCTTCCGCCCACACTGGCCCATGATCATACTGCGCAGCCCAA
AGGGTTGGTCCGCCCCGCGTGAAGTCGATGGCAAACTCCTTGAGGGCTTTTGGCGTGCCCACCAAATCCCTATCACCGACGTCCTCACCAATCCTTCACATCTTCAACTTC
TTGAATCGTGGATGAAGAGCTATAAGCCCGAGGAGCTCTTCACTCACGACGGCCGTTTGATCTCCGAACTCAAAGCCCTCGCTCCAACTGGCAATTCCCGTATGAGTGCC
AATCCGGTGGGCAATGGTGGGCTGCTACGGCGACCGCTGGACCTACCCGACTTCCGCAAGTACGCACTCACATCCATCGATCCGGGAGCCACCATCCGCGGCAGCATGGT
CAACATGTCACACTACCTCCGTGATGTGGTCGCCTTCAATCAGACCAACTTCCGTGTATTCGGGCCGGACGAAACTGAATCCAACAAGCTCTCCGAGATCTACAAAGCCG
GCAAGAAAGTCTGGCTGGCCGAATACTTCCCTGAAGATAACAACGGTGGAAACCTTTCCATGGCCGGTCGAGTGATGGAGATGCTTAGTGAACACACATGTGAAGGCTG
GCTAGAGGGCTACGTCCTCTCAGGCCGCCATGGGCTCCTTAATAGCTACGAGCCCTTCATCCACATCATCGACTCTATGGTGAACCAGCACTGCAAGTGGATCGAGAAAT
GCCTCGAAGTCGAATGGCGCGCCAAAGTGGCATCTTTGAACATCCTCCTCACCGCGACCGTCTGGCGCCAAGACCACAACGGATTCACTCACCAAGACCCAGGCTTCCTC
GATGTCGTGGCTAACAAGTCTCCCGAAGTCGTGCGCATCTACCTCCCGCCCGACGGAAACTCTCTCCTCTCCGTAATGGACCACTGCTTCCGCAGCGCCAACTACGTTAAC
GTCATTGTCGCCGATAAACAAGACCACATCCAATTCATGGACATGGACGCAGCAATTGCCCACTGCACCAAGGGCGTCGGGATCTGGGACTGGGCCAGTAACGACCAGG
GCGCGGAACCAGACGTTGTCATGGCCGCATGCGGCGACGTTCCAACCCACGAGGCACTAGCCGCCACAGCGCTCCTACGCGAACACCTTCCCCAGCTGAAAGTGCGCTTC
GTCAACGTGGTCGATCTTTTCAAACTCATGTCCAAGATCCATCACCCGCATGGAATGTCCGATCGGGAATGGAAGGCTATCTTCACGGCTGATAGGCCGATTGTgTTCAAT
TTCCACTCGTACCCGTGGCTTATTCACCGGTTGACGTATAAGcGACCCGGACAGGAGAATATCCATGTGAGGGGGTACAAGGAGAAGGGGAACATCGATACGCCATTCGA
GCTGGCGGTGAGGAATCAGACGGATCGGTATAGTTTGGCCGTTGATGCGATTGATCATGCCCGCGGACTGGGCAATACGGCGTCCGGAGTGAGAGAGAAGTTCCTGAAT
ATGCAGTTATTGGCCAAGCAGAAGGCATACGATGACGGCATTGATCCGGACTATATTCGGAACTGGACCTGGCAGTATCCGCGGAAGAAGGGGGAGGGCGTCcatcatcaccac
caccatTGAGCTTGATCCACTTAACGTTACTGAAATCATCAAACAGCTTGACGAATCTGGATATAAGATCGTTGGTGTCGATGTCAGCTCCGGAGTTGAGACAAATGGTGTTC
AGGATCTCGATAAGATACGTTCATTTGTCCAAGCAGCAAAGAGTGCCTTCTAGTGATTTAATAGCTCCATGTCAACAAGAATAAAACGCGTTTCGGGTTTACCTCTTCCAG
ATACAGCTCATCTGCAATGCATTAATGCATTGGACCTCGCAACCCTAGTACGCCCTTCAGGCTCCGGCGAAGCAGAAGAATAGCTTAGCAGAGTCTATTTTCATTTTCGGG
AGACGAGATCAAGCAGATCAACGGTCGTCAAGAGTCCTACGAGACTGAGGAATCCGCTCTTGGCTCCACGCGACTATATATTTGTCTCTAATTGTACTTTGACATGCTCCT
CTTCTTTACTCTGATAGCTTGACTATGAAAATTCCGTCACCAGCCCCTGGGTTCGCAAAGATAATTGCACTGTTTCTTCCTTGAACTCTCAAGCCTACAGGACACACATTCA
TCGTAGGTATAAACCTCGAAAATCATTCCTACTAAGATGGGTATACAATAGTAACCATGCATGGTTGCCTAGTGAATGCTCCGTAACACCCAATACGCCGGCCGAAACTT
TTTTACAACTCTCCTATGAGTCGTTTACCCAGAATGCACAGGTACACTTGTTTAGAGGTAATCCTTCTTTCTAGAAGTCCTCGTGTACTGTGTAAGCGCCCACTCCACATCT
CCACTCGCTCCGGGGAGACGGGCACAAGGTTCATGAACCGAAGAGGATGGGAACCCCGACCATGGCCTGCGGCCGCAGCTATCAGTCCTCTCAGCTGACGCCCCTCACG
AACCCTCGCACTCTCCCGAAATGCTCTTCGGTGTCCATGGCGCATCATCTCAGCAGCATCCCATGTCAAACCATGGCTTTGGGCCCACGGACTCTGTGGCCCTGCCGCAAC
ATCACCATCACCATCGACTCCCGCCCCATGCAGCGCTGCGCGCCCCAGGGCGTCATGGAGTGAATGTGGAATCTCCTCCTTTGCCCTCGGGCCCGCCGAGTGTGGTGGGCC
AGCCTGGAATGCCTGATCCAGCCCCCAGGCCTCGAGGACCAAAACTGAAGTTTACTCCCGAAGAGGACGCTCTACTGGTGGAGTTGAAGGAAAACAAGAACTTGACGTG
GAAGCAAATTGCAGACTTCTTCCCGGGCCGAACGAGCGGTACCTTGCAAGTCCGATACTGCACCAAGCTGAAGGCTAAGGATGTAGCTTGGAGTGACGAAATGGTTCGAT
TTGCTCCTGATGTATTTCTACGCCTGTCTCACACATGCTAATGAAGGGAATAGGTACAAAGGCTGCAGCGGGCAATGCACGAGTACGAGAACGATCGGTGGCGCATCATT
GCAGGGAAGGTTGGAAATGGCTTCACCCCAGCTGCTTGCCGCGAGAAAGCCATGCAGCTCCATGAGTAAAAGCGTTGGGGAATTTTCATATTTATATCTACTGTCGCCAG
ATTCGGCCCTGCTTGGACCCTCTGATCTCCTTACTCTCCATATTGGTTCAAATGTCGGGTCACCGATAGGGCTGGTGGTGCAGGCTTGTTGTAGGCACGGGAGGATGATCA
GCATAACTCTGAGTCACTATAGGGACGGGTTGATGTAAGGTATTAAGTGATGTATGATAATTCATTTTAGCCCGGGGGAACATATGGCGCCGGCATTTGTTCGTTCGCAAT
GAACCGACACTAGCGTCCGCTCTCGCAGTTTAGCACCGGCTGATCCCGGGCTGAACGCGGCCATTGCTCGGCCGGGGCATGTGTTCCTTATCTACGGCAGACCGCAGATG
ACCACTGGAGCAGATTATAGACCCTAAGCCCTAAGCCGGACACCCAATCGAGTAGGTCTGCGGACCAGGTCACTGCGGGCAGCCGGAGAAGCTCCGCAACCAATCAATC
CCCGGCGCTGACTAAGGGCAGGCGACCACGGGCCGAAGCGGCTTCAAACTCACCTCAACCTCCAAACTCCCTCATCTCCAAACGTCCTTGCCTTGTCTGCCGTCATTGCAA
CCCACCCACCAGGACACATGAGCAGCAAGTCCCAGCTCACCTACACTGCCCGTGCCAGCAAGCATCCCAATGCTCTGGCGAAGAGGCTGTTCGAGATTGCCGAGGCCAA
GAAaACCAATGTGACTGTCTCGGCTGACGTTACCACCACTAAGGAGCTACTAGATCTTGCTGACCGTCTCGGTCCCTACATTGCCGTGATCAAAACCCACATCGATATCCT
CTCTGATTTCAGCAACGAGACtATTGAGGGACTTAAGGCTCTCGCGCAGAAGCACAACTTTCTCATCTTCGAGGACCGCAAGTTCATTGACATCGGCAACACGGTCCAGAA
GCAATACCACGGCGGTACCCTCCGTATCTCGGAATGGGCCCACATCATCAACTGCAGCATTCTCCCTGGTGAGGGTATCGTCGAGGCTCTCGCTCAGACGGCGTCTGCACC
GGACTTCGCCTACGGCCCCGAACGCGGTCTGTTGATCTTGGCAGAGATGACCTCTAAGGGCTCCTTGGCTACCGGCCAGTACACTACTTCCTCGGTCGATTATGCCCGGAA
ATACAAGAACTTCGTTATGGGATTCGTGTCGACGCGCGCGTTGGGTGAGGTGCAGTCGGAAGTCAGCTCTCCTTCGGATGAGGAGGACTTTGTGGTtTTCACGACTGGTGT
GAACATTAACCTCCA

>pSF523_IC
TGGAGGaAGGGATCCGCCAAGGATGCTCTGGATCCGCATCCGGCCGCTCTTGCGCCCCATCAATCGCCCGACTATAAATCGAACTACTTTCGGCATCTTCTAGACTTCCTAATA
CCGCCTAGTCATAGCAGATTCAAGCTGAGAACACCACAAGTAAATATCACCCATCATGCTTACCCTGACCGTCCCTGAAAACTACGGGTATGTGCCAATTCTACAATTCCTTG
CAGACAATGCCATTCTCCCCATGAAGTCTGATGCTAACTATCCTGCAGCTCTGTCATTGCCGTCGCTCTGGGTGCCATCCCCGTCCTGAGCTTCGTCCATGGCGCCGTCGTGT
CTCGTCTCCGCAAGGAAGCTGATTGCCCCTACCCTCACTGCTATGCGACCGTAGAGCAGTGCAAGACCAACGTAAGCCAACCTCACACAAACAGGATTCCTCGAGCTAACA
TACATTCCGAACCGTGCAGCCCAAGGCCGAGCAGTTCAACTGCGCTCAGCGCGCTCATGCCAACTTCCTTGAGAACTCCAGCCAAACTATGCTCTTCCTCCTGGTAGCTGGA
CTGAAGTACCCCCAGTTGGCGACTGGCCTCGGAAGCATCTGGGTCCTCGGTCGCTCACTGTTCCTTTACGGATATGTGTACTCCGGCAAGCCGCGGGGTCGCGGTCGTTTGT
ACGGCAGCTTCTACTTGCTTGCACAGGGAGCTCTCTGGGGCTTGACGTCTTTTGGAGTTGCGAGGGAGTTGATTTCCTACTTCTAAGTTTGGACTGAATCCGTGGTGTGATT
GAGGTGATTGGCGATGTTTGGCTATACCAGCTATATGTAATAATCTCTACTGTATACTACTATTCAACGCATTTTACTATGCGTGCTGCTAGGGTCGGCAATGACAATGGCAATC
TGACTGACGTGGTCTATTTCTCCATGTGCAGCAGGGAATACGAGCTCCAATGGACCTCGGGAGTGGCACAGTCAATGGCAAGGAAACTGGAGcgcaaaaaaccccgcccctgacagggcg
gggtttttcgcGATCGGAGTCGATAAGCTTCGGAGAATATGGAGCTTCATCGAATCACCGGCAGTAAGCGAAGGAGAATGTGAAGCCAGGGGTGTATAGCCGTCGGCGAAATAGC
ATGCCATTAACCTAGGTACAGAAGTCCAATTGCTTCCGATCTGGTAAAAGATTCACGAGATAGTACCTTCTCCGAAGTAGGTAGAGCGAGTACCCGGCGCGTAAGCTCCCTA
ATTGGCCCATCCGGCATCTGTAGGGCGTCCAAATATCGTGCCTCTCCTGCTTTGCCCGGTGTATGAAACCGGAAAGGCCGCTCAGGAGCTGGCCAGCGGCGCAGACCGGGA
ACACAAGCTGGCAGTCGACCCATCCGGTGCTCTGCACTCGACCTGCTGAGGTCCCTCAGTCCCTGGTAGGCAGCTTTGCCCCGTCTGTCCGCCCGGTGTGTCGGCGGGGTT
GACAAGGTCGTTGCGTCAGTCCAACATTTGTTGCCATATTTTCCTGCTCTCCCCACCAGCTGCTCTTTTCTTTTCTCTTTCTTTTCCCATCTTCAGTATATTCATCTTCCCATCCA
AGAACCTTTATTTCCCCTAAGTAAGTACTTTGCTACATCCATACTCCATCCTTCCCATCCCTTATTCCTTTGAACCTTTCAGTTCGAGCTTTCCCACTTCATCGCAGCTTGACTA
ACAGCTACCCCGCTTGAGCAGACATCACCGAATTCACCATGTCTAGACTGGACAAGAGCAAAGTCATAAACGGCGCTCTGGAATTACTCAATGGAGTCGGTATCGAAGGCC
TGACGACAAGGAAACTCGCTCAAAAGCTGGGAGTTGAGCAGCCTACCCTGTACTGGCACGTGAAGAACAAGCGGGCCCTGCTCGATGCCCTGCCAATCGAGATGCTGGAC
AGGCATCATACCCACTTCTGCCCCCTGGAAGGCGAGTCATGGCAAGACTTTCTGCGGAACAACGCCAAGTCATTCCGCTGTGCTCTCCTCTCACATCGCGACGGGGCTAAA
GTGCATCTCGGCACCCGCCCAACAGAGAAACAGTACGAAACCCTGGAAAATCAGCTCGCGTTCCTGTGTCAGCAAGGCTTCTCCCTGGAGAACGCACTGTACGCTCTGTCC
GCCGTGGGCCACTTTACACTGGGCTGCGTATTGGAGGAACAGGAGCATCAAGTAGCAAAAGAGGAAAGAGAGACACCTACCACCGATTCTATGCCCCCACTTCTGAGACA
AGCAATTGAGCTGTTCGACCGGCAGGGAGCCGAACCTGCCTTCCTTTTCGGCCTGGAACTAATCATATGTGGCCTGGAGAAACAGCTAAAGTGCGAAAGCGGCGGGCCGGC
CGACGCCCTTGACGATTTTGACTTAGACATGCTCCCAGCCGATGCCCTTGACGACTTTGACCTTGATATGCTGCCTGCTGACGCTCTTGACGATTTTGACCTTGACATGCTCC
CCGGGTAACTAAGTAAGGATCCACTAGTACAGCAGAAGAATCTCTCTCCGCTGTTGCTTCAGTGTCTGCCATGCATTAACTTCATCCTACTGTCCTACCCGCAGTACCCATTC

ACATTTGCGGCAGATACCAGGTTGTTTTCTATTCCCTCGGTTCTTCAGTTCTTCAGATATTACATTACTCGAGAAATTGGCGGCGATGCAGGAGTTTGGTTGGTTTTAATTGTTT
CAGTCTCCTTGACCTGATTTGTATGACATGCAATGTTTCGACGGATGACTATCTCGGTGAATATACGTCATGAATCATGCGGCCGCGCGTATCACGAGGCCCTTTCGACTTCAC
TCGAGTTTACCACTCCCTATCAGTGATAGAGAAAAGTGAAAGTCGAGTTTACCACTCCCTATCAGTGATAGAGAAAAGTGAAAGTCGAGTTTACCACTCCCTATCAGTGATA
GAGAAAAGTGAAAGTCGAGTTTACCACTCCCTATCAGTGATAGAGAAAAGTGAAAGTCGAGTTTACCACTCCCTATCAGTGATAGAGAAAAGTGAAAGTCGAGTTTACCAC
TCCCTATCAGTGATAGAGAAAAGTGAAAGTCGAGTTTACCACTCCCTATCAGTGATAGAGAAAAGTGAAAGTCGAGCTCCCCATCTTCAGTATATTCATCTTCCCATCCAAGA
ACCTTTATTTCCCCTAAGTAAGTACTTTGCTACATCCATACTCCATCCTTCCCATCCCTTATTCCTTTGAACCTTTCAGTTCGAGCTTTCCCACTTCATCGCAGCTTGACTAACA
GCTACCCCGCTTGAGCAGACATCACCGTTTAAACACCATGACATCCCCTGTAATTGGTACTCCTTGGAAGAAGTTGAACGCCCCAGTCTCAGAAGAAGCATTGGAAGGTGTT
GATAAGTATTGGAGAGTTGCAAATTACTTGTCAATTGGTCAAATTTACTTGAGATCAAATCCATTGATGAAAGAACCATTCACTAGAGAAGATGTTAAACATAGATTGGTTGG
TCATTGGGGTACAACTCCAGGTTTAAATTTCTTGATTGGTCATATTAATAGATTCATTGCTGATCATGGTCAAAATACAGTTATTATTATGGGTCCAGGTCATGGTGGTCCAGCT
GGTACTTCACAATCTTACTTGGATGGTACTTATACAGAAACTTTTCCAAAAATTACTAAAGATGAAGCAGGTTTGCAAAAATTTTTCAGACAATTTTCTTATCCAGGTGGTATT
CCATCTCATTTTGCACCAGAAACACCAGGTTCAATTCATGAAGGTGGTGAATTGGGTTACGCTTTGTCTCATGCTTATGGTGCAATTATGGATAATCCATCTTTGTTTGTTCCA
GCTATTGTTGGTGACGGTGAAGCTGAAACTGGTCCATTGGCTACTGGTTGGCAATCAAATAAGTTGGTTAATCCAAGAACTGATGGTATTGTTTTACCAATTTTGCATTTGAAT
GGTTACAAAATTGCTAATCCAACAATTTTATCAAGAATTTCTGATGAAGAATTGCATGAATTTTTCCATGGTATGGGTTACGAACCATACGAATTTGTTGCAGGTTTTGATGATG
AAGATCATATGTCAATTCATAGAAGATTTGCTGAATTATGGGAAACAATTTGGGATGAAATTTGTGATATTAAAGCAACAGCTCAAACTGATAATGTTCATAGACCATTTTATCC
AATGTTGATTTTTAGAACACCAAAAGGTTGGACTTGTCCAAAATATATTGATGGTAAAAAGACTGAAGGTTCATGGAGATCACATCAAGTTCCATTAGCATCTGCTAGAGATA
CTGAAGCACATTTTGAAGTTTTGAAAAATTGGTTGGAATCTTATAAACCAGAAGAATTGTTTGATGCAAATGGTGCAGTTAAAGATGATGTTTTAGCTTTTATGCCAAAAGGT
GAATTGAGAATTGGTGCTAATCCAAATGCTAATGGTGGTGTTATTAGAAATGATTTGAAATTACCAAATTTGGAAGATTACGAAGTTAAAGAAGTTGCAGAATACGGTCATGG
TTGGGGTCAATTGGAAGCTACTAGAACTTTAGGTGCTTATACTAGAGATATTATTAAAAATAATCCAAGAGATTTTAGAATTTTTGGTCCAGATGAAACTGCTTCTAATAGATTG
CAAGCATCTTATGAAGTTACTAATAAGCAATGGGATGCTGGTTATATTTCAGATGAAGTTGATGAACATATGCATGTTTCAGGTCAAGTTGTTGAACAATTGTCAGAACATCAA
ATGGAAGGTTTCTTGGAAGCATACTTGTTAACAGGTAGACATGGTATTTGGTCATCTTATGAATCTTTTGTTCATGTTATTGATTCAATGTTAAATCAACATGCAAAATGGTTGG
AAGCTACAGTTAGAGAAATTCCATGGAGAAAACCAATTGCATCTATGAATTTGTTAGTTTCTTCACATGTTTGGAGACAAGATCATAATGGTTTTTCACATCAAGATCCAGGT
GTTACTTCTGTTTTGTTGAATAAGTGTTTTCATAATGATCATGTTATTGGTATTTACTTTGCAACTGATGCTAATATGTTGTTAGCTATTGCTGAAAAATGTTACAAATCAACTAA
TAAGATTAATGCAATTATTGCTGGTAAACAACCAGCAGCTACTTGGTTAACATTGGATGAAGCTAGAGCAGAATTAGAAAAAGGTGCAGCTGCTTGGGATTGGGCATCTACT
GCTAAAAATAATGATGAAGCTGAAGTTGTTTTAGCAGCAGCTGGTGACGTTCCAACTCAAGAAATTATGGCAGCTTCAGATAAATTGAAAGAATTGGGTATTAAATTCAAAG
TTGTTAATGTTGCAGATTTGTTATCATTGCAATCTGCTAAAGAAAATGATGAAGCATTAACTGATGAAGAATTTGCTGATATTTTTACAGCTGATAAACCAGTTTTATTTGCTTA
CCATTCTTATGCTCATGATGTTAGAGGTTTGATTTACGATAGACCAAATCATGATAATTTTAATGTTCATGGTTATGAAGAAGAAGGTTCAACTACAACTCCATACGATATGGTT
AGAGTTAATAGAATTGATAGATACGAATTGACTGCTGAAGCATTGAGAATGATTGATGCAGATAAATACGCAGATAAAATTGATGAATTGGAAAAATTCAGAGATGAAGCATT
TCAATTTGCAGTTGATAATGGTTATGATCATCCAGATTATACAGATTGGGTTTACTCAGGTGTAAATACCGACAAAAAGGGTGCTGTAACCGCTACCGCTGCTACCGCTGGTGA
CAACGAATAAGCTTGATCCACTTAACGTTACTGAAATCATCAAACAGCTTGACGAATCTGGATATAAGATCGTTGGTGTCGATGTCAGCTCCGGAGTTGAGACAAATGGTGTT
CAGGATCTCGATAAGATACGTTCATTTGTCCAAGCAGCAAAGAGTGCCTTCTAGTGATTTAATAGCTCCATGTCAACAAGAATAAAACGCGTTTCGGGTTTACCTCTTCCAGA
TACAGCTCATCTGCAATGCATTAATGCATTGGACCTCGCAACCCTAGTACGCCCTTCAGGCTCCGGCGAAGCAGAAGAATAGCTTAGCAGAGTCTATTTTCATTTTCGGGAGA
CGAGATCAAGCAGATCAACGGTCGTCAAGAGTCCTACGAGACTGAGGAATCCGCTCTTGGCTCCACGCGACTATATATTTGTCTCTAATTGTACTTTGACATGCTCCTCTTCTT
TACTCTGATAGCTTGACTATGAAAATTCCGTCACCAGCCCCTGGGTTCGCAAAGATAATTGCACTGTTTCTTCCTTGAACTCTCAAGCCTACAGGACACACATTCATCGTAGG
TATAAACCTCGAAAATCATTCCTACTAAGATGGGTATACAATAGTAACCATGCATGGTTGCCTAGTGAATGCTCCGTAACACCCAATACGCCGGCCGAAACTTTTTTACAACTC
TCCTATGAGTCGTTTACCCAGAATGCACAGGTACACTTGTTTAGAGGTAATCCTTCTTTCTAGAAGTCCTCGTGTACTGTGTAAGCGCCCACTCCACATCTCCACTCGCTCCG
GGGAGACGGGCACAAGGTTCATGAACCGAAGAGGATGGGAACCCCGACCATGGCCTGCGGCCGCAGCTATCAGTCCTCTCAGCTGACGCCCCTCACGAACCCTCGCACTC
TCCCGAAATGCTCTTCGGTGTCCATGGCGCATCATCTCAGCAGCATCCCATGTCAAACCATGGCTTTGGGCCCACGGACTCTGTGGCCCTGCCGCAACATCACCATCACCATC
GACTCCCGCCCCATGCAGCGCTGCGCGCCCCAGGGCGTCATGGAGTGAATGTGGAATCTCCTCCTTTGCCCTCGGGCCCGCCGAGTGTGGTGGGCCAGCCTGGAATGCCTG
ATCCAGCCCCCAGGCCTCGAGGACCAAAACTGAAGTTTACTCCCGAAGAGGACGCTCTACTGGTGGAGTTGAAGGAAAACAAGAACTTGACGTGGAAGCAAATTGCAGA
CTTCTTCCCGGGCCGAACGAGCGGTACCTTGCAAGTCCGATACTGCACCAAGCTGAAGGCTAAGGATGTAGCTTGGAGTGACGAAATGGTTCGATTTGCTCCTGATGTATTT
CTACGCCTGTCTCACACATGCTAATGAAGGGAATAGGTACAAAGGCTGCAGCGGGCAATGCACGAGTACGAGAACGATCGGTGGCGCATCATTGCAGGGAAGGTTGGAAAT
GGCTTCACCCCAGCTGCTTGCCGCGAGAAAGCCATGCAGCTCCATGAGTAAAAGCGTTGGGGAATTTTCATATTTATATCTACTGTCGCCAGATTCGGCCCTGCTTGGACCCT
CTGATCTCCTTACTCTCCATATTGGTTCAAATGTCGGGTCACCGATAGGGCTGGTGGTGCAGGCTTGTTGTAGGCACGGGAGGATGATCAGCATAACTCTGAGTCACTATAGG
GACGGGTTGATGTAAGGTATTAAGTGATGTATGATAATTCATTTTAGCCCGGGGGAACATATGGCGCCGGCATTTGTTCGTTCGCAATGAACCGACACTAGCGTCCGCTCTCG
CAGTTTAGCACCGGCTGATCCCGGGCTGAACGCGGCCATTGCTCGGCCGGGGCATGTGTTCCTTATCTACGGCAGACCGCAGATGACCACTGGAGCAGATTATAGACCCTAA
GCCCTAAGCCGGACACCCAATCGAGTAGGTCTGCGGACCAGGTCACTGCGGGCAGCCGGAGAAGCTCCGCAACCAATCAATCCCCGGCGCTGACTAAGGGCAGGCGACCA
CGGGCCGAAGCGGCTTCAAACTCACCTCAACCTCCAAACTCCCTCATCTCCAAACGTCCTTGCCTTGTCTGCCGTCATTGCAACCCACCCACCAGGACACATGAGCAGCAA
GTCCCAGCTCACCTACACTGCCCGTGCCAGCAAGCATCCCAATGCTCTGGCGAAGAGGCTGTTCGAGATTGCCGAGGCCAAGAAaACCAATGTGACTGTCTCGGCTGACGT
TACCACCACTAAGGAGCTACTAGATCTTGCTGACCGTCTCGGTCCCTACATTGCCGTGATCAAAACCCACATCGATATCCTCTCTGATTTCAGCAACGAGACtATTGAGGGACT
TAAGGCTCTCGCGCAGAAGCACAACTTTCTCATCTTCGAGGACCGCAAGTTCATTGACATCGGCAACACGGTCCAGAAGCAATACCACGGCGGTACCCTCCGTATCTCGGA
ATGGGCCCACATCATCAACTGCAGCATTCTCCCTGGTGAGGGTATCGTCGAGGCTCTCGCTCAGACGGCGTCTGCACCGGACTTCGCCTACGGCCCCGAACGCGGTCTGTTG
ATCTTGGCAGAGATGACCTCTAAGGGCTCCTTGGCTACCGGCCAGTACACTACTTCCTCGGTCGATTATGCCCGGAAATACAAGAACTTCGTTATGGGATTCGTGTCGACGCG
CGCGTTGGGTGAGGTGCAGTCGGAAGTCAGCTCTCCTTCGGATGAGGAGGACTTTGTGGTtTTCACGACTGGTGTGAACATTAACCTCCA

>pSF537_IC
TGGAGGaAGGGATCCGCCAAGGATGCTCTGGATCCGCATCCGGCCGCTCTTGCGCCCCATCAATCGCCCGACTATAAATCGAACTACTTTCGGCATCTTCTAGACTTCCTA
ATACCGCCTAGTCATAGCAGATTCAAGCTGAGAACACCACAAGTAAATATCACCCATCATGCTTACCCTGACCGTCCCTGAAAACTACGGGTATGTGCCAATTCTACAATT
CCTTGCAGACAATGCCATTCTCCCCATGAAGTCTGATGCTAACTATCCTGCAGCTCTGTCATTGCCGTCGCTCTGGGTGCCATCCCCGTCCTGAGCTTCGTCCATGGCGCCG
TCGTGTCTCGTCTCCGCAAGGAAGCTGATTGCCCCTACCCTCACTGCTATGCGACCGTAGAGCAGTGCAAGACCAACGTAAGCCAACCTCACACAAACAGGATTCCTCGA
GCTAACATACATTCCGAACCGTGCAGCCCAAGGCCGAGCAGTTCAACTGCGCTCAGCGCGCTCATGCCAACTTCCTTGAGAACTCCAGCCAAACTATGCTCTTCCTCCTGG
TAGCTGGACTGAAGTACCCCCAGTTGGCGACTGGCCTCGGAAGCATCTGGGTCCTCGGTCGCTCACTGTTCCTTTACGGATATGTGTACTCCGGCAAGCCGCGGGGTCGCG
GTCGTTTGTACGGCAGCTTCTACTTGCTTGCACAGGGAGCTCTCTGGGGCTTGACGTCTTTTGGAGTTGCGAGGGAGTTGATTTCCTACTTCTAAGTTTGGACTGAATCCGT
GGTGTGATTGAGGTGATTGGCGATGTTTGGCTATACCAGCTATATGTAATAATCTCTACTGTATACTACTATTCAACGCATTTTACTATGCGTGCTGCTAGGGTCGGCAATG
ACAATGGCAATCTGACTGACGTGGTCTATTTCTCCATGTGCAGCAGGGAATACGAGCTCCAATGGACCTCGGGAGTGGCACAGTCAATGGCAAGGAAACTGGAGcgcaaaaaa
ccccgcccctgacagggcggggtttttcgcGATCGGAGTCGATAAGCTTCGGAGAATATGGAGCTTCATCGAATCACCGGCAGTAAGCGAAGGAGAATGTGAAGCCAGGGGTGTATAGCC
GTCGGCGAAATAGCATGCCATTAACCTAGGTACAGAAGTCCAATTGCTTCCGATCTGGTAAAAGATTCACGAGATAGTACCTTCTCCGAAGTAGGTAGAGCGAGTACCCG
GCGCGTAAGCTCCCTAATTGGCCCATCCGGCATCTGTAGGGCGTCCAAATATCGTGCCTCTCCTGCTTTGCCCGGTGTATGAAACCGGAAAGGCCGCTCAGGAGCTGGCC
AGCGGCGCAGACCGGGAACACAAGCTGGCAGTCGACCCATCCGGTGCTCTGCACTCGACCTGCTGAGGTCCCTCAGTCCCTGGTAGGCAGCTTTGCCCCGTCTGTCCGCC
CGGTGTGTCGGCGGGGTTGACAAGGTCGTTGCGTCAGTCCAACATTTGTTGCCATATTTTCCTGCTCTCCCCACCAGCTGCTCTTTTCTTTTCTCTTTCTTTTCCCATCTTCA
GTATATTCATCTTCCCATCCAAGAACCTTTATTTCCCCTAAGTAAGTACTTTGCTACATCCATACTCCATCCTTCCCATCCCTTATTCCTTTGAACCTTTCAGTTCGAGCTTTC
CCACTTCATCGCAGCTTGACTAACAGCTACCCCGCTTGAGCAGACATCACCGAATTCACCATGTCTAGACTGGACAAGAGCAAAGTCATAAACGGCGCTCTGGAATTACT
CAATGGAGTCGGTATCGAAGGCCTGACGACAAGGAAACTCGCTCAAAAGCTGGGAGTTGAGCAGCCTACCCTGTACTGGCACGTGAAGAACAAGCGGGCCCTGCTCGAT
GCCCTGCCAATCGAGATGCTGGACAGGCATCATACCCACTTCTGCCCCCTGGAAGGCGAGTCATGGCAAGACTTTCTGCGGAACAACGCCAAGTCATTCCGCTGTGCTCT
CCTCTCACATCGCGACGGGGCTAAAGTGCATCTCGGCACCCGCCCAACAGAGAAACAGTACGAAACCCTGGAAAATCAGCTCGCGTTCCTGTGTCAGCAAGGCTTCTCCC
TGGAGAACGCACTGTACGCTCTGTCCGCCGTGGGCCACTTTACACTGGGCTGCGTATTGGAGGAACAGGAGCATCAAGTAGCAAAAGAGGAAAGAGAGACACCTACCAC
CGATTCTATGCCCCCACTTCTGAGACAAGCAATTGAGCTGTTCGACCGGCAGGGAGCCGAACCTGCCTTCCTTTTCGGCCTGGAACTAATCATATGTGGCCTGGAGAAACA
GCTAAAGTGCGAAAGCGGCGGGCCGGCCGACGCCCTTGACGATTTTGACTTAGACATGCTCCCAGCCGATGCCCTTGACGACTTTGACCTTGATATGCTGCCTGCTGACGC
TCTTGACGATTTTGACCTTGACATGCTCCCCGGGTAACTAAGTAAGGATCCACTAGTACAGCAGAAGAATCTCTCTCCGCTGTTGCTTCAGTGTCTGCCATGCATTAACTTC
ATCCTACTGTCCTACCCGCAGTACCCATTCACATTTGCGGCAGATACCAGGTTGTTTTCTATTCCCTCGGTTCTTCAGTTCTTCAGATATTACATTACTCGAGAAATTGGCG
GCGATGCAGGAGTTTGGTTGGTTTTAATTGTTTCAGTCTCCTTGACCTGATTTGTATGACATGCAATGTTTCGACGGATGACTATCTCGGTGAATATACGTCATGAATCATG
CGGCCGCGCGTATCACGAGGCCCTTTCGACTTCACTCGAGTTTACCACTCCCTATCAGTGATAGAGAAAAGTGAAAGTCGAGTTTACCACTCCCTATCAGTGATAGAGAA
AAGTGAAAGTCGAGTTTACCACTCCCTATCAGTGATAGAGAAAAGTGAAAGTCGAGTTTACCACTCCCTATCAGTGATAGAGAAAAGTGAAAGTCGAGTTTACCACTCCC
TATCAGTGATAGAGAAAAGTGAAAGTCGAGTTTACCACTCCCTATCAGTGATAGAGAAAAGTGAAAGTCGAGTTTACCACTCCCTATCAGTGATAGAGAAAAGTGAAAG
TCGAGCTCCCCATCTTCAGTATATTCATCTTCCCATCCAAGAACCTTTATTTCCCCTAAGTAAGTACTTTGCTACATCCATACTCCATCCTTCCCATCCCTTATTCCTTTGAA
CCTTTCAGTTCGAGCTTTCCCACTTCATCGCAGCTTGACTAACAGCTACCCCGCTTGAGCAGACATCACCGTTTAAACACCATGACATCCCCTGTAATTGGTACTCCTTGGA
AGAAGTTGAACGCCCCAGTCTCAGAAGAAGCATTGGAAGGTGTTGATAAGTATTGGAGAGTTGCAAATTACTTGTCAATTGGTCAAATTTACTTGAGATCAAATCCATTG
ATGAAAGAACCATTCACTAGAGAAGATGTTAAACATAGATTGGTTGGTCATTGGGGTACAACTCCAGGTTTAAATTTCTTGATTGGTCATATTAATAGATTCATTGCTGAT
CATGGTCAAAATACAGTTATTATTATGGGTCCAGGTCATGGTGGTCCAGCTGGTACTTCACAATCTTACTTGGATGGTACTTATACAGAAACTTTTCCAAAAATTACTAAA
GATGAAGCAGGTTTGCAAAAATTTTTCAGACAATTTTCTTATCCAGGTGGTATTCCATCTCATTTTGCACCAGAAACACCAGGTTCAATTCATGAAGGTGGTGAATTGGGT
TACGCTTTGTCTCATGCTTATGGTGCAATTATGGATAATCCATCTTTGTTTGTTCCAGCTATTGTTGGTGACGGTGAAGCTGAAACTGGTCCATTGGCTACTGGTTGGCAAT
CAAATAAGTTGGTTAATCCAAGAACTGATGGTATTGTTTTACCAATTTTGCATTTGAATGGTTACAAAATTGCTAATCCAACAATTTTATCAAGAATTTCTGATGAAGAATT
GCATGAATTTTTCCATGGTATGGGTTACGAACCATACGAATTTGTTGCAGGTTTTGATGATGAAGATCATATGTCAATTCATAGAAGATTTGCTGAATTATGGGAAACAAT
TTGGGATGAAATTTGTGATATTAAAGCAACAGCTCAAACTGATAATGTTCATAGACCATTTTATCCAATGTTGATTTTTAGAACACCAAAAGGTTGGACTTGTCCAAAATA
TATTGATGGTAAAAAGACTGAAGGTTCATGGAGATCACATCAAGTTCCATTAGCATCTGCTAGAGATACTGAAGCACATTTTGAAGTTTTGAAAAATTGGTTGGAATCTTA
TAAACCAGAAGAATTGTTTGATGCAAATGGTGCAGTTAAAGATGATGTTTTAGCTTTTATGCCAAAAGGTGAATTGAGAATTGGTGCTAATCCAAATGCTAATGGTGGTGT
TATTAGAAATGATTTGAAATTACCAAATTTGGAAGATTACGAAGTTAAAGAAGTTGCAGAATACGGTCATGGTTGGGGTCAATTGGAAGCTACTAGAACTTTAGGTGCTT
ATACTAGAGATATTATTAAAAATAATCCAAGAGATTTTAGAATTTTTGGTCCAGATGAAACTGCTTCTAATAGATTGCAAGCATCTTATGAAGTTACTAATAAGCAATGGG
ATGCTGGTTATATTTCAGATGAAGTTGATGAACATATGCATGTTTCAGGTCAAGTTGTTGAACAATTGTCAGAACATCAAATGGAAGGTTTCTTGGAAGCATACTTGTTAA
CAGGTAGACATGGTATTTGGTCATCTTATGAATCTTTTGTTCATGTTATTGATTCAATGTTAAATCAACATGCAAAATGGTTGGAAGCTACAGTTAGAGAAATTCCATGGA
GAAAACCAATTGCATCTATGAATTTGTTAGTTTCTTCACATGTTTGGAGACAAGATCATAATGGTTTTTCACATCAAGATCCAGGTGTTACTTCTGTTTTGTTGAATAAGTG
TTTTCATAATGATCATGTTATTGGTATTTACTTTGCAACTGATGCTAATATGTTGTTAGCTATTGCTGAAAAATGTTACAAATCAACTAATAAGATTAATGCAATTATTGCT
GGTAAACAACCAGCAGCTACTTGGTTAACATTGGATGAAGCTAGAGCAGAATTAGAAAAAGGTGCAGCTGCTTGGGATTGGGCATCTACTGCTAAAAATAATGATGAAG
CTGAAGTTGTTTTAGCAGCAGCTGGTGACGTTCCAACTCAAGAAATTATGGCAGCTTCAGATAAATTGAAAGAATTGGGTATTAAATTCAAAGTTGTTAATGTTGCAGATT

TGTTATCATTGCAATCTGCTAAAGAAAATGATGAAGCATTAACTGATGAAGAATTTGCTGATATTTTTACAGCTGATAAACCAGTTTTATTTGCTTACCATTCTTATGCTCA
TGATGTTAGAGGTTTGATTTACGATAGACCAAATCATGATAATTTTAATGTTCATGGTTATGAAGAAGAAGGTTCAACTACAACTCCATACGATATGGTTAGAGTTAATAG
AATTGATAGATACGAATTGACTGCTGAAGCATTGAGAATGATTGATGCAGATAAATACGCAGATAAAATTGATGAATTGGAAAAATTCAGAGATGAAGCATTTCAATTTG
CAGTTGATAATGGTTATGATCATCCAGATTATACAGATTGGGTTTACTCAGGTGTAAATACCGACAAAAAGGGTGCTGTAACCGCTACCGCTGCTACCGCTGGTGACAAC
GAAcatcatcaccaccaccatTAAGCTTGATCCACTTAACGTTACTGAAATCATCAAACAGCTTGACGAATCTGGATATAAGATCGTTGGTGTCGATGTCAGCTCCGGAGTTGAGAC
AAATGGTGTTCAGGATCTCGATAAGATACGTTCATTTGTCCAAGCAGCAAAGAGTGCCTTCTAGTGATTTAATAGCTCCATGTCAACAAGAATAAAACGCGTTTCGGGTTT
ACCTCTTCCAGATACAGCTCATCTGCAATGCATTAATGCATTGGACCTCGCAACCCTAGTACGCCCTTCAGGCTCCGGCGAAGCAGAAGAATAGCTTAGCAGAGTCTATTT
TCATTTTCGGGAGACGAGATCAAGCAGATCAACGGTCGTCAAGAGTCCTACGAGACTGAGGAATCCGCTCTTGGCTCCACGCGACTATATATTTGTCTCTAATTGTACTTT
GACATGCTCCTCTTCTTTACTCTGATAGCTTGACTATGAAAATTCCGTCACCAGCCCCTGGGTTCGCAAAGATAATTGCACTGTTTCTTCCTTGAACTCTCAAGCCTACAGG
ACACACATTCATCGTAGGTATAAACCTCGAAAATCATTCCTACTAAGATGGGTATACAATAGTAACCATGCATGGTTGCCTAGTGAATGCTCCGTAACACCCAATACGCC
GGCCGAAACTTTTTTACAACTCTCCTATGAGTCGTTTACCCAGAATGCACAGGTACACTTGTTTAGAGGTAATCCTTCTTTCTAGAAGTCCTCGTGTACTGTGTAAGCGCCC
ACTCCACATCTCCACTCGCTCCGGGGAGACGGGCACAAGGTTCATGAACCGAAGAGGATGGGAACCCCGACCATGGCCTGCGGCCGCAGCTATCAGTCCTCTCAGCTGAC
GCCCCTCACGAACCCTCGCACTCTCCCGAAATGCTCTTCGGTGTCCATGGCGCATCATCTCAGCAGCATCCCATGTCAAACCATGGCTTTGGGCCCACGGACTCTGTGGCC
CTGCCGCAACATCACCATCACCATCGACTCCCGCCCCATGCAGCGCTGCGCGCCCCAGGGCGTCATGGAGTGAATGTGGAATCTCCTCCTTTGCCCTCGGGCCCGCCGAGT
GTGGTGGGCCAGCCTGGAATGCCTGATCCAGCCCCCAGGCCTCGAGGACCAAAACTGAAGTTTACTCCCGAAGAGGACGCTCTACTGGTGGAGTTGAAGGAAAACAAGA
ACTTGACGTGGAAGCAAATTGCAGACTTCTTCCCGGGCCGAACGAGCGGTACCTTGCAAGTCCGATACTGCACCAAGCTGAAGGCTAAGGATGTAGCTTGGAGTGACGA
AATGGTTCGATTTGCTCCTGATGTATTTCTACGCCTGTCTCACACATGCTAATGAAGGGAATAGGTACAAAGGCTGCAGCGGGCAATGCACGAGTACGAGAACGATCGGT
GGCGCATCATTGCAGGGAAGGTTGGAAATGGCTTCACCCCAGCTGCTTGCCGCGAGAAAGCCATGCAGCTCCATGAGTAAAAGCGTTGGGGAATTTTCATATTTATATCT
ACTGTCGCCAGATTCGGCCCTGCTTGGACCCTCTGATCTCCTTACTCTCCATATTGGTTCAAATGTCGGGTCACCGATAGGGCTGGTGGTGCAGGCTTGTTGTAGGCACGG
GAGGATGATCAGCATAACTCTGAGTCACTATAGGGACGGGTTGATGTAAGGTATTAAGTGATGTATGATAATTCATTTTAGCCCGGGGGAACATATGGCGCCGGCATTTG
TTCGTTCGCAATGAACCGACACTAGCGTCCGCTCTCGCAGTTTAGCACCGGCTGATCCCGGGCTGAACGCGGCCATTGCTCGGCCGGGGCATGTGTTCCTTATCTACGGCA
GACCGCAGATGACCACTGGAGCAGATTATAGACCCTAAGCCCTAAGCCGGACACCCAATCGAGTAGGTCTGCGGACCAGGTCACTGCGGGCAGCCGGAGAAGCTCCGCA
ACCAATCAATCCCCGGCGCTGACTAAGGGCAGGCGACCACGGGCCGAAGCGGCTTCAAACTCACCTCAACCTCCAAACTCCCTCATCTCCAAACGTCCTTGCCTTGTCTGC
CGTCATTGCAACCCACCCACCAGGACACATGAGCAGCAAGTCCCAGCTCACCTACACTGCCCGTGCCAGCAAGCATCCCAATGCTCTGGCGAAGAGGCTGTTCGAGATTG
CCGAGGCCAAGAAaACCAATGTGACTGTCTCGGCTGACGTTACCACCACTAAGGAGCTACTAGATCTTGCTGACCGTCTCGGTCCCTACATTGCCGTGATCAAAACCCACA
TCGATATCCTCTCTGATTTCAGCAACGAGACtATTGAGGGACTTAAGGCTCTCGCGCAGAAGCACAACTTTCTCATCTTCGAGGACCGCAAGTTCATTGACATCGGCAACA
CGGTCCAGAAGCAATACCACGGCGGTACCCTCCGTATCTCGGAATGGGCCCACATCATCAACTGCAGCATTCTCCCTGGTGAGGGTATCGTCGAGGCTCTCGCTCAGACG
GCGTCTGCACCGGACTTCGCCTACGGCCCCGAACGCGGTCTGTTGATCTTGGCAGAGATGACCTCTAAGGGCTCCTTGGCTACCGGCCAGTACACTACTTCCTCGGTCGAT
TATGCCCGGAAATACAAGAACTTCGTTATGGGATTCGTGTCGACGCGCGCGTTGGGTGAGGTGCAGTCGGAAGTCAGCTCTCCTTCGGATGAGGAGGACTTTGTGGTtTTC
ACGACTGGTGTGAACATTAACCTCCA

>pSF545_IC
TGGAGGaAGGGATCCGCCAAGGATGCTCTGGATCCGCATCCGGCCGCTCTTGCGCCCCATCAATCGCCCGACTATAAATCGAACTACTTTCGGCATCTTCTAGACTTCCTA
ATACCGCCTAGTCATAGCAGATTCAAGCTGAGAACACCACAAGTAAATATCACCCATCATGCTTACCCTGACCGTCCCTGAAAACTACGGGTATGTGCCAATTCTACAATT
CCTTGCAGACAATGCCATTCTCCCCATGAAGTCTGATGCTAACTATCCTGCAGCTCTGTCATTGCCGTCGCTCTGGGTGCCATCCCCGTCCTGAGCTTCGTCCATGGCGCCG
TCGTGTCTCGTCTCCGCAAGGAAGCTGATTGCCCCTACCCTCACTGCTATGCGACCGTAGAGCAGTGCAAGACCAACGTAAGCCAACCTCACACAAACAGGATTCCTCGA
GCTAACATACATTCCGAACCGTGCAGCCCAAGGCCGAGCAGTTCAACTGCGCTCAGCGCGCTCATGCCAACTTCCTTGAGAACTCCAGCCAAACTATGCTCTTCCTCCTGG
TAGCTGGACTGAAGTACCCCCAGTTGGCGACTGGCCTCGGAAGCATCTGGGTCCTCGGTCGCTCACTGTTCCTTTACGGATATGTGTACTCCGGCAAGCCGCGGGGTCGCG
GTCGTTTGTACGGCAGCTTCTACTTGCTTGCACAGGGAGCTCTCTGGGGCTTGACGTCTTTTGGAGTTGCGAGGGAGTTGATTTCCTACTTCTAAGTTTGGACTGAATCCGT
GGTGTGATTGAGGTGATTGGCGATGTTTGGCTATACCAGCTATATGTAATAATCTCTACTGTATACTACTATTCAACGCATTTTACTATGCGTGCTGCTAGGGTCGGCAATG
ACAATGGCAATCTGACTGACGTGGTCTATTTCTCCATGTGCAGCAGGGAATACGAGCTCCAATGGACCTCGGGAGTGGCACAGTCAATGGCAAGGAAACTGGAGcgcaaaaaa
ccccgcccctgacagggcggggtttttcgcGATCGGAGTCGATAAGCTTCGGAGAATATGGAGCTTCATCGAATCACCGGCAGTAAGCGAAGGAGAATGTGAAGCCAGGGGTGTATAGCC
GTCGGCGAAATAGCATGCCATTAACCTAGGTACAGAAGTCCAATTGCTTCCGATCTGGTAAAAGATTCACGAGATAGTACCTTCTCCGAAGTAGGTAGAGCGAGTACCCG
GCGCGTAAGCTCCCTAATTGGCCCATCCGGCATCTGTAGGGCGTCCAAATATCGTGCCTCTCCTGCTTTGCCCGGTGTATGAAACCGGAAAGGCCGCTCAGGAGCTGGCC
AGCGGCGCAGACCGGGAACACAAGCTGGCAGTCGACCCATCCGGTGCTCTGCACTCGACCTGCTGAGGTCCCTCAGTCCCTGGTAGGCAGCTTTGCCCCGTCTGTCCGCC
CGGTGTGTCGGCGGGGTTGACAAGGTCGTTGCGTCAGTCCAACATTTGTTGCCATATTTTCCTGCTCTCCCCACCAGCTGCTCTTTTCTTTTCTCTTTCTTTTCCCATCTTCA
GTATATTCATCTTCCCATCCAAGAACCTTTATTTCCCCTAAGTAAGTACTTTGCTACATCCATACTCCATCCTTCCCATCCCTTATTCCTTTGAACCTTTCAGTTCGAGCTTTC
CCACTTCATCGCAGCTTGACTAACAGCTACCCCGCTTGAGCAGACATCACCGAATTCACCATGTCTAGACTGGACAAGAGCAAAGTCATAAACGGCGCTCTGGAATTACT
CAATGGAGTCGGTATCGAAGGCCTGACGACAAGGAAACTCGCTCAAAAGCTGGGAGTTGAGCAGCCTACCCTGTACTGGCACGTGAAGAACAAGCGGGCCCTGCTCGAT
GCCCTGCCAATCGAGATGCTGGACAGGCATCATACCCACTTCTGCCCCCTGGAAGGCGAGTCATGGCAAGACTTTCTGCGGAACAACGCCAAGTCATTCCGCTGTGCTCT
CCTCTCACATCGCGACGGGGCTAAAGTGCATCTCGGCACCCGCCCAACAGAGAAACAGTACGAAACCCTGGAAAATCAGCTCGCGTTCCTGTGTCAGCAAGGCTTCTCCC
TGGAGAACGCACTGTACGCTCTGTCCGCCGTGGGCCACTTTACACTGGGCTGCGTATTGGAGGAACAGGAGCATCAAGTAGCAAAAGAGGAAAGAGAGACACCTACCAC
CGATTCTATGCCCCCACTTCTGAGACAAGCAATTGAGCTGTTCGACCGGCAGGGAGCCGAACCTGCCTTCCTTTTCGGCCTGGAACTAATCATATGTGGCCTGGAGAAACA
GCTAAAGTGCGAAAGCGGCGGGCCGGCCGACGCCCTTGACGATTTTGACTTAGACATGCTCCCAGCCGATGCCCTTGACGACTTTGACCTTGATATGCTGCCTGCTGACGC
TCTTGACGATTTTGACCTTGACATGCTCCCCGGGTAACTAAGTAAGGATCCACTAGTACAGCAGAAGAATCTCTCTCCGCTGTTGCTTCAGTGTCTGCCATGCATTAACTTC
ATCCTACTGTCCTACCCGCAGTACCCATTCACATTTGCGGCAGATACCAGGTTGTTTTCTATTCCCTCGGTTCTTCAGTTCTTCAGATATTACATTACTCGAGAAATTGGCG
GCGATGCAGGAGTTTGGTTGGTTTTAATTGTTTCAGTCTCCTTGACCTGATTTGTATGACATGCAATGTTTCGACGGATGACTATCTCGGTGAATATACGTCATGAATCATG
CGGCCGCGCGTATCACGAGGCCCTTTCGACTTCACTCGAGTTTACCACTCCCTATCAGTGATAGAGAAAAGTGAAAGTCGAGTTTACCACTCCCTATCAGTGATAGAGAA
AAGTGAAAGTCGAGTTTACCACTCCCTATCAGTGATAGAGAAAAGTGAAAGTCGAGTTTACCACTCCCTATCAGTGATAGAGAAAAGTGAAAGTCGAGTTTACCACTCCC
TATCAGTGATAGAGAAAAGTGAAAGTCGAGTTTACCACTCCCTATCAGTGATAGAGAAAAGTGAAAGTCGAGTTTACCACTCCCTATCAGTGATAGAGAAAAGTGAAAG
TCGAGCTCCCCATCTTCAGTATATTCATCTTCCCATCCAAGAACCTTTATTTCCCCTAAGTAAGTACTTTGCTACATCCATACTCCATCCTTCCCATCCCTTATTCCTTTGAA
CCTTTCAGTTCGAGCTTTCCCACTTCATCGCAGCTTGACTAACAGCTACCCCGCTTGAGCAGACATCACCGTTTAAACACCATGCCTCCCAAAGCCGCTGAGAAGAAGCCC
AGCACCGGTGGCAAGGCCCCGGCAGGCGGCAAGGCTCCCGCTGAGAAGAAGGAAGCCGGCAAGAAaACTGCTGCCGCTGCCTCCGGCGACAAGAAGAAGCGTGGAAAG
ACCAGGAAGGAGACtTACTCGTCTTACATCTACAAGGTCTTGAAGCAGGTCCACCCTGATACCGGTATCTCGACTCGTGCTATGTCCATCCTGAACTCCTTTGTCAATGATA
TCTTCGAGCGTGTCGCGACCGAGGCCTCGAAACTTGCCGCTTACAACAAGAAGTCCACCATCTCTTCACGGGAGATCCAGACCTCTGTGAGGCTCATCCTTCCCGGTGAAT
TGGCGAAGCACGCGGTGTCGGAAGGCACCAAGGCTGTCACGAAGTACTCGTCGTCTGCCAAAATGGCCTCCTCCGAGGACGTCATCAAGGAGTTCATGCGCTTCAAGGTG
CGCATGGAGGGCTCCGTGAACGGCCACGAGTTCGAGATCGAGGGCGAGGGCGAGGGCCGCCCCTACGAGGGCACCCAGACCGCCAAGCTGAAGGTGACCAAGGGCGGC
CCCCTGCCCTTCGCCTGGGACATCCTGTCCCCCCAGTTCCAGTACGGCTCCAAGGTGTACGTGAAGCACCCCGCCGACATCCCCGACTACAAGAAGCTGTCCTTCCCCGAG
GGCTTCAAGTGGGAGCGCGTGATGAACTTCGAGGACGGCGGCGTGGTGACCGTGACCCAGGACTCCTCCCTGCAGGACGGCTCCTTCATCTACAAGGTGAAGTTCATCGG
CGTGAACTTCCCCTCCGACGGCCCCGTAATGCAGAAGAAAACTATGGGCTGGGAGGCCTCCACCGAGCGCCTGTACCCCCGCGACGGCGTGCTGAAGGGCGAGATCCAC
AAGGCCCTGAAGCTGAAGGACGGCGGCCACTACCTGGTGGAGTTCAAGTCCATCTACATGGCCAAGAAGCCCGTGCAGCTGCCCGGCTACTACTACGTGGACTCCAAGCT
GGACATCACCTCCCACAACGAGGACTACACCATCGTGGAGCAGTACGAGCGCGCCGAGGGCCGCCACCACCTGTTCCTGTAGGCTTGATCCACTTAACGTTACTGAAATC
ATCAAACAGCTTGACGAATCTGGATATAAGATCGTTGGTGTCGATGTCAGCTCCGGAGTTGAGACAAATGGTGTTCAGGATCTCGATAAGATACGTTCATTTGTCCAAGC
AGCAAAGAGTGCCTTCTAGTGATTTAATAGCTCCATGTCAACAAGAATAAAACGCGTTTCGGGTTTACCTCTTCCAGATACAGCTCATCTGCAATGCATTAATGCATTGGA
CCTCGCAACCCTAGTACGCCCTTCAGGCTCCGGCGAAGCAGAAGAATAGCTTAGCAGAGTCTATTTTCATTTTCGGGAGACGAGATCAAGCAGATCAACGGTCGTCAAGA
GTCCTACGAGACTGAGGAATCCGCTCTTGGCTCCACGCGACTATATATTTGTCTCTAATTGTACTTTGACATGCTCCTCTTCTTTACTCTGATAGCTTGACTATGAAAATTC
CGTCACCAGCCCCTGGGTTCGCAAAGATAATTGCACTGTTTCTTCCTTGAACTCTCAAGCCTACAGGACACACATTCATCGTAGGTATAAACCTCGAAAATCATTCCTACT
AAGATGGGTATACAATAGTAACCATGCATGGTTGCCTAGTGAATGCTCCGTAACACCCAATACGCCGGCCGAAACTTTTTTACAACTCTCCTATGAGTCGTTTACCCAGAA
TGCACAGGTACACTTGTTTAGAGGTAATCCTTCTTTCTAGAAGTCCTCGTGTACTGTGTAAGCGCCCACTCCACATCTCCACTCGCTCCGGGGAGTCGATAAGCTTCGGAG
AATATGGAGCTTCATCGAATCACCGGCAGTAAGCGAAGGAGAATGTGAAGCCAGGGGTGTATAGCCGTCGGCGAAATAGCATGCCATTAACCTAGGTACAGAAGTCCAA
TTGCTTCCGATCTGGTAAAAGATTCACGAGATAGTACCTTCTCCGAAGTAGGTAGAGCGAGTACCCGGCGCGTAAGCTCCCTAATTGGCCCATCCGGCATCTGTAGGGCG
TCCAAATATCGTGCCTCTCCTGCTTTGCCCGGTGTATGAAACCGGAAAGGCCGCTCAGGAGCTGGCCAGCGGCGCAGACCGGGAACACAAGCTGGCAGTCGACCCATCCG
GTGCTCTGCACTCGACCTGCTGAGGTCCCTCAGTCCCTGGTAGGCAGCTTTGCCCCGTCTGTCCGCCCGGTGTGTCGGCGGGGTTGACAAGGTCGTTGCGTCAGTCCAACA
TTTGTTGCCATATTTTCCTGCTCTCCCCACCAGCTGCTCTTTTCTTTTCTCTTTCTTTTCCCATCTTCAGTATATTCATCTTCCCATCCAAGAACCTTTATTTCCCCTAAGTAAG
TACTTTGCTACATCCATACTCCATCCTTCCCATCCCTTATTCCTTTGAACCTTTCAGTTCGAGCTTTCCCACTTCATCGCAGCTTGACTAACAGCTACCCCGCTTGAGCAGAC
ATCACCGAATTCACCATGTCTAGACTGGACAAGAGCAAAGTCATAAACGGCGCTCTGGAATTACTCAATGGAGTCGGTATCGAAGGCCTGACGACAAGGAAACTCGCTC
AAAAGCTGGGAGTTGAGCAGCCTACCCTGTACTGGCACGTGAAGAACAAGCGGGCCCTGCTCGATGCCCTGCCAATCGAGATGCTGGACAGGCATCATACCCACTTCTGC
CCCCTGGAAGGCGAGTCATGGCAAGACTTTCTGCGGAACAACGCCAAGTCATTCCGCTGTGCTCTCCTCTCACATCGCGACGGGGCTAAAGTGCATCTCGGCACCCGCCC
AACAGAGAAACAGTACGAAACCCTGGAAAATCAGCTCGCGTTCCTGTGTCAGCAAGGCTTCTCCCTGGAGAACGCACTGTACGCTCTGTCCGCCGTGGGCCACTTTACAC
TGGGCTGCGTATTGGAGGAACAGGAGCATCAAGTAGCAAAAGAGGAAAGAGAGACACCTACCACCGATTCTATGCCCCCACTTCTGAGACAAGCAATTGAGCTGTTCGA
CCGGCAGGGAGCCGAACCTGCCTTCCTTTTCGGCCTGGAACTAATCATATGTGGCCTGGAGAAACAGCTAAAGTGCGAAAGCGGCGGGCCGGCCGACGCCCTTGACGATT
TTGACTTAGACATGCTCCCAGCCGATGCCCTTGACGACTTTGACCTTGATATGCTGCCTGCTGACGCTCTTGACGATTTTGACCTTGACATGCTCCCCGGGTAACTAAGTAA
GGATCCACTAGTACAGCAGAAGAATCTCTCTCCGCTGTTGCTTCAGTGTCTGCCATGCATTAACTTCATCCTACTGTCCTACCCGCAGTACCCATTCACATTTGCGGCAGAT
ACCAGGTTGTTTTCTATTCCCTCGGTTCTTCAGTTCTTCAGATATTACATTACTCGAGAAATTGGCGGCGATGCAGGAGTTTGGTTGGTTTTAATTGTTTCAGTCTCCTTGAC
CTGATTTGTATGACATGCAATGTTTCGACGGATGACTATCTCGGTGAATATACGTCATGAATCATGCGGCCGCGCGTATCACGAGGCCCTTTCGACTTCACTCGAGTTTAC
CACTCCCTATCAGTGATAGAGAAAAGTGAAAGTCGAGTTTACCACTCCCTATCAGTGATAGAGAAAAGTGAAAGTCGAGTTTACCACTCCCTATCAGTGATAGAGAAAAG
TGAAAGTCGAGTTTACCACTCCCTATCAGTGATAGAGAAAAGTGAAAGTCGAGTTTACCACTCCCTATCAGTGATAGAGAAAAGTGAAAGTCGAGTTTACCACTCCCTAT
CAGTGATAGAGAAAAGTGAAAGTCGAGTTTACCACTCCCTATCAGTGATAGAGAAAAGTGAAAGTCGAGCTCCCCATCTTCAGTATATTCATCTTCCCATCCAAGAACCT
TTATTTCCCCTAAGTAAGTACTTTGCTACATCCATACTCCATCCTTCCCATCCCTTATTCCTTTGAACCTTTCAGTTCGAGCTTTCCCACTTCATCGCAGCTTGACTAACAGC
TACCCCGCTTGAGCAGACATCACCGTTTAAACACCATGGTGAGCAAGGGCGAGGAGCTGTTCACCGGGGTGGTGCCCATCCTGGTCGAGCTGGACGGCGACGTAAACGG
CCACAAGTTCAGCGTGCGCGGCGAGGGCGAGGGCGATGCCACCAACGGCAAGCTGACCCTGAAGTTCATCTGCACCACCGGCAAGCTGCCCGTGCCCTGGCCCACCCTC
GTGACCACCCTGACCTACGGCGTGCAGTGCTTCAGCCGCTACCCCGACCACATGAAGCAGCACGACTTCTTCAAGTCCGCCATGCCCGAAGGCTACGTCCAGGAGCGCAC
CATCACCTTCAAGGACGACGGCACCTACAAGACCCGCGCCGAGGTGAAGTTCGAGGGCGACACCCTGGTGAACCGCATCGAGCTGAAGGGCATCGACTTCAAGGAGGAC

GGCAACATCCTGGGGCACAAGCTGGAGTACAACTTCAACAGCCACAACGTCTATATCACCGCCGACAAGCAGAAGAACGGCATCAAGGCCAACTTCAAGATCCGCCACA
ACGTGGAGGACGGCAGCGTGCAGCTCGCCGACCACTACCAGCAGAACACCCCCATCGGCGACGGCCCCGTGCTGCTGCCCGACAACCACTACCTGAGCACCCAGTCCAA
GCTGAGCAAAGACCCCAACGAGAAGCGCGATCACATGGTCCTGCTGGAGTTCGTGACCGCCGCCGGGATCACTCACGGCATGGACGAGCTGTACAAGgccgccgccATGGAG
AACTACCAGAAGATCGAGAAGATTGGAGAAGGAACtTATGGCGTTGTCTACAAGGCTCGCGAGCTCACCCATCCCAATCGGATTGTCGCCCTCAAGAAGATTCGGCTAGA
GGCGGAGGATGAAGGCGTTCCTAGCACAGCCATCCGTGAAATCTCCCTACTCAAAGAGATGAGCGATCCCAATATTGTGCGACTCCTGAATATTGTGCATGCCGATGGCC
ACAAGCTCTATCTCGTtTTCGAATTCCTCGATCTCGACCTCAAGAAGTATATGGAGGCTCTTCCTGTCAGCGAAGGTGGACGTGGCAAAGCTCTTCCGGAAGGCTCTGCTTT
GAGCAAGAACATGGGCCTTGGCGATGCCATGGTCAAGAAGTTCATGGCTCAGCTAGTGGAGGGTATTCGTTACTGCCACAGCCACCGTATTTTACACCGTGATCTCAAGC
CCCAGAACCTTCTGATCGACCGCGATGGCAATCTTAAGCTGGCGGACTTTGGATTGGCTAGAGCTTTCGGTGTTCCTCTGAGAACCTACACCCATGAGGTTGTCACCCTTT
GGTACCGCTCCCCTGAGATTCTCTTGGGTGGTCGTCAGTACTCGACCGGCGTCGACATGTGGTCCGCTGGTGCCATTTTCGCGGAGATGTGCACTCGCAAGCCTTTGTTCC
CTGGTGATTCGGAGATTGATGAGATCTTCAAGATCTTCCGTCTCCTTGGTACCCCAGATGAAGCTATCTGGCCCGGCGTCACCTCTTTCCCCGACTTCAAACCCACTTTCCC
TAAATGGAAACGTGATGAAACCCGTGCACTCGTCCCTGACCTTGAAGAaGATGGTCTGGACCTCCTCGATGCTCTGCTCGAGTATGACCCTGCACGACGTATCTCCGCCAA
ACAGGCCTGCATGCATCCCTACTTCCGGAACGGCAGTGCCTACTACTCAGGACGTACCCGGAGGAATGGTTTTAACTAGGCTTGATCCACTTAACGTTACTGAAATCATCA
AACAGCTTGACGAATCTGGATATAAGATCGTTGGTGTCGATGTCAGCTCCGGAGTTGAGACAAATGGTGTTCAGGATCTCGATAAGATACGTTCATTTGTCCAAGCAGCA
AAGAGTGCCTTCTAGTGATTTAATAGCTCCATGTCAACAAGAATAAAACGCGTTTCGGGTTTACCTCTTCCAGATACAGCTCATCTGCAATGCATTAATGCATTGGACCTC
GCAACCCTAGTACGCCCTTCAGGCTCCGGCGAAGCAGAAGAATAGCTTAGCAGAGTCTATTTTCATTTTCGGGAGACGAGATCAAGCAGATCAACGGTCGTCAAGAGTCC
TACGAGACTGAGGAATCCGCTCTTGGCTCCACGCGACTATATATTTGTCTCTAATTGTACTTTGACATGCTCCTCTTCTTTACTCTGATAGCTTGACTATGAAAATTCCGTC
ACCAGCCCCTGGGTTCGCAAAGATAATTGCACTGTTTCTTCCTTGAACTCTCAAGCCTACAGGACACACATTCATCGTAGGTATAAACCTCGAAAATCATTCCTACTAAGA
TGGGTATACAATAGTAACCATGCATGGTTGCCTAGTGAATGCTCCGTAACACCCAATACGCCGGCCGAAACTTTTTTACAACTCTCCTATGAGTCGTTTACCCAGAATGCA
CAGGTACACTTGTTTAGAGGTAATCCTTCTTTCTAGAAGTCCTCGTGTACTGTGTAAGCGCCCACTCCACATCTCCACTCGCTAATTGGAGACGGGCACAAGGTTCATGAA
CCGAAGAGGATGGAACCCCGACCATGGCCTGCGGCCGCAGCTATCAGTCCTCTCAGCTGACGCCCCTCACGAACCCTCGCACTCTCCCGAAATGCTCTTCGGTGTCCAT
GGCGCATCATCTCAGCAGCATCCCATGTCAAACCATGGCTTTGGGCCCACGGACTCTGTGGCCCTGCCGCAACATCACCATCACCATCGACTCCCGCCCCATGCAGCGCTG
CGCGCCCCAGGGCGTCATGGAGTGAATGTGGAATCTCCTCCTTTGCCCTCGGGCCCGCCGAGTGTGGTGGGCCAGCCTGGAATGCCTGATCCAGCCCCCAGGCCTCGAGG
ACCAAAACTGAAGTTTACTCCCGAAGAGGACGCTCTACTGGTGGAGTTGAAGGAAAACAAGAACTTGACGTGGAAGCAAATTGCAGACTTCTTCCCGGGCCGAACGAGC
GGTACCTTGCAAGTCCGATACTGCACCAAGCTGAAGGCTAAGGATGTAGCTTGGAGTGACGAAATGGTTCGATTTGCTCCTGATGTATTTCTACGCCTGTCTCACACATGC
TAATGAAGGGAATAGGTACAAAGGCTGCAGCGGGCAATGCACGAGTACGAGAACGATCGGTGGCGCATCATTGCAGGGAAGGTTGGAAATGGCTTCACCCCAGCTGCTT
GCCGCGAGAAAGCCATGCAGCTCCATGAGTAAAAGCGTTGGGGAATTTTCATATTTATATCTACTGTCGCCAGATTCGGCCCTGCTTGGACCCTCTGATCTCCTTACTCTC
CATATTGGTTCAAATGTCGGGTCACCGATAGGGCTGGTGGTGCAGGCTTGTTGTAGGCACGGGAGGATGATCAGCATAACTCTGAGTCACTATAGGGACGGGTTGATGTA
AGGTATTAAGTGATGTATGATAATTCATTTTAGCCCGGGGGAACATATGGCGCCGGCATTTGTTCGTTCGCAATGAACCGACACTAGCGTCCGCTCTCGCAGTTTAGCACC
GGCTGATCCCGGGCTGAACGCGGCCATTGCTCGGCCGGGGCATGTGTTCCTTATCTACGGCAGACCGCAGATGACCACTGGAGCAGATTATAGACCCTAAGCCCTAAGCC
GGACACCCAATCGAGTAGGTCTGCGGACCAGGTCACTGCGGGCAGCCGGAGAAGCTCCGCAACCAATCAATCCCCGGCGCTGACTAAGGGCAGGCGACCACGGGCCGAA
GCGGCTTCAAACTCACCTCAACCTCCAAACTCCCTCATCTCCAAACGTCCTTGCCTTGTCTGCCGTCATTGCAACCCACCCACCAGGACACATGAGCAGCAAGTCCCAGCT
CACCTACACTGCCCGTGCCAGCAAGCATCCCAATGCTCTGGCGAAGAGGCTGTTCGAGATTGCCGAGGCCAAGAAaACCAATGTGACTGTCTCGGCTGACGTTACCACCA
CTAAGGAGCTACTAGATCTTGCTGACCGTCTCGGTCCCTACATTGCCGTGATCAAAACCCACATCGATATCCTCTCTGATTTCAGCAACGAGACtATTGAGGGACTTAAGG
CTCTCGCGCAGAAGCACAACTTTCTCATCTTCGAGGACCGCAAGTTCATTGACATCGGCAACACGGTCCAGAAGCAATACCACGGCGGTACCCTCCGTATCTCGGAATGG
GCCCACATCATCAACTGCAGCATTCTCCCTGGTGAGGGTATCGTCGAGGCTCTCGCTCAGACGGCGTCTGCACCGGACTTCGCCTACGGCCCCGAACGCGGTCTGTTGATC
TTGGCAGAGATGACCTCTAAGGGCTCCTTGGCTACCGGCCAGTACACTACTTCCTCGGTCGATTATGCCCGGAAATACAAGAACTTCGTTATGGGATTCGTGTCGACGCGC
GCGTTGGGTGAGGTGCAGTCGGAAGTCAGCTCTCCTTCGGATGAGGAGGACTTTGTGGTtTTCACGACTGGTGTGAACATTAACCTCCA

Table S2 Primer sequences for PCR verification of targeted genomic integration.

Purpose	Primer name	Sequence (5'-3')
PCR1	pyrG_5' out_fwd	TTTTGGTTAGCACCTACGCTAGTCTATCAG
Set A	cexA_rev	GGAAGTCGGGGTGTGATTTCAG
PCR1	pyrG_5' out_fwd	TTTTGGTTAGCACCTACGCTAGTCTATCAG
Set B	Tet-on_TcrgA_rev	CGCGGCCGCATGATTCATGACGTATAT
PCR1	pyrG_5' out_fwd	TTTTGGTTAGCACCTACGCTAGTCTATCAG
Set C	phkA_rev	ATACGTCAACCGGTGAATAAGCCAC
PCR1	pyrG_5' out_fwd	TTTTGGTTAGCACCTACGCTAGTCTATCAG
Set D	phkB_rev	GTCTCGTGAGTAGTGGGGGTAG
PCR1	pyrG_5' out_fwd	TTTTGGTTAGCACCTACGCTAGTCTATCAG
Set E	Xfspk_rev	GGTGTTTCTGGTGCAAAATGAGATG
PCR2	cexA_fwd	CTAGGCAATGGCTTTGGATGTATGTC
Set A	pyrG_3' out_rev	CATCGGAAGCACAATGAGGCGAGTTT
PCR2	tetO7_fwd	AAAAGTGAAAGTCGAGTTTACCACTCCCTATC
Set B	pyrG_3' out_rev	CATCGGAAGCACAATGAGGCGAGTTT
PCR2	trpC_fwd	CCATGCATGGTTGCCTAGTGAATGC
Set C	pyrG_3' out_rev	CATCGGAAGCACAATGAGGCGAGTTT
PCR3	pyrG_5' out_fwd	TTTTGGTTAGCACCTACGCTAGTCTATCAG
	pyrG_3' out_rev	CATCGGAAGCACAATGAGGCGAGTTT
Sequencing	pyrG_5'_seq_fwd	TCAAGCTCTTATTGTGTCGTTCAAGATTTGTTC GTATG
	pyrG_5'_seq_fwd2	GACTAATTCTCCGGATGTT
	PgpdA_seq_rev	GCTTCACATTCTCCTTCGCTTACTG
