# Supplementary material for: NHEJ and HDR can occur simultaneously during gene integration into the genome of Aspergillus niger
Source: Fungal Biol Biotechnol. 2024 Aug 5;11:10. doi: 10.1186/s40694-024-00180-7 (PMC11301975; doi:10.1186/s40694-024-00180-7)
Supplement: Supplementary file 2 — Supplementary Material 2 [file 40694_2024_180_MOESM2_ESM.pdf]

Supplementary Table S3 Analysis of genome integration events in *A. niger* pyrG^{m1} recipient strains.

Vector	Strain	Casette identifier	Cassette length (bp)	Primerset PCR1	MTR	HDR	MTR (%)	HDR (%)	Not integrated	Heterokaryotic HR	Heterokaryoitic MTR	Non-conclusive PCR	Transformants screened
pAH035	A621	*tauD:cexA:trpC*	3556	A	0	4	0	100					4
pAH035	K621	*tauD:cexA:trpC*	3556	A	1	1	50	50					2
						sum MTR (%)	16.7						
pAH025	A621	*aacB:cexA:trpC*	3577	A	0	2	0	100					2
pAH025	K621	*aacB:cexA:trpC*	3577	A	0	1	0	100					1
						sum MTR (%)	0						
pAH030	A621	*glaA:cexA:trpC*	3592	A	0	1	0	100	1				2
pAH030	K621	*glaA:cexA:trpC*	3592	A	0	1	0	100					1
pAH036	A621	*thiA:cexA:trpC*	3592	A	0	4	0	100					4
pAH036	K621	*thiA:cexA:trpC*	3592	A	1	2	33.3	66.7					3
pAH034	A621	*rplB:cexA:trpC*	3592	A	1	2	33.3	66.7					3
pAH034	K621	*rplB:cexA:trpC*	3592	A	0	1	0	100					1
pAH026	A621	*cetA:cexA:trpC*	3592	A	0	1	0	100					1
pAH026	K621	*cetA:cexA:trpC*	3592	A	1	0	100	0					1
pAH028	A621	*gadA:cexAtrpC*	3592	A	0	3	0	100					3
pAH028	K621	*gadA:cexA:trpC*	3592	A	2	0	100	0					2
pAH029	A621	*gdpA:cexA:trpC*	3592	A	0	3	0	100					3
pAH029	K621	*gpdA:cexA:trpC*	3592	A	0	3	0	100					3
pAH032	A621	*ncpA:cexA:trpC*	3592	A	0	0	0	0	2	1			3
pAR031	A621	*gldB:cexA:trpC*	3592	A	0	0	0	0			1		1
pAR031	K621	*gldB:cexA:trpC*	3592	A	1	3	25	75					4
pAH033	A621	*pepA:cexA:trpC*	3592	A	1	1	50	50					2
pAH033	K621	*pepA:cexA:trpC*	3592	A	0	2	0	100					2
pAH027	A621	*eglA:cexA:trpC*	3592	A	0	2	0	100					2
pAH027	K621	*eglA:cexA:trpC*	3592	A	0	1	0	100					1
						sum MTR (%)	19						
pSF516	A621	*Tet-on:H2B:dsRed*	4144	B		6	0	100					6
pSF515	A621	*Tet-on:H2A:sGFP:trpC*	4165	B	1	3	25	75					4°
pSF515	K621	*Tet-on:H2A:sGFP:trpC*	4165	B	1	1	50	50				1	3
						sum MTR (%)	33.3						
pSF525	A621	*Tet-on:strep:Asp:trpC*	4178	B		2	0	100					2
pSF529	A621	*glaA:phkB:trpC*	4206	D		2	0	100					2
pSF530	A621	*cetA:phkB:trpC*	4206	D		3	0	100					3
						sum MTR (%)	0						
pSF534	A621	*glaA:phkB:6xhis:trpC*	4224	D		3	0	100					3
pSF535	A621	*cetA:phkB:6xhis:trpC*	4224	D		3	0	100					3
						sum MTR (%)	0						
pSF527	A621	*cetA:phkA_mutPAM:6xhis:trpC*	4251	C	3	1	75	25				1	5
pSF526	A621	*glaA:phkA_mutPAM:6xhis:trpC*	4251	C	1	1	50	50					2
						sum MTR (%)	66.7						
pSF533	A621	*cetA:xfspk:6xhis:trpC*	4281	E	-	2	0	100					2
pSF532	A621	*glaA:xfspk:6xhis:trpC*	4281	E	1	2	33.3	66.7					3
						sum MTR (%)	20						

pSF522	A621	*Tet-on:zwf1:trpC*	4558	B	2	2	50	50					4
pSF544	A621	*Tet-on:sGFP:cdc28:trpC*	4737	B	1	2	33,3	66,7					3
pSF544	K621	*Tet-on:sGFP:cdc28:trpC*	4737	B		3	0	100					3
						sum MTR (%)	16,7						
pSF543	A621	*Tet-on:sGFP:tubulin alpha-1:trpC*	5129	B	2	5	28,6	71,4					7
pSF543	K621	*Tet-on:sGFP:tubulin alpha-1:trpC*	5129	B		4	0	100					4
						sum MTR (%)	18,2						
pSF536	A621	*Tet-on:phkB:6xhis:trpC*	5401		0	1	0	100					1
pSF503	A621	*Tet-on:gsdA_mutPAM:trpC*	5407	B	2	14	12,5	87,5	1	3			20
pSF531	A621	*Tet-on:phkB:trpC*	5464	B	0	1	0	100					1
pSF528	A621	*Tet-on:phkA_mutPAM:6xhis:trpC*	5518	B	0	1	0	100					1
pSF523	A621	*Tet-on:xfspk:trpC*	5521	B	2	1	66,7	33,3					3*°
pSF537	A621	*Tet-on:xfspk:6xhis:trpC*	5548	B	0	1	0	100					1
pSF545	A621	*Tet-on:H2B:dsRed:tet-on:sGFP:cdc28:trpC*	8898	B	4		100	0					4
pSF545	K621	*Tet-on:H2B:dsRed:tet-on:sGFP:cdc28:trpC*	8898	B	1		100	0				1	2
						sum MTR (%)	100						

* indicates clones that were sequenced and analyzed for this study.

° indicates clones of which PCR results are presented in Figure S1.

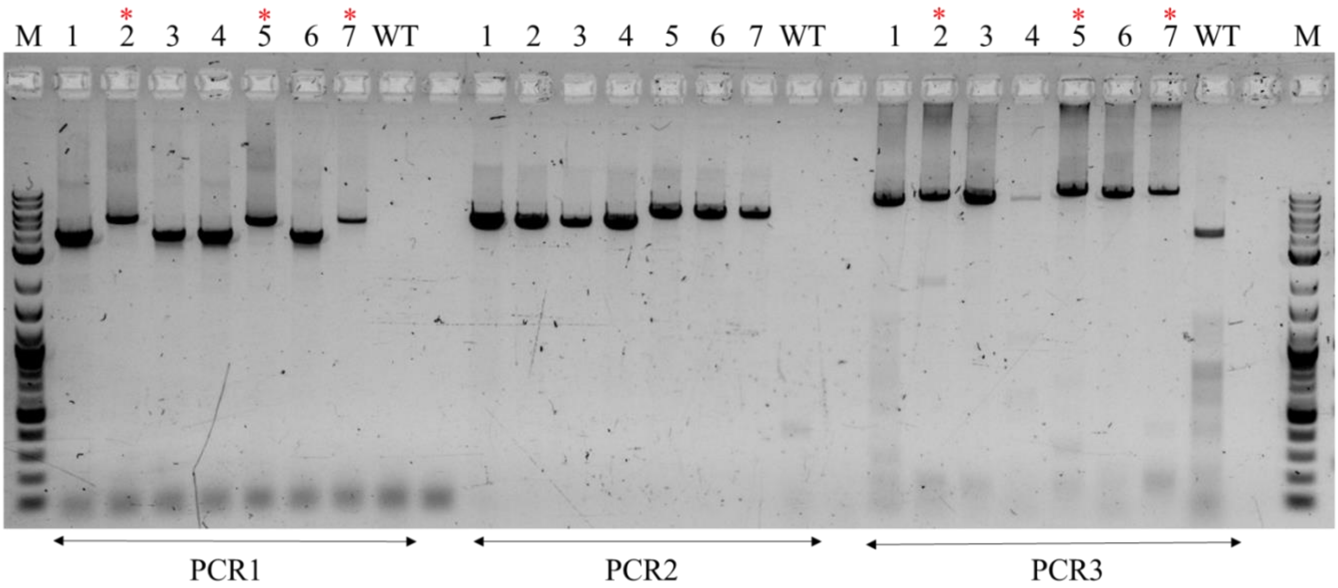

Supplementary Figure S1 Representative PCR results. PCR1, PCR2 and PCR3 were performed for verification of cassette integration at the targeted *pyrG* locus. Lanes 1-4 of each PCR are fragments amplified from clones transformed with vector pSF515. Lanes 5-7 are fragments amplified from clones transformed with vector pSF523. WT = *A. niger* ATCC 2015, M = 1 kb Plus DNA Ladder (NEB, Massachusetts, USA). Fragments of PCR1 are expected to have 3793 bp in all transformants. Fragments of PCR2 are expected to have 5852 and 6841 bp for clones derived from pSF515 and pSF523, respectively. Fragments of PCR3 are expected to have 9703 and 10692 bp for clones derived from pSF515 and pSF523, respectively. Red asterisks highlight PCR1 and PCR3 fragments that were longer than expected and indicate genomic integration via MTR.
